# Global, Regional, and National Cancer Incidence, Mortality, Years of Life Lost, Years Lived With Disability, and Disability-Adjusted Life-Years for 29 Cancer Groups, 1990 to 2016: A Systematic Analysis for the Global Burden of Disease Study

**DOI:** 10.1001/jamaoncol.2018.2706

**Published:** 2018-06-02

**Authors:** Global Burden of Disease Cancer Collaboration, Christina Fitzmaurice, Tomi F. Akinyemiju, Faris Hasan Al Lami, Tahiya Alam, Reza Alizadeh-Navaei, Christine Allen, Ubai Alsharif, Nelson Alvis-Guzman, Erfan Amini, Benjamin O. Anderson, Olatunde Aremu, Al Artaman, Solomon Weldegebreal Asgedom, Reza Assadi, Tesfay Mehari Atey, Leticia Avila-Burgos, Ashish Awasthi, Huda Omer Ba Saleem, Aleksandra Barac, James R. Bennett, Isabela M. Bensenor, Nickhill Bhakta, Hermann Brenner, Lucero Cahuana-Hurtado, Carlos A. Castañeda-Orjuela, Ferrán Catalá-López, Jee-Young Jasmine Choi, Devasahayam Jesudas Christopher, Sheng-Chia Chung, Maria Paula Curado, Lalit Dandona, Rakhi Dandona, José das Neves, Subhojit Dey, Samath D. Dharmaratne, David Teye Doku, Tim R. Driscoll, Manisha Dubey, Hedyeh Ebrahimi, Dumessa Edessa, Ziad El-Khatib, Aman Yesuf Endries, Florian Fischer, Lisa M. Force, Kyle J. Foreman, Solomon Weldemariam Gebrehiwot, Sameer Vali Gopalani, Giuseppe Grosso, Rahul Gupta, Bishal Gyawali, Randah Ribhi Hamadeh, Samer Hamidi, James Harvey, Hamid Yimam Hassen, Roderick J. Hay, Simon I. Hay, Behzad Heibati, Molla Kahssay Hiluf, Nobuyuki Horita, H. Dean Hosgood, Olayinka S. Ilesanmi, Kaire Innos, Farhad Islami, Mihajlo B. Jakovljevic, Sarah Charlotte Johnson, Jost B. Jonas, Amir Kasaeian, Tesfaye Dessale Kassa, Yousef Saleh Khader, Ejaz Ahmad Khan, Gulfaraz Khan, Young-Ho Khang, Mohammad Hossein Khosravi, Jagdish Khubchandani, Jacek A. Kopec, G. Anil Kumar, Michael Kutz, Deepesh Pravinkumar Lad, Alessandra Lafranconi, Qing Lan, Yirga Legesse, James Leigh, Shai Linn, Raimundas Lunevicius, Azeem Majeed, Reza Malekzadeh, Deborah Carvalho Malta, Lorenzo G. Mantovani, Brian J. McMahon, Toni Meier, Yohannes Adama Melaku, Mulugeta Melku, Peter Memiah, Walter Mendoza, Tuomo J. Meretoja, Haftay Berhane Mezgebe, Ted R. Miller, Shafiu Mohammed, Ali H. Mokdad, Mahmood Moosazadeh, Paula Moraga, Seyyed Meysam Mousavi, Vinay Nangia, Cuong Tat Nguyen, Vuong Minh Nong, Felix Akpojene Ogbo, Andrew Toyin Olagunju, Mahesh PA, Eun-Kee Park, Tejas Patel, David M. Pereira, Farhad Pishgar, Maarten J Postma, Farshad Pourmalek, Mostafa Qorbani, Anwar Rafay, Salman Rawaf, David Laith Rawaf, Gholamreza Roshandel, Saeid Safiri, Hamideh Salimzadeh, Juan Ramon Sanabria, Milena M. Santric Milicevic, Benn Sartorius, Maheswar Satpathy, Sadaf G. Sepanlou, Katya Anne Shackelford, Masood Ali Shaikh, Mahdi Sharif-Alhoseini, Jun She, Min-Jeong Shin, Ivy Shiue, Mark G. Shrime, Abiy Hiruye Sinke, Mekonnen Sisay, Amber Sligar, Muawiyyah Babale Sufiyan, Bryan L. Sykes, Rafael Tabarés-Seisdedos, Gizachew Assefa Tessema, Roman Topor-Madry, Tung Thanh Tran, Bach Xuan Tran, Kingsley Nnanna Ukwaja, Vasiliy Victorovich Vlassov, Stein Emil Vollset, Elisabete Weiderpass, Hywel C. Williams, Nigus Bililign Yimer, Naohiro Yonemoto, Mustafa Z. Younis, Christopher J. L. Murray, Mohsen Naghavi

**Affiliations:** 1 Division of Hematology, Department of Medicine, University of Washington, Seattle; 2 Institute for Health Metrics and Evaluation, University of Washington, Seattle; 3 Fred Hutchinson Cancer Research Center, Seattle, Washington; 4 Department of Epidemiology, University of Alabama at Birmingham; 5 Baghdad College of Medicine, Baghdad, Baghdad, Iraq; 6 Gastrointestinal Cancer Research Center, Mazandaran University of Medical Sciences, Sari, Iran; 7 Charite University Medicine Berlin, Charité Universitätsmedizin, Berlin, Berlin, Germany; 8 ALZAK Foundation–Universidad de la Costa, Universidad de Cartagena, Universidad de Cartagena, Cartagena de Indias, Colombia; 9 Endocrinology and Metabolism Population Sciences Institute, Tehran University of Medical Sciences, Tehran, Iran; 10 Uro-Oncology Research Center, Tehran University of Medical Sciences, Tehran, Iran; 11 University of Washington, Seattle; 12 Birmingham City, University Department of Public Health and Therapies, Birmingham, England; 13 University of Manitoba, Winnipeg, Manitoba, Canada; 14 Mekelle University, Mekelle, Ethiopia; 15 Mashhad University of Medical Sciences, Mashhad, Iran; 16 National Institute of Public Health, Cuernavaca, Morelos, Mexico; 17 Indian Institute of Public Health, Gandhinagar, Gujarat, India; 18 Faculty of Medicine and Health Sciences, Aden University, Aden, Yemen; 19 Faculty of Medicine, University of Belgrade, Belgrade, Belgrade, Serbia; 20 University of São Paulo, São Paulo, São Paulo, Brazil; 21 St Jude Children’s Research Hospital, Memphis, Tennessee; 22 German Cancer Research Center, Heidelberg, Germany; 23 Colombian National Health Observatory, Instituto Nacional de Salud, Bogota, Bogota, DC, Colombia; 24 Epidemiology and Public Health Evaluation Group, Public Health Department, Universidad Nacional de Colombia, Bogota, Colombia; 25 Department of Medicine, University of Valencia, INCLIVA Health Research Institute and CIBERSAM, Valencia, Spain; 26 Clinical Epidemiology Program, Ottawa Hospital Research Institute, Ottawa, ON, Canada; 27 Seoul National University Hospital, Seoul, South Korea; 28 Seoul National University Medical Library, Seoul, South Korea; 29 Christian Medical College, Vellore, Tamilnadu, India; 30 The Farr Institute of Health Informatics Research, Institute of Health Informatics, University College London, London, England; 31 Accamargo Cancer Center, Sao Paulo, Sao Paulo, Brazil; 32 International Prevention Research Institute, Ecully, France; 33 Public Health Foundation of India, Gurugram, National Capital Region, India; 34 INEB–Instituto de Engenharia Biomédica, University of Porto, Porto, Portugal; 35 i3S–Instituto de Investigação e Inovação em Saúde, University of Porto, Porto, Portugal; 36 Indian Institute of Public Health, Delhi, India; 37 Department of Community Medicine, Faculty of Medicine, University of Peradeniya, Peradeniya, Sri Lanka; 38 University of Cape Coast, Cape Coast, Ghana; 39 University of Tampere, Tampere, Finland; 40 Sydney School of Public Health, University of Sydney, Sydney, New South Wales, Australia; 41 International Institute for Population Sciences, Mumbai, Maharashtra, India; 42 Liver and Pancreaticobiliary Diseases Research Center, Digestive Disease Research Institute, Shariati Hospital, Tehran University of Medical Sciences, Tehran, Iran; 43 Haramaya University, Harar, Ethiopia; 44 Department of Global Health and Social Medicine, Harvard Medical School, Kigali, Rwanda; 45 Department of Public Health Sciences, Karolinska Institutet, Stockholm, Sweden; 46 Arba Minch University, Arba Minch, SNNPR, Ethiopia; 47 School of Public Health, Bielefeld University, Bielefeld, North Rhine-Westphalia, Germany; 48 Imperial College London, London, England; 49 College of Health Sciences, Mekelle University, Mekelle, Ethiopia; 50 Department of Health and Social Affairs, Government of the Federated States of Micronesia, Palikir, Pohnpei, Federated States of Micronesia; 51 University Hospital Policlinico “Vittorio Emanuele,” Catania, Italy; 52 NNEdPro Global Centre for Nutrition and Health, Cambridge, England; 53 West Virginia Bureau for Public Health, Charleston; 54 Aarhus University, Aarhus, Denmark; 55 Arabian Gulf University, Manama, Bahrain; 56 Haan Bin Mohammed Smart University, Dubai, United Arab Emirates; 57 Mizan Tepi University, Mizan Teferi, Ethiopia; 58 International Foundation for Dermatology, London, England; 59 King's College London, London, England; 60 Oxford Big Data Institute, Li Ka Shing Centre for Health Information and Discovery, University of Oxford, Oxford, England; 61 Air Pollution Research Center, Iran University of Medical Sciences, Tehran, Iran; 62 Samara University, Samara, Ethiopia; 63 Department of Pulmonology, Yokohama City University Graduate School of Medicine, Yokohama, Kanagawa, Japan; 64 Albert Einstein College of Medicine, Bronx, New York, USA; 65 National Public Health Institute, Monrovia, Monserrado County, Liberia; 66 National Institute for Health Development, Tallinn, Estonia; 67 Surveillance and Health Services Research, American Cancer Society, Atlanta, Georgia; 68 Faculty of Medical Sciences, University of Kragujevac, Kragujevac, Central Serbia, Serbia; 69 Center for Health Trends and Forecasts, University of Washington, Seattle; 70 Department of Ophthalmology, Medical Faculty Mannheim, Ruprecht-Karls-University Heidelberg, Mannheim, Germany; 71 Hematologic Malignancies Research Center, Tehran University of Medical Sciences, Tehran, Iran; 72 Hematology-Oncology and Stem Cell Transplantation Research Center, Tehran University of Medical Sciences, Tehran, Iran; 73 Department of Community Medicine, Public Health and Family Medicine, Jordan University of Science and Technology, Irbid, Jordan; 74 Health Services Academy, Islamabad, Punjab, Pakistan; 75 Department of Microbiology and Immunology, College of Medicine & Health Sciences, United Arab Emirates University, Al Ain, Abu Dhabi, United Arab Emirates; 76 Department of Health Policy and Management, Seoul National University College of Medicine, Seoul, South Korea; 77 Institute of Health Policy and Management, Seoul National University Medical Center, Seoul, South Korea; 78 Baqiyatallah University of Medical Sciences, Tehran, Iran; 79 International Otorhinolaryngology Research Association (IORA), Universal Scientific Education and Research Network (USERN), Tehran, Iran; 80 Department of Nutrition and Health Science, Ball State University, Muncie, Indiana; 81 University of British Columbia, Vancouver, British Columbia, Canada; 82 Post Graduate Institute of Medical Education and Research, Chandigarh, India; 83 University of Milano Bicocca, Monza, MB, Italy; 84 National Cancer Institute, Rockville, Maryland; 85 University of Sydney, Sydney, New South Wales, Australia; 86 University of Haifa, Haifa, Israel; 87 Aintree University Hospital National Health Service Foundation Trust, Liverpool, England; 88 School of Medicine, University of Liverpool, Liverpool, England; 89 Department of Primary Care & Public Health, Imperial College London, London, England; 90 Digestive Diseases Research Institute, Tehran University of Medical Sciences, Tehran, Iran; 91 Universidade Federal de Minas Gerais, Belo Horizonte, Minas Gerais, Brazil; 92 Alaska Native Tribal Health Consortium, Anchorage; 93 Competence Cluster for Nutrition and Cardiovascular Health (nutriCARD), Martin Luther University Halle-Wittenberg, Saale, Germany; 94 School of Medicine, University of Adelaide, Adelaide, South Australia, Australia; 95 School of Public Health, Mekelle University, Mekelle, Ethiopia; 96 University of Gondar, Gondar, Ethiopia; 97 University of West Florida, Pensacola, Florida; 98 United Nations Population Fund, Lima, Peru; 99 Comprehensive Cancer Center, Breast Surgery Unit, Helsinki University Hospital, Helsinki, Finland; 100 University of Helsinki, Helsinki, Finland; 101 Pacific Institute for Research & Evaluation, Calverton, Maryland; 102 School of Public Health, Curtin University, Perth, Western Australia, Australia; 103 Health Systems and Policy Research Unit, Ahmadu Bello University, Zaria, Nigeria; 104 Institute of Public Health, Heidelberg University, Heidelberg, Baden Wuettemberg, Germany; 105 Health Science Research Center, Addiction Institute, Mazandaran University of Medical Sciences, Sari, Iran; 106 Lancaster Medical School, Lancaster University, Lancaster, England; 107 Department of Health Management and Economics, School of Public Health, Tehran University of Medical Sciences, Tehran, Iran; 108 Suraj Eye Institute, Nagpur, Maharashtra, India; 109 Institute for Global Health Innovations, Duy Tan University, Da Nang, Vietnam; 110 Centre for Health Research, Western Sydney University, Sydney, New South Wales, Australia; 111 Department of Psychiatry, College of Medicine, University of Lagos, Lagos, Lagos State, Nigeria; 112 Department of Psychiatry, Lagos University Teaching Hospital, Lagos, Nigeria; 113 Discipline of Psychiatry, University of Adelaide, Adelaide, South Australia, Australia; 114 JSS Medical College (PA), JSS University, Mysore, Karnataka, India; 115 Department of Medical Humanities and Social Medicine, College of Medicine, Kosin University, Busan, South Korea; 116 White Plains Hospital, White Plains, New York; 117 REQUIMTE/LAQV, Laboratório de Farmacognosia, Departamento de Química, Faculdade de Farmácia, Universidade do Porto, Porto, Portugal; 118 Non-Communicable Diseases Research Center, Tehran University of Medical Sciences, Tehran, Iran; 119 University Medical Center Groningen, Groningen, the Netherlands; 120 University of Groningen, Groningen, the Netherlands; 121 Non-Communicable Diseases Research Center, Alborz University of Medical Sciences, Karaj, Iran; 122 Contech International Health Consultants, Lahore, Pakistan; 123 Contech School of Public Health, Lahore, Pakistan; 124 North Hampshire Hospitals, Basingstroke, England; 125 University College London Hospitals, London, England; 126 WHO Collaborating Centre, Imperial College of London, London, England; 127 Golestan Research Center of Gastroenterology and Hepatology, Golestan University of Medical Sciences, Gorgan, Iran; 128 Managerial Epidemiology Research Center, Department of Public Health, School of Nursing and Midwifery, Maragheh University of Medical Sciences, Maragheh, Iran; 129 Tehran University of Medical Sciences, Tehran, Iran; 130 Joan C. Edwards School of Medicine, Marshall University, Huntington, West Virginia; 131 Case Western Reserve University, Cleveland, Ohio; 132 Centre School of Public Health and Health Management, Faculty of Medicine, University of Belgrade, Belgrade, Belgrade, Serbia; 133 Institute of Social Medicine, Faculty of Medicine, University of Belgrade, Belgrade, Belgrade, Serbia; 134 Public Health Medicine, School of Nursing and Public Health, University of KwaZulu-Natal, Durban, South Africa; 135 UKZN Gastrointestinal Cancer Research Centre, South African Medical Research Council, Durban, South Africa; 136 Centre of Advanced Study in Psychology, Utkal University, Bhubaneswar, India; 137 Independent Consultant, Karachi, Pakistan; 138 Sina Trauma and Surgery Research Center, Tehran University of Medical Sciences, Tehran, Iran; 139 Department of Pulmonary Medicine, Zhongshan Hospital (She), Fudan University, Shanghai, China; 140 Department of Public Health Sciences, Korea University, Seoul, South Korea; 141 Alzheimer Scotland Dementia Research Centre, University of Edinburgh, Edinburgh, Scotland; 142 Institut für Medizinische Epidemiologie, Biometrie und Informatik, Martin Luther University Halle-Wittenberg, Saale, Germany; 143 Harvard Medical School, Kigali, Rwanda; 144 Ethiopian Medical Association, Addis Ababa, Ethiopia; 145 Ahmadu Bello University, Zaria, Nigeria; 146 Departments of Criminology, Law & Society, Sociology, and Public Health, University of California, Irvine; 147 University of Adelaide, Adelaide, South Australia, Australia; 148 Institute of Public Health, Faculty of Health Sciences, Jagiellonian University Medical College, Kraków, Poland; 149 Faculty of Health Sciences, Wroclaw Medical University, Wroclaw, Poland; 150 Johns Hopkins University, Baltimore, Maryland; 151 Hanoi Medical University, Hanoi, Vietnam; 152 Department of Internal Medicine, Federal Teaching Hospital, Abakaliki, Ebonyi State, Nigeria; 153 National Research University Higher School of Economics, Moscow, Russia; 154 Department of Research, Cancer Registry of Norway, Institute of Population-Based Cancer Research, Oslo, Norway; 155 Department of Community Medicine, Faculty of Health Sciences, University of Tromsø, The Arctic University of Norway, Tromsø, Norway; 156 Genetic Epidemiology Group, Folkhälsan Research Center, Helsinki, Finland; 157 Department of Medical Epidemiology and Biostatistics, Karolinska Institutet, Stockholm, Sweden; 158 Centre of Evidence-Based Dermatology, University of Nottingham, Nottingham, England; 159 Woldia University, Woldia, Amhara, Ethiopia; 160 Department of Biostatistics, School of Public Health, Kyoto University, Kyoto, Japan; 161 Jackson State University, Jackson, Mississippi

## Abstract

**Importance:**

The increasing burden due to cancer and other noncommunicable diseases poses a threat to human development, which has resulted in global political commitments reflected in the Sustainable Development Goals as well as the World Health Organization (WHO) Global Action Plan on Non-Communicable Diseases. To determine if these commitments have resulted in improved cancer control, quantitative assessments of the cancer burden are required.

**Objective:**

To assess the burden for 29 cancer groups over time to provide a framework for policy discussion, resource allocation, and research focus.

**Evidence Review:**

Cancer incidence, mortality, years lived with disability, years of life lost, and disability-adjusted life-years (DALYs) were evaluated for 195 countries and territories by age and sex using the Global Burden of Disease study estimation methods. Levels and trends were analyzed over time, as well as by the Sociodemographic Index (SDI). Changes in incident cases were categorized by changes due to epidemiological vs demographic transition.

**Findings:**

In 2016, there were 17.2 million cancer cases worldwide and 8.9 million deaths. Cancer cases increased by 28% between 2006 and 2016. The smallest increase was seen in high SDI countries. Globally, population aging contributed 17%; population growth, 12%; and changes in age-specific rates, −1% to this change. The most common incident cancer globally for men was prostate cancer (1.4 million cases). The leading cause of cancer deaths and DALYs was tracheal, bronchus, and lung cancer (1.2 million deaths and 25.4 million DALYs). For women, the most common incident cancer and the leading cause of cancer deaths and DALYs was breast cancer (1.7 million incident cases, 535 000 deaths, and 14.9 million DALYs). In 2016, cancer caused 213.2 million DALYs globally for both sexes combined. Between 2006 and 2016, the average annual age-standardized incidence rates for all cancers combined increased in 130 of 195 countries or territories, and the average annual age-standardized death rates decreased within that timeframe in 143 of 195 countries or territories.

**Conclusions and Relevance:**

Large disparities exist between countries in cancer incidence, deaths, and associated disability. Scaling up cancer prevention and ensuring universal access to cancer care are required for health equity and to fulfill the global commitments for noncommunicable disease and cancer control.

## Key Points

**Question:**

What is the cancer burden over time at the global and national levels measured in incidence, mortality, years lived with disability, years of life lost, and disability-adjusted life-years (DALYs)?

**Findings:**

In this systematic analysis, in 2016 there were 17.2 million incident cancer cases, 8.9 million deaths, and 213.2 million DALYs due to cancer worldwide. Between 2006 and 2016, incident cases increased by 28%, with the largest increase occurring in the least developed countries.

**Meaning:**

To achieve the Sustainable Development Goals as well as targets set in the World Health Organization Global Action Plan on noncommunicable diseases, cancer control planning and implementation as well as strategic investments are urgently needed.

## Teaser

This systematic analysis evaluates the cancer burden over time at the global and national levels measured in incidence, mortality, years lived with disability, years of life lost, and disability-adjusted life-years.

## Introduction

The year 2017 marked another milestone in the global commitment to control cancer. In May 2017, a new cancer resolution was adopted during the 70th World Health Assembly,^1^ suggesting that focused efforts are urgently needed to achieve the goals for the 2011 Political Declaration on the Prevention and Control of NCDs (noncommunicable diseases)^2,3^ (25% reduction in premature mortality from NCDs) as well as for the third Sustainable Development Goal (“by 2030 reduce by one-third premature mortality from non-communicable diseases [NCDs] through prevention and treatment, and promote mental health and wellbeing”).^4^ Even with increased awareness of the threat that NCDs pose to human development, progress on NCD control has been slow in most countries.^5^ This is despite the fact that we are now entering a time when reductions in mortality would be expected if the 2011 declaration had led to policy changes.^5,6^ Compared with other health threats like human immunodeficiency virus, tuberculosis, or malaria, cancer represents many drastically different diseases that require unique approaches for prevention, diagnosis, and treatment. Thus far, few countries have been able to overcome this challenge. To achieve equitable cancer control over the next decade, continued commitment from all stakeholders, appropriate funding, and effective approaches are necessary. The Global Burden of Disease (GBD) study provides data to direct efforts where they are most needed and to identify progress and obstacles in cancer control.

In this study, we describe the burden of cancer using results from the GBD 2016 study for 29 cancer groups by sex, age, and over time for 195 countries or territories.

## Methods

Methods have remained similar to the GBD 2015 study.^7^ As in each prior GBD study, the entire time series was reestimated, and results presented in this study supersede prior GBD studies. All cancers as defined in the *International Classification of Diseases* (*ICD*) were categorized into 29 cancer groups. Changes since GBD 2015 include new data additions, the addition of “other leukemia” as a cause, changes in the mortality-to-incidence ratio (MIR) estimation, as well as reporting estimates for nonmelanoma skin cancer (NMSC). For GBD 2016, we estimated national disease burden for 195 countries and territories. Descriptions of the methods can be found in the GBD 2016 publications as well as in the eAppendix, eFigures, and eTables in the Supplement.^8,9,10,11^ The GBD 2016 study is compliant with GATHER guidelines (eTable 1 in the Supplement). All rates are reported per 100 000 person-years. The GBD world population standard was used for the calculation of age-standardized rates.^12^ We report 95% uncertainty intervals (UIs) for all estimates.

### Estimation Framework

The GBD estimation process starts with cancer mortality. Data sources for cancer mortality include vital registration system (83% of data), cancer registry (14.4% of data), and verbal autopsy data (3% of data) (eTable 2 in the Supplement). Since cancer registries often exist in locations without cancer mortality data, cancer incidence data are used to model mortality by multiplying incidence with a separately modeled MIR. These mortality estimates are added to mortality data from the other sources and used in a cause of death ensemble model (CODEm).^8,13^ Each cancer type is estimated separately using covariates with a causal connection (eTable 8 in the Supplement). Final cancer-specific mortality estimates are divided by the MIR to estimate cancer incidence. Ten-year cancer prevalence is modeled using the MIR as a scalar to determine country-specific survival. Years lived with disability (YLDs) are estimated by dividing 10-year cancer prevalence into 4 sequelae: (1) diagnosis/treatment, (2) remission, (3) metastasic/disseminated, and (4) terminal phase. Each sequela prevalence is multiplied by a disability weight to estimate YLDs. For larynx, breast, colorectal, bladder, and prostate cancer, additional disability is estimated from procedures related to these cancers. Years of life lost (YLLs) are estimated by multiplying the estimated number of deaths by age with a standard life expectancy at that age.^14^ Disability-adjusted life-years (DALYs) are calculated by summing YLDs and YLLs. As in GBD 2015, we estimate the contribution of population aging, population growth, and change in age-specific rates on the change in incident cases between 2006 and 2016.^7^ We stratify results using Sociodemographic Index (SDI) quintiles. The SDI is a composite indicator including fertility, education, and income, and it has been shown to correlate well with health outcomes.^7^

## Results

### Global Incidence, Mortality, and DALYs

In 2016, there were 17.2 million (95% UI, 16.7-17.8 million) incident cancer cases worldwide and 8.9 million (95% UI, 8.8-9.1 million) cancer deaths (Table). Cancer caused 213.2 million (95% UI, 208.5-217.6 million) DALYs in 2016, of which 98% came from YLLs and 2% came from YLDs (eTable 15 and eFigure 4 in the Supplement). Globally, the odds of developing cancer during a lifetime (age 0-79 years) differed by sex: they were 1 in 3 for men and 1 in 5 for women (eTable 16 in the Supplement). These odds differ substantially among SDI quintiles ranging from 1 in 8 at the lowest SDI quintile to 1 in 2 at the highest SDI quintile for men and from 1 in 8 in the lowest SDI quintile to 1 in 3 in the highest quintile for women. In 2016, prostate, TBL (tracheal, bronchus, and lung), and colorectal cancer were the most common incident cancers in men—accounting for 40% of all cancer cases. The most common causes of cancer deaths for men were TBL, liver, and stomach cancer (Table). The leading causes for cancer DALYs in 2016 were TBL, liver, and stomach cancer (Web Table 3; available at http://ghdx.healthdata.org/node/350478). For women in 2016, the most common incident cancers were breast, colorectal, and NMSC accounting for 40% of all incident cases. The leading causes of cancer deaths and DALYs were breast, TBL, and colorectal cancer (Web Table 3; http://ghdx.healthdata.org/node/350478).

**Table.** 2016 Global Incidence and Deaths for All Cancers and 29 Specified Cancer Groups^a^

Cancer Type^b^	Incident Cases, Thousands^c^			ASIR^c^		Deaths, Thousands^c^			ASDR^c^	
	Total	Male	Female	Male	Female	Total	Male	Female	Male	Female
All neoplasms	17 228 (16 713-17 803)	9427 (9128-9794)	7800 (7538-8099)	306.8 (296.5-319.4)	213.9 (206.8-222.0)	8927 (8755-9089)	5172 (5054-5289)	3755 (3645-3862)	171.9 (167.9-175.7)	103.8 (100.8-106.8)
Lip and oral cavity	382 (371-392)	234 (224-244)	148 (145-151)	7.1 (6.8-7.4)	4.0 (4.0-4.1)	176 (169-183)	118 (112-124)	59 (56-62)	3.7 (3.5-3.8)	1.6 (1.5-1.7)
Nasopharynx	96 (91-101)	71 (67-76)	25 (23-26)	2.0 (1.9-2.1)	0.7 (0.6-0.7)	64 (61-67)	47 (44-50)	17 (16-17)	1.4 (1.3-1.5)	0.4 (0.4-0.5)
Other pharynx	170 (159-176)	128 (119-134)	42 (40-44)	3.8 (3.5-4.0)	1.1 (1.1-1.2)	119 (109-125)	87 (79-92)	32 (29-34)	2.6 (2.4-2.8)	0.9 (0.8-0.9)
Esophageal	443 (433-456)	321 (312-333)	122 (118-125)	10.2 (10.0-10.6)	3.4 (3.3-3.5)	415 (404-427)	306 (296-318)	108 (105-112)	9.9 (9.6-10.3)	3.0 (2.9-3.1)
Stomach	1157 (1134-1180)	766 (745-787)	391 (383-401)	25.0 (24.3-25.7)	10.8 (10.6-11.1)	834 (814-855)	536 (520-553)	298 (288-310)	17.9 (17.3-18.4)	8.3 (8.0-8.6)
Colon and rectum	1716 (1658-1795)	952 (918-1001)	763 (733-799)	31.6 (30.4-33.2)	21.2 (20.3-22.2)	830 (797-860)	450 (430-469)	380 (362-399)	15.5 (14.8-16.2)	10.5 (10.0-11.1)
Liver	1008 (953-1042)	736 (694-763)	272 (249-300)	22.3 (21.0-23.1)	7.5 (6.9-8.3)	829 (796-858)	590 (563-614)	239 (218-263)	18.3 (17.5-19.0)	6.6 (6.1-7.3)
Gallbladder and biliary tract	184 (169-193)	76 (62-84)	108 (104-112)	2.6 (2.1-2.9)	3.0 (2.9-3.1)	162 (149-171)	67 (54-75)	95 (90-99)	2.3 (1.9-2.6)	2.6 (2.5-2.7)
Pancreatic	418 (406-425)	219 (213-224)	198 (192-203)	7.3 (7.0-7.4)	5.5 (5.4-5.7)	405 (394-416)	213 (206-220)	192 (185-200)	7.1 (6.9-7.3)	5.4 (5.2-5.6)
Larynx	187 (184-191)	162 (159-167)	25 (24-25)	5.0 (4.9-5.1)	0.7 (0.7-0.7)	111 (108-115)	95 (92-99)	16 (15-16)	3.0 (2.9-3.1)	0.4 (0.4-0.5)
Tracheal, bronchus, and lung	2008 (1958-2055)	1369 (1328-1404)	638 (616-656)	44.9 (43.6-46.1)	17.8 (17.1-18.3)	1707 (1659-1753)	1177 (1135-1216)	530 (510-547)	39.1 (37.7-40.4)	14.8 (14.2-15.2)
Malignant skin melanoma	282 (243-314)	152 (136-164)	129 (99-153)	4.8 (4.3-5.1)	3.5 (2.7-4.2)	62 (54-67)	34 (30-37)	28 (22-32)	1.1 (1.0-1.2)	0.8 (0.6-0.9)
Nonmelanoma skin cancer	1521 (1109-2008)	848 (613-1159)	673 (490-884)	29.1 (21.2-40.0)	18.6 (13.6-24.4)	53 (51-55)	35 (34-37)	18 (17-19)	1.3 (1.2-1.3)	0.5 (0.5-0.5)
Nonmelanoma skin cancer (SCC)	635 (386-922)	397 (242-619)	238 (146-334)	14.3 (8.7-22.1)	6.6 (4.0-9.3)	53 (51-55)	35 (34-37)	18 (17-19)	1.3 (1.2-1.3)	0.5 (0.5-0.5)
Nonmelanoma skin cancer (BCC)	886 (574-1262)	451 (293-645)	436 (283-617)	14.9 (9.6-21.5)	12.0 (7.8-17.1)	NA	NA	NA	NA	NA
Breast	1702 (1629-1801)	20 (15-22)	1682 (1608-1780)	0.6 (0.5-0.7)	45.6 (43.6-48.2)	546 (517-582)	10 (7-11)	535 (506-573)	0.3 (0.2-0.4)	14.6 (13.8-15.6)
Cervical	511 (414-542)	NA	511 (414-542)	NA	13.7 (11.1-14.5)	247 (204-263)	NA	247 (204-263)	NA	6.7 (5.6-7.2)
Uterine	417 (401-442)	NA	417 (401-442)	NA	11.4 (10.9-12.0)	88 (83-92)	NA	88 (83-92)	NA	2.4 (2.3-2.6)
Ovarian	254 (242-260)	NA	254 (242-260)	NA	6.9 (6.6-7.1)	165 (157-173)	NA	165 (157-173)	NA	4.5 (4.3-4.7)
Prostate	1436 (1293-1619)	1436 (1293-1619)	NA	49.9 (45.0-56.1)	NA	381 (321-413)	381 (321-413)	NA	14.9 (12.7-16.2)	NA
Testicular	67 (64-70)	67 (64-70)	NA	1.8 (1.7-1.8)	NA	9 (8-9)	9 (8-9)	NA	0.2 (0.2-0.3)	NA
Kidney	342 (331-350)	211 (203-218)	131 (127-134)	6.5 (6.3-6.7)	3.6 (3.5-3.7)	132 (127-136)	86 (82-89)	46 (44-48)	2.9 (2.8-3.0)	1.3 (1.2-1.3)
Bladder	437 (427-448)	334 (325-342)	103 (99-107)	11.5 (11.2-11.8)	2.9 (2.7-3.0)	186 (180-192)	138 (133-142)	48 (46-50)	5.1 (4.9-5.3)	1.3 (1.3-1.4)
Brain and nervous system	330 (299-349)	175 (152-191)	155 (136-168)	5.1 (4.4-5.5)	4.2 (3.7-4.6)	227 (205-241)	128 (111-141)	99 (86-107)	3.8 (3.3-4.1)	2.7 (2.4-2.9)
Thyroid	238 (229-253)	76 (72-80)	162 (155-174)	2.2 (2.1-2.3)	4.4 (4.2-4.7)	43 (41-45)	17 (16-18)	26 (25-27)	0.6 (0.5-0.6)	0.7 (0.7-0.8)
Mesothelioma	35 (33-36)	24 (22-26)	10 (10-11)	0.8 (0.7-0.8)	0.3 (0.3-0.3)	30 (28-32)	22 (20-24)	8 (8-9)	0.7 (0.7-0.8)	0.2 (0.2-0.2)
Hodgkin lymphoma	73 (66-82)	45 (40-54)	28 (25-32)	1.2 (1.1-1.5)	0.8 (0.7-0.9)	29 (25-34)	19 (16-23)	10 (8-12)	0.5 (0.5-0.7)	0.3 (0.2-0.3)
Non-Hodgkin lymphoma	461 (428-482)	260 (232-285)	201 (190-207)	8.1 (7.3-8.9)	5.5 (5.3-5.7)	240 (221-248)	139 (123-146)	100 (96-104)	4.5 (4.0-4.7)	2.8 (2.7-2.9)
Multiple myeloma	139 (121-155)	75 (62-85)	64 (54-76)	2.4 (2.0-2.8)	1.8 (1.5-2.1)	98 (87-110)	51 (42-58)	47 (41-55)	1.7 (1.4-2.0)	1.3 (1.1-1.5)
Leukemia	467 (423-489)	269 (242-280)	197 (167-213)	8.4 (7.6-8.7)	5.5 (4.6-5.9)	310 (286-324)	180 (165-194)	130 (113-139)	5.8 (5.3-6.2)	3.6 (3.1-3.8)
Acute lymphoid leukemia	76 (66-80)	44 (38-47)	32 (25-35)	1.2 (1.1-1.3)	0.9 (0.7-1.0)	51 (46-56)	31 (28-34)	20 (17-24)	0.9 (0.8-1.0)	0.6 (0.5-0.7)
Chronic lymphoid leukemia	105 (98-113)	61 (56-70)	45 (40-48)	2.0 (1.9-2.3)	1.2 (1.1-1.3)	35 (33-40)	21 (19-25)	15 (13-16)	0.8 (0.7-0.9)	0.4 (0.4-0.4)
Acute myeloid leukemia	103 (91-108)	58 (49-63)	45 (38-48)	1.8 (1.5-1.9)	1.2 (1.1-1.3)	85 (78-90)	49 (44-54)	36 (32-39)	1.6 (1.4-1.7)	1.0 (0.9-1.1)
Chronic myeloid leukemia	32 (29-34)	19 (17-20)	14 (12-15)	0.6 (0.5-0.6)	0.4 (0.3-0.4)	22 (20-24)	12 (11-14)	10 (8-11)	0.4 (0.4-0.5)	0.3 (0.2-0.3)
Other leukemia	150 (127-161)	87 (73-93)	63 (48-70)	2.7 (2.3-2.9)	1.7 (1.3-1.9)	117 (103-123)	67 (59-74)	49 (40-53)	2.2 (1.9-2.3)	1.4 (1.1-1.5)
Other neoplasms	750 (682-772)	399 (349-414)	352 (328-362)	12.3 (10.8-12.8)	9.7 (9.0-9.9)	431 (393-444)	236 (205-246)	195 (182-201)	7.5 (6.6-7.8)	5.4 (5.0-5.6)

Abbreviations: ASDR, age-standardized death rate per 100 000 person-years; ASIR, age-standardized incidence rate per 100 000 person-years; BCC, basal cell carcinoma; NA, not applicable; SCC, squamous cell carcinoma; UI, uncertainty interval.

^a^ All data reported as number or rate (95% UI).

^b^ Cancer groups are defined based on *International Classification of Diseases, Ninth Revision* (*ICD-9*), and *International Statistical Classification of Diseases and Related Health Problems, Tenth Revision* (*ICD-10*) codes and include all codes pertaining to neoplasms (*ICD-9*, 140-208; *ICD-10*, C00-C96) except for Kaposi sarcoma (C46). eTables 4 and 5 in the Supplement detail how the original *ICD* codes were mapped to the standardized Global Burden of Disease cause list.

^c^ Detailed results for incidence, mortality, and disability-adjusted life-years for the global level, by Sociodemographic Index quintile, region, and country can be accessed in Web Tables 3-5 (http://ghdx.healthdata.org/node/350478) as well as at https://vizhub.healthdata.org/gbd-compare/.

**Figure 1.**
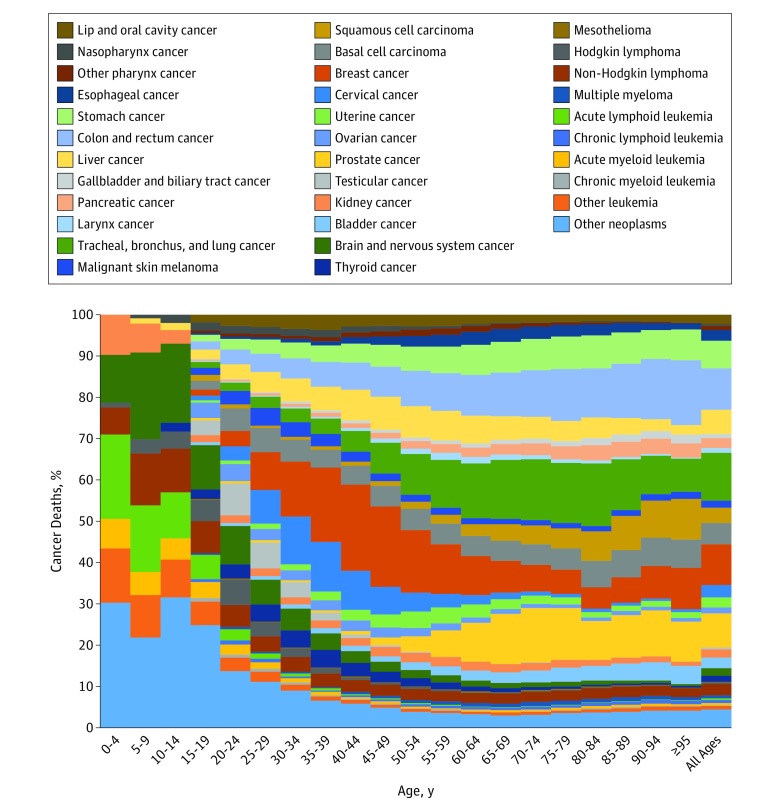
Age-Specific Global Contributions of Cancer Types to Total Cancer Incidence, Both Sexes, 2016

**Figure 2.**
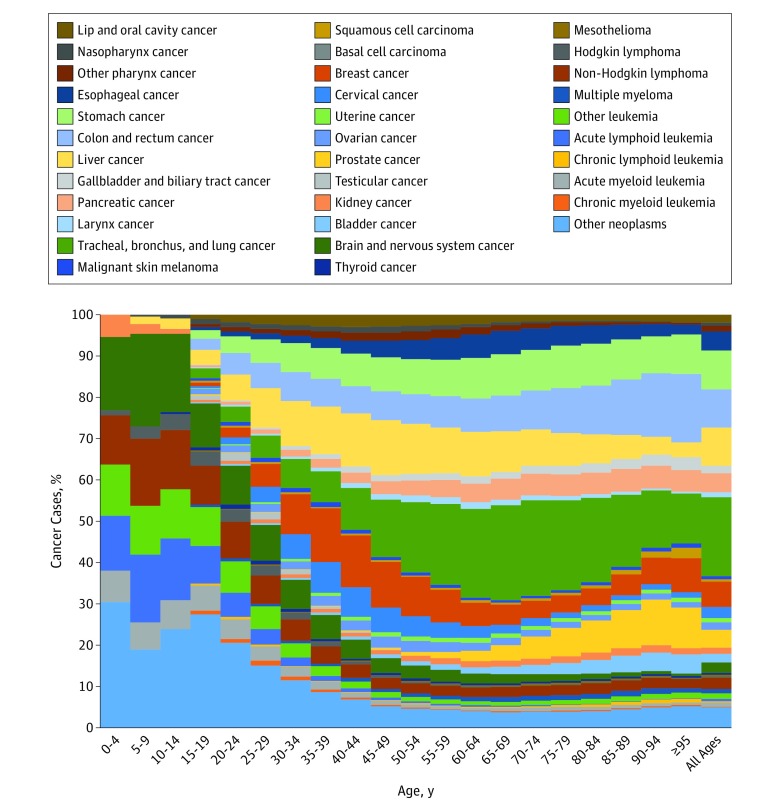
Age-Specific Global Contributions of Cancer Types to Total Cancer Mortality, Both Sexes, 2016

For childhood cancers (age 0-19 years), the most common cancers and causes of cancer deaths were other neoplasms (see eTables 4 and 5 in the Supplement for *ICD* codes included in “other neoplasms”), brain and nervous system cancers, and acute lymphoid leukemia (Figure 1 and Figure 2). For adolescents and young adults (age 20-39 years) the most common cancers globally were breast cancer, cervical cancer, and other neoplasms. The main causes of cancer deaths for this age group were other neoplasms, brain and nervous system cancers, and non-Hodgkin lymphoma. For the population older than 39 years, the cancers contributing the most incident cases were TBL, breast, prostate, and colorectal cancer, while the main contributors to cancer deaths were TBL, colorectal, and stomach cancer.

**Figure 3.**
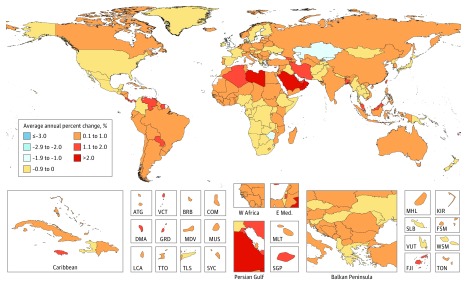
Average Annual Percent Change in Age-Standarized Incidence Rate in Both Sexes for All Cancers From 2006 to 2016 ATG indicates Antigua and Barbuda; BRB, Barbados; COM, Comoros; DMA, Dominica; E Med: Eastern Mediterranean; FJI, Fiji; FSM, Federated States of Micronesia; GRD, Grenada; KIR, Kiribati; KS, Kaposi sarcoma; LCA, Saint Lucia; MDV, Maldives; MLT, Malta; MUS, Mauritius; MHL, Marshall Islands; NMSC, nonmelanoma skin cancer; SGP, Singapore; SLB, Solomon Islands; SYC, Seychelles; TLS, Timor-Leste; TON, Tonga; TTO, Trinidad and Tobago; VCT, Saint Vincent and the Grenadines; VUT, Vanuatu; W Africa, West Africa; WSM, Samoa.

**Figure 4.**
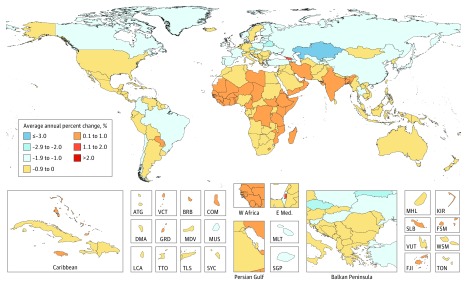
Average Annual Percent Change in Age-Standarized Mortality Rate in Both Sexes for All Cancers From 2006 to 2016 ATG indicates Antigua and Barbuda; BRB, Barbados; COM, Comoros; DMA, Dominica; E Med: Eastern Mediterranean; FJI, Fiji; FSM, Federated States of Micronesia; GRD, Grenada; KIR, Kiribati; KS, Kaposi sarcoma; LCA, Saint Lucia; MDV, Maldives; MLT, Malta; MUS, Mauritius; MHL, Marshall Islands; NMSC, nonmelanoma skin cancer; SGP, Singapore; SLB, Solomon Islands; SYC, Seychelles; TLS, Timor-Leste; TON, Tonga; TTO, Trinidad and Tobago; VCT, Saint Vincent and the Grenadines; VUT, Vanuatu; W Africa, West Africa; WSM, Samoa.

Between 2006 and 2016, the average annual age-standardized incidence rates (AAASIRs) forall cancers combined increased in 130 of 195 countries (Figure 3). In contrast, the average annual age-standardized death rates (AAASDRs) for all cancers combined decreased within that timeframe in 143 of 195 countries (Figure 4). Countries with an increase in AAASDR were largely located on the African continent and Middle East. Between 2006 and 2016 the AAASDR decreased in all SDI quintiles except for the low SDI quintile (eFigure 5 in the Supplement). The AAASDR decreased for most cancers in the high, and high-middle SDI quintiles, whereas the changes in AAASDR were more heterogeneous for the other SDI quintiles (eFigures 5-14 in the Supplement).

Incident cases for both sexes combined increased in all SDI quintiles between 2006 and 2016 for nearly all cancers (eTable 14 in the Supplement and Web Table 1; http://ghdx.healthdata.org/node/350478). The largest increase in cancer incident cases between 2006 and 2016 occurred in middle SDI countries, with a 38% increase, of which changing age structure contributed 25%; population growth, 7%; and changing age-specific incidence rates, 6%. The drivers behind increasing cancer incidence differed substantially by SDI. Whereas in the lowest SDI quintile, population growth was the major contributor to the increase in total cancer incidence, in low-middle SDI countries, population growth and aging contributed almost equally (16.6% and 15.3%, respectively), and in high-middle, and high-income countries, increased incidence was mainly driven by population aging (eTable 14 in the Supplement).

### Global Top 10 Cancers in 2016

**Figure 5.**
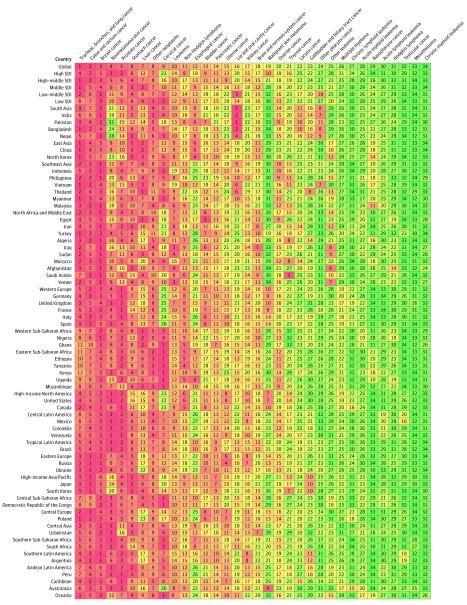
Cancers Ranked by Number of Incident Cases in Both Sexes, Globally, by Sociodemographic Index Status, and in the 50 Most Populous Countries, 2016

The global top 10 cancers were ranked by the highest number of incident cases, excluding “other neoplasms” (Figure 5).

#### 1. Tracheal, Bronchus, and Lung Cancer

**Figure 6.**
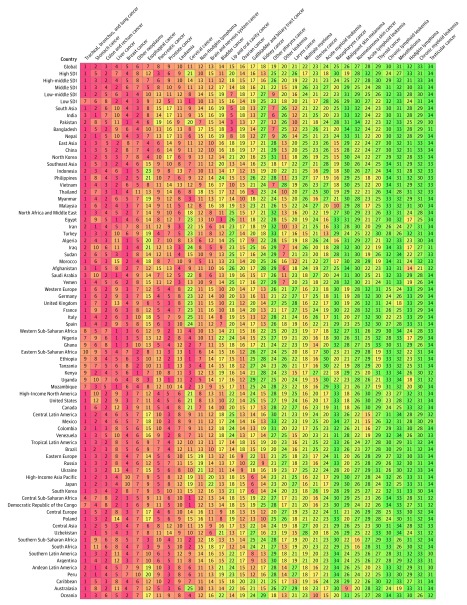
Cancers Ranked by Number of Deaths in Both Sexes, Globally, by Sociodemographic Index Status, and in the 50 Most Populous Countries, 2016

In 2016, there were 2.0 million (95% UI, 2.0-2.1 million) incident cases of TBL cancer and 1.7 million (95% UI, 1.7-1.8 million) deaths; TBL cancer caused 36.4 million (95% UI, 35.4-37.5 million) DALYs in 2016, of which 99% came from YLLs and 1% from YLDs (eTable 15 and eFigure 4 in the Supplement). Men were more likely to develop TBL cancer over a lifetime than women (1 in 18 men, 1 in 46 women) (eTable 16 in the Supplement). The odds were the highest in high SDI countries (1 in 14 men, 1 in 26 women). In low SDI countries, the odds were substantially lower (1 in 75 men, 1 in 172 women); TBL cancer was the leading cause of cancer globally and in high-middle and middle SDI countries (Figure 5). It was the most common cause of cancer deaths by absolute cases globally as well as in all SDI quintiles, except for the low SDI quintile, where TBL cancer ranked seventh (Figure 6). For men, TBL cancer was the most common incident cancer in 42 countries and the most common cause for cancer deaths in 108 countries (eFigures 17 and 19 in the Supplement). For women, TBL cancer was the most common incident cancer in 3 countries and the most common cause of cancer deaths in 25 countries (eFigures 18 and 20 in the Supplement).

Between 2006 and 2016, TBL cancer cases increased by 28% (95% UI, 25%-32%) (Web Table 1; http://ghdx.healthdata.org/node/350478). Changing age structure contributed 19%, and population growth, 12%. A decrease in age-specific incidence partially offset this increase and would have led to a 3% decrease in incidence if age structure and population size had remained constant between 2006 and 2016 (eTable 14 and eFigure 21 in the Supplement). ASIRs between 1990 and 2016 show diverging results between men and women globally and in high and high-middle SDI countries, with the ASIR in men decreasing but increasing in women. In middle SDI countries, ASIRs increased for both men and women but remained stable in low-middle and low SDI countries (eFigures 23 and 24 in the Supplement).

#### 2. Colon and Rectum Cancer

In 2016, there were 1.7 million (95% UI, 1.7-1.8 million) incident cases of colon and rectum cancer, and 830 000 (95% UI, 797 000-860 000) deaths (Table). Colon and rectum cancer caused 17.2 million (95% UI, 16.5-17.9 million) DALYs in 2016, of which 97% came from YLLs and 3% from YLDs (eTable 15 and eFigure 4 in the Supplement). The odds of developing colon and rectum cancer globally was higher for men than for women (1 in 26 men, 1 in 41 women; eTable 16 in the Supplement). The highest odds were in the high SDI quintile (1 in 15 men, 1 in 24 women), and the lowest in the low SDI quintile (1 in 112 men, 1 in 116 women). Between 2006 and 2016, incidence increased by 34% (95% UI, 28%-41%), from 1.3 million (95% UI, 1.27-1.30 million) to 1.7 million (95% UI, 1.66-1.79 million) cases (eTable 14 in the Supplement). Most of this increase can be explained by an aging and growing population (19% and 12%, respectively); however, even with the same population size and age structure, colorectal cancer cases would have increased by 2% between 2006 and 2016 due to changing age-specific incidence rates. ASIRs between 1990 and 2016 were similar for men and women for all levels of SDI except for the high-middle SDI quintile, where trends leveled off in women but increased in men (eFigures 25 and 26 in the Supplement). Between 2006 and 2016, for both sexes combined, the ASIR and ASDR decreased in the high SDI quintile. In the high-middle, and middle SDI quintile, the ASDR decreased but the ASIR increased, and for low-middle, and low SDI countries, both ASIR and ASDR increased (eFigure 7 in the Supplement).

#### 3. Breast Cancer

Breast cancer was the third most common incident cancer overall, with an estimated 1.7 million (95% UI, 1.6-1.8 million) incident cases in 2016. The vast majority occurred in women, (1.68 million; 95% UI, 1.61-1.78 million) (Table). Breast cancer was among the top 3 leading causes of cancer in all SDI quintiles except for the high and middle SDI quintiles, where it was the fifth and fourth most common cancer, respectively (Figure 5). It caused 535 000 (95% UI, 506 000-573 000) deaths in women and 10 000 (95% UI, 7000-11 000) deaths in men, making it the fifth leading cause of cancer deaths for both sexes combined in 2016 globally (Figure 6). For women, breast cancer was the leading cause of cancer death in 2016 (Table). Breast cancer caused 15.1 million (95% UI, 14.3-16.2 million) DALYs for both sexes, of which 95% came from YLLs and 5% from YLDs (eTable 15 and eFigure 4 in the Supplement). Globally, 1 in 20 women developed breast cancer over a lifetime (eTable 16 in the Supplement). For women, the odds of developing breast cancer were the highest in high SDI countries (1 in 10), and the lowest in low SDI countries (1 in 50). For women, breast cancer was the most common cancer in 131 countries and the most common cause of cancer deaths in 112 countries (eFigures 18 and 20 in the Supplement). Overall incident cases increased by 29% because of a change in the population age structure (contributing 16%), population growth (contributing 12%), and an increase in age-specific incidence rates (contributing 1%) (eFigure 21 in the Supplement). Between 2006 and 2016, ASIRs decreased or remained stable in high, and high-middle SDI countries, but increased in the other SDI quintiles. ASDR decreased within that timeframe in all SDI quintiles, except for the low SDI quintile, where it increased (eFigure 8 in the Supplement).

#### 4. Nonmelanoma Skin Cancer

In 2016, there were 1.5 million (95% UI, 1.1-2.0 million) incident cases of NMSC, of which 886 000 (95% UI, 574 000-1.3 million) were due to basal cell carcinoma (BCC) and 635 000 (95% UI, 386 000-922 000) due to squamous cell carcinoma (SCC). There were 53 000 (95% UI, 51 000-55 000) deaths due to NMSC (Table) and 1.0 million (95% UI, 981 000-1.1 million) DALYs, of which 97% came from YLLs and 3% from YLDs (eTable 15 and eFigure 4 in the Supplement). Over a lifetime, the odds of developing NMSC were 1 in 31 for men and 1 in 50 for women globally. For SCC in men, it ranged from 1 in 458 in low-middle SDI countries to 1 in 24 in high SDI countries; and for BCC, from 1 in 241 in low-middle SDI countries to 1 in 29 in high SDI countries (eTable 16 in the Supplement). An aging and growing population has led to a 12% (95% UI, 6%-19%) increase in NMSC cancer cases, from 1.4 million (95% UI, 999 000-1.8 million) in 2006-1.5 million (95% UI, 1.1-2.0 million) in 2016. The majority of this increase (20%) can be attributed to a change in the population age structure. Twelve percent can be attributed to population growth. Part of this increase was offset by a decrease in age-specific incidence rates between 2006 and 2016, which would have led to a 20% decrease in overall incidence of NMSC if the age structure and population size had remained stable during this timeframe (eTable 14 and eFigure 21 in the Supplement).

#### 5. Prostate Cancer

In 2016, there were 1.4 million (95% UI, 1.3-1.6 million) incident cases of prostate cancer and 381 000 (95% UI, 321 000-413 000) deaths. Prostate cancer caused 6.1 million (95% UI, 5.0-6.6 million) DALYs globally in 2016, with 91% coming from YLLs and 9% from YLDs (eTable 15 and eFigure 4 in the Supplement). Globally, the odds of developing prostate cancer was 1 in 16 ranging from 1 in 56 for low-middle SDI countries to 1 in 7 in high SDI countries (eTable 16 in the Supplement). In 2016, prostate cancer was the cancer with the highest incidence for men in 92 countries, and the leading cause of cancer deaths for men in 48 countries (eFigures 17 and 19 in the Supplement). The increasing incidence rates, together with an aging and growing population, have led to a 40% increase in prostate cancer cases since 2006: 1.0 million (95% UI, 942 000-1.1 million) in 2006 to 1.4 million (95% UI, 1.3-1.6 million) in 2016. Twenty percent of this increase can be attributed to a change in the population age structure, 12% to a change in the population size, and 7% to a change in the age-specific incidence rates (eTable 14 and eFigure 21 in the Supplement).

#### 6. Stomach Cancer

**Figure 7.**
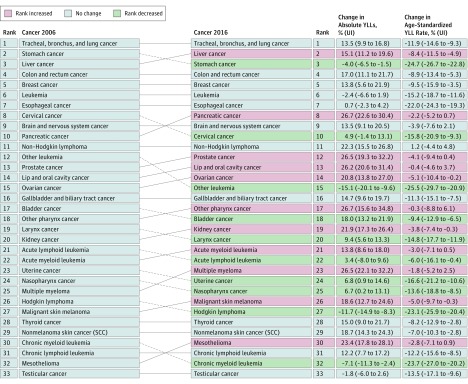
Cancers Ranked by Absolute Years of Life Lost (YLLs) Between 2006 and 2016^a^ SCC indicates squamous cell carcinoma; UI, uncertainty interval. ^a^Excluding “other cancer.”

In 2016, there were 1.2 million (95% UI, 1.1-1.2 million) incident cases of stomach cancer and 834 000 (95% UI, 814 000-855 000) deaths worldwide. Stomach cancer caused 18.3 million (95% UI, 17.9-18.9 million) DALYs in 2016, with 98% coming from YLLs and 2% coming from YLDs (eTable 15 and eFigure 4 in the Supplement). One in 32 men and 1 in 80 women developed stomach cancer over a lifetime. The highest odds for men were in middle SDI countries (1 in 24), and the lowest in low SDI countries (1 in 90). For women, the highest odds were in high-middle SDI countries (1 in 69) and the lowest in low SDI countries (1 in 140) (eTable 16 in the Supplement). Between 2006 and 2016, stomach cancer moved from the second leading cause of crude cancer YLLs to the third place with a 4% decrease (−4% change; 95% UI, −6.5% to −1.5%) in absolute YLLs (Figure 7). Overall, incidence between 2006 and 2016 increased by 15%, of which a change in the population age structure contributed 18%; population growth, 12%; and falling age-specific rates, −15%. (eTable 14 and eFigure 21 in the Supplement). ASIRs have dropped substantially since 1990 globally and for all SDI quintiles (eFigures 31 and 32 in the Supplement).

#### 7. Liver Cancer

In 2016, there were 1.0 million (95% UI, 953 000-1.0 million) incident cases of liver cancer globally and 829 000 (95% UI, 796 000-858 000) deaths. Liver cancer caused 21.1 million (95% UI, 20.3-22.0 million) DALYs in 2016, with 99% coming from YLLs and 1% coming from YLDs (eTable 15 and eFigure 4 in the Supplement). Globally, liver cancer was more common in men, with 1 in 38 men developing liver cancer compared with 1 in 111 women. The highest odds of developing liver cancer were in middle SDI countries (1 in 26 men, 1 in 76 women), whereas the lowest were seen in low-middle SDI countries (1 in 93 men, 1 in 195 women) (eTable 16 in the Supplement). Population aging and population growth were the drivers of the increase from 732 000 (95% UI, 702 000-747 000) cases in 2006 to 1.0 million (95% UI, 953 000-1.0 million) cases in 2016 (eTable 14 and eFigure 21 in the Supplement). Of the 38% increase in cases between 2006 and 2016, 16% was due to population aging, 12% due to population growth, and 9% due to an increase in age-specific incidence rates. Trends in ASIRs for liver cancer differ by SDI quintile. For women, rates decreased in the middle, low-middle, and low SDI quintiles, whereas they increased in the high SDI quintile (eFigure 33 in the Supplement). The same increase in the high SDI quintile can be seen in men (eFigure 34 in the Supplement). Between 2006 and 2016, ASIRs for both sexes increased in the high, high-middle, and middle SDI countries but decreased in the low-middle, and low SDI countries. The ASDRs during that timeframe decreased in all SDI quintiles except for the low SDI quintile, where it increased by 3% (eFigure 11 in the Supplement).

#### 8. Cervical Cancer

In 2016, 511 000 (95% UI, 414 000-542 000) women developed cervical cancer worldwide, and it caused 247 000 (95% UI, 204 000-263 000) deaths (Table). Cervical cancer caused 7.4 million (95% UI, 6.0-7.9 million) DALYs, with 97% coming from YLLs and 3% from YLDs (eTable 15 and eFigure 4 in the Supplement). Globally, 1 in 75 women developed cervical cancer during a lifetime (eTable 16 in the Supplement). The odds were the highest in low SDI countries (1 in 31), and the lowest in high SDI countries (1 in 117). Cervical cancer was the most common cause for cancer incidence and death in low SDI countries (Web Table 4; http://ghdx.healthdata.org/node/350478). In 2016, cervical cancer was the most common incident cancer for women in 51 countries (eFigure 18 in the Supplement) and the most common cause of cancer deaths in 42 countries (eFigure 20 in the Supplement). Between 2006 and 2016, incident cases increased by 9% (95% UI, 2%-17%) globally. Population growth contributed 12%, and population aging, 11%, while falling age-specific incidence rates offset this increase by −15% (eFigure 21 and eTable 14 in the Supplement). Deaths increased by 7% (95% UI, 1%-15%) between 2006 and 2016, and DALYs by 5% (95% UI, −1% to 13%) (Web Table 1; http://ghdx.healthdata.org/node/350478). ASIRs decreased globally, and for all SDI quintiles (eFigure 35 in the Supplement).

#### 9. Leukemia

In 2016, there were 467 000 (95% UI, 423 000-489 000) new cases of leukemia worldwide and 310 000 (95% UI, 286 000-324 000) deaths. In 2016, leukemia caused 10.2 million (95% UI, 9.3-10.8 million) DALYs globally, with 98% coming from YLLs and 2% from YLDs (eTable 15 and eFigure 4 in the Supplement). Globally, 1 in 118 men compared with 1 in 194 women developed leukemia. Between 2006 and 2016, incident cases increased by 26% from 370 000 (95% UI, 344 000-385 000) to 467 000 (95% UI, 423 000-489 000). The main contributors to this increase were population growth with 12%, population aging with 10%, and an increase in age-specific incidence rates with 3% (eFigure 21 and eTable 14 in the Supplement). ASIR trends between 1990 and 2016 for women showed decreasing trends in the low-middle SDI and low SDI quintiles but increasing trends over the last decade in high-middle and middle SDI quintiles (eFigure 36 in the Supplement). For men, rates remained stable between 1990 and 2016 in middle, low-middle, and low SDI countries but increased in high SDI and high-middle SDI countries (eFigure 37 in the Supplement).

#### 10. Non-Hodgkin Lymphoma

In 2016, there were 461 000 (95% UI, 428 000-482 000) incident cases of non-Hodgkin lymphoma and 240 000 (95% UI, 221 000-248 000) deaths. Non-Hodgkin lymphoma caused 6.8 million (95% UI, 6.2-7.1 million) DALYs in 2016, with 98% coming from YLLs and 2% from YLDs (eTable 15 and eFigure 4 in the Supplement). Globally, 1 in 110 men and 1 in 161 women developed non-Hodgkin lymphoma over a lifetime. The highest odds were in high SDI countries (1 in 48 for men, 1 in 70 for women), and the lowest were in low-middle SDI countries (1 in 274 for men, 1 in 401 for women) (eTable 16 in the Supplement). Globally, incident cases between 2006 and 2016 increased by 45% (95% UI, 38%-48%), of which 17% was due to increasing age-specific incidence rates, 15% to changing population age structure, and 12% to population growth (eTable 14 and eFigure 21 in the Supplement). In eFigures 38 and 39 in the Supplement, the slight increase in ASIRs between 1990 and 2016 is shown, with very similar trends for men and women and all SDI quintiles except for the high SDI quintile, where ASIRs increase more rapidly.

### Trends in Incidence for Less Common Cancers

Globally, incident cases for all cancers increased significantly between 2006 and 2016 for both sexes combined. Of the cancers other than the top 10, the top 3 cancers with the largest increase in incident cases were thyroid cancer (50% increase; 95% UI, 43%-59%; uterine cancer (40% increase; 95% UI, 34%-50%); and melanoma (39% increase; 95% UI, 33%-43%) (Web Table 1; http://ghdx.healthdata.org/node/350478). For thyroid cancer, of the 50% increase, 25% can be explained by rising age-specific incidence rates, 12% by an increase in population size, and 12% by a change in age structure. For uterine cancer, of the 40% increase, 18% was due to a change in the population age structure, 12% to an increase in population size, and 10% to a rise in age-specific incidence rates. For melanoma, of the 39% increase, 15% was due to a change in the population age-structure, 12% to population growth, and 11% to a change in age-specific incidence rates (eTable 14 in the Supplement).

## Discussion

We updated our previous reports and analyzed cancer registry, vital registration, and verbal autopsy data to estimate the burden of cancer for 195 countries and territories from 1990 to 2016.^7,15^ In this article, we focus on the changes over the last decade (2006-2016). All results presented can also be found online at https://vizhub.healthdata.org/gbd-compare/. Changes compared with our previous reports include the addition of NMSC, additional data sources (eTable 3 in the Supplement), and improvements in the estimation of the MIR.

We found that the global cancer burden between 2006 and 2016 increased in terms of incident cases, deaths, and DALYs, with vast heterogeneity by cancer type, location, and sex. A large proportion of the increase in cancer incidence can be explained by improving life expectancy and population growth—a development that can at least partially be attributed to a reduced burden from other common diseases.^8,14^ However, the contribution of population aging vs population growth to changes in incident cases differs substantially based on socioeconomic development. This leads to very different compositions of cancer types contributing to total incident cases in a population depending on the age structure.

Despite the rapidly increasing cancer burden in lower SDI countries, the odds of developing cancer and age-standardized rates are still higher in countries of higher SDI. Notable exceptions are cancers with infectious etiologies like cervical, liver, and stomach cancer.

### Infectious Causes for Cancer

Cervical cancer is the most striking example of inequity for cancers of infectious etiologies, where women in low SDI countries are almost 4 times more likely to develop cervical cancer compared with women in high SDI countries, and where cervical cancer is the most common cause of cancer incidence and deaths. A positive development is that cervical cancer ASIRs have fallen in all SDI quintiles, likely due to improvements in primary and secondary prevention as well as improvements in SDI.^16^ However, with almost a quarter million women still dying annually from a preventable cancer, much work is left to be done.^17^

Stomach and liver cancer are another example of the large heterogeneity in the burden of cancers with infectious etiologies. As is the case with cervical cancer, a positive development is that stomach cancer rates have fallen in all SDI quintiles over the last decade. Liver cancer is the leading cause of cancer deaths in many lower SDI countries but only the seventh leading cause of cancer deaths in high SDI countries. A concerning trend, however, is that rates in higher SDI countries are increasing, which has been attributed to increasing risk factors like nonalcoholic steatohepatitis, alcohol abuse, and hepatitis B and C in certain populations.^18,19,20^

### Potential for Cancer Prevention

The mostly positive development for cancers with an infectious etiology can at least partially be attributed to the large prevention potential. Common cancers without infectious etiologies but also with a large prevention potential include TBL cancer through tobacco control; colorectal cancer through screening, dietary interventions, and the promotion of physical activity; and skin cancer through prevention of excessive UV exposure. For TBL cancer, ASIRs in higher SDI countries have decreased over the last decade, which can be attributed to tobacco control.^21^ However, even though TBL cancer rates in lower SDI countries are below the rates in higher SDI countries, ASIRs in lower SDI countries increased between 2006 and 2016. This highlights the importance of focusing tobacco control efforts on lower SDI countries, to avoid these countries’ having to experience the same tragedy of unnecessary tobacco-related deaths that many high SDI countries have had to face. In addition to tobacco control, indoor and outdoor air pollution have to be considered as important risk factors for lung cancer in certain locations.^11^ When considering the value of prevention strategies, the benefit in reducing diseases other than cancer can be considerable, as is the case for example with tobacco control.

Unfortunately, cancer prevention efforts are less effective for common cancers like breast and prostate cancer, as well as hematological malignant conditions like leukemia and non-Hodgkin lymphoma, and pediatric cancers. Also, cancer prevention always has to be seen in conjunction with expanding access to early detection and treatment to not neglect cohorts of current or future patients for whom cancer prevention efforts come too late.

### Need for Access to Cancer Care

Since even in the best-case scenario only a fraction of cancers are preventable under current conditions, providing universal access to health care is crucial for cancer control.^11^ Especially the finding of increasing ASIRs for some cancers at the same time as ASDRs are decreasing in higher SDI countries, which points to the benefits of early cancer detection and effective treatment but also highlights the potential for overdiagnosis.^22^

Globally, most of the cancer burden still comes from YLLs rather than YLDs, reflecting a higher burden of deaths than disability. However, as cancer treatment improves and the population ages, survivorship care becomes an essential part of the cancer continuum. Over the last decade, resource-stratified guidelines that encompass this continuum from diagnosis through survivorship to end-of-life care have been developed.^23^ Together with the GBD estimates and other data on the local burden of cancer, these guidelines provide countries helpful tools when designing health policies and cancer control plans.

### Limitations

For effective cancer control and resource allocation, information on the local cancer burden but also on the burden of other diseases is crucial. The GBD estimates fill a gap where actual data on disease burden are sparse or unavailable. However, in these cases, estimates have wide uncertainty, and it remains crucial to improve data collection through the expansion and creation of vital registration systems, cancer registries, health surveys, and other data systems. Differences in data collection practices and coding systems, as well as quality of data sources, remain major challenges, as do underreporting of cancers requiring advanced diagnostics in low-resource settings (eg, brain cancer, leukemias, and others). Cancers that are common in the pediatric population but rare in adults are aggregated to an “other neoplasm” group, encompassing about 30% of the pediatric cancer burden and making these estimates less valuable for cancer control.

## Conclusions

With the annual updates of the GBD cancer estimates, our goal is to provide relevant and current information on the global, regional, and national burden of cancer. The GBD 2016 study offers new insights into the magnitude of cancer disparities. With population aging and the epidemiological transition, cancer incidence will increase in the future, further widening the cancer divide if current trends continue. The data showing the disparities and knowledge on the root causes exist, as do the tools to reduce them. However, strategic investments in cancer control and implementation of effective programs to ensure universal access to cancer care are required to achieve the Sustainable Development Goals as well as targets set in the WHO Global Action Plan on NCDs.

## Notes

Supplement. eAppendix. eTables 1 through 16. eFigures 1 through 72.
