# Supplementary material for: Global, Regional, and National Cancer Incidence, Mortality, Years of Life Lost, Years Lived With Disability, and Disability-Adjusted Life-Years for 29 Cancer Groups, 1990 to 2016: A Systematic Analysis for the Global Burden of Disease Study
Source: JAMA Oncol. 2018 Jun 2;4(11). doi: 10.1001/jamaoncol.2018.2706 (PMC6248091; doi:10.1001/jamaoncol.2018.2706)
Supplement: Supplement. — eAppendix. eTables 1 through 16. eFigures 1 through 72. [file jamaoncol-4-1553-s001.pdf]

Supplementary Online Content

Global Burden of Disease Cancer Collaboration. Global, Regional, and National Cancer Incidence, Mortality, Years of Life Lost, Years Lived With Disability, and Disability-Adjusted Life-Years for 29 Cancer Groups, 1990 to 2016
A Systematic Analysis for the Global Burden of Disease Study. *JAMA Oncology*. Published online June 2, 2018. doi:10.1001/jamaoncol.2018.2706

eAppendix.

eTables 1 through 16.

eFigures 1 through 72.

This supplementary material has been provided by the authors to give readers additional information about their work.

© 2018 Fitzmaurice C et al. *JAMA Oncology*.

Supplementary Online Content

Global Burden of Disease Cancer Collaboration. Global, regional, and national cancer incidence, mortality, years of life lost, years lived with disability, and disability-adjusted life years for 29 cancer groups, 1990 to 2016: a systematic analysis for the Global Burden of Disease Study 2016.

eAppendix

Definition of indicator 5

Data sources 5

Cancer incidence data sources 5

Mortality/incidence ratio data sources 5

Cancer mortality data sources 5

Bias of categories of input data 5

Data analysis 6

Cancer registry data formatting 6

Cause of death database formatting 9

CODEm models 9

Liver cancer etiology split models 9

CodCorrect 10

Incidence estimation 10

Prevalence and YLD estimation 10

Probability of cancer 13

References 14

© 2018 Fitzmaurice C et al. *JAMA Oncology*.

eTable 1: GATHER Guidelines checklist 17
eTable 2: Sources for cancer incidence and mortality-to-incidence ratio data by country, year, and registry 19
eTable 3: Number of site-years for cancer mortality data by type 46
eTable 4: List of International Classification of Diseases (ICD) codes mapped to the Global Burden of Disease cause list for cancer incidence data 48
eTable 5: List of International Classification of Diseases (ICD) codes mapped to the Global Burden of Disease cause list for cancer mortality data 50
eTable 6: Undefined cancer code categories (ICD-10) and respective target codes for cancer registry incidence data 53
eTable 7: Sociodemographic Index groupings by geography, based on 2016 values 53
eTable 8: Covariates selected for CODEm for each GBD cancer group and expected direction of covariate 59
eTable 9: Comparison of GBD 2015 and GBD 2016 covariates used and level of covariates 83
eTable 10: Results for CODEm model testing 108
eTable 11: Percent change before and after CodCorrect by cancer for all ages, both sexes combined, 2016 118
eTable 12: Duration of four prevalence phases by cancer 120
eTable 13: Disability weights 123
eTable 14: Decomposition of trends in incidence globally, and by SDI quintile, both sexes, 2006 to 2016 124
eTable 15: Contribution of YLDs and YLLs to DALYs by cancer, global, both sexes, 2016 150
eTable 16: Probability of developing cancer within selected age intervals, global, and by SDI quintile, by sex, 2006-2016 in % (odds) 151

eFigure 1: Flowchart GBD cancer mortality, YLL estimation 178
eFigure 2: Flowchart GBD cancer incidence, prevalence, YLD estimation 179
eFigure 3: Sociodemographic Index quintiles, 2016 180
eFigure 4: Contribution of YLDs and YLLs to DALYs by cancer, global, both sexes, 2016 181
eFigure 5: Average annual percent change in age-standardized death and incidence rates between 2006 and 2016 for both sexes, by SDI quintile, all neoplasms 182
eFigure 6: Average annual percent change in age-standardized death and incidence rates between 2006 and 2016 for both sexes, by SDI quintile, tracheal, bronchus, and lung cancer 183
eFigure 7: Average annual percent change in age-standardized death and incidence rates between 2006 and 2016 for both sexes, by SDI quintile, colon and rectum cancer 184
eFigure 8: Average annual percent change in age-standardized death and incidence rates between 2006 and 2016 for both sexes, by SDI quintile, breast cancer 185
eFigure 9: Average annual percent change in age-standardized death and incidence rates between 2006 and 2016, by SDI quintile, prostate cancer 186
eFigure 10: Average annual percent change in age-standardized death and incidence rates between 2006 and 2016 for both sexes, by SDI quintile, stomach cancer 187
eFigure 11: Average annual percent change in age-standardized death and incidence rates between 2006 and 2016 for both sexes, by SDI quintile, liver cancer 188

© 2018 Fitzmaurice C et al. *JAMA Oncology*.

eFigure 12: Average annual percent change in age-standardized death and incidence rates between 2006 and 2016, by SDI quintile, cervical cancer 189
eFigure 13: Average annual percent change in age-standardized death and incidence rates between 2006 and 2016 for both sexes, by SDI quintile, leukemia........ 190
eFigure 14: Average annual percent change in age-standardized death and incidence rates between 2006 and 2016 for both sexes, by SDI quintile, non-Hodgkin lymphoma 191
eFigure 15: Cancer ranking by total incidence based on global level for developing and developed regions and all countries, both sexes, 2016........ 201
eFigure 16: Cancer ranking by total mortality based on global level for developing and developed regions and all countries, both sexes, 2016 210
eFigure 17: Top-ranked cancers by absolute incident cases for all ages in males, 2016 211
eFigure 18: Top-ranked cancers by absolute incident cases for all ages in females, 2016 212
eFigure 19: Top-ranked cancers by absolute deaths for all ages in males, 2016 213
eFigure 20: Top-ranked cancers by absolute deaths for all ages in females, 2016 214
eFigure 21: Global Decomposition of Changes in Cancer Incident Cases due to Population Growth, Population Aging, and Changes in Age-specific Incidence Rates, Both Sexes, 2006 to 2016. 215
eFigure 22: Average annual change in age-standardized mortality rate between 2006 and 2016 for both sexes by SDI and cancer (........ **Error! Bookmark not defined.**
eFigure 23: Trends in Age-Standardized Incidence Rates for Tracheal, Bronchus, and Lung Cancer, 1990-2016, Female........ 216
eFigure 24: Trends in Age-Standardized Incidence Rates for Tracheal, Bronchus, and Lung Cancer, 1990-2016, Male 216
eFigure 25: Trends in Age-Standardized Incidence Rates for Colon and Rectum Cancer, 1990-2016, Female........ 217
eFigure 26: Trends in Age-Standardized Incidence Rates for Colon and Rectum Cancer, 1990-2016, Male 217
eFigure 27: Trends in Age-Standardized Incidence Rates for Breast Cancer, 1990-2016, Female........ 218
eFigure 28: Trends in Age-Standardized Incidence Rates for NMSC, 1990-2016, Female 218
eFigure 29: Trends in Age-Standardized Incidence Rates for NMSC, 1990-2016, Male........ 219
eFigure 30: Trends in Age-Standardized Incidence Rates for Prostate Cancer, 1990-2016, Male 219
eFigure 31: Trends in Age-Standardized Incidence Rates for Stomach Cancer, 1990-2016, Female 220
eFigure 32: Trends in Age-Standardized Incidence Rates for Stomach Cancer, 1990-2016, Male........ 220
eFigure 33: Trends in Age-Standardized Incidence Rates for Liver Cancer, 1990-2016, Female 221
eFigure 34: Trends in Age-Standardized Incidence Rates for Liver Cancer, 1990-2016, Male 221
eFigure 35: Trends in Age-Standardized Incidence Rates for Cervical Cancer, 1990-2016, Female 222
eFigure 36: Trends in Age-Standardized Incidence Rates for Leukemia, 1990-2016, Female........ 222
eFigure 37: Trends in Age-Standardized Incidence Rates for Leukemia, 1990-2016, Male 223
eFigure 38: Trends in Age-Standardized Incidence Rates for Non-Hodgkin lymphoma, 1990-2016, Female 223
eFigure 39: Trends in Age-Standardized Incidence Rates for Non-Hodgkin lymphoma, 1990-2016, Male 224
eFigure 40: Trends in Age-Standardized Incidence Rates for Esophageal Cancer, 1990-2016, Female... 225
eFigure 41: Trends in Age-Standardized Incidence Rates for Esophageal Cancer, 1990-2016, Male 225
eFigure 42: Trends in Age-Standardized Incidence Rates for Bladder Cancer, 1990-2016, Female........ 226

© 2018 Fitzmaurice C et al. *JAMA Oncology*.

eFigure 43: Trends in Age-Standardized Incidence Rates for Bladder Cancer, 1990-2016, Male 226
eFigure 44: Trends in Age-Standardized Incidence Rates for Pancreatic Cancer, 1990-2016, Female 227
eFigure 45: Trends in Age-Standardized Incidence Rates for Pancreatic Cancer, 1990-2016, Male........ 227
eFigure 46: Trends in Age-Standardized Incidence Rates for Uterine Cancer, 1990-2016, Female......... 228
eFigure 47: Trends in Age-Standardized Incidence Rates for Lip and oral cavity Cancer, 1990-2016, Female........ 228
eFigure 48: Trends in Age-Standardized Incidence Rates for Lip and oral cavity Cancer, 1990-2016, Male 229
eFigure 49: Trends in Age-Standardized Incidence Rates for Kidney Cancer, 1990-2016, Female 229
eFigure 50: Trends in Age-Standardized Incidence Rates for Kidney Cancer, 1990-2016, Male.............. 230
eFigure 51: Trends in Age-Standardized Incidence Rates for Brain and nervous system cancer, 1990-2016, Female........ 230
eFigure 52: Trends in Age-Standardized Incidence Rates for Brain and nervous system cancer, 1990-2016, Male 231
eFigure 53: Trends in Age-Standardized Incidence Rates for Melanoma, 1990-2016, Female................ 231
eFigure 54: Trends in Age-Standardized Incidence Rates for Melanoma, 1990-2016, Male 232
eFigure 55: Trends in Age-Standardized Incidence Rates for Ovarian Cancer, 1990-2016, Female 232
eFigure 56: Trends in Age-Standardized Incidence Rates for Thyroid Cancer, 1990-2016, Female......... 233
eFigure 57: Trends in Age-Standardized Incidence Rates for Thyroid Cancer, 1990-2016, Male 233
eFigure 58: Trends in Age-Standardized Incidence Rates for Larynx Cancer, 1990-2016, Female........... 234
eFigure 59: Trends in Age-Standardized Incidence Rates for Larynx Cancer, 1990-2016, Male 234
eFigure 60: Trends in Age-Standardized Incidence Rates for Gallbladder Cancer, 1990-2016, Female .. 235
eFigure 61: Trends in Age-Standardized Incidence Rates for Gallbladder Cancer, 1990-2016, Male...... 235
eFigure 62: Trends in Age-Standardized Incidence Rates for Other Pharynx Cancer, 1990-2016, Female 236
eFigure 63: Trends in Age-Standardized Incidence Rates for Other Pharynx Cancer, 1990-2016, Male . 236
eFigure 64: Trends in Age-Standardized Incidence Rates for Multiple Myeloma, 1990-2016, Female ... 237
eFigure 65: Trends in Age-Standardized Incidence Rates for Multiple Myeloma, 1990-2016, Male....... 237
eFigure 66: Trends in Age-Standardized Incidence Rates for Nasopharynx Cancer, 1990-2016, Female 238
eFigure 67: Trends in Age-Standardized Incidence Rates for Nasopharynx Cancer, 1990-2016, Male ... 238
eFigure 68: Trends in Age-Standardized Incidence Rates for Hodgkin Lymphoma, 1990-2016, Female. 239
eFigure 69: Trends in Age-Standardized Incidence Rates for Hodgkin Lymphoma, 1990-2016, Male 239
eFigure 70: Trends in Age-Standardized Incidence Rates for Testicular Cancer, 1990-2016, Male......... 240
eFigure 71: Trends in Age-Standardized Incidence Rates for Mesothelioma, 1990-2016, Female.......... 240
eFigure 72: Trends in Age-Standardized Incidence Rates for Mesothelioma, 1990-2016, Male 241

© 2018 Fitzmaurice C et al. *JAMA Oncology*.

Definition of indicator

The GBD cause list is organized in a hierarchy. Levels 1 and 2 represent general groupings. The broad group "neoplasms." which includes all cancer causes, is at Level 2 under the Level 1 group Non-communicable diseases. Level 3 includes 29 cancer groups, and Level 4 includes 37 groups since in Level 4, leukemia, liver cancer, and non-melanoma skin cancer are further subdivided. In this publication, estimates for the GBD cancer groups, for both sexes, for the time from 1980 to 2016, and for the 5-year GBD age groups (0-5; 5-9; etc. until 95+) are presented for 195 countries or territories. All ICD9 codes pertaining to cancer (140-209) and ICD10 codes (C00-C96) except for Kaposi sarcoma (ICD10: C46) are included in these estimates.

eTable 4 and eTable 5 list all ICD codes and their respective GBD cause. Countries and territories reported can be found in eTable 7.

Data sources

Cancer incidence data sources

Cancer incidence was sought from individual cancer registries or aggregated databases of cancer registry data like "Cancer Incidence In Five Continents" (CI5),"[1–10] EUREG,[11] or NORDCAN.[12] Data were excluded if they were not representative of the coverage population (e.g., hospital-based registries), if they did not cover all malignant neoplasms as defined in ICD9 (140-208) or ICD10 (C00-C96) (e.g., specialty cancer registry), if they did not include data for both sexes and all age groups, if the data were limited to years prior to 1980, or if the source did not provide details on the population covered. Preference was given to registries with national coverage over those with only local coverage, except those from countries where the GBD study provides subnational estimates. A list of the cancer registries included in our analysis and the years covered can be found in eTable 2. Additional metadata for each source are available in the online GBD citation tool, http://ghdx.healthdata.org/gbd-2016/data-input-sources.

Mortality/incidence ratio data sources

Most cancer registries only report cancer incidence. However, if a cancer registry also reported cancer mortality, mortality data were also extracted from the source to be used in the mortality to incidence estimation. eTable 2 lists the registries used for the estimation of mortality-to-incidence ratios.

Cancer mortality data sources

A detailed description of the data sources and processing steps for the cause of death database can be found in the appendix to the GBD 2016 paper "Global, regional, and national age-sex specific mortality for 264 causes of death, 1980–2016: a systematic analysis for the Global Burden of Disease Study 2016."[13]

Bias of categories of input data

Bias of the input data included for the COD database is described elsewhere.[13] Cancer registry data can be biased in multiple ways. A high proportion of ill-defined cancer cases in the registry data requires redistribution of these cases to other cancers, which introduces a potential for bias. Changes between coding systems can lead to artificial differences in disease estimates; however, we adjust for this bias by mapping the different coding systems to the GBD causes. Underreporting of cancers that require advanced diagnostic techniques (e.g., leukemia, brain, pancreatic, and liver cancer) can be an issue in cancer registries from low-income countries. On the other hand, misclassification of metastatic sites as primary cancer can lead to overestimation of cancer sites that are common sites for metastases like brain or liver. Since many cancer registries are located in urban areas, the representativeness of the

© 2018 Fitzmaurice C et al. *JAMA Oncology*.

registry for the general population can also be problematic. The accuracy of mortality data reported in cancer registries usually depends on the quality of the vital registration system. If the vital registration system is incomplete or of poor quality, the mortality-to-incidence ratio can be biased to lower ratios.

Data analysis

Flowcharts describing the conceptual overview of the data processing are available in eFigure 1 and eFigure 2.

Cancer registry data formatting

Cancer registry data went through multiple processing steps before integration with the COD database. First, the original data were transformed into standardized files, which included standardization of format, categorization, and registry names (#1 in eFigure 1).

Second, some cancer registries report individual codes as well as aggregated totals (e.g., C18, C19, and C20 are reported individually but the aggregated group of C18–C20 (colorectal cancer) is also reported in the registry data). The data processing step "subtotal recalculation" (#2 in flowchart) verifies these totals and subtracts the values of any individual codes from the aggregates.

In the third step (#3 in the flowchart), cancer registry incidence data and cancer registry mortality data are mapped to GBD causes. A different map is used for incidence and for mortality data because of the assumption that there are no deaths for certain cancers. One example is basal cell carcinoma of the skin. In the cancer registry incidence data, basal cell carcinoma is mapped to non-melanoma skin cancer (basal cell carcinoma). However, if basal cell skin cancer is recorded in the cancer registry mortality data, the deaths are instead mapped to non-melanoma skin cancer (squamous cell carcinoma) under the assumption that they were indeed misclassified squamous cell skin cancers. Other examples are benign or in situ neoplasms. Benign or in situ neoplasms found in the cancer registry incidence dataset were simply dropped from that dataset. The same neoplasms reported in a cancer registry mortality dataset were mapped to the respective invasive cancer (e.g., melanoma in situ in the cancer registry incidence dataset was dropped from the dataset; melanoma in situ in the cancer registry mortality dataset was mapped to melanoma). Mapping for incidence and mortality data can be found in eTable 4 and eTable 5.

In the fourth data processing step (#4 in the flowchart), cancer registry data were standardized to the GBD age groups. Age-specific incidence rates were generated using CI5, SEER, and NORDCAN data, while age-specific mortality rates were generated from the CoD data.[13] Age-specific weights were then generated by applying the age-specific rates to a given registry population that required age-splitting to produce the expected number of cases/deaths for that registry by age. The expected number of cases/deaths for each sex, age, and cancer were then normalized to 1, creating final, age-specific proportions. These proportions were then applied to the total number of cases/deaths by sex and cancer to get the age-specific number of cases/deaths.

In the rare case that the cancer registry only contained data for both sexes combined, the age-specific cases/deaths were split and re-assigned to separate sexes using the same weights that are used for the age-splitting process. Starting from the expected number of deaths, proportions were generated by sex for each age (e.g., if for ages 15-19 years old there are 6 expected deaths for males and 4 expected deaths for females, then 60% of the combined-sex deaths for ages 15-19 years would be assigned to males and the remaining 40% would be assigned to females).

In the fifth step (#5 in the flowchart), data for cause entries that are aggregates of GBD causes were redistributed. Examples of these aggregated causes include some registries reporting ICD10 codes C00-

© 2018 Fitzmaurice C et al. *JAMA Oncology*.

C14 together as, “lip, oral cavity, and pharyngeal cancer.” These groups were broken down into subcauses that could be mapped to single GBD causes. In this example, those include lip and oral cavity cancer (C00-C08), nasopharyngeal cancer (C11), cancer of other parts of the pharynx (C09-C10, C12-C13), and “Malignant neoplasm of other and ill-defined sites in the lip, oral cavity, and pharynx” (C14). To redistribute the data, weights were created using the same method employed in age-sex splitting (see step four above). For the undefined code (C14 in the example) an “average all cancer” weight was used, which was generated by adding all cases rom SEER/NORDCAN/CI5 and dividing those by the combined population. Then, proportions were generated by subcause for each aggregate cause as in the sex splitting example above (see step four). The total number of cases from the aggregated group (C00-C14) was recalculated for each subgroup and the undefined code (C14). C14 was then redistributed as a “garbage code” in step six. Distinct proportions were used for C46 (Kaposi sarcoma). C46 entries were redistributed as “other cancer” and HIV.

In the sixth step (#6 in the flowchart), unspecified codes (“garbage code”) were redistributed. Redistribution of cancer registry incidence and mortality data mirrored the process of the redistribution used in the cause of death database and has not changed compared to GBD 2013.[13]

In the seventh step (#7 in the flowchart), duplicate or redundant sources were removed from the processed cancer registry dataset. Duplicate sources were present if, for example, the cancer registry was part of the CI5 database but we also had data from the registry directly. Redundancies occurred and were removed as described in “Inclusion and Exclusion Criteria,” where more detailed data were available, or when national registry data could replace regionally representative data. From here, two parallel selection processes were run to generate input data for the MI models and to generate incidence for final mortality estimation. Higher priority was given to registry data from the most standardized source when creating the final incidence input, whereas for the MI model input, only sources that reported incidence and mortality were used. This is different from GBD 2015, where mortality and incidence could come from different sources as long as they covered the same population.

In the eighth step (#8 in the flowchart), the processed incidence and mortality data from cancer registries were matched by cancer, age, sex, year, and location to generate MI ratios. These MI ratios were used as input for a three-step modeling approach using the general GBD ST-GPR approach with SDI as a covariate in the linear step mixed effects model using a logit link function. Predictions were made without the random effects. The ST-GPR model has three main hyper-parameters that control for smoothing across time, age, and geography. The time adjustment parameter (λ) was set to 2, which aims to borrow strength from neighboring time points (i.e., the exposure in this year is highly correlated with exposure in the previous year but less so further back in time). The age adjustment parameter ω was set to 0.5, which borrows strength from data in neighboring age groups. The space adjustment parameter ξ was set to 0.95 in locations with data and to 0.5 in locations without data (the higher ξ was applied when at least one age-sex group in the country of estimation had at least five unique data points. The lower ξ was applied when estimating data-scarce countries). Zeta aims to borrow strength across the hierarchy of geographical locations.[13] For the amplitude parameter in the Gaussian process regression we used 2 and for the scale we used a value of 15.

We have modified the approach to estimate MI ratios compared to GBD 2015. Since for GBD 2015 MI ratio predictions for some cancers yielded similar predictions for low-SDI countries without data as for high-SDI countries, we refined the estimation process. Inclusion criteria for the MI ratio input data were changed to only include mortality and incidence data if they were reported by the same source. We excluded MI ratios reported in the CI5[4,4–10] since mortality data used for the calculation of these MI ratios by definition has to be independent from the cancer registry. We also revised the outliering

© 2018 Fitzmaurice C et al. *JAMA Oncology*.

process and excluded data based on the SDI quintile categorization rather than on development status. For each cancer, MI ratios from locations in SDI quintiles 1-4 (low to high-middle SDI) were dropped if they were below the median of MI ratios from locations in SDI quintile 5 (high SDI). We also dropped MI ratios from locations in SDI quintiles 1-4 if the MI ratios were above the third quartile + 1.5 * IQR (inter-quartile range). We dropped all MIR that were based on less than 25 cases to avoid noise due to small numbers except for mesothelioma and acute lymphoid leukemia, where we dropped MIR that were based on less than 10 cases because of lower data availability for these two cancers. We also aggregated incidence and mortality to the youngest 5-year age bin where we had at least 50 data points to avoid MIR predictions in young age groups that were based on few data points. The MIR in the age-bin that was used to aggregate MIR was used to backfill the MIR for younger age groups.
Since MI ratios can be above 1, especially in older age groups and cancers with low cure rates, we used the 95th percentile of the cleaned dataset that only included MIR that were based on 50 or more cases to cap the MIR input data. This "upper cap" was used to allow MIR over 1 but to constrain the MIR to a maximum level. To run the logit model, the input data were divided by the upper caps and model predictions after ST-GPR was rescaled by multiplying them by the upper caps.
Upper caps used for GBD 2016 were the following:

**Age group**	**Maximum MIR**
0-4	0.57
5-9	0.69
10-14	0.81
15-19	0.84
20-24	0.72
25-29	0.62
30-34	0.69
35-39	0.78
40-44	0.86
45-49	0.89
50-54	0.92
55-59	0.95
60-64	0.99
65-69	1.04
70-74	1.10
75-79	1.17
80+	1.32

To constrain the model at the lower end, we used the 5th percentile of the cancer-specific cleaned MIR input data to replace all model predictions with this lower cap.
Final MI ratios were matched with the cancer registry incidence dataset in the ninth step (#9 in the flowchart) to generate mortality estimates (Incidence * Mortality/Incidence = Mortality) (#10 in the flowchart). The final mortality estimates were then uploaded into the COD database (#11 in the flowchart). Cancer-specific mortality modeling then followed the general CODEm process.

© 2018 Fitzmaurice C et al. *JAMA Oncology*.

Cause of death database formatting

Formatting of data sources for the cause of death database has been described in detail elsewhere (#11 in the flowchart).[13]

CODEm models

Mortality estimates for each cancer were generated using CODEm (#12 in the flowchart). Methods describing the CODEm approach have been described elsewhere.[13,14] In brief, the CODEm modeling approach is based on the principles that all types of available data should be used even if data quality varies; that individual models but also ensemble models should be tested for their predictive validity; and that the best model or sets of models should be chosen based on the out of sample predictive validity. Models were run separately for countries with extensive and complete vital registration data and countries with less VR data to prevent an inflation in the uncertainty around the estimates in "data-rich" countries. Covariates were selected based on a possible predictive relationship between the covariate and the specific cancer mortality. Level 1 covariates have a proven strong relationship with the outcome such as etiological or biological roles. Level 2 covariates have a strong relationship but not a direct biological link. Covariates that are more distal in the causal chain or are mediated through Level 1 or 2 covariates are categorized as Level 3.[14] Differences in covariate selection between GBD 2015 and GBD 2016 by cause and direction of the covariate can be found in eTable 9.

Liver cancer etiology split models

To find the proportion of liver cancer cases due to the four etiology groups included in GBD (1. Liver cancer due to hepatitis B, 2. Liver cancer due to hepatitis C, 3. Liver cancer due to alcohol, 4. Liver cancer due to other causes), a systematic literature search was performed on 10/24/2016 in PubMed using the search string *("liver neoplasms"[All Fields] OR "HCC"[All Fields] OR "liver cancer"[All Fields] OR "Carcinoma, Hepatocellular"[Mesh]) AND (("hepatitis B"[All Fields] OR "Hepatitis B"[Mesh] OR "Hepatitis B virus"[Mesh] OR "Hepatitis B Antibodies"[Mesh] OR "Hepatitis B Antigens"[Mesh]) OR ("hepatitis C"[All Fields] OR "Hepatitis C"[Mesh] OR "hepatitis C antibodies"[MESH] OR "Hepatitis C Antigens"[Mesh] OR "Hepacivirus"[Mesh]) OR ("alcohol"[All Fields] OR "Alcohol Drinking"[Mesh] OR "Alcohol-Related Disorders"[Mesh] OR "Alcoholism"[Mesh] OR "Alcohol-Induced Disorders"[Mesh])) AND ("2015"[PDAT] : "2016"[PDAT]) NOT (animals[MeSH] NOT humans[MeSH])*. Studies were included if the study population was representative of the liver cancer population for the respective location. For each study the proportions of liver cancer due to the three specific risk factors were calculated. Remaining risk factors were included under a combined "other" group. Cryptogenic cases were only included if other etiologies like viral hepatitis or alcoholic cirrhosis had been excluded. If multiple risk factors were reported for an individual patient these were apportioned proportionally to the individual risk factors. The proportion data found through the systematic literature review were used as input for four separate DisMod-MR 2.1 models to determine the proportion of liver cancers due to the four subgroups for all locations, both sexes, and all age groups (step #16 in the flowchart). A study covariate was used for publications that only assessed liver cancer in a cirrhotic population. The reference, or "gold standard," that was used for crosswalking was the compilation of all studies that assessed the etiology of liver cancer in a general population. A study covariate was also used for studies that only assessed hepatocellular carcinoma (HCC) as opposed to all primary liver cancers. The reference or "gold standard" that was used for crosswalking was the compilation of all studies that assessed the etiology of liver cancer for the population with all primary liver cancers.

For liver cancer due to hepatitis C and hepatitis B, a prior value of 0 was set between age 0 and 0.01. For

© 2018 Fitzmaurice C et al. *JAMA Oncology*.

liver cancer due to alcohol a prior value of 0 was set for ages 0 to 5 years. For liver cancer due to hepatitis C, hepatitis C (IgG) seroprevalence was used as a covariate as well as a covariate for alcohol (liters per capita) and hepatitis B prevalence (HBsAg seroprevalence), forcing a negative relationship between the alcohol and hepatitis B covariate and the outcome of liver cancer due to hepatitis C proportion. For liver cancer due to hepatitis B, seroprevalence of HBsAg was used as a covariate as well as a covariate for alcohol and hepatitis C IgG seroprevalence, forcing a negative relationship between the alcohol and hepatitis C covariate and the outcome of liver cancer due to hepatitis B proportion. For liver cancer due to alcohol, alcohol (liters per capita) was used as a covariate as well as a covariate for proportion of alcohol abstainers, hepatitis B and hepatitis C seroprevalence, forcing a negative relationship between the proportion of alcohol abstainers, hepatitis B and hepatitis C covariates and the outcome of liver cancer due to alcohol proportion. All covariates used were modeled independently. To ensure consistency between cirrhosis and liver cancer estimates and to take advantage of the data for the respective other related cause (e.g., liver cancer due to hepatitis C and the related cause cirrhosis due to hepatitis C), we generated covariates from the liver cancer proportion models that we used in the cirrhosis etiology proportion models. We then created covariates from the cirrhosis etiology proportion models and used those in the liver cancer etiology models.

Since the proportion models are run independently of each other, the final proportion models were scaled to sum to 100% within each age, sex, year, and location, by dividing each proportion by the sum of the four (step # 17). For the liver cancer subtype mortality estimates, we multiplied the parent cause "liver cancer" by the corresponding scaled proportions (step # 18). Single cause estimates were adjusted to fit into the separately modeled all-cause mortality in the process CodCorrect.

CodCorrect

CODEm models estimate the individual cause-level mortality without taking into account the all-cause mortality (#13 in the flowchart). To ensure that all single causes add up to the all-cause mortality and that all child-causes add up to the parent cause, an algorithm called "CodCorrect" is used (#14 and #15 in the flowchart). Details regarding the algorithm can be found elsewhere.[13]

Incidence estimation

GBD cancer incidence estimates were generated by dividing final mortality estimates (after CodCorrect adjustment) by the MI ratio for the specific cancer (#1 eFigure 2). To propagate uncertainty from the MI ratios and the mortality estimates to incidence, this process was done at the 1,000-draw level. It was assumed that uncertainty in the MI ratio is independent of uncertainty in the estimated age-specific death rates.

Prevalence and YLD estimation

Prevalence is estimated as 10-year prevalence for all cancers as in GBD 2013 and GBD 2015.[15] To estimate cancer prevalence, relative cancer survival was estimated by scaling cancer-specific survival between a "best case" and "worst case" survival. The methods and input data used to generate the best and worst case survival as well as to scale countries between these boundaries remained the same as for the GBD 2013 and GBD 2015 studies (# 2, 3, and 5 in the flowchart).[15] Since the cause "other leukemia" was added for GBD 2016, survival data were updated using SEER 1973 survival data for "other leukemia" as the worst-case scenario and SEER 2010 survival data as the best-case survival. To transform relative to absolute survival (adjusting for background mortality) GBD 2016 lifetables were used (# 6 and 7 in the flowchart).[16] The access to cancer care variable to scale countries between the

© 2018 Fitzmaurice C et al. *JAMA Oncology*.

best and worst case survival was estimated using the same method as for GBD 2013 and GBD 2015 (# 4 in the flowchart):[15]

$$Access\ to\ care = 1 - \frac{Age\ standardized\ MI\ ratio_{cys} - Age\ standardized\ MI\ ratio_{min}}{Age\ standardized\ MI\ ratio_{max} - Age\ standardized\ MI\ ratio_{min}}$$

c=country; y=year; s=sex; Age-standardized MI $ratio_{min}$=lowest MI ratio for all countries and years; Age-standardized MI $ratio_{max}$=highest MI ratio for all countries and years

© 2018 Fitzmaurice C et al. JAMA Oncology.

Duration of the treatment phases (1. diagnosis and primary therapy; 2. Controlled phases; 3. Metastatic phase; 4. Terminal phase) remained the same as for GBD 2013 and GBD 2015, with the exception of the "other leukemia" cause, which was added for GBD 2016 (eTable 12). Total prevalence time was divided into phases 1, 3, and 4 for the population that died within 10 years, and the remaining prevalence was attributed to the controlled phase. For the population that survived beyond 10 years, prevalence person time was attributed to phase 1 and phase 2 (#8 in the flowchart). YLDs were calculated by multiplying each phase with the respective disability weight (eTable 13). To generate the total YLDs for each cancer (with the exception of cancers where additional disability is added due to procedures – see next paragraph) the YLDs for each cancer sequela were added (step 13 in eFigure 2).

Additional disability was estimated for breast cancer (disability due to mastectomy), larynx cancer (disability due to laryngectomy), colon and rectum cancer (disability due to stoma), bladder cancer (disability due to incontinence), and prostatectomy (disability due to incontinence and impotence) (#10 in eFigure 2). Hospital data were used to estimate the number of cancer patients undergoing mastectomy, laryngectomy, stoma, prostatectomy, and cystectomy. These proportions remained the same as in GBD 2013 and GBD 2015 and were used as input for proportion models that were run in DisMod-MR 2.0 (#9 in eFigure 2).[15,17] The procedure proportions (proportion of cancer population that undergoes procedures) from hospital data was used as input for a proportion model in DisMod-MR 2.0 in order to estimate the proportions for all locations, by age, and by sex.

Since colostomy or ileostomy procedures are done for reasons other than cancer, a literature review was done to determine the proportion of ostomies due to colorectal cancer. The "all cause" colostomy proportions were multiplied by 0.58 based on the results of the literature review showing that on average 58% of ostomies are done for colorectal cancer. [18–20]

The final procedure proportions were applied to the incidence cases of the respective cancers and multiplied with the proportion of the incidence population surviving for 10 years to determine the incident cases of the cancer population that underwent procedures. These incident cases were used again as an input for DisMod-MR 2.1, with a remission specification of zero and an excess mortality rate prior of 0 to 0.1. This approach was different compared to GBD 2015, where we used the cause-specific mortality of the specific cancer to obtain prevalence of the sequela. The approach was changed since the mortality for the population undergoing disease-specific procedures (e.g., mastectomy for breast cancer) is likely closer to the general population after they have survived for a period of time compared to the cause-specific mortality of the underlying disease (e.g., breast cancer).

Since disability associated with prostatectomy comes from impotence and incontinence and not from the prostatectomy itself, 18% of the prostatectomy prevalence was assumed to be incontinent and 55% was assumed to be impotent based on a literature review done for GBD 2013.[21–28]

Since all sequelae for a cause need to be mutually exclusive, the controlled phase for the cancers with additional procedure-related disability was adjusted to only include the population without procedure-related disability (= controlled phases prevalence of the total population – controlled phase prevalence of the proportion that experienced procedure related disability) (#11 in eFigure 2). The disability weight for the prevalence of the population that experiences additional disability was adjusted to reflect the combined disability of the controlled phase as well as the procedure.

© 2018 Fitzmaurice C et al. JAMA Oncology.

Lastly, the procedure sequelae prevalence and general sequelae prevalence were multiplied with disability weights (eTable 13) for the procedures to obtain the number of YLDs (#10 in eFigure 2). The sum of these YLDs are the final YLD estimate associated with each cancer.

Probability of cancer

The cumulative probability of developing cancer for certain age groups and an approximated lifetime risk for all cancer groups (age 0 to 79) as well as the odds of developing cancer for 2016 were calculated. The method use does not take into account competing risks of death. The cancer risk is approximated using the following formula[29]:

$Cumulative\ risk = 1 - e^{-cumulative\ rate}$

© 2018 Fitzmaurice C et al. JAMA Oncology.

References

1. Doll R, Payne P, Waterhouse J. *Cancer Incidence in Five Continents I*. Geneva: Union Internationale Contre le Cancer; 1966.

2. Doll R, Muir C, Waterhouse J. *Cancer Incidence in Five Continents II*. Geneva: Union Internationale Contre le Cancer, Geneva; 1970.

3. Waterhouse J, Muir C, Correa P, Powell J. *Cancer Incidence in Five Continents III*. Lyon: IARC; 1976.

4. Waterhouse J, Muir C, Shanmugaratnam K, Powell J. *Cancer Incidence in Five Continents IV*. Lyon: IARC; 1982.

5. Muir C, Mack T, Powell J, Whelan S. *Cancer Incidence in Five Continents V*. Lyon: IARC; 1987.

6. Parkin D, Muir C, Whelan S, Gao Y, Ferlay J, Powell J. *Cancer Incidence in Five Continents VI*. Lyon: IARC; 1992.

7. Parkin D, Whelan S, Ferlay J, Raymond L, Young J. *Cancer Incidence in Five Continents VII*. Lyon: IARC; 1997.

8. Parkin D, Whelan S, Ferlay J, Teppo L, Thomas D. *Cancer Incidence in Five Continents VIII*. Lyon: IARC; 2002.

9. Curado M, Edwards B, Shin H, et al. *Cancer Incidence in Five Continents IX*. Lyon: IARC; 2007. http://www.iarc.fr/en/publications/pdfs-online/epi/sp160/CI5vol9-A.pdf.

10. Forman D, Bray F, Brewster D, et al. Cancer Incidence in Five Continents X. http://ci5.iarc.fr. Published 2013.

11. Steliarova-Foucher E, O'Callaghan M, Ferlay J, Masuyer E, Forman D, Comber H, Bray F. European Cancer Observatory: Cancer Incidence, Mortality, Prevalence and Survival in Europe. International Agency for Research on Cancer. http://eco.iarc.fr. Accessed August 10, 2016.

12. Engholm G, Ferlay J, Christensen N, et al. NORDCAN--a Nordic tool for cancer information, planning, quality control and research. *Acta Oncol*. 2010;49(5):725-736. doi:10.3109/02841861003782017.

13. Abajobir AA, Abbafati C, Abbas KM, et al. Global, regional, and national age-sex specific mortality for 264 causes of death, 1980–2016: a systematic analysis for the Global Burden of Disease Study 2016. *The Lancet*. 2017;390(10100):1151-1210. doi:10.1016/S0140-6736(17)32152-9.

14. Foreman KJ, Lozano R, Lopez AD, Murray CJ. Modeling causes of death: an integrated approach using CODEm. *Popul Health Metr*. 2012;10(1):1. doi:10.1186/1478-7954-10-1.

15. Fitzmaurice C, Dicker D, Pain A, et al. The Global Burden of Cancer 2013. *JAMA Oncol*. May 2015. doi:10.1001/jamaoncol.2015.0735.

16. GBD Mortality and Causes of Death Collaborators. Global, regional, and national life expectancy, all-cause and cause specific mortality for 249 causes of death, 1980–2015: a systematic analysis for the Global Burden of Disease Study 2015. *The Lancet*. August 2016.

© 2018 Fitzmaurice C et al. JAMA Oncology.

17. Flaxman AD, Vos T, Murray C. *An Integrative Metaregression Framework for Descriptive Epidemiology*. University of Washington Press; 2015.

18. Canova C, Giorato E, Roveron G, Turrini P, Zanotti R. Validation of a stoma-specific quality of life questionnaire in a sample of patients with colostomy or ileostomy. *Colorectal Dis Off J Assoc Coloproctology G B Irel*. 2013;15(11):e692-698. doi:10.1111/codi.12324.

19. Caricato M, Ausania F, Ripetti V, Bartolozzi F, Campoli G, Coppola R. Retrospective analysis of long-term defunctioning stoma complications after colorectal surgery. *Colorectal Dis Off J Assoc Coloproctology G B Irel*. 2007;9(6):559-561. doi:10.1111/j.1463-1318.2006.01187.x.

20. Erwin-Toth P, Thompson SJ, Davis JS. Factors impacting the quality of life of people with an ostomy in North America: results from the Dialogue Study. *J Wound Ostomy Cont Nurs Off Publ Wound Ostomy Cont Nurses Soc WOCN*. 2012;39(4):417-422; quiz 423-424. doi:10.1097/WON.0b013e318259c441.

21. Catalona WJ, Carvalhal GF, Mager DE, Smith DS. Potency, continence and complication rates in 1,870 consecutive radical retropubic prostatectomies. *J Urol*. 1999;162(2):433-438.

22. Donnellan SM, Duncan HJ, MacGregor RJ, Russell JM. Prospective assessment of incontinence after radical retropubic prostatectomy: objective and subjective analysis. *Urology*. 1997;49(2):225-230. doi:10.1016/S0090-4295(96)00451-7.

23. Eastham JA, Kattan MW, Rogers E, et al. Risk factors for urinary incontinence after radical prostatectomy. *J Urol*. 1996;156(5):1707-1713.

24. Kundu SD, Roehl KA, Eggener SE, Antenor JAV, Han M, Catalona WJ. Potency, continence and complications in 3,477 consecutive radical retropubic prostatectomies. *J Urol*. 2004;172(6 Pt 1):2227-2231.

25. Potosky AL, Davis WW, Hoffman RM, et al. Five-Year Outcomes After Prostatectomy or Radiotherapy for Prostate Cancer: The Prostate Cancer Outcomes Study. *JNCI J Natl Cancer Inst*. 2004;96(18):1358-1367. doi:10.1093/jnci/djh259.

26. Sacco E, Prayer-Galetti T, Pinto F, et al. Urinary incontinence after radical prostatectomy: incidence by definition, risk factors and temporal trend in a large series with a long-term follow-up. *BJU Int*. 2006;97(6):1234-1241. doi:10.1111/j.1464-410X.2006.06185.x.

27. Stanford JL, Feng Z, Hamilton AS, et al. Urinary and sexual function after radical prostatectomy for clinically localized prostate cancer: the Prostate Cancer Outcomes Study. *JAMA*. 2000;283(3):354-360.

28. Walsh PC, Marschke P, Ricker D, Burnett AL. Patient-reported urinary continence and sexual function after anatomic radical prostatectomy. *Urology*. 2000;55(1):58-61.

29. Esteve J, Benhamou E, Raymond L. *Descriptive Epidemiology*. Vol VI. IARC Scientific Publications No.128. Lyon, France: IARC Publications; 1994.

© 2018 Fitzmaurice C et al. JAMA Oncology.

30. Neal RD, Din NU, Hamilton W, et al. Comparison of cancer diagnostic intervals before and after implementation of NICE guidelines: analysis of data from the UK General Practice Research Database. *Br J Cancer*. 2014;110(3):584-592. doi:10.1038/bjc.2013.791.

31. *Surveillance, Epidemiology, and End Results (SEER) Program (Www.seer.cancer.gov) SEER*Stat Database: Incidence - SEER 18 Regs Research Data + Hurricane Katrina Impacted Louisiana Cases, Nov 2012 Sub (1973-2010 Varying) - Linked To County Attributes - Total U.S., 1969-2011 Counties, National Cancer Institute, DCCPS, Surveillance Research Program, Surveillance Systems Branch, Released April 2013, Based on the November 2012 Submission.*

32. Allgar VL, Neal RD. Delays in the diagnosis of six cancers: analysis of data from the National Survey of NHS Patients: Cancer. *Br J Cancer*. 2005;92(11):1959-1970. doi:10.1038/sj.bjc.6602587.

33. Neal RD, Cannings-John R, Hood K, et al. Excision of malignant melanomas in North Wales: effect of location and surgeon on time to diagnosis and quality of excision. *Fam Pract*. 2008;25(4):221-227. doi:10.1093/fampra/cmn036.

34. Nolan RC, Chan MT-L, Heenan PJ. A clinicopathologic review of lethal nonmelanoma skin cancers in Western Australia. *J Am Acad Dermatol*. 2005;52(1):101-108. doi:10.1016/j.jaad.2004.08.016.

35. Kewalramani T, Nimer SD, Zelenetz AD, et al. Progressive disease following autologous transplantation in patients with chemosensitive relapsed or primary refractory Hodgkin's disease or aggressive non-Hodgkin's lymphoma. *Bone Marrow Transplant*. 2003;32(7):673-679. doi:10.1038/sj.bmt.1704214.

36. Esteban D, Tovar N, Jiménez R, et al. Patients with relapsed/refractory chronic lymphocytic leukaemia may benefit from inclusion in clinical trials irrespective of the therapy received: a case-control retrospective analsysis. *Blood Cancer J*. 2015;5:e356. doi:10.1038/bcj.2015.78.

© 2018 Fitzmaurice C et al. JAMA Oncology.

eTable 1: GATHER Guidelines checklist

**Objectives and funding**	**Reported in the manuscript/appendix**
1 Define the indicator(s), populations (including age, sex, and geographic entities), and time period(s) for which estimates were made.	See appendix: "Definition of indicator"
2 List the funding sources for the work.	See main manuscript
**Data Inputs**	
**For all data inputs from multiple sources that are synthesized as part of the study:**	
3 Describe how the data were identified and how the data were accessed.	See appendix: "Data sources"
4 Specify the inclusion and exclusion criteria. Identify all ad-hoc exclusions.	See appendix: "Data sources"
5 Provide information about all included data sources and their main characteristics. For each data source used, report reference information or contact name/institution, population represented, data collection method, year(s) of data collection, sex and age range, diagnostic criteria or measurement method, and sample size, as relevant.	http://ghdx.healthdata.org/gbd-2016/data-input-sources
6 Identify and describe any categories of input data that have potentially important biases (e.g., based on characteristics listed in item 5).	See appendix: "Bias of categories of input data"
**For data inputs that contribute to the analysis but were not synthesized as part of the study:**	
7 Describe and give sources for any other data inputs.	http://ghdx.healthdata.org/gbd-2016/data-input-sources
**For all data inputs:**	
8 Provide all data inputs in a file format from which data can be efficiently extracted (e.g., a spreadsheet rather than a PDF), including all relevant meta-data listed in item 5. For any data inputs that cannot be shared because of ethical or legal reasons, such as third-party ownership, provide a contact name or the name of the institution that retains the right to the data.	http://ghdx.healthdata.org/gbd-2016/data-input-sources
**DATA ANALYSIS**	
9 Provide a conceptual overview of the data analysis method. A diagram may be helpful.	See eFigure 1: Flowchart GBD cancer mortality, YLL estimation<br>See eFigure 2: Flowchart GBD cancer incidence, prevalence, YLD estimation
10 Provide a detailed description of all steps of the analysis, including mathematical formulae. This description should cover, as relevant, data cleaning, data pre-processing, data adjustments and weighting of data sources, and mathematical or statistical model(s).	See appendix: "Data analysis"
11 Describe how candidate models were evaluated and how the final model(s) were selected.	See appendix "CODEm models"<br>See eTable 8: Covariates selected for CODEm for each GBD cancer group

© 2018 Fitzmaurice C et al. JAMA Oncology.

	and expected direction of covariate
12 Provide the results of an evaluation of model performance, if done, as well as the results of any relevant sensitivity analysis.	See eTable 10: Results for CODEm model testing
13 Describe methods of calculating uncertainty of the estimates. State which sources of uncertainty were, and were not, accounted for in the uncertainty analysis.	See appendix "Data analysis"
14 State how analytic or statistical source code used to generate estimates can be accessed.	http://ghdx.healthdata.org/gbd-2016-code
**RESULTS AND DISCUSSION**	
15 Provide published estimates in a file format from which data can be efficiently extracted.	GBD 2016 estimates are available online (http://vizhub.healthdata.org/gbd-compare).
16 Report a quantitative measure of the uncertainty of the estimates (e.g., uncertainty intervals).	Done
17 Interpret results in light of existing evidence. If updating a previous set of estimates, describe the reasons for changes in estimates.	eTable 2: Sources for cancer incidence and mortality-to-incidence ratio data by country, year, and registry<br>eTable 9: Comparison of GBD 2015 and GBD 2016 covariates used and level of covariates
18 Discuss limitations of the estimates. Include a discussion of any modelling assumptions or data limitations that affect interpretation of the estimates.	See main manuscript "Limitations"

© 2018 Fitzmaurice C et al. JAMA Oncology.

eTable 2: Sources for cancer incidence and mortality-to-incidence ratio data by country, year, and registry

Location	Registry	Years available from registry	Years used for incidence	Years available for MI ratio	Years used for MI ratio
Algeria	Algiers	1993-1997	5	1993-1997	0
Algeria	Batna	2000-2006	7	2000-2006	0
Algeria	Oran	2005-2006	2	2005-2006	0
Algeria	Setif	1986-2007	18	1986-2007	0
Antilles except Aruba	Antilles except Aruba	1973-1982	0	NA	NA
Argentina	Bahia Blanca	1993-2007	15	1993-2007	0
Argentina	Concordia	1990-1997	10	1990-1997	0
Argentina	Cordoba	2003-2007	4	2004-2007	0
Argentina	Mendoza	2003-2007	5	2003-2007	0
Argentina	Tierra del Fuego	2003-2007	5	2003-2007	0
Australia	Capital Territory	1978-2007	25	1983-2007	0
Australia	National Registry	1982-2007	26	1968-2007	26
Australia	New South Wales	1973-2007	25	1983-2007	0
Australia	Northern Territory	1993-2007	10	1998-2007	0
Australia	Queensland	1982-2007	15	1993-2007	0
Australia	South Australia	1977-2007	31	1977-2007	0
Australia	Tasmania	1978-2007	30	1978-2007	0
Australia	Victoria	1982-2007	25	1983-2007	0
Australia	Western Australia	1982-2007	25	1983-2007	0
Austria	National Registry	1983-2010	31	1983-2010	7
Austria	Salzburg	NA	NA	1999-2006	0
Austria	Tyrol	1988-2007	0	1988-2007	0
Austria	Vorarlberg	1993-2007	0	1993-2007	0
Bahrain	National Registry	1998-2007	10	1998-2007	0
Belarus	National Registry	1983-2007	25	1983-2007	0
Belgium	Antwerp	1998-2002	5	1998-2002	0

© 2018 Fitzmaurice C et al. JAMA Oncology.

Location	Registry	Years available from registry	Years used for incidence	Years available for MI ratio	Years used for MI ratio
Belgium	Flanders	1998-2001	4	1998-2001	0
Belgium	Flanders except Limburg	1997-1998	2	1997-1998	0
Belgium	Limburg	1997-1998	2	1997-1998	0
Belgium	National Registry	2003-2010	7	2003-2010	0
Bermuda	Bermuda	1983-1987	5	1983-1987	0
Brazil	Aracaju	1996-2012	17	1996-2012	0
Brazil	Barretos	2008-2013	6	2008-2013	0
Brazil	Belem	1989-2009	17	1989-2009	0
Brazil	Belo Horizonte	2000-2008	9	2000-2008	0
Brazil	Brasilia	1998-2001	4	1998-2001	0
Brazil	Campinas	1991-2005	15	1991-2005	0
Brazil	Campo Grande	2000-2009	6	2000-2009	0
Brazil	Cuiaba	2000-2007	8	2000-2007	0
Brazil	Curitiba	1998-2010	13	1998-2010	0
Brazil	Distrito Federal	1999-2002	4	1999-2002	0
Brazil	Espirito Santo	1997-2012	16	1997-2012	0
Brazil	Florianopolis	2008-2012	5	2008-2012	0
Brazil	Fortaleza	1978-2006	25	1978-2006	0
Brazil	Goiania	1988-2009	22	1988-2009	0
Brazil	Jahu	1996-2013	18	1996-2013	0
Brazil	Joao Pessoa	1999-2010	12	1999-2010	0
Brazil	Manaus	1999-2006	8	1999-2006	0
Brazil	Mato Grosso Interior	2001-2005	5	2001-2005	0
Brazil	Natal	1999-2005	7	1999-2005	0
Brazil	Palmas	2000-2012	13	2000-2012	0
Brazil	Pocos de Caldas	2007-2011	5	2007-2011	0
Brazil	Porto Alegre	1979-2006	22	1979-2006	0
Brazil	Recife	1968-2010	17	1968-2010	0

© 2018 Fitzmaurice C et al. JAMA Oncology.

Location	Registry	Years available from registry	Years used for incidence	Years available for MI ratio	Years used for MI ratio
Brazil	Roraima	2003-2010	8	2003-2010	0
Brazil	Salvador	1996-2005	10	1996-2005	0
Brazil	Santos	2008-2009	2	2008-2009	0
Brazil	Sao Paulo	1969-2011	17	1969-2011	0
Brazil	Teresina	2000-2006	7	2000-2006	0
Bulgaria	National Registry	1993-2010	12	1993-2010	12
Canada	Alberta	1960-2007	32	1960-2007	0
Canada	British Columbia	1969-2007	32	1969-2007	0
Canada	Manitoba	1958-2007	32	1958-2007	0
Canada	Maritime	1969-1987	9	1969-1987	0
Canada	National Registry	1978-2007	30	1978-2007	0
Canada	New Brunswick	1962-2007	25	1962-2007	0
Canada	Newfoundland	1969-2002	26	1969-2002	0
Canada	Newfoundland and Labrador	1960-2007	9	1960-2007	0
Canada	Northwest Territories	1983-2007	0	1983-2007	0
Canada	Northwest Territories and Yukon	1973-1987	5	1973-1987	0
Canada	Nova Scotia	1978-2007	20	1978-2007	0
Canada	Ontario	1969-2007	23	1969-2007	0
Canada	Prince Edward Island	1978-2007	16	1978-2007	0
Canada	Quebec	1963-2007	13	1963-2007	0
Canada	Saskatchewan	1960-2007	34	1960-2007	0
Canada	Yukon	1983-2007	0	1983-2007	0
Chile	Antofagasta	2003-2007	0	2003-2007	0
Chile	Bio Bio	2003-2007	5	2003-2007	0
Chile	Los Rios	2003-2007	5	2003-2007	0
Chile	National Registry	1959-1961	0	1959-1961	0
Chile	Valdivia	1998-2007	10	1998-2007	0
China	Anshan	1998-2011	11	1998-2011	13

© 2018 Fitzmaurice C et al. JAMA Oncology.

Location	Registry	Years available from registry	Years used for incidence	Years available for MI ratio	Years used for MI ratio
China	Baoding	2009-2011	2	2009-2011	2
China	Beijing	1990-2011	22	1990-2011	21
China	Beijing Rural Areas	2011	0	2011	0
China	Beiliu	2011	1	2011	1
China	Bengbu	2011	1	2011	1
China	Benxi	2003-2011	8	2003-2011	8
China	Bijiang District, Tongren	2011	1	2011	1
China	Bincheng District, Binzhou	2011	1	2011	1
China	Binghai	2011	1	2011	1
China	Boli	2011	1	2011	1
China	Cangwu	2011	1	2011	1
China	Cangzhou	2011	1	2011	1
China	Changfeng	2011	1	2011	1
China	Changle	1990-2011	21	1990-2011	21
China	Changning	2011	1	2011	1
China	Changzhou	2011	1	2011	1
China	Chifeng	2009-2011	2	2009-2011	2
China	Chuzhou District, Huai'an	2004-2007	3	2004-2007	0
China	Ci County	1990-2009	23	1990-2009	12
China	Cili	2011	1	2011	1
China	Cixi	2011	1	2011	1
China	Cixian	2011	1	2011	1
China	Daan	2011	1	2011	1
China	Dafeng	2003-2011	6	2003-2011	8
China	Dalian City	1998-2011	12	1998-2011	13
China	Dancheng	2011	1	2011	1
China	Dandong	2008-2011	3	2008-2011	3
China	Daoli District, Harbin City	2005-2011	5	2005-2011	6

© 2018 Fitzmaurice C et al. JAMA Oncology.

Location	Registry	Years available from registry	Years used for incidence	Years available for MI ratio	Years used for MI ratio
China	Dawukou	2011	1	2011	1
China	Dazhu	2011	1	2011	1
China	Decheng District, Dezhou	2011	1	2011	1
China	Dehui	2009-2011	2	2009-2011	2
China	Dingan	2011	1	2011	1
China	Donggang	2009-2011	2	2009-2011	2
China	Donghai County	2004-2011	3	2004-2011	2
China	Dunhuang	2011	1	2011	1
China	Faku	2011	1	2011	1
China	Feicheng	1998-2011	13	1998-2011	13
China	Feidong	2011	1	2011	1
China	Feixi County	2009-2011	2	2009-2011	2
China	Fusui County	1990-2011	14	1990-2011	8
China	Fuyuan	2011	1	2011	1
China	Ganyu	2004-2011	2	2004-2011	1
China	Ganzhou District, Zhangye	2011	1	2011	1
China	Gaomi	2011	1	2011	1
China	Gaotang	2011	1	2011	1
China	Gejiu	2004-2011	3	2004-2011	2
China	Gongan	2011	1	2011	1
China	Guangrao	2011	1	2011	1
China	Guangzhou City	2000-2011	9	2000-2011	11
China	Guannan	2011	1	2011	1
China	Guanyun County	2004-2011	3	2004-2011	3
China	Guilin	2011	1	2011	1
China	Guyuan	2011	1	2011	1
China	Hai'an County	2009-2011	2	2009-2011	2
China	Haimen	2003-2011	7	2003-2011	8

© 2018 Fitzmaurice C et al. JAMA Oncology.

Location	Registry	Years available from registry	Years used for incidence	Years available for MI ratio	Years used for MI ratio
China	Hainan	2011	1	2011	1
China	Haining	1998-2011	11	1998-2011	13
China	Hangzhou City	2000-2011	9	2000-2011	11
China	Hanjiang District, Putian	2011	1	2011	1
China	Hefei	2011	1	2011	1
China	Hengdong County	2009-2011	2	2009-2011	2
China	Hepu	2011	1	2011	1
China	Hetian	2011	1	2011	1
China	Hong Kong Special Administrative Region of China	1974-2013	43	1974-2013	8
China	Hongta District, Yuxi	2011	1	2011	1
China	Hongtong	2011	1	2011	1
China	Hongze	2011	1	2011	1
China	Huai'an District, Huai'an	1998-2009	12	1998-2009	12
China	Huaiyin District, Huai'an	2009-2011	2	2009-2011	2
China	Huangdao District, Qingdao	2011	1	2011	1
China	Huian	2011	1	2011	1
China	Huichuan District, Zunyi	2011	1	2011	1
China	Huinong	2011	1	2011	1
China	Huixian	2011	1	2011	1
China	Huzhu	2011	1	2011	1
China	Jiangmen	2011	1	2011	1
China	Jianhu County	2003-2011	6	2003-2011	8
China	Jianou	2011	1	2011	1
China	Jianping	2011	1	2011	1
China	Jiashan County	1990-2011	22	1990-2011	21
China	Jiaxing	2000-2011	10	2000-2011	11
China	Jilin	2011	1	2011	1

© 2018 Fitzmaurice C et al. JAMA Oncology.

Location	Registry	Years available from registry	Years used for incidence	Years available for MI ratio	Years used for MI ratio
China	Jinan	2011	1	2011	1
China	Jingan	2011	1	2011	1
China	Jingtai County	2009-2011	2	2009-2011	2
China	Jingxian	2011	1	2011	1
China	Jingyang	2011	1	2011	1
China	Jinhu County	2007-2011	3	2007-2011	4
China	Jintan District	2003-2011	6	2003-2011	7
China	Jinzhai	2011	1	2011	1
China	Jiulongpo District, Chongqing	2004-2011	3	2004-2011	4
China	Jiyuan	2011	1	2011	1
China	Junan	2011	1	2011	1
China	Kaihua	2011	1	2011	1
China	Kailu	2011	1	2011	1
China	Kaiyang	2011	1	2011	1
China	Kangping	2011	1	2011	1
China	Kunes County	2009	1	2009	1
China	Lanping	2011	1	2011	1
China	Lanzhou	2011	1	2011	1
China	Leishan	2011	1	2011	1
China	Leshan	2011	1	2011	1
China	Lhasa	2011	1	2011	1
China	Liangzhou District	2008-2011	3	2008-2011	3
China	Lianhu District, Xi'an	2011	1	2011	1
China	Lianshui	2011	1	2011	1
China	Lianyungang	2004-2011	4	2004-2011	4
China	Lingbi	2011	1	2011	1
China	Linhe District, Bayannaoer	2011	1	2011	1
China	Linqu County	1998-2011	11	1998-2011	13

© 2018 Fitzmaurice C et al. JAMA Oncology.

Location	Registry	Years available from registry	Years used for incidence	Years available for MI ratio	Years used for MI ratio
China	Lintan	2011	1	2011	1
China	Linxian	2011	1	2011	1
China	Linzhou	1990-2011	19	1990-2011	21
China	Liuzhou	2009-2011	2	2009-2011	2
China	Liyang	2011	1	2011	1
China	Longnan	2011	1	2011	1
China	Longquanyi District, Chengdu	2011	1	2011	1
China	Lujiang	2011	1	2011	1
China	Luoshan	2011	1	2011	1
China	Luoyang	2011	1	2011	1
China	Lushan	2011	1	2011	1
China	Ma'anshan	2003-2011	6	2003-2011	8
China	Macao Special Administrative Region of China	2003-2007	5	2003-2007	0
China	Macheng	2011	1	2011	1
China	Maiji District, Tianshui	2011	1	2011	1
China	Mayang	2011	1	2011	1
China	Meixian	2011	1	2011	1
China	Minhe	2011	1	2011	1
China	Naidong	2011	1	2011	1
China	Nangang District, Harbin City	1992-2011	20	1992-2011	14
China	Nantong	2011	1	2011	1
China	Neixiang	2011	1	2011	1
China	Ningyang	2011	1	2011	1
China	Nongqishi	2011	1	2011	1
China	Pengzhou	2011	1	2011	1
China	Pingluo	2011	1	2011	1
China	Qianxi County	2009-2011	2	2009-2011	2
China	Qidong County	1983-2011	35	1983-2011	21

© 2018 Fitzmaurice C et al. JAMA Oncology.

Location	Registry	Years available from registry	Years used for incidence	Years available for MI ratio	Years used for MI ratio
China	Qingdao	2011	1	2011	1
China	Qinghe	2011	1	2011	1
China	Qingpu	2011	1	2011	1
China	Qingyang District, Chengdu	2009-2011	2	2009-2011	2
China	Qinhuangdao	2011	1	2011	1
China	Qionghai	2011	1	2011	1
China	Renhe District, Panzhihua	2011	1	2011	1
China	Rushan	2011	1	2011	1
China	Sanmenxia	2011	1	2011	1
China	Sanya	2011	1	2011	1
China	Shangdong	1993-1997	0	NA	NA
China	Shanggao	2011	1	2011	1
China	Shanghai	1975-2011	31	1975-2011	21
China	Shangyu	2009-2011	2	2009-2011	2
China	Shangzhi	2009-2011	2	2009-2011	2
China	Shangzhou District, Shangluo	2011	1	2011	1
China	Shapingba District, Chongqing	2011	1	2011	1
China	Shenqiu	2011	1	2011	1
China	Shenyang City	2003-2011	7	2003-2011	8
China	Shenzen City	2004-2011	2	2004-2011	1
China	Shexian County	2003-2011	8	2003-2011	8
China	Sheyang County	2008-2011	3	2008-2011	3
China	Shifeng District, Zhuzhou	2011	1	2011	1
China	Shihezi	2011	1	2011	1
China	Shouxian	2011	1	2011	1
China	Shouyang	2011	1	2011	1
China	Sihui	1998-2011	11	1998-2011	13
China	Suzhou	2006-2011	4	2006-2011	5

© 2018 Fitzmaurice C et al. JAMA Oncology.

Location	Registry	Years available from registry	Years used for incidence	Years available for MI ratio	Years used for MI ratio
China	Taixing	2004-2011	4	2004-2011	6
China	Tengchong	2011	1	2011	1
China	Tengzhou	2011	1	2011	1
China	Tianchang	2011	1	2011	1
China	Tianjin	1981-2011	22	1981-2011	16
China	Tianjin Rural Areas	2011	0	2011	0
China	Tianshan District, Urumqi	2011	1	2011	1
China	Tong'an District, Xiamen	2011	1	2011	1
China	Tongguan	2011	1	2011	1
China	Tonghua	2011	1	2011	1
China	Tongling	2008-2011	3	2008-2011	3
China	Wanzhouqu District, Chongqing	2011	1	2011	1
China	Wenshang County	2009-2011	2	2009-2011	2
China	Wuan	2011	1	2011	1
China	Wufeng	2011	1	2011	1
China	Wuhan City	1990-2011	22	1990-2011	18
China	Wuhu	2011	1	2011	1
China	Wuning	2011	1	2011	1
China	Wuwei	2004	1	2004	0
China	Wuxi	2006-2011	2	2006-2011	2
China	Xiamen City	2009-2011	2	2009-2011	2
China	Xiang'an District, Xiamen	2011	1	2011	1
China	Xiangfang District, Harbin	2011	1	2011	1
China	Xianju County	2009-2011	2	2009-2011	2
China	Xilinhaote	2011	1	2011	1
China	Xinghualing District, Taiyuan	2011	1	2011	1
China	Xining	2009-2011	2	2009-2011	2
China	Xinyuan	2011	1	2011	1

© 2018 Fitzmaurice C et al. JAMA Oncology.

Location	Registry	Years available from registry	Years used for incidence	Years available for MI ratio	Years used for MI ratio
China	Xinzhou District, Shangrao	2011	1	2011	1
China	Xiping County	2009-2011	2	2009-2011	2
China	Xuanwei	2011	1	2011	1
China	Xuyi County	2009-2011	2	2009-2011	2
China	Xuzhou	2011	1	2011	1
China	Yakeshi	2011	1	2011	1
China	Yancheng	2011	1	2011	1
China	Yancheng District, Luohe	2011	1	2011	1
China	Yangcheng County	2003-2011	6	2003-2011	8
China	Yangquan	2009-2011	2	2009-2011	2
China	Yangshan	2011	1	2011	1
China	Yangzhong	1998-2011	12	1998-2011	13
China	Yanji	2009-2011	2	2009-2011	2
China	Yanshi	2009-2011	2	2009-2011	2
China	Yantai	2011	1	2011	1
China	Yanting County	1998-2011	10	1998-2011	13
China	Yinchuan	2011	1	2011	1
China	Yingdong District, Fuyang	2011	1	2011	1
China	Yingshan	2011	1	2011	1
China	Yiyuan	2011	1	2011	1
China	Yongding	2011	1	2011	1
China	Yongqiao District, Suzhou	2011	1	2011	1
China	Yuanhui District, Luohe	2011	1	2011	1
China	Yuanqu	2011	1	2011	1
China	Yucheng	2011	1	2011	1
China	Yuci District, Jinzhong	2011	1	2011	1
China	Yueyanglou	2011	1	2011	1
China	Yunmeng County	2009-2011	2	2009-2011	2

© 2018 Fitzmaurice C et al. JAMA Oncology.

Location	Registry	Years available from registry	Years used for incidence	Years available for MI ratio	Years used for MI ratio
China	Yuzhong District, Chongqing	2011	1	2011	1
China	Yuzhou	2011	1	2011	1
China	Zanhuang	2011	1	2011	1
China	Zhanggong District	2009	1	2009	1
China	Zhanggong District, Ganzhou	2011	1	2011	1
China	Zhangqiu	2011	1	2011	1
China	Zhaoling District, Luohe	2011	1	2011	1
China	Zhaoyuan	2011	1	2011	1
China	Zhongshan	1998-2011	12	1998-2011	13
China	Zhongshan County	2004-2007	3	2004-2007	0
China	Zhongwei	2011	1	2011	1
China	Zhongxiang	2011	1	2011	1
China	Zhuanghe	2009-2011	2	2009-2011	2
China	Zhuhai	2011	1	2011	1
China	Ziliujing District	2009	1	2009	1
China	Ziliujing District, Zigong	2011	1	2011	1
China	Zixing	2011	1	2011	1
China	Zoucheng	2011	1	2011	1
Colombia	Bucaramanga	2003-2007	5	2003-2007	0
Colombia	Cali	1962-2007	40	1962-2007	0
Colombia	Manizales	2003-2007	5	2003-2007	0
Colombia	National Registry	2003-2010	0	2003-2010	0
Colombia	Pasto	2003-2007	5	2003-2007	0
Costa Rica	National Registry	1980-2011	33	1980-2013	0
Cote d'Ivoire	National Registry	1995-1997	3	1995-1997	0
Croatia	National Registry	1988-2010	23	1988-2010	8
Cuba	National Registry	1968-1987	11	1968-1986	0
Cuba	Villa Clara	1995-2007	7	1995-2007	0

© 2018 Fitzmaurice C et al. JAMA Oncology.

Location	Registry	Years available from registry	Years used for incidence	Years available for MI ratio	Years used for MI ratio
Cyprus	National Registry	1998-2007	9	1998-2007	4
Czech Republic	National Registry	1983-2010	27	1983-2010	5
Denmark	National Registry	1953-2014	50	1953-2014	35
Ecuador	Cuenca	2003-2007	5	2003-2007	0
Ecuador	Quito	1985-2007	25	1985-2007	0
Egypt	Aswan	2008	1	2008	0
Egypt	Damietta	2009	1	2009	0
Egypt	Gharbiah	1999-2007	9	1999-2007	0
Egypt	Minia	2009	1	2009	0
Estonia	National Registry	1968-2011	44	1968-2009	5
Fiji	National Registry	1998-2010	13	1998-2010	11
Finland	National Registry	1953-2014	50	1953-2014	35
France	Bas Rhin	1975-2007	33	1975-2007	0
France	Calvados	1978-2007	30	1978-2007	0
France	Calvados Digestive	1978-2009	6	1978-2009	0
France	Cote d'Or	1980-2009	30	1980-2009	0
France	Doubs	1977-2009	36	1977-2009	0
France	Finistere Digestive	1984-2009	26	1984-2009	0
France	Haut Rhin	1988-2009	21	1988-2009	0
France	Herault	1987-2009	26	1987-2009	0
France	Isere	1979-2009	30	1979-2009	0
France	Loire Atlantique	1991-2009	18	1991-2009	0
France	Manche	1994-2009	19	1994-2009	0
France	Nord	2005-2009	3	2005-2009	0
France	Normandy	2002-2009	8	2002-2009	0
France	Somme	1982-2009	27	1982-2009	0
France	Tarn	1982-2009	27	1982-2009	0
France	Vendee	1998-2007	10	1998-2007	0

© 2018 Fitzmaurice C et al. JAMA Oncology.

Location	Registry	Years available from registry	Years used for incidence	Years available for MI ratio	Years used for MI ratio
French Polynesia	French Polynesia	1988-2002	0	NA	NA
Germany	Bavaria	2002-2007	0	2002-2007	0
Germany	Berlin	1998-2007	2	1998-2007	0
Germany	Brandenburg	1998-2007	2	1998-2007	0
Germany	Bremen	2000-2008	0	2000-2008	0
Germany	Eastern States (former GDR)	1964-1989	22	1964-1989	0
Germany	Free State of Saxony	1998-2007	2	1998-2007	0
Germany	Hamburg	1969-2009	16	1969-2010	2
Germany	Lower Saxony	2003-2007	0	2003-2007	0
Germany	Mecklenburg	1998-2007	2	1998-2007	0
Germany	Mecklenburg-West Pomerania	1998-2007	0	1998-2007	0
Germany	Munich	1998-2007	0	1998-2007	0
Germany	National Registry	2000-2010	11	2000-2010	11
Germany	North Rhine Westphalia	1998-2007	2	1994-2007	2
Germany	Rhineland Palatinate	2000-2007	0	2000-2007	0
Germany	Saarland	1968-2007	30	1968-2007	30
Germany	Saxony-Anhalt	1998-2007	2	1998-2007	0
Germany	Schleswig Holstein	1998-2007	2	1998-2007	2
Germany	Thuringen	1998-2007	2	1998-2007	0
Germany	Westphalia	1998-2007	0	1998-2007	0
Greece	National Registry	1990-1991	0	1990-1991	0
Greenland	Greenland	1980-2014	35	1980-2014	32
Grenada	St. George's Central Hospital	1996-2000	5	1996-2000	0
Guinea	Conakry	1992-1995	4	1992-1995	0
Hungary	County Szabolcs-Szatmar	1962-1987	18	1962-1987	0
Hungary	County Vas	1962-1987	20	1962-1987	0
Hungary	Miskolc	1962-1966	0	1962-1966	0
Hungary	National Registry	2001-2011	11	2001-2011	0

© 2018 Fitzmaurice C et al. JAMA Oncology.

Location	Registry	Years available from registry	Years used for incidence	Years available for MI ratio	Years used for MI ratio
Iceland	National Registry	1955-2014	50	1955-2014	35
India	Ahmedabad	1983-2005	12	NA	NA
India	Ahmedabad Rural	2006-2010	5	NA	NA
India	Ahmedabad Urban	2006-2013	7	NA	NA
India	Aizawl	2005-2014	5	NA	NA
India	Aurangabad	2005-2014	9	NA	NA
India	Bangalore	1982-2012	24	NA	NA
India	Barshi Expanded	2009-2012	2	NA	NA
India	Barshi Rural	1988-2014	20	NA	NA
India	Bhopal	2004-2013	11	NA	NA
India	Cachar	2007-2014	7	NA	NA
India	Chandigarh Union Territory	2013	1	NA	NA
India	Chennai	1982-2013	34	NA	NA
India	Delhi	1993-2012	21	NA	NA
India	Dibrugarh	2005-2014	11	NA	NA
India	Dindigul Ambilikkai	2003-2013	7	NA	NA
India	Imphal	2005-2014	5	NA	NA
India	Kamrup Urban	2005-2014	11	NA	NA
India	Karunagappally	1991-2007	17	NA	NA
India	Kolkata	2005-2012	6	NA	NA
India	Kollam	2006-2014	8	NA	NA
India	Manipur	2006-2010	5	NA	NA
India	Manipur Excl Imphal West	2009-2014	5	NA	NA
India	Mansa District	2013	1	NA	NA
India	Meghalaya	2010-2014	5	NA	NA
India	Mizoram	2003-2010	10	NA	NA
India	Mizoram Excl Aizawl	2005-2014	5	NA	NA
India	Mumbai	1964-2012	50	NA	NA

© 2018 Fitzmaurice C et al. JAMA Oncology.

Location	Registry	Years available from registry	Years used for incidence	Years available for MI ratio	Years used for MI ratio
India	Nagaland	2010-2014	4	NA	NA
India	Nagpur	1980-2013	20	NA	NA
India	Naharlagun Excl Papum Pare	2012-2014	3	NA	NA
India	Papum Pare	2012-2014	3	NA	NA
India	Pasighat	2012-2014	3	NA	NA
India	Patiala District	2012-2014	3	NA	NA
India	Pune	1973-2013	32	NA	NA
India	S.A.S Nagar District	2013	1	NA	NA
India	Sangrur District	2013	1	NA	NA
India	Sikkim	2003-2014	14	NA	NA
India	Silchar	2005-2006	2	NA	NA
India	Tamil Nadu	2012-2013	2	NA	NA
India	Tripura	2010-2014	4	NA	NA
India	Trivandrum	1991-2014	21	NA	NA
India	Wardha	2010-2014	5	NA	NA
Iran	Ardabil	1985-2008	8	1985-2008	0
Iran	Golestan	1996-2007	8	1996-2007	0
Iran	National Registry	2003-2007	8	2003-2007	0
Iraq	National Registry	2007-2011	3	2007-2011	0
Ireland	National Registry	1994-2010	16	1994-2010	0
Ireland	Southern Ireland	1980-1992	12	1980-1992	0
Israel	National Registry	1960-2010	13	1960-2010	0
Italy	Alto Adige	2003-2006	4	2003-2006	0
Italy	Biella	1995-2007	13	1995-2007	0
Italy	Brescia	1999-2007	7	1999-2006	0
Italy	Catania and Messina	2003-2005	3	2003-2005	0
Italy	Catanzaro	2003-2007	5	2003-2007	0
Italy	Como	2003-2007	5	2003-2007	0

© 2018 Fitzmaurice C et al. JAMA Oncology.

Location	Registry	Years available from registry	Years used for incidence	Years available for MI ratio	Years used for MI ratio
Italy	Ferrara	1991-2007	17	1991-2007	0
Italy	Florence	1985-2002	18	1985-2002	0
Italy	Florence and Prato	1985-2007	21	1985-2005	0
Italy	Friuli Venezia Giulia	2003-2007	5	2003-2007	0
Italy	Genoa	1986-2007	18	1986-2006	0
Italy	Latina	1983-2007	12	1983-2007	0
Italy	Lecco	2003-2007	5	2003-2007	0
Italy	Macerata	1991-2000	10	1991-2000	0
Italy	Mantua	2003-2007	3	2003-2005	0
Italy	Milan	1999-2007	8	1999-2006	0
Italy	Modena	1988-2007	23	1988-2007	0
Italy	Naples	1998-2007	10	1998-2007	0
Italy	National Registry	2006-2009	4	2006-2009	4
Italy	North East Italy	1995-2002	8	1995-2002	0
Italy	Nuoro	2003-2007	5	2003-2007	0
Italy	Palermo	2003-2007	4	2003-2006	0
Italy	Parma	1978-2007	30	1978-2007	0
Italy	Ragusa	1978-2007	31	1981-2007	0
Italy	Reggio Emilia	1998-2007	10	1998-2007	0
Italy	Romanga	1985-2007	22	1986-2007	0
Italy	Salerno	1998-2007	9	1998-2007	0
Italy	Sassari	1993-2007	15	1993-2007	0
Italy	Sondrio	1998-2007	14	1998-2007	0
Italy	South Lombard	2003-2005	3	2003-2005	0
Italy	South Tyrol	2003-2007	0	NA	NA
Italy	Syracuse	1999-2007	9	1999-2007	0
Italy	Torino	1984-2007	23	1985-2007	0
Italy	Trapani	2003-2006	4	2003-2006	0

© 2018 Fitzmaurice C et al. JAMA Oncology.

Location	Registry	Years available from registry	Years used for incidence	Years available for MI ratio	Years used for MI ratio
Italy	Trento	2003-2006	4	2003-2006	0
Italy	Trieste	1983-1992	6	1984-1992	0
Italy	Umbria	1994-2007	13	1994-2007	1
Italy	Varese	1976-2007	32	1976-2007	0
Italy	Veneto	1988-2007	17	1988-2006	0
Jamaica	National Registry	1958-2007	25	1958-2007	0
Japan	Aichi	1998-2007	11	1998-2007	1
Japan	Fukui	1998-2007	10	1998-2007	0
Japan	Fukuoka	1974-1975	2	1974-1975	0
Japan	Hiroshima	1978-2007	28	1978-2007	0
Japan	Miyagi	1959-2007	35	1959-2007	0
Japan	Nagasaki	1973-2007	35	1973-2007	0
Japan	National Registry	1975-2010	36	1958-2013	36
Japan	Niigata	2003-2007	5	2003-2007	0
Japan	Okayama	1966-1969	0	1966-1969	0
Japan	Osaka	1963-2007	38	1963-2007	0
Japan	Saga	1984-2007	18	1984-2007	0
Japan	Yamagata	1983-2002	23	1983-2002	0
Jordan	National Registry	2001-2008	8	2001-2008	0
Kenya	Nairobi	2000-2002	3	2000-2002	0
Kuwait	National Registry	1979-2007	28	1979-2007	0
Kyrgyzstan	National Registry	1986-1987	2	1986-1987	0
La Martinique	La Martinique	1981-2002	0	NA	NA
La Reunion	La Reunion	1988-1994	0	NA	NA
Latvia	National Registry	1983-2007	24	1983-2007	5
Lebanon	National Registry	1998-2007	4	1998-2007	0
Libya	Benghazi	2003-2005	3	2003-2005	0
Lithuania	National Registry	1978-2011	33	1978-2011	2

© 2018 Fitzmaurice C et al. JAMA Oncology.

Location	Registry	Years available from registry	Years used for incidence	Years available for MI ratio	Years used for MI ratio
Malawi	Blantyre	1994-2007	10	1994-2007	0
Malaysia	National Registry	2003	1	2003	0
Malaysia	Penang	1998-2007	9	1998-2007	0
Malaysia	Sarawak	1998-2002	5	1998-2002	0
Mali	Bamako	1987-1996	11	1987-1996	0
Malta	National Registry	1969-2010	27	1969-2010	15
Mongolia	National Registry	2003-2007	0	2003-2007	0
Morocco	Greater Casablanca	2004	1	2004	0
Mozambique	Lourenco Marques	1956-1960	0	1956-1960	0
Namibia	National Registry	2000-2009	10	2000-2009	0
Netherlands	Eindhoven	1973-2007	27	1973-2007	0
Netherlands	Maastricht	1986-2002	3	1986-2002	0
Netherlands	National Registry	1989-2015	19	1989-2007	0
Netherlands	Three Provinces	1960-1962	0	1960-1962	0
New Zealand	National Registry	1968-2014	32	1983-2014	5
Nigeria	Calabar	2009-2013	5	2009-2013	0
Nigeria	Ibadan	1960-1969	0	1960-1969	0
Nigeria	Midwestern Nigeria	NA	NA	2008-2009	0
Norway	National Registry	1953-2014	50	1953-2014	35
Oman	National Registry	1993-2012	20	1993-2012	0
Pakistan	South Karachi	1995-2002	8	1995-2002	0
Palestine	West Bank	2010-2011	2	2010-2011	0
Panama	National Registry	1988-2011	23	1988-2011	12
Paraguay	Asuncion Region	1988-1989	2	1988-1989	0
Peru	Lima	1990-1991	2	1990-1991	0
Peru	Trujillo	1984-2002	19	1984-2002	1
Philippines	Manila	1983-2007	25	1983-2007	0
Philippines	Rizal	1978-2007	20	1978-2007	0

© 2018 Fitzmaurice C et al. JAMA Oncology.

Location	Registry	Years available from registry	Years used for incidence	Years available for MI ratio	Years used for MI ratio
Poland	Cieszyn	1968-1977	5	1973-1977	0
Poland	Cieszyn and Nowy Sacz	1968-1972	5	1968-1972	0
Poland	Cracow	1968-2006	26	1973-2006	0
Poland	Cracow City and District	1965-1972	5	1965-1972	0
Poland	Four Rural Areas	1965-1966	0	1965-1966	0
Poland	Katowice	1965-1977	5	1965-1974	0
Poland	Kielce	1988-2007	13	1988-2007	0
Poland	Lower Silesia	1984-2007	14	1984-2007	0
Poland	National Registry	1999-2011	13	1999-2011	13
Poland	Nowy Sacz	1973-1986	13	1973-1986	0
Poland	Opole	1985-1987	3	1985-1987	0
Poland	Rzeszow	2003-2007	0	2003-2007	0
Poland	Warsaw	1988-2002	11	1988-2002	0
Poland	Warsaw Rural	1968-1987	15	1968-1987	0
Poland	Warsaw Urban	1965-2002	27	1965-2002	0
Portugal	Azores	1997-2011	14	1981-2012	15
Portugal	Centre	2003-2007	5	2003-2007	5
Portugal	North Portugal	2000-2006	7	2000-2006	7
Portugal	Porto	1998-2002	5	1998-2002	0
Portugal	South Portugal	1998-2007	10	1998-2007	0
Portugal	Vila Nova de Gaia	1983-1997	10	1983-1997	0
Puerto Rico	National Registry	NA	26	NA	NA
Qatar	National Registry	2003-2007	5	2003-2007	0
Romania	Banat Region	1967	0	1967	0
Romania	Cluj	2007	1	2007	1
Romania	County Cluj	1974-1987	14	1974-1987	0
Romania	County Timis	1970-1972	3	1970-1972	0
Romania	Timisoara	2008	1	2008	0

© 2018 Fitzmaurice C et al. JAMA Oncology.

Location	Registry	Years available from registry	Years used for incidence	Years available for MI ratio	Years used for MI ratio
Russia	St Petersburg	1983-2007	19	1983-2007	0
Samoa	National Registry	1980-1988	9	1980-1988	0
Saudi Arabia	National Registry	1994-2012	10	1994-2012	0
Saudi Arabia	Riyadh	2003-2007	5	2003-2007	0
Senegal	Dakar	1969-1974	6	1969-1974	0
Serbia	Central Serbia	2003-2007	4	2003-2007	5
Serbia	National Registry	1999-2002	4	1999-2002	0
Serbia	Vojvodina	1988-1997	10	1988-1997	0
Seychelles	National Registry	2009-2011	3	2009-2011	0
Singapore	National Registry	1950-2008	44	1950-2008	0
Slovakia	National Registry	1968-2007	42	1968-2010	30
Slovenia	National Registry	1956-2010	49	1956-2010	8
South Africa	Johannesburg, Bantu	1953-1955	0	1953-1955	0
South Africa	National Registry	2003-2011	4	2003-2011	0
South Africa	PROMEC	1998-2007	10	1998-2007	0
South Korea	Busan	1996-2007	2	1996-2007	0
South Korea	Daegu	1997-2007	2	1997-2007	0
South Korea	Daejeon	1998-2007	0	1998-2007	0
South Korea	Gwangju	1998-2007	0	1998-2007	0
South Korea	Incheon	1998-2007	0	1998-2007	0
South Korea	Jejudo	2000-2007	3	2000-2007	0
South Korea	Kangwha County	1986-1997	12	1986-1997	0
South Korea	National Registry	1999-2012	12	1999-2012	0
South Korea	Seoul	1993-2007	5	1993-2007	0
South Korea	Ulsan	1999-2007	0	1999-2007	0
Spain	Albacete	1991-2007	16	1991-2007	6
Spain	Asturias	1988-2007	17	1988-2007	5
Spain	Balears	1988-2005	18	1988-2005	0

© 2018 Fitzmaurice C et al. JAMA Oncology.

Location	Registry	Years available from registry	Years used for incidence	Years available for MI ratio	Years used for MI ratio
Spain	Basque Country	1986-2007	20	1986-2008	4
Spain	Canary Islands	1993-2006	10	1993-2006	0
Spain	Ciudad Real	2004-2007	4	2004-2007	0
Spain	Cuenca	1993-2007	15	1993-2007	5
Spain	Girona	1980-2007	27	1980-2008	23
Spain	Granada	1985-2007	22	1985-2007	23
Spain	La Rioja	1993-2007	12	1993-2007	13
Spain	Mallorca	1988-2007	14	1988-2007	0
Spain	Murcia	1983-2007	24	1983-2007	25
Spain	Navarra	1973-2007	32	1973-2008	31
Spain	Tarragona	1980-2007	25	1980-2007	24
Spain	Zaragoza	1968-2000	33	1968-2000	0
Sri Lanka	National Registry	2001-2005	6	2001-2005	0
Sweden	National Registry	1958-2014	50	1958-2014	35
Sweden	Stockholm	1990-2010	21	1990-2010	0
Sweden	Sweden except Stockholm	1990-2010	21	1990-2010	0
Switzerland	Basel	1981-2007	7	1981-2007	0
Switzerland	Geneva	1970-2008	19	1970-2008	29
Switzerland	Graubunden	1989-1997	0	1989-1997	0
Switzerland	Graubunden and Glarus	1989-2009	0	1980-2009	20
Switzerland	National Registry	1986-2013	25	1989-2013	0
Switzerland	Neuchatel	1974-2007	13	1974-2008	5
Switzerland	St Gallen - Appenzell	1980-2009	9	1980-2009	29
Switzerland	Ticino	1996-2007	0	1996-2007	0
Switzerland	Valais	1989-2007	0	1989-2007	0
Switzerland	Vaud	1975-2007	13	1975-2008	5
Switzerland	Zurich	1980-2009	9	1980-2009	0
Taiwan	National Registry	1980-2007	28	1980-2007	28

© 2018 Fitzmaurice C et al. JAMA Oncology.

Location	Registry	Years available from registry	Years used for incidence	Years available for MI ratio	Years used for MI ratio
Thailand	Bangkok	1995-2007	11	1995-2007	0
Thailand	Chiang Mai	1983-2007	29	1983-2007	0
Thailand	Chonburi	2001-2007	8	2001-2007	0
Thailand	Khon Kaen	1988-2007	19	1988-2007	0
Thailand	Lampang	1993-2007	18	1993-2007	0
Thailand	Lop Buri	2001-2003	3	2001-2003	0
Thailand	Nakhon Phanom	2001-2003	3	2001-2003	0
Thailand	Prachuap Khiri	2001-2003	3	2001-2003	0
Thailand	Rayong	2001-2003	3	2001-2003	0
Thailand	Songkhla	1993-2007	16	1993-2007	0
Thailand	Surat Thani	2001-2003	3	2001-2003	0
Thailand	Ubon Ratchathani	2001-2003	3	2001-2003	0
Thailand	Udon Thani	2001-2003	3	2001-2003	0
The Gambia	National Registry	1987-1998	15	1987-1998	0
Trinidad and Tobago	National Registry	1995-2006	12	1995-2006	12
Tunisia	Centre Sousse	1998-2002	5	1998-2002	0
Tunisia	North Tunisia	2003-2005	3	2003-2005	0
Turkey	Ankara	2002-2005	4	2002-2005	0
Turkey	Antalya	1998-2008	13	1998-2008	0
Turkey	Edirne	2002-2007	6	2002-2007	0
Turkey	Eight Provinces	2006-2007	2	2006-2007	0
Turkey	Erzurum	2002-2005	4	2002-2005	0
Turkey	Eskisehir	2002-2005	4	2002-2005	0
Turkey	Izmir	1998-2008	14	1998-2008	0
Turkey	Nine Provinces	2008	1	2008	0
Turkey	Samsun	2002-2005	4	2002-2005	0
Turkey	Trabzon	2002-2007	5	2002-2007	0

© 2018 Fitzmaurice C et al. JAMA Oncology.

Location	Registry	Years available from registry	Years used for incidence	Years available for MI ratio	Years used for MI ratio
Uganda	Kampala	1954-2013	24	1954-2013	0
Ukraine	National Registry	2000-2010	7	2000-2010	4
United Kingdom	Aryshire	1970-1972	3	1970-1972	0
United Kingdom	East Anglia	1988-1997	0	1988-1997	0
United Kingdom	East Midlands	1981-2014	34	1981-2014	20
United Kingdom	East Scotland	1973-1987	5	1973-1987	0
United Kingdom	East of England	1981-2014	33	1981-2014	20
United Kingdom	England	1993-2007	10	1993-2007	0
United Kingdom	England and Wales	1979-1990	0	1979-1990	0
United Kingdom	Greater London	1981-2014	34	1981-2014	20
United Kingdom	Merseyside and Cheshire	1959-2002	11	1959-2002	0
United Kingdom	North East England	1981-2014	34	1981-2014	20
United Kingdom	North East Scotland	1973-1987	5	1973-1987	0
United Kingdom	North Scotland	1973-1987	5	1973-1987	0
United Kingdom	North West England	1973-2014	40	1973-2014	20
United Kingdom	Northern England and Yorkshire	1998-2007	0	1998-2007	0
United Kingdom	Northern Ireland	1993-2011	18	1993-2011	0
United Kingdom	Oxford	1963-2007	18	1963-2007	0
United Kingdom	Scotland	1963-2014	40	1963-2014	25
United Kingdom	South East England	1981-2014	34	1981-2014	20
United Kingdom	South East Scotland	1973-1987	5	1973-1987	0
United Kingdom	South Thames	1960-2007	20	1960-1997	0
United Kingdom	South West England	1960-2014	37	1960-2014	20
United Kingdom	Thames	1998-2007	0	1991-2007	0
United Kingdom	Trent	1963-2007	3	1963-2007	0
United Kingdom	Wales	1981-2011	31	1981-2011	20
United Kingdom	Wessex	1988-1992	0	1988-1992	0
United Kingdom	West Midlands	1960-2014	44	1960-2014	20

© 2018 Fitzmaurice C et al. JAMA Oncology.

Location	Registry	Years available from registry	Years used for incidence	Years available for MI ratio	Years used for MI ratio
United Kingdom	West Scotland	1975-1992	0	1975-1992	0
United Kingdom	Yorkshire	1983-2002	7	1983-2002	0
United Kingdom	Yorkshire and the Humber	1981-2014	34	1981-2014	20
United States	Alabama	1998-2011	10	1998-2007	0
United States	Alameda County	1969-1987	5	1983-1987	0
United States	Alaska	1998-2011	10	1998-2007	0
United States	Alaska Natives	1992-2013	0	NA	NA
United States	Arizona	1998-2011	10	1998-2007	0
United States	Arkansas	2001-2011	5	2003-2007	0
United States	Atlanta	1973-2013	39	1973-2013	36
United States	California	1998-2011	10	1998-2007	0
United States	California except SF, SJ-M, & LA	2000-2013	14	2000-2013	9
United States	Central California	1988-1992	5	1988-1992	0
United States	Colorado	1998-2011	10	1998-2007	0
United States	Connecticut	1960-2013	41	1960-2013	37
United States	Delaware	1999-2011	5	2003-2007	0
United States	Detroit	1969-2013	41	1973-2013	37
United States	District of Columbia	1998-2011	5	1998-2002	0
United States	El Paso	1960-1970	0	1960-1970	0
United States	Florida	1998-2011	10	1998-2007	0
United States	Georgia	1998-2011	10	1998-2007	0
United States	Greater Georgia	1973-2013	14	1973-2013	13
United States	Hawaii	1960-2013	46	1960-2013	37
United States	Idaho	1998-2011	10	1998-2007	0
United States	Illinois	1998-2011	10	1998-2007	0
United States	Indiana	1998-2011	10	1998-2007	0
United States	Iowa	1969-2013	41	1973-2013	37
United States	Kansas	1999-2011	0	NA	NA

© 2018 Fitzmaurice C et al. JAMA Oncology.

Location	Registry	Years available from registry	Years used for incidence	Years available for MI ratio	Years used for MI ratio
United States	Kentucky	1973-2013	19	1973-2013	19
United States	Los Angeles	1973-2013	41	1973-2013	24
United States	Louisiana	1973-2013	29	1973-2013	18
United States	Maine	1998-2011	10	1998-2007	0
United States	Maryland	1999-2011	0	NA	NA
United States	Massachusetts	1998-2011	10	1998-2007	0
United States	Michigan	1998-2011	10	1998-2007	0
United States	Minnesota	1999-2011	0	NA	NA
United States	Mississippi	2003-2011	5	2003-2007	0
United States	Missouri	1998-2011	10	1998-2007	0
United States	Montana	1998-2011	10	1998-2007	0
United States	National Registry	1962-2007	0	1962-2007	0
United States	Nebraska	1999-2011	5	2003-2007	0
United States	Nevada	1959-2010	0	1959-1966	0
United States	New Hampshire	1999-2011	5	2003-2007	0
United States	New Jersey	1973-2013	24	1973-2013	19
United States	New Mexico	1969-2013	45	1969-2013	37
United States	New Orleans	1974-2007	17	1983-2007	0
United States	New York	1993-2011	15	1993-2007	0
United States	New York City	1978-1987	0	NA	NA
United States	New York except New York City	1959-1987	0	1959-1961	0
United States	North Carolina	1999-2011	5	2003-2007	0
United States	North Dakota	1999-2011	5	2003-2007	0
United States	Ohio	1998-2011	10	1998-2007	0
United States	Oklahoma	1998-2011	10	1998-2007	0
United States	Oregon	1998-2011	10	1998-2007	0
United States	Pennsylvania	1998-2011	10	1998-2007	0
United States	Rhode Island	1998-2011	10	1998-2007	0

© 2018 Fitzmaurice C et al. JAMA Oncology.

Location	Registry	Years available from registry	Years used for incidence	Years available for MI ratio	Years used for MI ratio
United States	Rural Georgia	1973-2013	22	1973-2013	24
United States	San Francisco	1969-2007	29	1973-2007	0
United States	San Francisco, Oakland, San Mateo, and Surrounding Area	1973-2013	41	1973-2013	36
United States	San Jose Monterey	1973-2013	22	1973-2013	24
United States	Seattle	1973-2013	40	1973-2013	36
United States	South Carolina	1998-2011	10	1998-2007	0
United States	South Dakota	2001-2011	5	2003-2007	0
United States	Tennessee	2003-2011	5	2003-2007	0
United States	Texas	1998-2011	10	1998-2007	0
United States	Utah	1966-2013	41	1973-2013	37
United States	Vermont	1998-2011	10	1998-2007	0
United States	Virginia	2003-2011	5	2003-2007	0
United States	Washington	1998-2011	10	1998-2007	0
United States	West Virginia	1998-2011	10	1998-2007	0
United States	Wisconsin	1998-2011	10	1998-2007	0
United States	Wyoming	1999-2011	5	2003-2007	0
Uruguay	Montevideo	1990-1995	6	1990-1995	0
Uruguay	National Registry	2002-2007	8	2002-2007	0
Vietnam	Hanoi	1991-1997	8	1991-1997	0
Vietnam	Ho Chi Minh	1995-1998	4	1995-1998	0
Zimbabwe	Bulawayo	1963-1972	5	1963-1972	0
Zimbabwe	Harare	1990-2006	17	1990-2006	0
Zimbabwe	National Registry	2005-2006	2	2005-2006	0

© 2018 Fitzmaurice C et al. JAMA Oncology.

eTable 3: Number of site-years for cancer mortality data by type

Cause	VR GBD 2015	VR GBD 2016	VR change GBD 2015 to GBD 2016	VA GBD 2015	VA GBD 2016	VA change GBD 2015 to GBD 2016	CR GBD 2015	CR GBD 2016	CR change GBD 2015 to GBD 2016	Total GBD 2015	Total GBD 2016	Total change GBD 2015 to GBD 2016
Lip and oral cavity cancer	10049	15962	59%	134	585	337%	2399	2758	15%	12582	19305	53%
Nasopharynx cancer	10070	15988	59%				2354	2806	19%	12424	18794	51%
Other pharynx cancer	10050	15967	59%		374		2323	2782	20%	12373	19123	55%
Esophageal cancer	10408	16298	57%	132	590	347%	2411	2849	18%	12951	19737	52%
Stomach cancer	10414	16304	57%		374		2432	2873	18%	12846	19551	52%
Colon and rectum cancer	10413	16303	57%	134	602	349%	2432	2873	18%	12979	19778	52%
Liver cancer	10118	16028	58%		374		2419	2859	18%	12537	19261	54%
Gallbladder and biliary tract cancer	10090	14972	48%				2331	2779	19%	12421	17751	43%
Pancreatic cancer	9796	15701	60%				2418	2862	18%	12214	18563	52%
Larynx cancer	10390	16280	57%		374		2405	2846	18%	12795	19500	52%
Tracheal, bronchus, and lung cancer	10413	16303	57%	147	602	310%	2413	2853	18%	12973	19758	52%
Malignant skin melanoma	10103	16018	59%				2305	2704	17%	12408	18722	51%
Non-melanoma skin cancer	9680	15581	61%				1230		-100%	10910	15581	43%
Non-melanoma skin cancer (squamous-cell carcinoma)	8526	14204	67%				1230		-100%	9756	14204	46%
Breast cancer	10403	16294	57%	153	618	304%	2417	2861	18%	12973	19773	52%

© 2018 Fitzmaurice C et al. JAMA Oncology.

Cause	VR GBD 2015	VR GBD 2016	VR change GBD 2015 to GBD 2016	VA GBD 2015	VA GBD 2016	VA change GBD 2015 to GBD 2016	CR GBD 2015	CR GBD 2016	CR change GBD 2015 to GBD 2016	Total GBD 2015	Total GBD 2016	Total change GBD 2015 to GBD 2016
Cervical cancer	10406	16295	57%		370		2382	2809	18%	12788	19474	52%
Uterine cancer	10325	16280	58%		374		2373	2831	19%	12698	19485	53%
Ovarian cancer	9802	15710	60%				2371	2845	20%	12173	18555	52%
Prostate cancer	10356	16247	57%				2351	2826	20%	12707	19073	50%
Testicular cancer	8869	14549	64%	84	130	55%	2274	2734	20%	11227	17413	55%
Kidney cancer	10095	16010	59%				2387	2716	14%	12482	18726	50%
Bladder cancer	10100	16010	59%				2426	2656	9%	12526	18666	49%
Brain and nervous system cancer	9801	15705	60%	1	378	37700%	2418	2885	19%	12220	18968	55%
Thyroid cancer	9713	15634	61%				2375	2826	19%	12088	18460	53%
Mesothelioma	9095	7418	-18%				1293		-100%	10388	7418	-29%
Hodgkin lymphoma	10078	15991	59%				2289	2762	21%	12367	18753	52%
Non-Hodgkin lymphoma	10102	16011	58%				2394	2860	19%	12496	18871	51%
Multiple myeloma	10086	16005	59%				2294	2737	19%	12380	18742	51%
Leukemia	10964	16851	54%		585		2396	2925	22%	13360	20361	52%
Acute lymphoid leukemia	8236	13930	69%				260	1130	335%	8496	15060	77%
Chronic lymphoid leukemia	8226	13926	69%				249	1104	343%	8475	15030	77%
Acute myeloid leukemia	8235	13930	69%				259	1118	332%	8494	15048	77%
Chronic myeloid leukemia	8234	13930	69%				259	1124	334%	8493	15054	77%
Other leukemia		15986			211			1913			18110	
Other neoplasms	10486	16149	54%				2444	2951	21%	12930	19100	48%

VR: vital registration system data, VA: verbal autopsy data, CR: cancer registry data

© 2018 Fitzmaurice C et al. JAMA Oncology.

eTable 4: List of International Classification of Diseases (ICD) codes mapped to the Global Burden of Disease cause list for cancer incidence data

Cause	ICD10	ICD9
Lip and oral cavity cancer	C00-C07, C08-C08.9, Z85.81-Z85.810	140-145.9, V76.42
Nasopharynx cancer	C11-C11.9	147-147.9
Other pharynx cancer	C09-C10.9, C12-C13.9	146-146.9, 148-148.9
Esophageal cancer	C15-C15.9, Z85.01	150-150.9
Stomach cancer	C16-C16.9, Z12.0, Z85.02-Z85.028	151-151.9, 209.23, V10.04
Colon and rectum cancer	C18-C19.0, C20, C21-C21.8, Z12.1-Z12.13, Z85.03-Z85.048, Z86.010	153-154.9, 209.1-209.17, V10.05-V10.06, V76.41, V76.5-V76.52
Liver cancer	C22-C22.4, C22.7-C22.9, Z85.05	155-155.9, V10.07
Liver cancer due to hepatitis B		
Liver cancer due to hepatitis C		
Liver cancer due to alcohol use		
Liver cancer due to other causes		
Gallbladder and biliary tract cancer	C23, C24-C24.9	156-156.9
Pancreatic cancer	C25-C25.9, Z85.07	157-157.9
Larynx cancer	C32-C32.9, Z85.21	161-161.9, V10.21
Tracheal, bronchus, and lung cancer	C33, C34-C34.92, Z12.2, Z80.1-Z80.2, Z85.1-Z85.20	162-162.9, 209.21, V10.1-V10.20, V16.1-V16.2, V16.4-V16.40
Malignant skin melanoma	C43-C43.9, Z85.82-Z85.828	172-172.9
Non-melanoma skin cancer	C44-C44.99	173-173.99, 216-216.9, 232-232.9
Non-melanoma skin cancer (squamous-cell carcinoma)	C44.02, C44.12-C44.129, C44.22-C44.229, C44.32-C44.329, C44.42, C44.52-C44.529, C44.62-C44.629, C44.72-C44.729, C44.82, C44.92	173.02, 173.12, 173.22, 173.32, 173.42, 173.52, 173.62, 173.72, 173.82, 173.92
Non-melanoma skin cancer (basal-cell carcinoma)	C44.01, C44.11-C44.119, C44.21-C44.219, C44.31-C44.319, C44.41, C44.51-C44.519, C44.61-C44.619, C44.71-C44.719, C44.81, C44.91	173.01, 173.11, 173.21, 173.31, 173.41, 173.51, 173.60-173.61, 173.71, 173.81, 173.91

© 2018 Fitzmaurice C et al. JAMA Oncology.

Cause	ICD10	ICD9
Breast cancer	C50-C50.629, C50.8-C50.929, Z12.3-Z12.39, Z80.3, Z85.3, Z86.000	174-175.9, V10.3, V16.3
Cervical cancer	C53-C53.9, Z12.4, Z85.41	180-180.9, V10.41, V72.32
Uterine cancer	C54-C54.3, C54.8-C54.9, Z85.42, Z86.001	182-182.9
Ovarian cancer	C56-C56.2, C56.9, Z80.41, Z85.43	183-183.0, 183.8-183.9, V10.43, V16.41
Prostate cancer	C61-C61.9, Z12.5, Z80.42, Z85.46	185-185.9, V10.46, V16.42, V76.44
Testicular cancer	C62-C62.92, Z80.43, Z85.47-Z85.48	186-186.9, V10.47-V10.48, V16.43
Kidney cancer	C64-C64.2, C64.9-C65.9, Z80.51, Z85.52-Z85.54	189-189.1, 189.5-189.6, 209.24
Bladder cancer	C67-C67.9, Z12.6-Z12.79, Z80.52, Z85.51	188-188.9, V10.51, V16.52, V76.3
Brain and nervous system cancer	C70-C70.1, C70.9-C72.9, Z85.841-Z85.848, Z86.011	191-191.9
Thyroid cancer	C73, Z85.850	193-193.9
Mesothelioma	C45-C45.2, C45.7, C45.9	
Hodgkin lymphoma	C81-C81.49, C81.7-C81.79, C81.9-C81.99, Z85.71-Z85.72	201-201.98, V10.72
Non-Hodgkin lymphoma	C82-C85.29, C85.7-C86.6, C96-C96.9	200-200.9, 202-202.98
Multiple myeloma	C88-C90.32	203-203.9
Leukemia	C91-C93.7, C93.9-C95.2, C95.7-C95.92, Z80.6, Z85.6	204-208.92, V10.59-V10.69, V16.6
Acute lymphoid leukemia	C91.0-C91.02	204.0-204.02
Chronic lymphoid leukemia	C91.1-C91.12	204.1-204.12
Acute myeloid leukemia	C92.0-C92.02, C92.3-C92.62, C93.0-C93.02, C94.0-C94.02, C94.2-C94.22, C94.4-C94.5	205.0-205.02, 205.3-205.32, 206.0-206.02, 207.0
Chronic myeloid leukemia	C92.1-C92.12	205.1-205.12, 206.1-206.12, 207.1
Other leukemia	C91.2-C91.9, C92.2, C92.7-C92.9, C93.1-C93.9, C94.1, C94.3, C94.6-C95.9	204.2-204.9, 205.2, 205.8-205.9, 206.2-207, 207.2-208.9

© 2018 Fitzmaurice C et al. JAMA Oncology.

Cause	ICD10	ICD9
Other neoplasms	C17-C17.9, C30-C30.1, C31-C31.9, C37-C37.0, C38-C38.8, C40-C41.4, C41.8-C41.9, C47-C4A, C51-C52, C57-C57.8, C58-C58.0, C60-C60.9, C63-C63.8, C66-C66.9, C68.0-C68.8, C69-C69.92, C74-C75.5, C75.8	152-152.9, 158-158.9, 160-160.9, 163-164.9, 170-171.9, 181-181.9, 183.2-183.5, 184-184.9, 187-187.9, 189.2-189.4, 189.8-190.9, 192-192.9, 194-194.8, 209-209.03, 209.22, 209.25-209.27, 209.31-209.36

eTable 5: List of International Classification of Diseases (ICD) codes mapped to the Global Burden of Disease cause list for cancer mortality data

Cause	ICD10	ICD9
Lip and oral cavity cancer	C00-C08.9, D10.0-D10.5, D11-D11.9	140-145.9, 210.0-210.6, 235.0
Nasopharynx cancer	C11-C11.9, D10.6	147-147.9, 210.7-210.9
Other pharynx cancer	C09-C10.9, C12-C13.9, D10.7	146-146.9, 148-148.9
Esophageal cancer	C15-C15.9, D00.1, D13.0	150-150.9, 211.0, 230.1
Stomach cancer	C16-C16.9, D00.2, D13.1, D37.1	151-151.9, 211.1, 230.2
Colon and rectum cancer	C18-C21.9, D01.0-D01.3, D12-D12.9, D37.3-D37.5	153-154.9, 209.1, 209.5, 211.3-211.4, 230.3-230.6
Liver cancer	C22-C22.9, D13.4	155-155.9, 211.5
Liver cancer due to hepatitis B		
Liver cancer due to hepatitis C		
Liver cancer due to alcohol use		
Liver cancer due to other causes		
Gallbladder and biliary tract cancer	C23-C24.9, D13.5	156-156.9
Pancreatic cancer	C25-C25.9, D13.6-D13.7	157-157.9, 211.6-211.7
Larynx cancer	C32-C32.9, D02.0, D14.1, D38.0	161-161.9, 212.1, 231.0, 235.6
Tracheal, bronchus, and lung cancer	C33-C34.9, D02.1-D02.3, D14.2-D14.3, D38.1	162-162.9, 212.2-212.3, 231.1-231.2, 235.7
Malignant skin melanoma	C43-C43.9, D03-D03.9, D22-D23.9, D48.5	172-172.9

© 2018 Fitzmaurice C et al. JAMA Oncology.

Cause	ICD10	ICD9
Non-melanoma skin cancer	C44-C44.9, D04-D04.9, D49.2	173-173.9, 222.4, 232-232.9, 238.2
Non-melanoma skin cancer (squamous-cell carcinoma)	C44-C44.9, D04-D04.9, D49.2	173-173.9, 222.4, 232-232.9, 238.2
Breast cancer	C50-C50.9, D05-D05.9, D24-D24.9, D48.6, D49.3, N60-N60.9	174-175.9, 217-217.8, 233.0, 238.3, 239.3, 610-610.9
Cervical cancer	C53-C53.9, D06-D06.9, D26.0	180-180.9, 219.0, 233.1
Uterine cancer	C54-C54.9, D07.0-D07.2, N87-N87.9	182-182.8, 233.2
Ovarian cancer	C56-C56.9, D27-D27.9, D39.1	183-183.0, 220-220.9, 236.2
Prostate cancer	C61-C61.9, D07.5, D29.1, D40.0	185-185.9, 222.2, 236.5
Testicular cancer	C62-C62.9, D29.2-D29.8, D40.1-D40.8	186-186.9, 222.0, 222.3, 236.4
Kidney cancer	C64-C65.9, D30.0-D30.1, D41.0-D41.1	189.0-189.1, 189.5-189.6, 223.0-223.1
Bladder cancer	C67-C67.9, D09.0, D30.3, D41.4-D41.8, D49.4	188-188.9, 223.3, 233.7, 236.7, 239.4
Brain and nervous system cancer	C70-C72.9	191-192.9
Thyroid cancer	C73-C73.9, D09.3, D09.8, D34-D34.9, D44.0	193-193.9, 226-226.9
Mesothelioma	C45-C45.9	
Hodgkin lymphoma	C81-C81.9	201-201.9
Non-Hodgkin lymphoma	C82-C86.6, C96-C96.9	200-200.9, 202-202.9
Multiple myeloma	C88-C90.9	203-203.9
Leukemia	C91-C95.9	204-208.9
Acute lymphoid leukemia	C91.0	204.0
Chronic lymphoid leukemia	C91.1	204.1
Acute myeloid leukemia	C92.0, C92.3-C92.6, C93.0, C94.0, C94.2, C94.4-C94.5	205.0, 205.3, 206.0, 207.0
Chronic myeloid leukemia	C92.1	205.1, 206.1, 207.1
Other leukemia	C91.2-C91.9, C92.2, C92.7-C92.9, C93.1-C93.9, C94.1, C94.3, C94.6-C95.9	204.2-204.9, 205.2, 205.8-205.9, 206.2-207, 207.2-208.9

© 2018 Fitzmaurice C et al. JAMA Oncology.

Cause	ICD10	ICD9
Other neoplasms	C17-C17.9, C30-C31.9, C37-C38.8, C40-C41.9, C47-C4A, C51-C52.9, C57-C57.8, C58-C58.0, C60-C60.9, C63-C63.8, C66-C66.9, C68.0-C68.8, C69-C69.9, C74-C75.8, D07.4, D09.2, D13.2-D13.3, D14.0, D15-D16.9, D28.0-D28.1, D28.7, D29.0, D30.2, D30.4-D30.8, D31-D33.9, D35-D36, D36.1-D36.7, D37.2, D38.2-D38.5, D39.2, D39.8, D41.2-D41.3, D42-D43.9, D44.1-D44.8, D45-D47.0, D47.2-D47.9, D48.0-D48.4, D49.6, K31.7, K62.0-K62.1, K63.5, N84.0-N84.1	152-152.9, 158-158.9, 160-160.9, 163-164.9, 170-171.9, 181-181.9, 182.9, 183.2-183.8, 184.0-184.4, 184.8, 187.1-187.8, 189.2-189.4, 189.8, 190-190.9, 194-194.8, 209.0, 209.4, 211.2, 211.8, 212.0, 212.4-212.8, 213-213.9, 221.0-221.8, 222.1, 222.8, 223.2, 223.8, 224-225.9, 227-228.9, 229.0, 229.8, 230.7-230.8, 233.4-233.5, 234.0-234.8, 235.4, 235.8, 236.1, 237-237.3, 237.5-237.9, 238.0-238.1, 238.4-238.9, 239.2, 239.6, 569.0
ICD codes specifying benign neoplasms in the raw mortality data from vital registration systems were mapped to the respective malignant cause.		

© 2018 Fitzmaurice C et al. JAMA Oncology.

eTable 6: Undefined cancer code categories (ICD-10) and respective target codes for cancer registry incidence data

Unspecified site cancer codes	Target codes for redistribution of these unspecified site cancer
C14,C14.0-C14.3,C14.8	C00-C13.99
C26,C26.0,C26.1,C26.8,C26.9	C15.00-C25.99
C39,C39.0,C39.8,C39.9	C30.00-C38.99, C45.00-C45.99
C55,C55.1,C55.9	C53.00-C54.99
C57.9	C51.00-C54.99, C56.00-C58.99
C68.9	C64.00-C68.89
C63.9	C60.00-C63.89
C75.9	C73.00-C75.89
C76,C76.4,C76.5,C76.8,C77,C77.3-C77.5,C77.8,C77.9,C78,C79,C79.2-C79.9,C80,C80.0,C80.2	C00-C99 (Except any unspecified site cancer codes)
C76.0,C76.1,C77.0,C77.1,C78.0-C78.3	C00-C13.99 ,C15, C30-C34.99,C37-C38.99, C40-C42.99, C43-C50.99, C69-C73.9
C76.2,C76.3,C77.2,C7.5,C78.4-C78.8,C79.0,C79.1	C15.00-C25.99, C45.00-C45.99, C48.00-C54.99, C56.00-C58.99 , C61, C63.00-C63.89, C64.00-C68.99, C74.00-C75.89, C81.00-C88.99

eTable 7: Sociodemographic Index groupings by geography, based on 2016 values

Location	SDI quintile
Andorra	High SDI
Australia	High SDI
Austria	High SDI
Belgium	High SDI
Brunei	High SDI
Canada	High SDI
Croatia	High SDI
Cyprus	High SDI
Czech Republic	High SDI
Denmark	High SDI
Estonia	High SDI
Finland	High SDI
France	High SDI
Georgia	High SDI
Germany	High SDI
Greece	High SDI

© 2018 Fitzmaurice C et al. JAMA Oncology.

Iceland	High SDI
Ireland	High SDI
Italy	High SDI
Japan	High SDI
Latvia	High SDI
Lithuania	High SDI
Luxembourg	High SDI
Malta	High SDI
Netherlands	High SDI
New Zealand	High SDI
Norway	High SDI
Poland	High SDI
Puerto Rico	High SDI
Singapore	High SDI
Slovakia	High SDI
Slovenia	High SDI
South Korea	High SDI
Sweden	High SDI
Switzerland	High SDI
Taiwan	High SDI
United Kingdom	High SDI
United States	High SDI
Virgin Islands, U.S.	High SDI
Antigua and Barbuda	High-middle SDI
Argentina	High-middle SDI
Armenia	High-middle SDI
Azerbaijan	High-middle SDI
Barbados	High-middle SDI
Belarus	High-middle SDI
Bermuda	High-middle SDI
Bulgaria	High-middle SDI
Chile	High-middle SDI
Cuba	High-middle SDI
Georgia	High-middle SDI
Greenland	High-middle SDI
Guam	High-middle SDI
Hungary	High-middle SDI
Iran	High-middle SDI
Israel	High-middle SDI
Kazakhstan	High-middle SDI
Kuwait	High-middle SDI

© 2018 Fitzmaurice C et al. JAMA Oncology.

Lebanon	High-middle SDI
Libya	High-middle SDI
Macedonia	High-middle SDI
Malaysia	High-middle SDI
Mauritius	High-middle SDI
Montenegro	High-middle SDI
Northern Mariana Islands	High-middle SDI
Panama	High-middle SDI
Portugal	High-middle SDI
Qatar	High-middle SDI
Romania	High-middle SDI
Russia	High-middle SDI
Saudi Arabia	High-middle SDI
Serbia	High-middle SDI
Spain	High-middle SDI
The Bahamas	High-middle SDI
Trinidad and Tobago	High-middle SDI
Turkey	High-middle SDI
Turkmenistan	High-middle SDI
Ukraine	High-middle SDI
United Arab Emirates	High-middle SDI
Albania	Middle SDI
Algeria	Middle SDI
American Samoa	Middle SDI
Bahrain	Middle SDI
Bosnia and Herzegovina	Middle SDI
Botswana	Middle SDI
Brazil	Middle SDI
China	Middle SDI
Colombia	Middle SDI
Costa Rica	Middle SDI
Dominica	Middle SDI
Dominican Republic	Middle SDI
Ecuador	Middle SDI
Egypt	Middle SDI
El Salvador	Middle SDI
Equatorial Guinea	Middle SDI

© 2018 Fitzmaurice C et al. JAMA Oncology.

Fiji	Middle SDI
Grenada	Middle SDI
Guyana	Middle SDI
Indonesia	Middle SDI
Jamaica	Middle SDI
Jordan	Middle SDI
Maldives	Middle SDI
Mexico	Middle SDI
Moldova	Middle SDI
Mongolia	Middle SDI
Oman	Middle SDI
Paraguay	Middle SDI
Peru	Middle SDI
Philippines	Middle SDI
Saint Lucia	Middle SDI
Saint Vincent and the Grenadines	Middle SDI
Seychelles	Middle SDI
South Africa	Middle SDI
Sri Lanka	Middle SDI
Suriname	Middle SDI
Thailand	Middle SDI
Tunisia	Middle SDI
Uruguay	Middle SDI
Uzbekistan	Middle SDI
Venezuela	Middle SDI
Vietnam	Middle SDI
Bangladesh	Low-middle SDI
Belize	Low-middle SDI
Bhutan	Low-middle SDI
Bolivia	Low-middle SDI
Cambodia	Low-middle SDI
Cameroon	Low-middle SDI
Cape Verde	Low-middle SDI
Congo	Low-middle SDI
Federated States of Micronesia	Low-middle SDI
Gabon	Low-middle SDI
Ghana	Low-middle SDI
Guatemala	Low-middle SDI
Honduras	Low-middle SDI
India	Low-middle SDI

© 2018 Fitzmaurice C et al. JAMA Oncology.

Iraq	Low-middle SDI
Kenya	Low-middle SDI
Kyrgyzstan	Low-middle SDI
Laos	Low-middle SDI
Lesotho	Low-middle SDI
Marshall Islands	Low-middle SDI
Mauritania	Low-middle SDI
Morocco	Low-middle SDI
Myanmar	Low-middle SDI
Namibia	Low-middle SDI
Nepal	Low-middle SDI
Nicaragua	Low-middle SDI
Nigeria	Low-middle SDI
North Korea	Low-middle SDI
Pakistan	Low-middle SDI
Samoa	Low-middle SDI
Sudan	Low-middle SDI
Swaziland	Low-middle SDI
Syria	Low-middle SDI
Tajikistan	Low-middle SDI
Timor-Leste	Low-middle SDI
Tonga	Low-middle SDI
Vanuatu	Low-middle SDI
Zambia	Low-middle SDI
Zimbabwe	Low-middle SDI
Afghanistan	Low SDI
Angola	Low SDI
Benin	Low SDI
Burkina Faso	Low SDI
Burundi	Low SDI
Central African Republic	Low SDI
Chad	Low SDI
Comoros	Low SDI
Cote d'Ivoire	Low SDI
Democratic Republic of the Congo	Low SDI
Djibouti	Low SDI
Eritrea	Low SDI
Ethiopia	Low SDI
Guinea	Low SDI

© 2018 Fitzmaurice C et al. JAMA Oncology.

Guinea-Bissau	Low SDI
Haiti	Low SDI
Kiribati	Low SDI
Liberia	Low SDI
Madagascar	Low SDI
Malawi	Low SDI
Mali	Low SDI
Mozambique	Low SDI
Niger	Low SDI
Palestine	Low SDI
Papua New Guinea	Low SDI
Rwanda	Low SDI
Sao Tome and Principe	Low SDI
Senegal	Low SDI
Sierra Leone	Low SDI
Solomon Islands	Low SDI
Somalia	Low SDI
South Sudan	Low SDI
Tanzania	Low SDI
The Gambia	Low SDI
Togo	Low SDI
Uganda	Low SDI
Yemen	Low SDI

© 2018 Fitzmaurice C et al. JAMA Oncology.

eTable 8: Covariates selected for CODEm for each GBD cancer group and expected direction of covariate

Cause	Sex	Age start	Age end	Direction	Covariate
Esophageal cancer	Male	15-19 years	95+ years	1	Tobacco (cigarettes per capita)
Esophageal cancer	Male	15-19 years	95+ years	-1	Education (years per capita)
Esophageal cancer	Male	15-19 years	95+ years	-1	Fruits (kcal per capita)
Esophageal cancer	Male	15-19 years	95+ years	-1	LDI (I$ per capita)
Esophageal cancer	Male	15-19 years	95+ years	1	Mean BMI
Esophageal cancer	Male	15-19 years	95+ years	1	Indoor Air Pollution (All Cooking Fuels)
Esophageal cancer	Male	15-19 years	95+ years	-1	Sanitation (proportion with access)
Esophageal cancer	Male	15-19 years	95+ years	1	Smoking Prevalence
Esophageal cancer	Male	15-19 years	95+ years	-1	Vegetables (kcal per capita)
Esophageal cancer	Male	15-19 years	95+ years	-1	Improved Water Source (proportion with access)
Esophageal cancer	Male	15-19 years	95+ years	1	Log-transformed age-standardized SEV scalar: Esophag C
Esophageal cancer	Male	15-19 years	95+ years	-1	Socio-demographic Index
Esophageal cancer	Male	15-19 years	95+ years	-1	Healthcare access and quality index
Esophageal cancer	Female	15-19 years	95+ years	1	Alcohol (liters per capita)
Esophageal cancer	Female	15-19 years	95+ years	1	Tobacco (cigarettes per capita)
Esophageal cancer	Female	15-19 years	95+ years	-1	Education (years per capita)
Esophageal cancer	Female	15-19 years	95+ years	-1	Fruits (kcal per capita)
Esophageal cancer	Female	15-19 years	95+ years	-1	LDI (I$ per capita)
Esophageal cancer	Female	15-19 years	95+ years	1	Mean BMI
Esophageal cancer	Female	15-19 years	95+ years	1	Indoor Air Pollution (All Cooking Fuels)
Esophageal cancer	Female	15-19 years	95+ years	-1	Sanitation (proportion with access)
Esophageal cancer	Female	15-19 years	95+ years	1	Smoking Prevalence
Esophageal cancer	Female	15-19 years	95+ years	-1	Vegetables (kcal per capita)
Esophageal cancer	Female	15-19 years	95+ years	-1	Improved Water Source (proportion with access)
Esophageal cancer	Female	15-19 years	95+ years	1	Log-transformed age-standardized SEV scalar: Esophag C
Esophageal cancer	Female	15-19 years	95+ years	0	Socio-demographic Index
Esophageal cancer	Female	15-19 years	95+ years	-1	Healthcare access and quality index
Esophageal cancer	Female	15-19 years	95+ years	0	LDI (I$ per capita)
Esophageal cancer	Female	15-19 years	95+ years	-1	Socio-demographic Index

© 2018 Fitzmaurice C et al. JAMA Oncology.

Cause	Sex	Age start	Age end	Direction	Covariate
Esophageal cancer	Female	15-19 years	95+ years	-1	fruits adjusted(g)
Esophageal cancer	Female	15-19 years	95+ years	-1	vegetables adjusted(g)
Esophageal cancer	Male	15-19 years	95+ years	0	LDI (I$ per capita)
Esophageal cancer	Male	15-19 years	95+ years	1	Log-transformed SEV scalar: Esophag C
Esophageal cancer	Male	15-19 years	95+ years	-1	fruits adjusted(g)
Esophageal cancer	Male	15-19 years	95+ years	-1	vegetables adjusted(g)
Stomach cancer	Male	15-19 years	95+ years	1	Alcohol (liters per capita)
Stomach cancer	Male	15-19 years	95+ years	1	Tobacco (cigarettes per capita)
Stomach cancer	Male	15-19 years	95+ years	1	Cumulative Cigarettes (10 Years)
Stomach cancer	Male	15-19 years	95+ years	1	Cumulative Cigarettes (15 Years)
Stomach cancer	Male	15-19 years	95+ years	0	LDI (I$ per capita)
Stomach cancer	Male	15-19 years	95+ years	1	Mean BMI
Stomach cancer	Male	15-19 years	95+ years	1	Indoor Air Pollution (All Cooking Fuels)
Stomach cancer	Male	15-19 years	95+ years	1	Outdoor Air Pollution (PM2.5)
Stomach cancer	Male	15-19 years	95+ years	-1	Sanitation (proportion with access)
Stomach cancer	Male	15-19 years	95+ years	1	Smoking Prevalence
Stomach cancer	Male	15-19 years	95+ years	-1	Improved Water Source (proportion with access)
Stomach cancer	Male	15-19 years	95+ years	1	Log-transformed SEV scalar: Stomach C
Stomach cancer	Male	15-19 years	95+ years	0	Socio-demographic Index
Stomach cancer	Male	15-19 years	95+ years	-1	fruits adjusted (g)
Stomach cancer	Male	15-19 years	95+ years	-1	vegetables adjusted (g)
Stomach cancer	Male	15-19 years	95+ years	-1	Healthcare access and quality index
Stomach cancer	Male	15-19 years	95+ years	1	Diet high in sodium
Stomach cancer	Female	15-19 years	95+ years	1	Alcohol (liters per capita)
Stomach cancer	Female	15-19 years	95+ years	1	Tobacco (cigarettes per capita)
Stomach cancer	Female	15-19 years	95+ years	1	Cumulative Cigarettes (10 Years)
Stomach cancer	Female	15-19 years	95+ years	-1	Education (years per capita)
Stomach cancer	Female	15-19 years	95+ years	0	LDI (I$ per capita)
Stomach cancer	Female	15-19 years	95+ years	1	Mean BMI
Stomach cancer	Female	15-19 years	95+ years	1	Indoor Air Pollution (All Cooking Fuels)

© 2018 Fitzmaurice C et al. JAMA Oncology.

Cause	Sex	Age start	Age end	Direction	Covariate
Stomach cancer	Female	15-19 years	95+ years	1	Outdoor Air Pollution (PM2.5)
Stomach cancer	Female	15-19 years	95+ years	-1	Sanitation (proportion with access)
Stomach cancer	Female	15-19 years	95+ years	1	Smoking Prevalence
Stomach cancer	Female	15-19 years	95+ years	-1	Improved Water Source (proportion with access)
Stomach cancer	Female	15-19 years	95+ years	1	Log-transformed SEV scalar: Stomach C
Stomach cancer	Female	15-19 years	95+ years	0	Socio-demographic Index
Stomach cancer	Female	15-19 years	95+ years	-1	fruits adjusted (g)
Stomach cancer	Female	15-19 years	95+ years	-1	vegetables adjusted (g)
Stomach cancer	Female	15-19 years	95+ years	-1	Healthcare access and quality index
Stomach cancer	Female	15-19 years	95+ years	1	Diet high in sodium
Liver cancer	Male	5-9 years	95+ years	1	Alcohol (liters per capita)
Liver cancer	Male	5-9 years	95+ years	1	Tobacco (cigarettes per capita)
Liver cancer	Male	5-9 years	95+ years	1	Cumulative Cigarettes (15 Years)
Liver cancer	Male	5-9 years	95+ years	1	Cumulative Cigarettes (20 Years)
Liver cancer	Male	5-9 years	95+ years	1	Diabetes Age-Standardized Prevalence (proportion)
Liver cancer	Male	5-9 years	95+ years	-1	Education (years per capita)
Liver cancer	Male	5-9 years	95+ years	0	LDI (I$ per capita)
Liver cancer	Male	5-9 years	95+ years	1	Mean BMI
Liver cancer	Male	5-9 years	95+ years	1	Percent of total calories consumed as saturated fat
Liver cancer	Male	5-9 years	95+ years	1	Log-transformed SEV scalar: Liver C
Liver cancer	Male	5-9 years	95+ years	0	Socio-demographic Index
Liver cancer	Male	5-9 years	95+ years	1	red meats adjusted (g)
Liver cancer	Male	5-9 years	95+ years	1	Hepatitis B (HBsAg) Seroprevalence
Liver cancer	Male	5-9 years	95+ years	1	Hepatitis C (IgG) Seroprevalence
Liver cancer	Male	5-9 years	95+ years	-1	Healthcare access and quality index
Liver cancer	Female	5-9 years	95+ years	1	Alcohol (liters per capita)
Liver cancer	Female	5-9 years	95+ years	1	Tobacco (cigarettes per capita)
Liver cancer	Female	5-9 years	95+ years	1	Cumulative Cigarettes (15 Years)
Liver cancer	Female	5-9 years	95+ years	1	Cumulative Cigarettes (20 Years)
Liver cancer	Female	5-9 years	95+ years	1	Diabetes Age-Standardized Prevalence (proportion)

© 2018 Fitzmaurice C et al. JAMA Oncology.

Cause	Sex	Age start	Age end	Direction	Covariate
Liver cancer	Female	5-9 years	95+ years	-1	Education (years per capita)
Liver cancer	Female	5-9 years	95+ years	0	LDI (I$ per capita)
Liver cancer	Female	5-9 years	95+ years	1	Mean BMI
Liver cancer	Female	5-9 years	95+ years	1	Percent of total calories consumed as saturated fat
Liver cancer	Female	5-9 years	95+ years	1	Log-transformed SEV scalar: Liver C
Liver cancer	Female	5-9 years	95+ years	0	Socio-demographic Index
Liver cancer	Female	5-9 years	95+ years	1	red meats adjusted (g)
Liver cancer	Female	5-9 years	95+ years	1	Hepatitis B (HBsAg) Seroprevalence
Liver cancer	Female	5-9 years	95+ years	1	Hepatitis C (IgG) Seroprevalence
Liver cancer	Female	5-9 years	95+ years	-1	Healthcare access and quality index
Larynx cancer	Male	15-19 years	95+ years	1	Alcohol (liters per capita)
Larynx cancer	Male	15-19 years	95+ years	1	Tobacco (cigarettes per capita)
Larynx cancer	Male	15-19 years	95+ years	1	Cumulative Cigarettes (10 Years)
Larynx cancer	Male	15-19 years	95+ years	1	Cumulative Cigarettes (15 Years)
Larynx cancer	Male	15-19 years	95+ years	1	Cumulative Cigarettes (20 Years)
Larynx cancer	Male	15-19 years	95+ years	1	Cumulative Cigarettes (5 Years)
Larynx cancer	Male	15-19 years	95+ years	-1	Education (years per capita)
Larynx cancer	Male	15-19 years	95+ years	0	LDI (I$ per capita)
Larynx cancer	Male	15-19 years	95+ years	1	Population Density (over 1000 ppl/sqkm, proportion)
Larynx cancer	Male	15-19 years	95+ years	1	Population Density (under 150 ppl/sqkm, proportion)
Larynx cancer	Male	15-19 years	95+ years	1	Smoking Prevalence
Larynx cancer	Male	15-19 years	95+ years	1	Log-transformed SEV scalar: Larynx C
Larynx cancer	Male	15-19 years	95+ years	0	Socio-demographic Index
Larynx cancer	Male	15-19 years	95+ years	-1	fruits adjusted (g)
Larynx cancer	Male	15-19 years	95+ years	-1	vegetables adjusted (g)
Larynx cancer	Male	15-19 years	95+ years	-1	Healthcare access and quality index
Larynx cancer	Female	15-19 years	95+ years	1	Alcohol (liters per capita)
Larynx cancer	Female	15-19 years	95+ years	1	Tobacco (cigarettes per capita)
Larynx cancer	Female	15-19 years	95+ years	1	Cumulative Cigarettes (10 Years)
Larynx cancer	Female	15-19 years	95+ years	1	Cumulative Cigarettes (15 Years)

© 2018 Fitzmaurice C et al. JAMA Oncology.

Cause	Sex	Age start	Age end	Direction	Covariate
Larynx cancer	Female	15-19 years	95+ years	1	Cumulative Cigarettes (20 Years)
Larynx cancer	Female	15-19 years	95+ years	1	Cumulative Cigarettes (5 Years)
Larynx cancer	Female	15-19 years	95+ years	-1	Education (years per capita)
Larynx cancer	Female	15-19 years	95+ years	0	LDI (I$ per capita)
Larynx cancer	Female	15-19 years	95+ years	1	Population Density (over 1000 ppl/sqkm, proportion)
Larynx cancer	Female	15-19 years	95+ years	1	Population Density (under 150 ppl/sqkm, proportion)
Larynx cancer	Female	15-19 years	95+ years	1	Smoking Prevalence
Larynx cancer	Female	15-19 years	95+ years	1	Log-transformed SEV scalar: Larynx C
Larynx cancer	Female	15-19 years	95+ years	0	Socio-demographic Index
Larynx cancer	Female	15-19 years	95+ years	-1	fruits adjusted (g)
Larynx cancer	Female	15-19 years	95+ years	-1	vegetables adjusted (g)
Larynx cancer	Female	15-19 years	95+ years	-1	Healthcare access and quality index
Tracheal, bronchus, and lung cancer	Female	15-19 years	95+ years	1	Tobacco (cigarettes per capita)
Tracheal, bronchus, and lung cancer	Female	15-19 years	95+ years	1	Cumulative Cigarettes (10 Years)
Tracheal, bronchus, and lung cancer	Female	15-19 years	95+ years	1	Cumulative Cigarettes (15 Years)
Tracheal, bronchus, and lung cancer	Female	15-19 years	95+ years	1	Cumulative Cigarettes (20 Years)
Tracheal, bronchus, and lung cancer	Female	15-19 years	95+ years	1	Cumulative Cigarettes (5 Years)
Tracheal, bronchus, and lung cancer	Female	15-19 years	95+ years	0	Education (years per capita)
Tracheal, bronchus, and lung cancer	Female	15-19 years	95+ years	0	LDI (I$ per capita)
Tracheal, bronchus, and lung cancer	Female	15-19 years	95+ years	1	Indoor Air Pollution (All Cooking Fuels)
Tracheal, bronchus, and lung cancer	Female	15-19 years	95+ years	1	Outdoor Air Pollution (PM2.5)
Tracheal, bronchus, and lung cancer	Female	15-19 years	95+ years	1	Smoking Prevalence
Tracheal, bronchus, and lung cancer	Female	15-19 years	95+ years	1	Log-transformed SEV scalar: Lung C
Tracheal, bronchus, and lung cancer	Female	15-19 years	95+ years	1	Log-transformed age-standardized SEV scalar: Lung C
Tracheal, bronchus, and lung cancer	Female	15-19 years	95+ years	0	Socio-demographic Index
Tracheal, bronchus, and lung cancer	Female	15-19 years	95+ years	-1	Healthcare access and quality index
Tracheal, bronchus, and lung cancer	Male	15-19 years	95+ years	1	Tobacco (cigarettes per capita)
Tracheal, bronchus, and lung cancer	Male	15-19 years	95+ years	1	Cumulative Cigarettes (10 Years)
Tracheal, bronchus, and lung cancer	Male	15-19 years	95+ years	1	Cumulative Cigarettes (15 Years)
Tracheal, bronchus, and lung cancer	Male	15-19 years	95+ years	1	Cumulative Cigarettes (20 Years)

© 2018 Fitzmaurice C et al. JAMA Oncology.

Cause	Sex	Age start	Age end	Direction	Covariate
Tracheal, bronchus, and lung cancer	Male	15-19 years	95+ years	1	Cumulative Cigarettes (5 Years)
Tracheal, bronchus, and lung cancer	Male	15-19 years	95+ years	0	Education (years per capita)
Tracheal, bronchus, and lung cancer	Male	15-19 years	95+ years	0	LDI (I$ per capita)
Tracheal, bronchus, and lung cancer	Male	15-19 years	95+ years	1	Indoor Air Pollution (All Cooking Fuels)
Tracheal, bronchus, and lung cancer	Male	15-19 years	95+ years	1	Outdoor Air Pollution (PM2.5)
Tracheal, bronchus, and lung cancer	Male	15-19 years	95+ years	1	Smoking Prevalence
Tracheal, bronchus, and lung cancer	Male	15-19 years	95+ years	1	Log-transformed SEV scalar: Lung C
Tracheal, bronchus, and lung cancer	Male	15-19 years	95+ years	1	Log-transformed age-standardized SEV scalar: Lung C
Tracheal, bronchus, and lung cancer	Male	15-19 years	95+ years	0	Socio-demographic Index
Tracheal, bronchus, and lung cancer	Male	15-19 years	95+ years	-1	Healthcare access and quality index
Breast cancer	Male	15-19 years	95+ years	1	Alcohol (liters per capita)
Breast cancer	Male	15-19 years	95+ years	1	Cumulative Cigarettes (10 Years)
Breast cancer	Male	15-19 years	95+ years	-1	Education (years per capita)
Breast cancer	Male	15-19 years	95+ years	1	Saturated Fats (kcal per capita)
Breast cancer	Male	15-19 years	95+ years	0	LDI (I$ per capita)
Breast cancer	Male	15-19 years	95+ years	1	Mean BMI
Breast cancer	Male	15-19 years	95+ years	1	Log-transformed SEV scalar: Breast C
Breast cancer	Male	15-19 years	95+ years	0	Socio-demographic Index
Breast cancer	Male	15-19 years	95+ years	-1	fruits adjusted (g)
Breast cancer	Male	15-19 years	95+ years	-1	vegetables adjusted (g)
Breast cancer	Male	15-19 years	95+ years	-1	Healthcare access and quality index
Breast cancer	Female	15-19 years	95+ years	1	Alcohol (liters per capita)
Breast cancer	Female	15-19 years	95+ years	-1	Age-Specific Fertility Rate
Breast cancer	Female	15-19 years	95+ years	1	Cumulative Cigarettes (10 Years)
Breast cancer	Female	15-19 years	95+ years	-1	Education (years per capita)
Breast cancer	Female	15-19 years	95+ years	1	Saturated Fats (kcal per capita)
Breast cancer	Female	15-19 years	95+ years	0	LDI (I$ per capita)
Breast cancer	Female	15-19 years	95+ years	1	Mean BMI
Breast cancer	Female	15-19 years	95+ years	-1	Total Fertility Rate
Breast cancer	Female	15-19 years	95+ years	1	Log-transformed SEV scalar: Breast C

© 2018 Fitzmaurice C et al. JAMA Oncology.

Cause	Sex	Age start	Age end	Direction	Covariate
Breast cancer	Female	15-19 years	95+ years	0	Socio-demographic Index
Breast cancer	Female	15-19 years	95+ years	-1	fruits adjusted (g)
Breast cancer	Female	15-19 years	95+ years	-1	vegetables adjusted (g)
Breast cancer	Female	15-19 years	95+ years	-1	Healthcare access and quality index
Cervical cancer	Female	15-19 years	95+ years	1	Abortion On-Demand Illegal (binary)
Cervical cancer	Female	15-19 years	95+ years	1	Age-Specific Fertility Rate
Cervical cancer	Female	15-19 years	95+ years	1	Cumulative Cigarettes (10 Years)
Cervical cancer	Female	15-19 years	95+ years	1	Cumulative Cigarettes (15 Years)
Cervical cancer	Female	15-19 years	95+ years	1	Cumulative Cigarettes (5 Years)
Cervical cancer	Female	15-19 years	95+ years	-1	Education (years per capita)
Cervical cancer	Female	15-19 years	95+ years	-1	Health System Access 2 (unitless)
Cervical cancer	Female	15-19 years	95+ years	0	LDI (I$ per capita)
Cervical cancer	Female	15-19 years	95+ years	1	Smoking Prevalence
Cervical cancer	Female	15-19 years	95+ years	1	Total Fertility Rate
Cervical cancer	Female	15-19 years	95+ years	0	Socio-demographic Index
Cervical cancer	Female	15-19 years	95+ years	-1	fruits adjusted (g)
Cervical cancer	Female	15-19 years	95+ years	-1	vegetables adjusted (g)
Cervical cancer	Female	15-19 years	95+ years	1	HIV age-standardized prevalence
Cervical cancer	Female	15-19 years	95+ years	-1	Healthcare access and quality index
Uterine cancer	Female	15-19 years	95+ years	1	Tobacco (cigarettes per capita)
Uterine cancer	Female	15-19 years	95+ years	1	Cumulative Cigarettes (10 Years)
Uterine cancer	Female	15-19 years	95+ years	1	Cumulative Cigarettes (5 Years)
Uterine cancer	Female	15-19 years	95+ years	1	Diabetes Age-Standardized Prevalence (proportion)
Uterine cancer	Female	15-19 years	95+ years	-1	Education (years per capita)
Uterine cancer	Female	15-19 years	95+ years	-1	Health System Access (unitless)
Uterine cancer	Female	15-19 years	95+ years	0	LDI (I$ per capita)
Uterine cancer	Female	15-19 years	95+ years	1	Mean BMI
Uterine cancer	Female	15-19 years	95+ years	1	Smoking Prevalence
Uterine cancer	Female	15-19 years	95+ years	0	Total Fertility Rate
Uterine cancer	Female	15-19 years	95+ years	1	Log-transformed SEV scalar: Uterus C

© 2018 Fitzmaurice C et al. JAMA Oncology.

Cause	Sex	Age start	Age end	Direction	Covariate
Uterine cancer	Female	15-19 years	95+ years	0	Socio-demographic Index
Uterine cancer	Female	15-19 years	95+ years	-1	fruits adjusted (g)
Uterine cancer	Female	15-19 years	95+ years	-1	vegetables adjusted (g)
Uterine cancer	Female	15-19 years	95+ years	-1	Healthcare access and quality index
Prostate cancer	Male	15-19 years	95+ years	0	Education (years per capita)
Prostate cancer	Male	15-19 years	95+ years	0	LDI (I$ per capita)
Prostate cancer	Male	15-19 years	95+ years	1	Percent of total calories consumed as saturated fat
Prostate cancer	Male	15-19 years	95+ years	1	Log-transformed SEV scalar: Prostate C
Prostate cancer	Male	15-19 years	95+ years	0	Socio-demographic Index
Prostate cancer	Male	15-19 years	95+ years	-1	Healthcare access and quality index
Colon and rectum cancer	Male	15-19 years	95+ years	1	Alcohol (liters per capita)
Colon and rectum cancer	Male	15-19 years	95+ years	1	Tobacco (cigarettes per capita)
Colon and rectum cancer	Male	15-19 years	95+ years	1	Diabetes Age-Standardized Prevalence (proportion)
Colon and rectum cancer	Male	15-19 years	95+ years	-1	Education (years per capita)
Colon and rectum cancer	Male	15-19 years	95+ years	-1	Health System Access 2 (unitless)
Colon and rectum cancer	Male	15-19 years	95+ years	0	LDI (I$ per capita)
Colon and rectum cancer	Male	15-19 years	95+ years	1	Mean BMI
Colon and rectum cancer	Male	15-19 years	95+ years	1	Smoking Prevalence
Colon and rectum cancer	Male	15-19 years	95+ years	1	Log-transformed SEV scalar: Colorect C
Colon and rectum cancer	Male	15-19 years	95+ years	0	Socio-demographic Index
Colon and rectum cancer	Male	15-19 years	95+ years	-1	fruits adjusted (g)
Colon and rectum cancer	Male	15-19 years	95+ years	-1	milk adjusted (g)
Colon and rectum cancer	Male	15-19 years	95+ years	-1	nuts seeds adjusted (g)
Colon and rectum cancer	Male	15-19 years	95+ years	-1	PUFA adjusted (percent)
Colon and rectum cancer	Male	15-19 years	95+ years	1	red meats adjusted (g)
Colon and rectum cancer	Male	15-19 years	95+ years	-1	vegetables adjusted (g)
Colon and rectum cancer	Male	15-19 years	95+ years	-1	whole grains adjusted (g)
Colon and rectum cancer	Male	15-19 years	95+ years	-1	Healthcare access and quality index
Colon and rectum cancer	Female	15-19 years	95+ years	1	Alcohol (liters per capita)
Colon and rectum cancer	Female	15-19 years	95+ years	1	Tobacco (cigarettes per capita)

© 2018 Fitzmaurice C et al. JAMA Oncology.

Cause	Sex	Age start	Age end	Direction	Covariate
Colon and rectum cancer	Female	15-19 years	95+ years	1	Diabetes Age-Standardized Prevalence (proportion)
Colon and rectum cancer	Female	15-19 years	95+ years	-1	Education (years per capita)
Colon and rectum cancer	Female	15-19 years	95+ years	-1	Health System Access 2 (unitless)
Colon and rectum cancer	Female	15-19 years	95+ years	0	LDI (I$ per capita)
Colon and rectum cancer	Female	15-19 years	95+ years	1	Mean BMI
Colon and rectum cancer	Female	15-19 years	95+ years	1	Smoking Prevalence
Colon and rectum cancer	Female	15-19 years	95+ years	1	Log-transformed SEV scalar: Colorect C
Colon and rectum cancer	Female	15-19 years	95+ years	0	Socio-demographic Index
Colon and rectum cancer	Female	15-19 years	95+ years	-1	fruits adjusted (g)
Colon and rectum cancer	Female	15-19 years	95+ years	-1	milk adjusted (g)
Colon and rectum cancer	Female	15-19 years	95+ years	-1	nuts seeds adjusted (g)
Colon and rectum cancer	Female	15-19 years	95+ years	-1	PUFA adjusted (percent)
Colon and rectum cancer	Female	15-19 years	95+ years	1	red meats adjusted (g)
Colon and rectum cancer	Female	15-19 years	95+ years	-1	vegetables adjusted (g)
Colon and rectum cancer	Female	15-19 years	95+ years	-1	whole grains adjusted (g)
Colon and rectum cancer	Female	15-19 years	95+ years	-1	Healthcare access and quality index
Lip and oral cavity cancer	Male	15-19 years	95+ years	1	Alcohol (liters per capita)
Lip and oral cavity cancer	Male	15-19 years	95+ years	1	Tobacco (cigarettes per capita)
Lip and oral cavity cancer	Male	15-19 years	95+ years	1	Cumulative Cigarettes (10 Years)
Lip and oral cavity cancer	Male	15-19 years	95+ years	1	Cumulative Cigarettes (15 Years)
Lip and oral cavity cancer	Male	15-19 years	95+ years	1	Cumulative Cigarettes (20 Years)
Lip and oral cavity cancer	Male	15-19 years	95+ years	1	Cumulative Cigarettes (5 Years)
Lip and oral cavity cancer	Male	15-19 years	95+ years	-1	Education (years per capita)
Lip and oral cavity cancer	Male	15-19 years	95+ years	-1	Health System Access 2 (unitless)
Lip and oral cavity cancer	Male	15-19 years	95+ years	0	LDI (I$ per capita)
Lip and oral cavity cancer	Male	15-19 years	95+ years	1	Smoking Prevalence
Lip and oral cavity cancer	Male	15-19 years	95+ years	1	Log-transformed SEV scalar: Mouth C
Lip and oral cavity cancer	Male	15-19 years	95+ years	0	Socio-demographic Index
Lip and oral cavity cancer	Male	15-19 years	95+ years	-1	fruits adjusted (g)
Lip and oral cavity cancer	Male	15-19 years	95+ years	1	red meats adjusted (g)

© 2018 Fitzmaurice C et al. JAMA Oncology.

Cause	Sex	Age start	Age end	Direction	Covariate
Lip and oral cavity cancer	Male	15-19 years	95+ years	-1	vegetables adjusted (g)
Lip and oral cavity cancer	Male	15-19 years	95+ years	-1	Healthcare access and quality index
Lip and oral cavity cancer	Female	15-19 years	95+ years	1	Alcohol (liters per capita)
Lip and oral cavity cancer	Female	15-19 years	95+ years	1	Cumulative Cigarettes (10 Years)
Lip and oral cavity cancer	Female	15-19 years	95+ years	1	Cumulative Cigarettes (20 Years)
Lip and oral cavity cancer	Female	15-19 years	95+ years	-1	Education (years per capita)
Lip and oral cavity cancer	Female	15-19 years	95+ years	-1	Fruits (kcal per capita)
Lip and oral cavity cancer	Female	15-19 years	95+ years	-1	Health System Access 2 (unitless)
Lip and oral cavity cancer	Female	15-19 years	95+ years	-1	LDI (I$ per capita)
Lip and oral cavity cancer	Female	15-19 years	95+ years	1	Red Meat (kcal per capita)
Lip and oral cavity cancer	Female	15-19 years	95+ years	1	Smoking Prevalence
Lip and oral cavity cancer	Female	15-19 years	95+ years	-1	Vegetables (kcal per capita)
Lip and oral cavity cancer	Female	15-19 years	95+ years	0	Socio-demographic Index
Lip and oral cavity cancer	Female	15-19 years	95+ years	-1	Healthcare access and quality index
Nasopharynx cancer	Female	5-9 years	95+ years	1	Alcohol (liters per capita)
Nasopharynx cancer	Female	5-9 years	95+ years	1	Tobacco (cigarettes per capita)
Nasopharynx cancer	Female	5-9 years	95+ years	1	Cumulative Cigarettes (10 Years)
Nasopharynx cancer	Female	5-9 years	95+ years	1	Cumulative Cigarettes (15 Years)
Nasopharynx cancer	Female	5-9 years	95+ years	1	Cumulative Cigarettes (20 Years)
Nasopharynx cancer	Female	5-9 years	95+ years	1	Cumulative Cigarettes (5 Years)
Nasopharynx cancer	Female	5-9 years	95+ years	-1	Education (years per capita)
Nasopharynx cancer	Female	5-9 years	95+ years	-1	Health System Access 2 (unitless)
Nasopharynx cancer	Female	5-9 years	95+ years	0	LDI (I$ per capita)
Nasopharynx cancer	Female	5-9 years	95+ years	1	Population Density (over 1000 ppl/sqkm, proportion)
Nasopharynx cancer	Female	5-9 years	95+ years	1	Population Density (under 150 ppl/sqkm, proportion)
Nasopharynx cancer	Female	5-9 years	95+ years	1	Smoking Prevalence
Nasopharynx cancer	Female	5-9 years	95+ years	1	Log-transformed SEV scalar: Nasoph C
Nasopharynx cancer	Female	5-9 years	95+ years	0	Socio-demographic Index
Nasopharynx cancer	Female	5-9 years	95+ years	-1	fruits adjusted (g)
Nasopharynx cancer	Female	5-9 years	95+ years	-1	vegetables adjusted (g)

© 2018 Fitzmaurice C et al. JAMA Oncology.

Cause	Sex	Age start	Age end	Direction	Covariate
Nasopharynx cancer	Female	5-9 years	95+ years	-1	whole grains adjusted (g)
Nasopharynx cancer	Male	5-9 years	95+ years	1	Alcohol (liters per capita)
Nasopharynx cancer	Male	5-9 years	95+ years	1	Tobacco (cigarettes per capita)
Nasopharynx cancer	Male	5-9 years	95+ years	1	Cumulative Cigarettes (10 Years)
Nasopharynx cancer	Male	5-9 years	95+ years	1	Cumulative Cigarettes (15 Years)
Nasopharynx cancer	Male	5-9 years	95+ years	1	Cumulative Cigarettes (20 Years)
Nasopharynx cancer	Male	5-9 years	95+ years	1	Cumulative Cigarettes (5 Years)
Nasopharynx cancer	Male	5-9 years	95+ years	-1	Education (years per capita)
Nasopharynx cancer	Male	5-9 years	95+ years	-1	Health System Access 2 (unitless)
Nasopharynx cancer	Male	5-9 years	95+ years	0	LDI (I$ per capita)
Nasopharynx cancer	Male	5-9 years	95+ years	1	Population Density (over 1000 ppl/sqkm, proportion)
Nasopharynx cancer	Male	5-9 years	95+ years	1	Population Density (under 150 ppl/sqkm, proportion)
Nasopharynx cancer	Male	5-9 years	95+ years	1	Smoking Prevalence
Nasopharynx cancer	Male	5-9 years	95+ years	1	Log-transformed SEV scalar: Nasoph C
Nasopharynx cancer	Male	5-9 years	95+ years	0	Socio-demographic Index
Nasopharynx cancer	Male	5-9 years	95+ years	-1	fruits adjusted (g)
Nasopharynx cancer	Male	5-9 years	95+ years	-1	vegetables adjusted (g)
Nasopharynx cancer	Male	5-9 years	95+ years	-1	whole grains adjusted (g)
Other pharynx cancer	Male	15-19 years	95+ years	1	Alcohol (liters per capita)
Other pharynx cancer	Male	15-19 years	95+ years	1	Cumulative Cigarettes (5 Years)
Other pharynx cancer	Male	15-19 years	95+ years	-1	Education (years per capita)
Other pharynx cancer	Male	15-19 years	95+ years	0	LDI (I$ per capita)
Other pharynx cancer	Male	15-19 years	95+ years	1	Population Density (over 1000 ppl/sqkm, proportion)
Other pharynx cancer	Male	15-19 years	95+ years	1	Population Density (under 150 ppl/sqkm, proportion)
Other pharynx cancer	Male	15-19 years	95+ years	1	Smoking Prevalence
Other pharynx cancer	Male	15-19 years	95+ years	-1	Health System Access (capped)
Other pharynx cancer	Male	15-19 years	95+ years	1	Log-transformed SEV scalar: Oth Phar C
Other pharynx cancer	Male	15-19 years	95+ years	0	Socio-demographic Index
Other pharynx cancer	Male	15-19 years	95+ years	-1	fruits adjusted (g)
Other pharynx cancer	Male	15-19 years	95+ years	-1	vegetables adjusted (g)

© 2018 Fitzmaurice C et al. JAMA Oncology.

Cause	Sex	Age start	Age end	Direction	Covariate
Other pharynx cancer	Male	15-19 years	95+ years	-1	whole grains adjusted (g)
Other pharynx cancer	Female	15-19 years	95+ years	1	Alcohol (liters per capita)
Other pharynx cancer	Female	15-19 years	95+ years	1	Cumulative Cigarettes (5 Years)
Other pharynx cancer	Female	15-19 years	95+ years	-1	Education (years per capita)
Other pharynx cancer	Female	15-19 years	95+ years	0	LDI (I$ per capita)
Other pharynx cancer	Female	15-19 years	95+ years	1	Population Density (over 1000 ppl/sqkm, proportion)
Other pharynx cancer	Female	15-19 years	95+ years	1	Population Density (under 150 ppl/sqkm, proportion)
Other pharynx cancer	Female	15-19 years	95+ years	1	Smoking Prevalence
Other pharynx cancer	Female	15-19 years	95+ years	-1	Health System Access (capped)
Other pharynx cancer	Female	15-19 years	95+ years	1	Log-transformed SEV scalar: Oth Phar C
Other pharynx cancer	Female	15-19 years	95+ years	0	Socio-demographic Index
Other pharynx cancer	Female	15-19 years	95+ years	-1	fruits adjusted (g)
Other pharynx cancer	Female	15-19 years	95+ years	-1	vegetables adjusted (g)
Other pharynx cancer	Female	15-19 years	95+ years	-1	whole grains adjusted (g)
Gallbladder and biliary tract cancer	Female	15-19 years	95+ years	1	Alcohol (liters per capita)
Gallbladder and biliary tract cancer	Female	15-19 years	95+ years	1	Tobacco (cigarettes per capita)
Gallbladder and biliary tract cancer	Female	15-19 years	95+ years	1	Cumulative Cigarettes (10 Years)
Gallbladder and biliary tract cancer	Female	15-19 years	95+ years	1	Cumulative Cigarettes (5 Years)
Gallbladder and biliary tract cancer	Female	15-19 years	95+ years	1	Diabetes Age-Standardized Prevalence (proportion)
Gallbladder and biliary tract cancer	Female	15-19 years	95+ years	-1	Education (years per capita)
Gallbladder and biliary tract cancer	Female	15-19 years	95+ years	1	Health System Access 2 (unitless)
Gallbladder and biliary tract cancer	Female	15-19 years	95+ years	0	LDI (I$ per capita)
Gallbladder and biliary tract cancer	Female	15-19 years	95+ years	1	Mean BMI
Gallbladder and biliary tract cancer	Female	15-19 years	95+ years	1	Smoking Prevalence
Gallbladder and biliary tract cancer	Female	15-19 years	95+ years	1	Log-transformed SEV scalar: Gallblad C
Gallbladder and biliary tract cancer	Female	15-19 years	95+ years	0	Socio-demographic Index
Gallbladder and biliary tract cancer	Female	15-19 years	95+ years	-1	fruits adjusted (g)
Gallbladder and biliary tract cancer	Female	15-19 years	95+ years	-1	vegetables adjusted (g)
Gallbladder and biliary tract cancer	Female	15-19 years	95+ years	-1	Healthcare access and quality index
Gallbladder and biliary tract cancer	Female	15-19 years	95+ years	-1	Health System Access (capped)

© 2018 Fitzmaurice C et al. JAMA Oncology.

Cause	Sex	Age start	Age end	Direction	Covariate
Gallbladder and biliary tract cancer	Male	15-19 years	95+ years	1	Alcohol (liters per capita)
Gallbladder and biliary tract cancer	Male	15-19 years	95+ years	1	Tobacco (cigarettes per capita)
Gallbladder and biliary tract cancer	Male	15-19 years	95+ years	1	Cumulative Cigarettes (10 Years)
Gallbladder and biliary tract cancer	Male	15-19 years	95+ years	1	Cumulative Cigarettes (5 Years)
Gallbladder and biliary tract cancer	Male	15-19 years	95+ years	1	Diabetes Age-Standardized Prevalence (proportion)
Gallbladder and biliary tract cancer	Male	15-19 years	95+ years	-1	Education (years per capita)
Gallbladder and biliary tract cancer	Male	15-19 years	95+ years	0	LDI (I$ per capita)
Gallbladder and biliary tract cancer	Male	15-19 years	95+ years	1	Mean BMI
Gallbladder and biliary tract cancer	Male	15-19 years	95+ years	1	Smoking Prevalence
Gallbladder and biliary tract cancer	Male	15-19 years	95+ years	-1	Health System Access (capped)
Gallbladder and biliary tract cancer	Male	15-19 years	95+ years	1	Log-transformed SEV scalar: Gallblad C
Gallbladder and biliary tract cancer	Male	15-19 years	95+ years	0	Socio-demographic Index
Gallbladder and biliary tract cancer	Male	15-19 years	95+ years	-1	fruits adjusted (g)
Gallbladder and biliary tract cancer	Male	15-19 years	95+ years	-1	vegetables adjusted (g)
Gallbladder and biliary tract cancer	Male	15-19 years	95+ years	-1	Healthcare access and quality index
Pancreatic cancer	Male	15-19 years	95+ years	1	Alcohol (liters per capita)
Pancreatic cancer	Male	15-19 years	95+ years	1	Tobacco (cigarettes per capita)
Pancreatic cancer	Male	15-19 years	95+ years	1	Cumulative Cigarettes (10 Years)
Pancreatic cancer	Male	15-19 years	95+ years	1	Cumulative Cigarettes (20 Years)
Pancreatic cancer	Male	15-19 years	95+ years	1	Cumulative Cigarettes (5 Years)
Pancreatic cancer	Male	15-19 years	95+ years	1	Diabetes Age-Standardized Prevalence (proportion)
Pancreatic cancer	Male	15-19 years	95+ years	-1	Education (years per capita)
Pancreatic cancer	Male	15-19 years	95+ years	0	LDI (I$ per capita)
Pancreatic cancer	Male	15-19 years	95+ years	1	Mean BMI
Pancreatic cancer	Male	15-19 years	95+ years	1	Percent of total calories consumed as saturated fat
Pancreatic cancer	Male	15-19 years	95+ years	1	Smoking Prevalence
Pancreatic cancer	Male	15-19 years	95+ years	1	Total Calories (kcal per capita)
Pancreatic cancer	Male	15-19 years	95+ years	1	Log-transformed SEV scalar: Pancreas C
Pancreatic cancer	Male	15-19 years	95+ years	0	Socio-demographic Index
Pancreatic cancer	Male	15-19 years	95+ years	-1	fruits adjusted (g)

© 2018 Fitzmaurice C et al. JAMA Oncology.

Cause	Sex	Age start	Age end	Direction	Covariate
Pancreatic cancer	Male	15-19 years	95+ years	1	red meats adjusted (g)
Pancreatic cancer	Male	15-19 years	95+ years	-1	vegetables adjusted (g)
Pancreatic cancer	Male	15-19 years	95+ years	-1	Healthcare access and quality index
Pancreatic cancer	Female	15-19 years	95+ years	1	Alcohol (liters per capita)
Pancreatic cancer	Female	15-19 years	95+ years	1	Tobacco (cigarettes per capita)
Pancreatic cancer	Female	15-19 years	95+ years	1	Cumulative Cigarettes (10 Years)
Pancreatic cancer	Female	15-19 years	95+ years	1	Cumulative Cigarettes (20 Years)
Pancreatic cancer	Female	15-19 years	95+ years	1	Cumulative Cigarettes (5 Years)
Pancreatic cancer	Female	15-19 years	95+ years	1	Diabetes Age-Standardized Prevalence (proportion)
Pancreatic cancer	Female	15-19 years	95+ years	-1	Education (years per capita)
Pancreatic cancer	Female	15-19 years	95+ years	0	LDI (I$ per capita)
Pancreatic cancer	Female	15-19 years	95+ years	1	Mean BMI
Pancreatic cancer	Female	15-19 years	95+ years	1	Percent of total calories consumed as saturated fat
Pancreatic cancer	Female	15-19 years	95+ years	1	Smoking Prevalence
Pancreatic cancer	Female	15-19 years	95+ years	1	Log-transformed SEV scalar: Pancreas C
Pancreatic cancer	Female	15-19 years	95+ years	0	Socio-demographic Index
Pancreatic cancer	Female	15-19 years	95+ years	-1	fruits adjusted (g)
Pancreatic cancer	Female	15-19 years	95+ years	1	red meats adjusted (g)
Pancreatic cancer	Female	15-19 years	95+ years	-1	vegetables adjusted (g)
Pancreatic cancer	Female	15-19 years	95+ years	1	energy unadjusted (kcal)
Pancreatic cancer	Female	15-19 years	95+ years	-1	Healthcare access and quality index
Pancreatic cancer	Male	15-19 years	95+ years	1	energy unadjusted (kcal)
Pancreatic cancer	Female	15-19 years	95+ years	1	vegetables adjusted (g)
Pancreatic cancer	Female	15-19 years	95+ years	-1	vegetables unadjusted (g)
Malignant skin melanoma	Male	15-19 years	95+ years	1	Alcohol (liters per capita)
Malignant skin melanoma	Male	15-19 years	95+ years	-1	Education (years per capita)
Malignant skin melanoma	Male	15-19 years	95+ years	0	LDI (I$ per capita)
Malignant skin melanoma	Male	15-19 years	95+ years	0	Latitude Under 15 (proportion)
Malignant skin melanoma	Male	15-19 years	95+ years	0	Latitude 15 to 30 (proportion)
Malignant skin melanoma	Male	15-19 years	95+ years	-1	Latitude 30 to 45 (proportion)

© 2018 Fitzmaurice C et al. JAMA Oncology.

Cause	Sex	Age start	Age end	Direction	Covariate
Malignant skin melanoma	Male	15-19 years	95+ years	-1	Latitude Over 45 (proportion)
Malignant skin melanoma	Male	15-19 years	95+ years	0	Socio-demographic Index
Malignant skin melanoma	Male	15-19 years	95+ years	-1	fruits adjusted (g)
Malignant skin melanoma	Male	15-19 years	95+ years	-1	vegetables adjusted (g)
Malignant skin melanoma	Male	15-19 years	95+ years	-1	Healthcare access and quality index
Malignant skin melanoma	Female	15-19 years	95+ years	1	Alcohol (liters per capita)
Malignant skin melanoma	Female	15-19 years	95+ years	-1	Education (years per capita)
Malignant skin melanoma	Female	15-19 years	95+ years	0	LDI (I$ per capita)
Malignant skin melanoma	Female	15-19 years	95+ years	0	Latitude Under 15 (proportion)
Malignant skin melanoma	Female	15-19 years	95+ years	0	Latitude 15 to 30 (proportion)
Malignant skin melanoma	Female	15-19 years	95+ years	-1	Latitude 30 to 45 (proportion)
Malignant skin melanoma	Female	15-19 years	95+ years	-1	Latitude Over 45 (proportion)
Malignant skin melanoma	Female	15-19 years	95+ years	0	Socio-demographic Index
Malignant skin melanoma	Female	15-19 years	95+ years	-1	fruits adjusted (g)
Malignant skin melanoma	Female	15-19 years	95+ years	-1	vegetables adjusted (g)
Malignant skin melanoma	Female	15-19 years	95+ years	-1	Healthcare access and quality index
Non-melanoma skin cancer	Male	15-19 years	95+ years	1	Cumulative Cigarettes (10 Years)
Non-melanoma skin cancer	Male	15-19 years	95+ years	1	Cumulative Cigarettes (15 Years)
Non-melanoma skin cancer	Male	15-19 years	95+ years	1	Cumulative Cigarettes (5 Years)
Non-melanoma skin cancer	Male	15-19 years	95+ years	-1	Education (years per capita)
Non-melanoma skin cancer	Male	15-19 years	95+ years	0	Average latitude
Non-melanoma skin cancer	Male	15-19 years	95+ years	0	LDI (I$ per capita)
Non-melanoma skin cancer	Male	15-19 years	95+ years	1	Smoking Prevalence
Non-melanoma skin cancer	Male	15-19 years	95+ years	0	Socio-demographic Index
Non-melanoma skin cancer	Male	15-19 years	95+ years	-1	Healthcare access and quality index
Non-melanoma skin cancer	Male	15-19 years	95+ years	-1	Health System Access (capped)
Non-melanoma skin cancer	Female	15-19 years	95+ years	1	Cumulative Cigarettes (10 Years)
Non-melanoma skin cancer	Female	15-19 years	95+ years	1	Cumulative Cigarettes (15 Years)
Non-melanoma skin cancer	Female	15-19 years	95+ years	1	Cumulative Cigarettes (5 Years)
Non-melanoma skin cancer	Female	15-19 years	95+ years	-1	Education (years per capita)

© 2018 Fitzmaurice C et al. JAMA Oncology.

Cause	Sex	Age start	Age end	Direction	Covariate
Non-melanoma skin cancer	Female	15-19 years	95+ years	0	Average latitude
Non-melanoma skin cancer	Female	15-19 years	95+ years	0	LDI (I$ per capita)
Non-melanoma skin cancer	Female	15-19 years	95+ years	1	Smoking Prevalence
Non-melanoma skin cancer	Female	15-19 years	95+ years	0	Socio-demographic Index
Non-melanoma skin cancer	Female	15-19 years	95+ years	-1	Healthcare access and quality index
Non-melanoma skin cancer	Female	15-19 years	95+ years	-1	Health System Access (capped)
Ovarian cancer	Female	15-19 years	95+ years	1	Alcohol (liters per capita)
Ovarian cancer	Female	15-19 years	95+ years	1	Tobacco (cigarettes per capita)
Ovarian cancer	Female	15-19 years	95+ years	-1	Contraception (Modern) Prevalence (proportion)
Ovarian cancer	Female	15-19 years	95+ years	1	Cumulative Cigarettes (20 Years)
Ovarian cancer	Female	15-19 years	95+ years	1	Diabetes Age-Standardized Prevalence (proportion)
Ovarian cancer	Female	15-19 years	95+ years	-1	Education (years per capita)
Ovarian cancer	Female	15-19 years	95+ years	0	LDI (I$ per capita)
Ovarian cancer	Female	15-19 years	95+ years	1	Mean BMI
Ovarian cancer	Female	15-19 years	95+ years	1	Percent of total calories consumed as saturated fat
Ovarian cancer	Female	15-19 years	95+ years	1	Smoking Prevalence
Ovarian cancer	Female	15-19 years	95+ years	0	Total Fertility Rate
Ovarian cancer	Female	15-19 years	95+ years	1	Log-transformed SEV scalar: Ovary C
Ovarian cancer	Female	15-19 years	95+ years	0	Socio-demographic Index
Ovarian cancer	Female	15-19 years	95+ years	-1	fruits adjusted (g)
Ovarian cancer	Female	15-19 years	95+ years	-1	vegetables adjusted (g)
Ovarian cancer	Female	15-19 years	95+ years	1	energy unadjusted (kcal)
Ovarian cancer	Female	15-19 years	95+ years	-1	Healthcare access and quality index
Testicular cancer	Male	15-19 years	95+ years	1	Cumulative Cigarettes (10 Years)
Testicular cancer	Male	15-19 years	95+ years	1	Cumulative Cigarettes (15 Years)
Testicular cancer	Male	15-19 years	95+ years	1	Cumulative Cigarettes (5 Years)
Testicular cancer	Male	15-19 years	95+ years	-1	Education (years per capita)
Testicular cancer	Male	15-19 years	95+ years	-1	Fruits (kcal per capita)
Testicular cancer	Male	15-19 years	95+ years	-1	Health System Access 2 (unitless)
Testicular cancer	Male	15-19 years	95+ years	-1	LDI (I$ per capita)

© 2018 Fitzmaurice C et al. JAMA Oncology.

Cause	Sex	Age start	Age end	Direction	Covariate
Testicular cancer	Male	15-19 years	95+ years	-1	Vegetables (kcal per capita)
Testicular cancer	Male	15-19 years	95+ years	0	Socio-demographic Index
Testicular cancer	Male	15-19 years	95+ years	-1	Healthcare access and quality index
Kidney cancer	Male	0-6 days	95+ years	1	Alcohol (liters per capita)
Kidney cancer	Male	0-6 days	95+ years	1	Cumulative Cigarettes (10 Years)
Kidney cancer	Male	0-6 days	95+ years	1	Cumulative Cigarettes (15 Years)
Kidney cancer	Male	0-6 days	95+ years	1	Cumulative Cigarettes (5 Years)
Kidney cancer	Male	0-6 days	95+ years	1	Diabetes Age-Standardized Prevalence (proportion)
Kidney cancer	Male	0-6 days	95+ years	-1	Education (years per capita)
Kidney cancer	Male	0-6 days	95+ years	-1	Health System Access 2 (unitless)
Kidney cancer	Male	0-6 days	95+ years	0	LDI (I$ per capita)
Kidney cancer	Male	0-6 days	95+ years	1	Mean BMI
Kidney cancer	Male	0-6 days	95+ years	1	Systolic Blood Pressure (mmHg)
Kidney cancer	Male	0-6 days	95+ years	1	Smoking Prevalence
Kidney cancer	Male	0-6 days	95+ years	1	Log-transformed SEV scalar: Kidney C
Kidney cancer	Male	0-6 days	95+ years	0	Socio-demographic Index
Kidney cancer	Female	0-6 days	95+ years	1	Alcohol (liters per capita)
Kidney cancer	Female	0-6 days	95+ years	1	Cumulative Cigarettes (10 Years)
Kidney cancer	Female	0-6 days	95+ years	1	Cumulative Cigarettes (15 Years)
Kidney cancer	Female	0-6 days	95+ years	1	Cumulative Cigarettes (5 Years)
Kidney cancer	Female	0-6 days	95+ years	1	Diabetes Age-Standardized Prevalence (proportion)
Kidney cancer	Female	0-6 days	95+ years	-1	Education (years per capita)
Kidney cancer	Female	0-6 days	95+ years	-1	Health System Access 2 (unitless)
Kidney cancer	Female	0-6 days	95+ years	-1	LDI (I$ per capita)
Kidney cancer	Female	0-6 days	95+ years	1	Mean BMI
Kidney cancer	Female	0-6 days	95+ years	1	Systolic Blood Pressure (mmHg)
Kidney cancer	Female	0-6 days	95+ years	1	Smoking Prevalence
Kidney cancer	Female	0-6 days	95+ years	0	Total Fertility Rate
Kidney cancer	Female	0-6 days	95+ years	1	Total Calories (kcal per capita)
Kidney cancer	Female	0-6 days	95+ years	1	Log-transformed SEV scalar: Kidney C

© 2018 Fitzmaurice C et al. JAMA Oncology.

Cause	Sex	Age start	Age end	Direction	Covariate
Kidney cancer	Female	0-6 days	95+ years	1	Socio-demographic Index
Kidney cancer	Female	0-6 days	95+ years	0	LDI (I$ per capita)
Kidney cancer	Female	0-6 days	95+ years	0	Socio-demographic Index
Bladder cancer	Male	15-19 years	95+ years	1	Alcohol (liters per capita)
Bladder cancer	Male	15-19 years	95+ years	1	Cumulative Cigarettes (10 Years)
Bladder cancer	Male	15-19 years	95+ years	1	Cumulative Cigarettes (15 Years)
Bladder cancer	Male	15-19 years	95+ years	1	Cumulative Cigarettes (5 Years)
Bladder cancer	Male	15-19 years	95+ years	-1	Education (years per capita)
Bladder cancer	Male	15-19 years	95+ years	0	LDI (I$ per capita)
Bladder cancer	Male	15-19 years	95+ years	1	Population Density (over 1000 ppl/sqkm, proportion)
Bladder cancer	Male	15-19 years	95+ years	1	Population Density (under 150 ppl/sqkm, proportion)
Bladder cancer	Male	15-19 years	95+ years	1	Smoking Prevalence
Bladder cancer	Male	15-19 years	95+ years	1	Log-transformed SEV scalar: Bladder C
Bladder cancer	Male	15-19 years	95+ years	0	Socio-demographic Index
Bladder cancer	Male	15-19 years	95+ years	-1	fruits adjusted (g)
Bladder cancer	Male	15-19 years	95+ years	-1	vegetables adjusted (g)
Bladder cancer	Male	15-19 years	95+ years	-1	Healthcare access and quality index
Bladder cancer	Female	15-19 years	95+ years	1	Alcohol (liters per capita)
Bladder cancer	Female	15-19 years	95+ years	1	Cumulative Cigarettes (10 Years)
Bladder cancer	Female	15-19 years	95+ years	1	Cumulative Cigarettes (15 Years)
Bladder cancer	Female	15-19 years	95+ years	1	Cumulative Cigarettes (5 Years)
Bladder cancer	Female	15-19 years	95+ years	-1	Education (years per capita)
Bladder cancer	Female	15-19 years	95+ years	0	LDI (I$ per capita)
Bladder cancer	Female	15-19 years	95+ years	1	Population Density (over 1000 ppl/sqkm, proportion)
Bladder cancer	Female	15-19 years	95+ years	1	Population Density (under 150 ppl/sqkm, proportion)
Bladder cancer	Female	15-19 years	95+ years	1	Smoking Prevalence
Bladder cancer	Female	15-19 years	95+ years	1	Log-transformed SEV scalar: Bladder C
Bladder cancer	Female	15-19 years	95+ years	0	Socio-demographic Index
Bladder cancer	Female	15-19 years	95+ years	-1	fruits adjusted (g)
Bladder cancer	Female	15-19 years	95+ years	-1	vegetables adjusted (g)

© 2018 Fitzmaurice C et al. JAMA Oncology.

Cause	Sex	Age start	Age end	Direction	Covariate
Bladder cancer	Female	15-19 years	95+ years	-1	Healthcare access and quality index
Brain and nervous system cancer	Female	0-6 days	95+ years	1	Alcohol (liters per capita)
Brain and nervous system cancer	Female	0-6 days	95+ years	1	Cumulative Cigarettes (10 Years)
Brain and nervous system cancer	Female	0-6 days	95+ years	1	Cumulative Cigarettes (15 Years)
Brain and nervous system cancer	Female	0-6 days	95+ years	-1	Education (years per capita)
Brain and nervous system cancer	Female	0-6 days	95+ years	0	LDI (I$ per capita)
Brain and nervous system cancer	Female	0-6 days	95+ years	1	Cholesterol (total, mean per capita)
Brain and nervous system cancer	Female	0-6 days	95+ years	1	Systolic Blood Pressure (mmHg)
Brain and nervous system cancer	Female	0-6 days	95+ years	1	Percent of total calories consumed as saturated fat
Brain and nervous system cancer	Female	0-6 days	95+ years	1	Smoking Prevalence
Brain and nervous system cancer	Female	0-6 days	95+ years	0	Socio-demographic Index
Brain and nervous system cancer	Female	0-6 days	95+ years	-1	fruits adjusted (g)
Brain and nervous system cancer	Female	0-6 days	95+ years	1	red meats adjusted (g)
Brain and nervous system cancer	Female	0-6 days	95+ years	-1	vegetables adjusted (g)
Brain and nervous system cancer	Female	0-6 days	95+ years	-1	Healthcare access and quality index
Brain and nervous system cancer	Male	0-6 days	95+ years	1	Alcohol (liters per capita)
Brain and nervous system cancer	Male	0-6 days	95+ years	1	Cumulative Cigarettes (10 Years)
Brain and nervous system cancer	Male	0-6 days	95+ years	1	Cumulative Cigarettes (15 Years)
Brain and nervous system cancer	Male	0-6 days	95+ years	-1	Education (years per capita)
Brain and nervous system cancer	Male	0-6 days	95+ years	0	LDI (I$ per capita)
Brain and nervous system cancer	Male	0-6 days	95+ years	1	Cholesterol (total, mean per capita)
Brain and nervous system cancer	Male	0-6 days	95+ years	1	Systolic Blood Pressure (mmHg)
Brain and nervous system cancer	Male	0-6 days	95+ years	1	Percent of total calories consumed as saturated fat
Brain and nervous system cancer	Male	0-6 days	95+ years	1	Smoking Prevalence
Brain and nervous system cancer	Male	0-6 days	95+ years	-1	Vegetables (kcal per capita)
Brain and nervous system cancer	Male	0-6 days	95+ years	0	Socio-demographic Index
Brain and nervous system cancer	Male	0-6 days	95+ years	-1	fruits adjusted (g)
Brain and nervous system cancer	Male	0-6 days	95+ years	1	red meats adjusted (g)
Brain and nervous system cancer	Male	0-6 days	95+ years	-1	vegetables adjusted (g)
Brain and nervous system cancer	Male	0-6 days	95+ years	-1	Healthcare access and quality index

© 2018 Fitzmaurice C et al. JAMA Oncology.

Cause	Sex	Age start	Age end	Direction	Covariate
Thyroid cancer	Female	10-14 years	95+ years	1	Alcohol (liters per capita)
Thyroid cancer	Female	10-14 years	95+ years	1	Tobacco (cigarettes per capita)
Thyroid cancer	Female	10-14 years	95+ years	-1	Education (years per capita)
Thyroid cancer	Female	10-14 years	95+ years	0	LDI (I$ per capita)
Thyroid cancer	Female	10-14 years	95+ years	1	Mean BMI
Thyroid cancer	Female	10-14 years	95+ years	-1	Sanitation (proportion with access)
Thyroid cancer	Female	10-14 years	95+ years	1	Smoking Prevalence
Thyroid cancer	Female	10-14 years	95+ years	-1	Improved Water Source (proportion with access)
Thyroid cancer	Female	10-14 years	95+ years	1	Log-transformed SEV scalar: Thyroid C
Thyroid cancer	Female	10-14 years	95+ years	0	Socio-demographic Index
Thyroid cancer	Female	10-14 years	95+ years	-1	fruits adjusted (g)
Thyroid cancer	Female	10-14 years	95+ years	1	red meats adjusted (g)
Thyroid cancer	Female	10-14 years	95+ years	-1	vegetables adjusted (g)
Thyroid cancer	Female	10-14 years	95+ years	-1	Healthcare access and quality index
Thyroid cancer	Male	10-14 years	95+ years	1	Alcohol (liters per capita)
Thyroid cancer	Male	10-14 years	95+ years	1	Tobacco (cigarettes per capita)
Thyroid cancer	Male	10-14 years	95+ years	-1	Education (years per capita)
Thyroid cancer	Male	10-14 years	95+ years	0	LDI (I$ per capita)
Thyroid cancer	Male	10-14 years	95+ years	1	Mean BMI
Thyroid cancer	Male	10-14 years	95+ years	-1	Sanitation (proportion with access)
Thyroid cancer	Male	10-14 years	95+ years	1	Smoking Prevalence
Thyroid cancer	Male	10-14 years	95+ years	-1	Improved Water Source (proportion with access)
Thyroid cancer	Male	10-14 years	95+ years	1	Log-transformed SEV scalar: Thyroid C
Thyroid cancer	Male	10-14 years	95+ years	0	Socio-demographic Index
Thyroid cancer	Male	10-14 years	95+ years	-1	fruits adjusted (g)
Thyroid cancer	Male	10-14 years	95+ years	1	red meats adjusted (g)
Thyroid cancer	Male	10-14 years	95+ years	-1	vegetables adjusted (g)
Thyroid cancer	Male	10-14 years	95+ years	-1	Healthcare access and quality index
Thyroid cancer	Male	10-14 years	95+ years	2	Smoking Prevalence
Mesothelioma	Female	15-19 years	95+ years	1	Asbestos production (binary)

© 2018 Fitzmaurice C et al. JAMA Oncology.

Cause	Sex	Age start	Age end	Direction	Covariate
Mesothelioma	Female	15-19 years	95+ years	1	Asbestos production (kg) per capita
Mesothelioma	Female	15-19 years	95+ years	1	Cumulative Cigarettes (5 Years)
Mesothelioma	Female	15-19 years	95+ years	-1	Education (years per capita)
Mesothelioma	Female	15-19 years	95+ years	1	Gold production (binary)
Mesothelioma	Female	15-19 years	95+ years	1	Gold production (kg) per capita
Mesothelioma	Female	15-19 years	95+ years	0	LDI (I$ per capita)
Mesothelioma	Female	15-19 years	95+ years	1	Indoor Air Pollution (All Cooking Fuels)
Mesothelioma	Female	15-19 years	95+ years	1	Elevation Over 1500m (proportion)
Mesothelioma	Female	15-19 years	95+ years	1	Elevation 500 to 1500m (proportion)
Mesothelioma	Female	15-19 years	95+ years	1	Population Density (over 1000 ppl/sqkm, proportion)
Mesothelioma	Female	15-19 years	95+ years	1	Population Over 65 (proportion)
Mesothelioma	Female	15-19 years	95+ years	1	Smoking Prevalence
Mesothelioma	Female	15-19 years	95+ years	1	Log-transformed SEV scalar: Mesothel
Mesothelioma	Female	15-19 years	95+ years	0	Socio-demographic Index
Mesothelioma	Female	15-19 years	95+ years	1	Asbestos consumption (metric tons per year per capita)
Mesothelioma	Female	15-19 years	95+ years	-1	Healthcare access and quality index
Mesothelioma	Male	15-19 years	95+ years	1	Cumulative Cigarettes (5 Years)
Mesothelioma	Male	15-19 years	95+ years	-1	Education (years per capita)
Mesothelioma	Male	15-19 years	95+ years	1	Gold production (binary)
Mesothelioma	Male	15-19 years	95+ years	1	Gold production (kg) per capita
Mesothelioma	Male	15-19 years	95+ years	0	LDI (I$ per capita)
Mesothelioma	Male	15-19 years	95+ years	1	Indoor Air Pollution (All Cooking Fuels)
Mesothelioma	Male	15-19 years	95+ years	1	Elevation Over 1500m (proportion)
Mesothelioma	Male	15-19 years	95+ years	1	Elevation 500 to 1500m (proportion)
Mesothelioma	Male	15-19 years	95+ years	1	Population Density (over 1000 ppl/sqkm, proportion)
Mesothelioma	Male	15-19 years	95+ years	1	Population Over 65 (proportion)
Mesothelioma	Male	15-19 years	95+ years	1	Smoking Prevalence
Mesothelioma	Male	15-19 years	95+ years	0	Socio-demographic Index
Mesothelioma	Male	15-19 years	95+ years	1	Asbestos consumption (metric tons per year per capita)
Mesothelioma	Male	15-19 years	95+ years	-1	Healthcare access and quality index

© 2018 Fitzmaurice C et al. JAMA Oncology.

Cause	Sex	Age start	Age end	Direction	Covariate
Hodgkin lymphoma	Male	0-6 days	95+ years	-1	Education (years per capita)
Hodgkin lymphoma	Male	0-6 days	95+ years	0	LDI (I$ per capita)
Hodgkin lymphoma	Male	0-6 days	95+ years	0	Socio-demographic Index
Hodgkin lymphoma	Male	0-6 days	95+ years	-1	Healthcare access and quality index
Hodgkin lymphoma	Female	0-6 days	95+ years	-1	Education (years per capita)
Hodgkin lymphoma	Female	0-6 days	95+ years	0	LDI (I$ per capita)
Hodgkin lymphoma	Female	0-6 days	95+ years	0	Socio-demographic Index
Hodgkin lymphoma	Female	0-6 days	95+ years	-1	Healthcare access and quality index
Non-Hodgkin lymphoma	Male	0-6 days	95+ years	1	Alcohol (liters per capita)
Non-Hodgkin lymphoma	Male	0-6 days	95+ years	1	Cumulative Cigarettes (10 Years)
Non-Hodgkin lymphoma	Male	0-6 days	95+ years	-1	Health System Access 2 (unitless)
Non-Hodgkin lymphoma	Male	0-6 days	95+ years	0	LDI (I$ per capita)
Non-Hodgkin lymphoma	Male	0-6 days	95+ years	1	Smoking Prevalence
Non-Hodgkin lymphoma	Male	0-6 days	95+ years	0	Socio-demographic Index
Non-Hodgkin lymphoma	Male	0-6 days	95+ years	-1	Health System Access (capped)
Non-Hodgkin lymphoma	Male	0-6 days	95+ years	-1	Healthcare access and quality index
Non-Hodgkin lymphoma	Female	0-6 days	95+ years	1	Alcohol (liters per capita)
Non-Hodgkin lymphoma	Female	0-6 days	95+ years	1	Cumulative Cigarettes (10 Years)
Non-Hodgkin lymphoma	Female	0-6 days	95+ years	-1	Health System Access 2 (unitless)
Non-Hodgkin lymphoma	Female	0-6 days	95+ years	0	LDI (I$ per capita)
Non-Hodgkin lymphoma	Female	0-6 days	95+ years	1	Smoking Prevalence
Non-Hodgkin lymphoma	Female	0-6 days	95+ years	0	Socio-demographic Index
Non-Hodgkin lymphoma	Female	0-6 days	95+ years	-1	Healthcare access and quality index
Non-Hodgkin lymphoma	Female	0-6 days	95+ years	0	Total Fertility Rate
Multiple myeloma	Male	15-19 years	95+ years	1	Alcohol (liters per capita)
Multiple myeloma	Male	15-19 years	95+ years	1	Tobacco (cigarettes per capita)
Multiple myeloma	Male	15-19 years	95+ years	-1	Education (years per capita)
Multiple myeloma	Male	15-19 years	95+ years	0	LDI (I$ per capita)
Multiple myeloma	Male	15-19 years	95+ years	1	Mean BMI
Multiple myeloma	Male	15-19 years	95+ years	-1	Sanitation (proportion with access)

© 2018 Fitzmaurice C et al. JAMA Oncology.

Cause	Sex	Age start	Age end	Direction	Covariate
Multiple myeloma	Male	15-19 years	95+ years	1	Smoking Prevalence
Multiple myeloma	Male	15-19 years	95+ years	-1	Improved Water Source (proportion with access)
Multiple myeloma	Male	15-19 years	95+ years	0	Socio-demographic Index
Multiple myeloma	Male	15-19 years	95+ years	-1	fruits adjusted (g)
Multiple myeloma	Male	15-19 years	95+ years	1	red meats adjusted (g)
Multiple myeloma	Male	15-19 years	95+ years	-1	vegetables adjusted (g)
Multiple myeloma	Male	15-19 years	95+ years	-1	Healthcare access and quality index
Multiple myeloma	Female	15-19 years	95+ years	1	Alcohol (liters per capita)
Multiple myeloma	Female	15-19 years	95+ years	1	Tobacco (cigarettes per capita)
Multiple myeloma	Female	15-19 years	95+ years	-1	Education (years per capita)
Multiple myeloma	Female	15-19 years	95+ years	0	LDI (I$ per capita)
Multiple myeloma	Female	15-19 years	95+ years	1	Mean BMI
Multiple myeloma	Female	15-19 years	95+ years	-1	Sanitation (proportion with access)
Multiple myeloma	Female	15-19 years	95+ years	1	Smoking Prevalence
Multiple myeloma	Female	15-19 years	95+ years	-1	Improved Water Source (proportion with access)
Multiple myeloma	Female	15-19 years	95+ years	0	Socio-demographic Index
Multiple myeloma	Female	15-19 years	95+ years	-1	fruits adjusted (g)
Multiple myeloma	Female	15-19 years	95+ years	1	red meats adjusted (g)
Multiple myeloma	Female	15-19 years	95+ years	-1	vegetables adjusted (g)
Multiple myeloma	Female	15-19 years	95+ years	-1	Healthcare access and quality index
Leukemia	Female	0-6 days	95+ years	1	Alcohol (liters per capita)
Leukemia	Female	0-6 days	95+ years	1	Tobacco (cigarettes per capita)
Leukemia	Female	0-6 days	95+ years	1	Cumulative Cigarettes (10 Years)
Leukemia	Female	0-6 days	95+ years	1	Cumulative Cigarettes (15 Years)
Leukemia	Female	0-6 days	95+ years	1	Cumulative Cigarettes (20 Years)
Leukemia	Female	0-6 days	95+ years	1	Cumulative Cigarettes (5 Years)
Leukemia	Female	0-6 days	95+ years	-1	Education (years per capita)
Leukemia	Female	0-6 days	95+ years	-1	Health System Access 2 (unitless)
Leukemia	Female	0-6 days	95+ years	0	LDI (I$ per capita)
Leukemia	Female	0-6 days	95+ years	1	Smoking Prevalence

© 2018 Fitzmaurice C et al. JAMA Oncology.

Cause	Sex	Age start	Age end	Direction	Covariate
Leukemia	Female	0-6 days	95+ years	1	Log-transformed SEV scalar: Leukemia
Leukemia	Female	0-6 days	95+ years	1	Log-transformed age-standardized SEV scalar: Leukemia
Leukemia	Female	0-6 days	95+ years	0	Socio-demographic Index
Leukemia	Male	0-6 days	95+ years	1	Alcohol (liters per capita)
Leukemia	Male	0-6 days	95+ years	1	Tobacco (cigarettes per capita)
Leukemia	Male	0-6 days	95+ years	1	Cumulative Cigarettes (10 Years)
Leukemia	Male	0-6 days	95+ years	1	Cumulative Cigarettes (15 Years)
Leukemia	Male	0-6 days	95+ years	1	Cumulative Cigarettes (20 Years)
Leukemia	Male	0-6 days	95+ years	1	Cumulative Cigarettes (5 Years)
Leukemia	Male	0-6 days	95+ years	-1	Education (years per capita)
Leukemia	Male	0-6 days	95+ years	-1	Health System Access 2 (unitless)
Leukemia	Male	0-6 days	95+ years	0	LDI (I$ per capita)
Leukemia	Male	0-6 days	95+ years	1	Smoking Prevalence
Leukemia	Male	0-6 days	95+ years	1	Log-transformed SEV scalar: Leukemia
Leukemia	Male	0-6 days	95+ years	1	Log-transformed age-standardized SEV scalar: Leukemia
Leukemia	Male	0-6 days	95+ years	0	Socio-demographic Index
Leukemia	Female	0-6 days	95+ years	-1	Health System Access (capped)
Leukemia	Female	0-6 days	95+ years	-1	Healthcare access and quality index
Other neoplasms	Male	0-6 days	95+ years	1	Tobacco (cigarettes per capita)
Other neoplasms	Male	0-6 days	95+ years	-1	Education (years per capita)
Other neoplasms	Male	0-6 days	95+ years	-1	Health System Access 2 (unitless)
Other neoplasms	Male	0-6 days	95+ years	0	LDI (I$ per capita)
Other neoplasms	Male	0-6 days	95+ years	-1	Nuts & Seeds (kcal per capita)
Other neoplasms	Male	0-6 days	95+ years	1	Smoking Prevalence
Other neoplasms	Male	0-6 days	95+ years	0	Socio-demographic Index
Other neoplasms	Male	0-6 days	95+ years	-1	fruits adjusted (g)
Other neoplasms	Male	0-6 days	95+ years	-1	PUFA adjusted (percent)
Other neoplasms	Male	0-6 days	95+ years	-1	vegetables adjusted (g)
Other neoplasms	Male	0-6 days	95+ years	-1	Healthcare access and quality index
Other neoplasms	Female	0-6 days	95+ years	1	Tobacco (cigarettes per capita)

© 2018 Fitzmaurice C et al. JAMA Oncology.

Cause	Sex	Age start	Age end	Direction	Covariate
Other neoplasms	Female	0-6 days	95+ years	-1	Education (years per capita)
Other neoplasms	Female	0-6 days	95+ years	-1	Health System Access 2 (unitless)
Other neoplasms	Female	0-6 days	95+ years	0	LDI (I$ per capita)
Other neoplasms	Female	0-6 days	95+ years	1	Smoking Prevalence
Other neoplasms	Female	0-6 days	95+ years	0	Socio-demographic Index
Other neoplasms	Female	0-6 days	95+ years	-1	fruits adjusted (g)
Other neoplasms	Female	0-6 days	95+ years	-1	nuts seeds adjusted (g)
Other neoplasms	Female	0-6 days	95+ years	-1	PUFA adjusted (percent)
Other neoplasms	Female	0-6 days	95+ years	-1	vegetables adjusted (g)
Other neoplasms	Female	0-6 days	95+ years	-1	Healthcare access and quality index
Other neoplasms	Male	0-6 days	95+ years	-1	nuts seeds adjusted (g)

eTable 9: Comparison of GBD 2015 and GBD 2016 covariates used and level of covariates

Cause	Sex	Covariate	GBD 2015			GBD 2016		
			Level 1	Level 2	Level 3	Level 1	Level 2	Level 3
Esophageal cancer	Male	Alcohol (liters per capita)	X			X		
Esophageal cancer	Male	Tobacco (cigarettes per capita)	X			X		
Esophageal cancer	Male	Education (years per capita)			X			X
Esophageal cancer	Male	Fruits (kcal per capita)	X			X		
Esophageal cancer	Male	LDI (I$ per capita)			X			X
Esophageal cancer	Male	Mean BMI	X			X		
Esophageal cancer	Male	Indoor Air Pollution (All Cooking Fuels)		X			X	
Esophageal cancer	Male	Sanitation (proportion with access)		X			X	
Esophageal cancer	Male	Smoking Prevalence	X			X		
Esophageal cancer	Male	Vegetables (kcal per capita)		X			X	
Esophageal cancer	Male	Improved Water Source (proportion with access)		X			X	
Esophageal cancer	Male	Socio-demographic Index			X		X	

© 2018 Fitzmaurice C et al. JAMA Oncology.

Cause	Sex	Covariate	GBD 2015			GBD 2016		
			Level 1	Level 2	Level 3	Level 1	Level 2	Level 3
Esophageal cancer	Female	Alcohol (liters per capita)	X			X		
Esophageal cancer	Female	Tobacco (cigarettes per capita)	X			X		
Esophageal cancer	Female	Education (years per capita)			X			X
Esophageal cancer	Female	Fruits (kcal per capita)	X			X		
Esophageal cancer	Female	LDI (I$ per capita)			X			X
Esophageal cancer	Female	Mean BMI	X			X		
Esophageal cancer	Female	Indoor Air Pollution (All Cooking Fuels)		X			X	
Esophageal cancer	Female	Sanitation (proportion with access)		X			X	
Esophageal cancer	Female	Smoking Prevalence	X			X		
Esophageal cancer	Female	Vegetables (kcal per capita)		X			X	
Esophageal cancer	Female	Improved Water Source (proportion with access)		X			X	
Esophageal cancer	Female	Socio-demographic Index			X			X
Esophageal cancer	Female	Socio-demographic Index			X		X	
Stomach cancer	Male	Alcohol (liters per capita)	X			X		
Stomach cancer	Male	Tobacco (cigarettes per capita)	X			X		
Stomach cancer	Male	Cumulative Cigarettes (10 Years)	X			X		
Stomach cancer	Male	Cumulative Cigarettes (15 Years)	X			X		
Stomach cancer	Male	LDI (I$ per capita)			X			X
Stomach cancer	Male	Mean BMI		X			X	
Stomach cancer	Male	Indoor Air Pollution (All Cooking Fuels)		X			X	
Stomach cancer	Male	Outdoor Air Pollution (PM2.5)		X			X	
Stomach cancer	Male	Sanitation (proportion with access)		X			X	
Stomach cancer	Male	Smoking Prevalence	X			X		
Stomach cancer	Male	Improved Water Source (proportion with access)		X			X	
Stomach cancer	Male	Log-transformed SEV scalar: Stomach C	X			X		

© 2018 Fitzmaurice C et al. JAMA Oncology.

Cause	Sex	Covariate	GBD 2015			GBD 2016		
			Level 1	Level 2	Level 3	Level 1	Level 2	Level 3
Stomach cancer	Male	Socio-demographic Index			X			X
Stomach cancer	Female	Alcohol (liters per capita)	X			X		
Stomach cancer	Female	Tobacco (cigarettes per capita)	X			X		
Stomach cancer	Female	Cumulative Cigarettes (10 Years)	X			X		
Stomach cancer	Female	Education (years per capita)			X			X
Stomach cancer	Female	LDI (I$ per capita)			X			X
Stomach cancer	Female	Mean BMI		X			X	
Stomach cancer	Female	Indoor Air Pollution (All Cooking Fuels)		X			X	
Stomach cancer	Female	Outdoor Air Pollution (PM2.5)		X			X	
Stomach cancer	Female	Sanitation (proportion with access)		X			X	
Stomach cancer	Female	Smoking Prevalence	X			X		
Stomach cancer	Female	Improved Water Source (proportion with access)		X			X	
Stomach cancer	Female	Log-transformed SEV scalar: Stomach C	X			X		
Stomach cancer	Female	Socio-demographic Index			X			X
Liver cancer	Male	Cumulative Cigarettes (15 Years)	X			X		
Liver cancer	Male	Tobacco (cigarettes per capita)	X			X		
Liver cancer	Male	Education (years per capita)			X			X
Liver cancer	Male	LDI (I$ per capita)			X			X
Liver cancer	Male	Cumulative Cigarettes (20 Years)	X			X		
Liver cancer	Male	Diabetes Age-Standardized Prevalence (proportion)		X			X	
Liver cancer	Male	Mean BMI		X			X	
Liver cancer	Male	Alcohol (liters per capita)	X			X		
Liver cancer	Male	Log-transformed SEV scalar: Liver C	X			X		
Liver cancer	Male	Socio-demographic Index			X			X
Liver cancer	Female	Cumulative Cigarettes (15 Years)	X			X		

© 2018 Fitzmaurice C et al. JAMA Oncology.

Cause	Sex	Covariate	GBD 2015			GBD 2016		
			Level 1	Level 2	Level 3	Level 1	Level 2	Level 3
Liver cancer	Female	Tobacco (cigarettes per capita)	X			X		
Liver cancer	Female	Education (years per capita)			X			X
Liver cancer	Female	LDI (I$ per capita)			X			X
Liver cancer	Female	Cumulative Cigarettes (20 Years)	X			X		
Liver cancer	Female	Diabetes Age-Standardized Prevalence (proportion)		X			X	
Liver cancer	Female	Mean BMI		X			X	
Liver cancer	Female	Alcohol (liters per capita)	X			X		
Liver cancer	Female	Log-transformed SEV scalar: Liver C	X			X		
Liver cancer	Female	Socio-demographic Index			X			X
Larynx cancer	Male	Cumulative Cigarettes (10 Years)		X			X	
Larynx cancer	Male	Cumulative Cigarettes (15 Years)		X			X	
Larynx cancer	Male	Cumulative Cigarettes (20 Years)		X			X	
Larynx cancer	Male	Cumulative Cigarettes (5 Years)		X			X	
Larynx cancer	Male	Tobacco (cigarettes per capita)		X			X	
Larynx cancer	Male	Education (years per capita)			X			X
Larynx cancer	Male	Log-transformed SEV scalar: Larynx C	X			X		
Larynx cancer	Male	LDI (I$ per capita)			X			X
Larynx cancer	Male	Smoking Prevalence		X			X	
Larynx cancer	Male	Alcohol (liters per capita)	X			X		
Larynx cancer	Male	Population Density (under 150 ppl/sqkm, proportion)		X			X	
Larynx cancer	Male	Population Density (over 1000 ppl/sqkm, proportion)		X			X	
Larynx cancer	Male	Socio-demographic Index			X			X
Larynx cancer	Female	Cumulative Cigarettes (10 Years)		X			X	
Larynx cancer	Female	Cumulative Cigarettes (15 Years)		X			X	
Larynx cancer	Female	Cumulative Cigarettes (20 Years)		X			X	

© 2018 Fitzmaurice C et al. JAMA Oncology.

Cause	Sex	Covariate	GBD 2015			GBD 2016		
			Level 1	Level 2	Level 3	Level 1	Level 2	Level 3
Larynx cancer	Female	Cumulative Cigarettes (5 Years)		X			X	
Larynx cancer	Female	Tobacco (cigarettes per capita)		X			X	
Larynx cancer	Female	Education (years per capita)			X			X
Larynx cancer	Female	Log-transformed SEV scalar: Larynx C	X			X		
Larynx cancer	Female	LDI (I$ per capita)			X			X
Larynx cancer	Female	Smoking Prevalence		X			X	
Larynx cancer	Female	Alcohol (liters per capita)	X			X		
Larynx cancer	Female	Population Density (under 150 ppl/sqkm, proportion)		X			X	
Larynx cancer	Female	Population Density (over 1000 ppl/sqkm, proportion)		X			X	
Larynx cancer	Female	Socio-demographic Index			X			X
Tracheal, bronchus, and lung cancer	Male	Tobacco (cigarettes per capita)	X			X		
Tracheal, bronchus, and lung cancer	Male	Cumulative Cigarettes (10 Years)	X			X		
Tracheal, bronchus, and lung cancer	Male	Cumulative Cigarettes (15 Years)	X			X		
Tracheal, bronchus, and lung cancer	Male	Cumulative Cigarettes (20 Years)	X			X		
Tracheal, bronchus, and lung cancer	Male	Cumulative Cigarettes (5 Years)	X			X		
Tracheal, bronchus, and lung cancer	Male	Education (years per capita)			X			X
Tracheal, bronchus, and lung cancer	Male	LDI (I$ per capita)			X			X
Tracheal, bronchus, and lung cancer	Male	Indoor Air Pollution (All Cooking Fuels)		X			X	
Tracheal, bronchus, and	Male	Outdoor Air Pollution (PM2.5)		X			X	

© 2018 Fitzmaurice C et al. JAMA Oncology.

Cause	Sex	Covariate	GBD 2015			GBD 2016		
			Level 1	Level 2	Level 3	Level 1	Level 2	Level 3
lung cancer								
Tracheal, bronchus, and lung cancer	Male	Smoking Prevalence	X			X		
Tracheal, bronchus, and lung cancer	Male	Socio-demographic Index			X			X
Tracheal, bronchus, and lung cancer	Female	Tobacco (cigarettes per capita)	X			X		
Tracheal, bronchus, and lung cancer	Female	Cumulative Cigarettes (10 Years)	X			X		
Tracheal, bronchus, and lung cancer	Female	Cumulative Cigarettes (15 Years)	X			X		
Tracheal, bronchus, and lung cancer	Female	Cumulative Cigarettes (20 Years)	X			X		
Tracheal, bronchus, and lung cancer	Female	Cumulative Cigarettes (5 Years)	X			X		
Tracheal, bronchus, and lung cancer	Female	Education (years per capita)			X			X
Tracheal, bronchus, and lung cancer	Female	LDI (I$ per capita)			X			X
Tracheal, bronchus, and lung cancer	Female	Indoor Air Pollution (All Cooking Fuels)		X			X	
Tracheal, bronchus, and lung cancer	Female	Outdoor Air Pollution (PM2.5)		X			X	
Tracheal, bronchus, and lung cancer	Female	Smoking Prevalence	X			X		
Tracheal, bronchus, and lung cancer	Female	Socio-demographic Index			X			X
Breast cancer	Male	Cumulative Cigarettes (10 Years)		X			X	
Breast cancer	Male	Alcohol (liters per capita)	X			X		
Breast cancer	Male	Education (years per capita)			X			X

© 2018 Fitzmaurice C et al. JAMA Oncology.

Cause	Sex	Covariate	GBD 2015			GBD 2016		
			Level 1	Level 2	Level 3	Level 1	Level 2	Level 3
Breast cancer	Male	LDI (I$ per capita)			X			X
Breast cancer	Male	Log-transformed SEV scalar: Breast C	X			X		
Breast cancer	Male	Socio-demographic Index			X			X
Breast cancer	Male	Mean BMI	X			X		
Breast cancer	Female	Cumulative Cigarettes (10 Years)		X			X	
Breast cancer	Female	Alcohol (liters per capita)	X			X		
Breast cancer	Female	Education (years per capita)			X			X
Breast cancer	Female	LDI (I$ per capita)			X			X
Breast cancer	Female	Log-transformed SEV scalar: Breast C	X			X		
Breast cancer	Female	Socio-demographic Index			X			X
Breast cancer	Female	Mean BMI	X			X		
Breast cancer	Female	Total Fertility Rate		X			X	
Cervical cancer	Female	Abortion On-Demand Illegal (binary)	X			X		
Cervical cancer	Female	Cumulative Cigarettes (10 Years)	X			X		
Cervical cancer	Female	Cumulative Cigarettes (15 Years)	X			X		
Cervical cancer	Female	Cumulative Cigarettes (5 Years)	X			X		
Cervical cancer	Female	Education (years per capita)			X			X
Cervical cancer	Female	Health System Access 2 (unitless)		X			X	
Cervical cancer	Female	LDI (I$ per capita)			X			X
Cervical cancer	Female	Smoking Prevalence		X			X	
Cervical cancer	Female	Total Fertility Rate		X			X	
Cervical cancer	Female	Socio-demographic Index			X			X
Cervical cancer	Female	HIV age-standardized prevalence	X			X		
Uterine cancer	Female	Cumulative Cigarettes (10 Years)		X			X	
Uterine cancer	Female	Cumulative Cigarettes (5 Years)		X			X	
Uterine cancer	Female	Tobacco (cigarettes per capita)		X			X	
Uterine cancer	Female	Education (years per capita)			X			X

© 2018 Fitzmaurice C et al. JAMA Oncology.

Cause	Sex	Covariate	GBD 2015			GBD 2016		
			Level 1	Level 2	Level 3	Level 1	Level 2	Level 3
Uterine cancer	Female	Health System Access (unitless)		X			X	
Uterine cancer	Female	Diabetes Age-Standardized Prevalence (proportion)		X			X	
Uterine cancer	Female	Smoking Prevalence		X			X	
Uterine cancer	Female	Mean BMI	X			X		
Uterine cancer	Female	Total Fertility Rate		X			X	
Uterine cancer	Female	Socio-demographic Index			X			X
Uterine cancer	Female	LDI (I$ per capita)			X			X
Uterine cancer	Female	Log-transformed SEV scalar: Uterus C	X			X		
Prostate cancer	Male	Socio-demographic Index			X			X
Prostate cancer	Male	Log-transformed SEV scalar: Prostate C	X			X		
Prostate cancer	Male	Education (years per capita)			X			X
Prostate cancer	Male	LDI (I$ per capita)			X			X
Colon and rectum cancer	Male	Alcohol (liters per capita)	X			X		
Colon and rectum cancer	Male	Tobacco (cigarettes per capita)	X			X		
Colon and rectum cancer	Male	Diabetes Age-Standardized Prevalence (proportion)		X			X	
Colon and rectum cancer	Male	Education (years per capita)			X			X
Colon and rectum cancer	Male	Health System Access 2 (unitless)		X			X	
Colon and rectum cancer	Male	LDI (I$ per capita)			X			X
Colon and rectum cancer	Male	Mean BMI	X			X		
Colon and rectum cancer	Male	Smoking Prevalence	X			X		
Colon and rectum cancer	Male	Log-transformed SEV scalar: Colorect C	X			X		
Colon and rectum cancer	Male	Socio-demographic Index			X			X
Colon and rectum cancer	Female	Alcohol (liters per capita)	X			X		
Colon and rectum cancer	Female	Tobacco (cigarettes per capita)		X			X	
Colon and rectum cancer	Female	Diabetes Age-Standardized Prevalence (proportion)		X			X	

© 2018 Fitzmaurice C et al. JAMA Oncology.

Cause	Sex	Covariate	GBD 2015			GBD 2016		
			Level 1	Level 2	Level 3	Level 1	Level 2	Level 3
Colon and rectum cancer	Female	Education (years per capita)			X			X
Colon and rectum cancer	Female	Health System Access 2 (unitless)		X			X	
Colon and rectum cancer	Female	LDI (I$ per capita)			X			X
Colon and rectum cancer	Female	Mean BMI	X			X		
Colon and rectum cancer	Female	Smoking Prevalence		X			X	
Colon and rectum cancer	Female	Log-transformed SEV scalar: Colorect C	X			X		
Colon and rectum cancer	Female	Socio-demographic Index			X			X
Lip and oral cavity cancer	Male	Alcohol (liters per capita)	X			X		
Lip and oral cavity cancer	Male	Cumulative Cigarettes (10 Years)	X			X		
Lip and oral cavity cancer	Male	Cumulative Cigarettes (20 Years)	X			X		
Lip and oral cavity cancer	Male	Education (years per capita)			X			X
Lip and oral cavity cancer	Male	Health System Access 2 (unitless)		X			X	
Lip and oral cavity cancer	Male	LDI (I$ per capita)			X			X
Lip and oral cavity cancer	Male	Smoking Prevalence	X			X		
Lip and oral cavity cancer	Male	Log-transformed SEV scalar: Mouth C	X			X		
Lip and oral cavity cancer	Male	Socio-demographic Index			X			X
Lip and oral cavity cancer	Female	Alcohol (liters per capita)	X			X		
Lip and oral cavity cancer	Female	Cumulative Cigarettes (10 Years)	X			X		
Lip and oral cavity cancer	Female	Cumulative Cigarettes (20 Years)	X			X		
Lip and oral cavity cancer	Female	Education (years per capita)			X			X
Lip and oral cavity cancer	Female	Fruits (kcal per capita)		X			X	
Lip and oral cavity cancer	Female	Health System Access 2 (unitless)		X			X	
Lip and oral cavity cancer	Female	LDI (I$ per capita)			X			X
Lip and oral cavity cancer	Female	Red Meat (kcal per capita)		X			X	
Lip and oral cavity cancer	Female	Smoking Prevalence	X			X		
Lip and oral cavity cancer	Female	Vegetables (kcal per capita)	X			X		
Lip and oral cavity cancer	Female	Socio-demographic Index			X			X

© 2018 Fitzmaurice C et al. JAMA Oncology.

Cause	Sex	Covariate	GBD 2015			GBD 2016		
			Level 1	Level 2	Level 3	Level 1	Level 2	Level 3
Nasopharynx cancer	Male	Alcohol (liters per capita)	X			X		
Nasopharynx cancer	Male	Education (years per capita)			X			X
Nasopharynx cancer	Male	Health System Access 2 (unitless)		X			X	
Nasopharynx cancer	Male	LDI (I$ per capita)			X			X
Nasopharynx cancer	Male	Population Density (over 1000 ppl/sqkm, proportion)		X			X	
Nasopharynx cancer	Male	Population Density (under 150 ppl/sqkm, proportion)		X			X	
Nasopharynx cancer	Male	Log-transformed SEV scalar: Nasoph C	X			X		
Nasopharynx cancer	Male	Socio-demographic Index			X			X
Nasopharynx cancer	Female	Alcohol (liters per capita)	X			X		
Nasopharynx cancer	Female	Education (years per capita)			X			X
Nasopharynx cancer	Female	Health System Access 2 (unitless)		X			X	
Nasopharynx cancer	Female	LDI (I$ per capita)			X			X
Nasopharynx cancer	Female	Population Density (over 1000 ppl/sqkm, proportion)		X			X	
Nasopharynx cancer	Female	Population Density (under 150 ppl/sqkm, proportion)		X			X	
Nasopharynx cancer	Female	Log-transformed SEV scalar: Nasoph C	X			X		
Nasopharynx cancer	Female	Socio-demographic Index			X			X
Other pharynx cancer	Male	Alcohol (liters per capita)	X			X		
Other pharynx cancer	Male	Cumulative Cigarettes (5 Years)		X			X	
Other pharynx cancer	Male	Education (years per capita)			X			X
Other pharynx cancer	Male	LDI (I$ per capita)			X			X
Other pharynx cancer	Male	Population Density (over 1000 ppl/sqkm, proportion)		X			X	
Other pharynx cancer	Male	Population Density (under 150 ppl/sqkm, proportion)		X			X	

© 2018 Fitzmaurice C et al. JAMA Oncology.

Cause	Sex	Covariate	GBD 2015			GBD 2016		
			Level 1	Level 2	Level 3	Level 1	Level 2	Level 3
Other pharynx cancer	Male	Smoking Prevalence	X			X		
Other pharynx cancer	Male	Health System Access (capped)		X			X	
Other pharynx cancer	Male	Log-transformed SEV scalar: Oth Phar C	X			X		
Other pharynx cancer	Male	Socio-demographic Index			X			X
Other pharynx cancer	Female	Alcohol (liters per capita)	X			X		
Other pharynx cancer	Female	Cumulative Cigarettes (5 Years)		X			X	
Other pharynx cancer	Female	Education (years per capita)			X			X
Other pharynx cancer	Female	LDI (I$ per capita)			X			X
Other pharynx cancer	Female	Population Density (over 1000 ppl/sqkm, proportion)		X			X	
Other pharynx cancer	Female	Population Density (under 150 ppl/sqkm, proportion)		X			X	
Other pharynx cancer	Female	Smoking Prevalence	X			X		
Other pharynx cancer	Female	Health System Access (capped)		X			X	
Other pharynx cancer	Female	Log-transformed SEV scalar: Oth Phar C	X			X		
Other pharynx cancer	Female	Socio-demographic Index			X			X
Gallbladder and biliary tract cancer	Female	Cumulative Cigarettes (10 Years)		X			X	
Gallbladder and biliary tract cancer	Female	Cumulative Cigarettes (5 Years)		X			X	
Gallbladder and biliary tract cancer	Female	Tobacco (cigarettes per capita)		X			X	
Gallbladder and biliary tract cancer	Female	Education (years per capita)			X			X
Gallbladder and biliary tract cancer	Female	Health System Access (capped)		X			X	
Gallbladder and biliary tract cancer	Female	LDI (I$ per capita)			X			X
Gallbladder and biliary tract	Female	Log-transformed SEV scalar: Gallblad C	X			X		

© 2018 Fitzmaurice C et al. JAMA Oncology.

Cause	Sex	Covariate	GBD 2015			GBD 2016		
			Level 1	Level 2	Level 3	Level 1	Level 2	Level 3
cancer								
Gallbladder and biliary tract cancer	Female	Diabetes Age-Standardized Prevalence (proportion)		X			X	
Gallbladder and biliary tract cancer	Female	Smoking Prevalence		X			X	
Gallbladder and biliary tract cancer	Female	Alcohol (liters per capita)		X			X	
Gallbladder and biliary tract cancer	Female	Mean BMI	X			X		
Gallbladder and biliary tract cancer	Female	Socio-demographic Index			X			X
Gallbladder and biliary tract cancer	Male	Cumulative Cigarettes (10 Years)		X			X	
Gallbladder and biliary tract cancer	Male	Cumulative Cigarettes (5 Years)		X			X	
Gallbladder and biliary tract cancer	Male	Tobacco (cigarettes per capita)		X			X	
Gallbladder and biliary tract cancer	Male	Education (years per capita)			X			X
Gallbladder and biliary tract cancer	Male	Health System Access (capped)		X			X	
Gallbladder and biliary tract cancer	Male	LDI (I$ per capita)			X			X
Gallbladder and biliary tract cancer	Male	Log-transformed SEV scalar: Gallblad C	X			X		
Gallbladder and biliary tract cancer	Male	Diabetes Age-Standardized Prevalence (proportion)		X			X	
Gallbladder and biliary tract cancer	Male	Smoking Prevalence		X			X	
Gallbladder and biliary tract	Male	Alcohol (liters per capita)		X			X	

© 2018 Fitzmaurice C et al. JAMA Oncology.

Cause	Sex	Covariate	GBD 2015			GBD 2016		
			Level 1	Level 2	Level 3	Level 1	Level 2	Level 3
cancer								
Gallbladder and biliary tract cancer	Male	Mean BMI	X			X		
Gallbladder and biliary tract cancer	Male	Socio-demographic Index			X			X
Pancreatic cancer	Male	Cumulative Cigarettes (10 Years)	X			X		
Pancreatic cancer	Male	Total Calories (kcal per capita)		X			X	
Pancreatic cancer	Male	Cumulative Cigarettes (20 Years)	X			X		
Pancreatic cancer	Male	Cumulative Cigarettes (5 Years)		X			X	
Pancreatic cancer	Male	Tobacco (cigarettes per capita)	X			X		
Pancreatic cancer	Male	Education (years per capita)			X			X
Pancreatic cancer	Male	Log-transformed SEV scalar: Pancreas C	X			X		
Pancreatic cancer	Male	Diabetes Age-Standardized Prevalence (proportion)		X			X	
Pancreatic cancer	Male	Smoking Prevalence	X			X		
Pancreatic cancer	Male	Alcohol (liters per capita)	X			X		
Pancreatic cancer	Male	Mean BMI	X			X		
Pancreatic cancer	Male	Socio-demographic Index			X			X
Pancreatic cancer	Male	LDI (I$ per capita)			X			X
Pancreatic cancer	Female	Cumulative Cigarettes (10 Years)	X			X		
Pancreatic cancer	Female	Cumulative Cigarettes (20 Years)	X			X		
Pancreatic cancer	Female	Cumulative Cigarettes (5 Years)	X			X		
Pancreatic cancer	Female	Tobacco (cigarettes per capita)	X			X		
Pancreatic cancer	Female	Education (years per capita)			X			X
Pancreatic cancer	Female	Log-transformed SEV scalar: Pancreas C	X			X		
Pancreatic cancer	Female	Diabetes Age-Standardized Prevalence (proportion)		X			X	
Pancreatic cancer	Female	Smoking Prevalence	X			X		

© 2018 Fitzmaurice C et al. JAMA Oncology.

Cause	Sex	Covariate	GBD 2015			GBD 2016		
			Level 1	Level 2	Level 3	Level 1	Level 2	Level 3
Pancreatic cancer	Female	Alcohol (liters per capita)		X			X	
Pancreatic cancer	Female	Mean BMI	X			X		
Pancreatic cancer	Female	Socio-demographic Index			X			X
Pancreatic cancer	Female	LDI (I$ per capita)			X			X
Malignant skin melanoma	Male	Education (years per capita)			X			X
Malignant skin melanoma	Male	LDI (I$ per capita)			X			X
Malignant skin melanoma	Male	Alcohol (liters per capita)	X				X	
Malignant skin melanoma	Male	Alcohol (liters per capita)	X			X		
Malignant skin melanoma	Male	Latitude 15 to 30 (proportion)		X			X	
Malignant skin melanoma	Male	Latitude Over 45 (proportion)		X			X	
Malignant skin melanoma	Male	Latitude 30 to 45 (proportion)		X			X	
Malignant skin melanoma	Male	Socio-demographic Index			X			X
Malignant skin melanoma	Female	Education (years per capita)			X			X
Malignant skin melanoma	Female	LDI (I$ per capita)			X			X
Malignant skin melanoma	Female	Alcohol (liters per capita)	X			X		
Malignant skin melanoma	Female	Latitude 15 to 30 (proportion)		X			X	
Malignant skin melanoma	Female	Latitude Over 45 (proportion)		X			X	
Malignant skin melanoma	Female	Latitude 30 to 45 (proportion)		X			X	
Malignant skin melanoma	Female	Socio-demographic Index			X			X
Non-melanoma skin cancer	Female	Cumulative Cigarettes (10 Years)	X			X		
Non-melanoma skin cancer	Female	Cumulative Cigarettes (15 Years)	X			X		
Non-melanoma skin cancer	Female	Socio-demographic Index			X			X
Non-melanoma skin cancer	Female	Cumulative Cigarettes (5 Years)	X			X		
Non-melanoma skin cancer	Female	Education (years per capita)			X			X
Non-melanoma skin cancer	Female	Health System Access (capped)	X				X	
Non-melanoma skin cancer	Female	LDI (I$ per capita)			X			X
Non-melanoma skin cancer	Female	Average latitude		X			X	

© 2018 Fitzmaurice C et al. JAMA Oncology.

Cause	Sex	Covariate	GBD 2015			GBD 2016		
			Level 1	Level 2	Level 3	Level 1	Level 2	Level 3
Non-melanoma skin cancer	Female	Smoking Prevalence	X			X		
Non-melanoma skin cancer	Male	Cumulative Cigarettes (10 Years)	X			X		
Non-melanoma skin cancer	Male	Cumulative Cigarettes (15 Years)	X			X		
Non-melanoma skin cancer	Male	Socio-demographic Index			X			X
Non-melanoma skin cancer	Male	Cumulative Cigarettes (5 Years)	X			X		
Non-melanoma skin cancer	Male	Education (years per capita)			X			X
Non-melanoma skin cancer	Male	Health System Access (capped)	X				X	
Non-melanoma skin cancer	Male	LDI (I$ per capita)			X			X
Non-melanoma skin cancer	Male	Average latitude		X			X	
Non-melanoma skin cancer	Male	Smoking Prevalence	X			X		
Ovarian cancer	Female	Cumulative Cigarettes (20 Years)	X			X		
Ovarian cancer	Female	Smoking Prevalence		X			X	
Ovarian cancer	Female	Tobacco (cigarettes per capita)	X			X		
Ovarian cancer	Female	Contraception (Modern) Prevalence (proportion)	X			X		
Ovarian cancer	Female	LDI (I$ per capita)			X			X
Ovarian cancer	Female	Alcohol (liters per capita)	X			X		
Ovarian cancer	Female	Diabetes Age-Standardized Prevalence (proportion)		X			X	
Ovarian cancer	Female	Log-transformed SEV scalar: Ovary C	X			X		
Ovarian cancer	Female	Education (years per capita)			X			X
Ovarian cancer	Female	Mean BMI		X			X	
Ovarian cancer	Female	Total Fertility Rate		X			X	
Ovarian cancer	Female	Socio-demographic Index			X			X
Testicular cancer	Male	Cumulative Cigarettes (10 Years)		X			X	
Testicular cancer	Male	Cumulative Cigarettes (15 Years)		X			X	
Testicular cancer	Male	Cumulative Cigarettes (5 Years)		X			X	

© 2018 Fitzmaurice C et al. JAMA Oncology.

Cause	Sex	Covariate	GBD 2015			GBD 2016		
			Level 1	Level 2	Level 3	Level 1	Level 2	Level 3
Testicular cancer	Male	Education (years per capita)			X			X
Testicular cancer	Male	Fruits (kcal per capita)		X			X	
Testicular cancer	Male	Health System Access 2 (unitless)		X			X	
Testicular cancer	Male	LDI (I$ per capita)			X			X
Testicular cancer	Male	Vegetables (kcal per capita)		X			X	
Testicular cancer	Male	Socio-demographic Index			X			X
Kidney cancer	Male	Cumulative Cigarettes (10 Years)	X			X		
Kidney cancer	Male	Cumulative Cigarettes (15 Years)	X			X		
Kidney cancer	Male	Socio-demographic Index			X			X
Kidney cancer	Male	Cumulative Cigarettes (5 Years)	X			X		
Kidney cancer	Male	Alcohol (liters per capita)		X			X	
Kidney cancer	Male	Education (years per capita)			X			X
Kidney cancer	Male	LDI (I$ per capita)			X			X
Kidney cancer	Male	Health System Access 2 (unitless)		X			X	
Kidney cancer	Male	Diabetes Age-Standardized Prevalence (proportion)		X			X	
Kidney cancer	Male	Smoking Prevalence		X			X	
Kidney cancer	Male	Systolic Blood Pressure (mmHg)		X			X	
Kidney cancer	Male	Mean BMI	X			X		
Kidney cancer	Male	Log-transformed SEV scalar: Kidney C	X			X		
Kidney cancer	Female	Cumulative Cigarettes (10 Years)	X			X		
Kidney cancer	Female	Cumulative Cigarettes (15 Years)	X			X		
Kidney cancer	Female	Socio-demographic Index			X			X
Kidney cancer	Female	Cumulative Cigarettes (5 Years)	X			X		
Kidney cancer	Female	Alcohol (liters per capita)		X			X	
Kidney cancer	Female	Total Calories (kcal per capita)		X			X	
Kidney cancer	Female	Education (years per capita)			X			X

© 2018 Fitzmaurice C et al. JAMA Oncology.

Cause	Sex	Covariate	GBD 2015			GBD 2016		
			Level 1	Level 2	Level 3	Level 1	Level 2	Level 3
Kidney cancer	Female	LDI (I$ per capita)			X			X
Kidney cancer	Female	Health System Access 2 (unitless)		X			X	
Kidney cancer	Female	Diabetes Age-Standardized Prevalence (proportion)		X			X	
Kidney cancer	Female	Total Fertility Rate			X			X
Kidney cancer	Female	Smoking Prevalence		X			X	
Kidney cancer	Female	Systolic Blood Pressure (mmHg)		X			X	
Kidney cancer	Female	Mean BMI	X			X		
Kidney cancer	Female	Log-transformed SEV scalar: Kidney C	X			X		
Bladder cancer	Male	Cumulative Cigarettes (10 Years)	X			X		
Bladder cancer	Male	Cumulative Cigarettes (15 Years)	X			X		
Bladder cancer	Male	Cumulative Cigarettes (5 Years)	X			X		
Bladder cancer	Male	Education (years per capita)			X			X
Bladder cancer	Male	LDI (I$ per capita)			X			X
Bladder cancer	Male	Socio-demographic Index			X			X
Bladder cancer	Male	Smoking Prevalence	X			X		
Bladder cancer	Male	Alcohol (liters per capita)		X			X	
Bladder cancer	Male	Log-transformed SEV scalar: Bladder C	X			X		
Bladder cancer	Male	Population Density (under 150 ppl/sqkm, proportion)		X			X	
Bladder cancer	Male	Population Density (over 1000 ppl/sqkm, proportion)		X			X	
Bladder cancer	Female	Cumulative Cigarettes (10 Years)	X			X		
Bladder cancer	Female	Cumulative Cigarettes (15 Years)	X			X		
Bladder cancer	Female	Cumulative Cigarettes (5 Years)	X			X		
Bladder cancer	Female	Education (years per capita)			X			X
Bladder cancer	Female	LDI (I$ per capita)			X			X
Bladder cancer	Female	Socio-demographic Index			X			X

© 2018 Fitzmaurice C et al. JAMA Oncology.

Cause	Sex	Covariate	GBD 2015			GBD 2016		
			Level 1	Level 2	Level 3	Level 1	Level 2	Level 3
Bladder cancer	Female	Smoking Prevalence	X			X		
Bladder cancer	Female	Alcohol (liters per capita)		X			X	
Bladder cancer	Female	Log-transformed SEV scalar: Bladder C	X			X		
Bladder cancer	Female	Population Density (under 150 ppl/sqkm, proportion)		X			X	
Bladder cancer	Female	Population Density (over 1000 ppl/sqkm, proportion)		X			X	
Brain and nervous system cancer	Male	Cumulative Cigarettes (10 Years)	X			X		
Brain and nervous system cancer	Male	Cumulative Cigarettes (15 Years)	X			X		
Brain and nervous system cancer	Male	Vegetables (kcal per capita)		X			X	
Brain and nervous system cancer	Male	Smoking Prevalence	X			X		
Brain and nervous system cancer	Male	Education (years per capita)			X			X
Brain and nervous system cancer	Male	LDI (I$ per capita)			X			X
Brain and nervous system cancer	Male	Alcohol (liters per capita)	X			X		
Brain and nervous system cancer	Male	Systolic Blood Pressure (mmHg)		X			X	
Brain and nervous system cancer	Male	Cholesterol (total, mean per capita)		X			X	
Brain and nervous system cancer	Male	Socio-demographic Index			X			X
Brain and nervous system cancer	Female	Cumulative Cigarettes (10 Years)	X			X		
Brain and nervous system	Female	Cumulative Cigarettes (15 Years)	X			X		

© 2018 Fitzmaurice C et al. JAMA Oncology.

Cause	Sex	Covariate	GBD 2015			GBD 2016		
			Level 1	Level 2	Level 3	Level 1	Level 2	Level 3
cancer								
Brain and nervous system cancer	Female	Smoking Prevalence	X			X		
Brain and nervous system cancer	Female	Education (years per capita)			X			X
Brain and nervous system cancer	Female	LDI (I$ per capita)			X			X
Brain and nervous system cancer	Female	Alcohol (liters per capita)	X			X		
Brain and nervous system cancer	Female	Systolic Blood Pressure (mmHg)		X			X	
Brain and nervous system cancer	Female	Cholesterol (total, mean per capita)		X			X	
Brain and nervous system cancer	Female	Socio-demographic Index			X			X
Thyroid cancer	Male	Smoking Prevalence	X				X	
Thyroid cancer	Male	Smoking Prevalence	X			X		
Thyroid cancer	Male	Sanitation (proportion with access)		X			X	
Thyroid cancer	Male	Tobacco (cigarettes per capita)	X				X	
Thyroid cancer	Male	Education (years per capita)			X			X
Thyroid cancer	Male	Log-transformed SEV scalar: Thyroid C	X			X		
Thyroid cancer	Male	LDI (I$ per capita)			X			X
Thyroid cancer	Male	Socio-demographic Index			X			X
Thyroid cancer	Male	Alcohol (liters per capita)	X			X		
Thyroid cancer	Male	Mean BMI		X			X	
Thyroid cancer	Male	Improved Water Source (proportion with access)		X			X	
Thyroid cancer	Female	Smoking Prevalence	X				X	
Thyroid cancer	Female	Sanitation (proportion with access)		X			X	

© 2018 Fitzmaurice C et al. JAMA Oncology.

Cause	Sex	Covariate	GBD 2015			GBD 2016		
			Level 1	Level 2	Level 3	Level 1	Level 2	Level 3
Thyroid cancer	Female	Tobacco (cigarettes per capita)	X				X	
Thyroid cancer	Female	Education (years per capita)			X			X
Thyroid cancer	Female	Log-transformed SEV scalar: Thyroid C	X			X		
Thyroid cancer	Female	LDI (I$ per capita)			X			X
Thyroid cancer	Female	Socio-demographic Index			X			X
Thyroid cancer	Female	Alcohol (liters per capita)	X			X		
Thyroid cancer	Female	Mean BMI		X			X	
Thyroid cancer	Female	Improved Water Source (proportion with access)		X			X	
Mesothelioma	Male	Cumulative Cigarettes (5 Years)	X			X		
Mesothelioma	Male	Education (years per capita)			X			X
Mesothelioma	Male	Gold production (binary)		X			X	
Mesothelioma	Male	Gold production (kg) per capita		X			X	
Mesothelioma	Male	LDI (I$ per capita)			X			X
Mesothelioma	Male	Elevation Over 1500m (proportion)		X			X	
Mesothelioma	Male	Elevation 500 to 1500m (proportion)		X			X	
Mesothelioma	Male	Population Density (over 1000 ppl/sqkm, proportion)		X			X	
Mesothelioma	Male	Population Over 65 (proportion)		X			X	
Mesothelioma	Male	Smoking Prevalence	X			X		
Mesothelioma	Male	Socio-demographic Index			X			X
Mesothelioma	Female	Asbestos production (binary)	X			X		
Mesothelioma	Female	Asbestos production (kg) per capita		X			X	
Mesothelioma	Female	Cumulative Cigarettes (5 Years)	X			X		
Mesothelioma	Female	Education (years per capita)			X			X
Mesothelioma	Female	Gold production (binary)		X			X	
Mesothelioma	Female	Gold production (kg) per capita		X			X	

© 2018 Fitzmaurice C et al. JAMA Oncology.

Cause	Sex	Covariate	GBD 2015			GBD 2016		
			Level 1	Level 2	Level 3	Level 1	Level 2	Level 3
Mesothelioma	Female	LDI (I$ per capita)			X			X
Mesothelioma	Female	Elevation Over 1500m (proportion)		X			X	
Mesothelioma	Female	Elevation 500 to 1500m (proportion)		X			X	
Mesothelioma	Female	Population Density (over 1000 ppl/sqkm, proportion)		X			X	
Mesothelioma	Female	Population Over 65 (proportion)		X			X	
Mesothelioma	Female	Smoking Prevalence	X			X		
Mesothelioma	Female	Log-transformed SEV scalar: Mesothel	X			X		
Mesothelioma	Female	Socio-demographic Index			X			X
Hodgkin lymphoma	Male	Education (years per capita)			X			X
Hodgkin lymphoma	Male	LDI (I$ per capita)			X			X
Hodgkin lymphoma	Male	Socio-demographic Index			X			X
Hodgkin lymphoma	Female	Education (years per capita)			X			X
Hodgkin lymphoma	Female	LDI (I$ per capita)			X			X
Hodgkin lymphoma	Female	Socio-demographic Index			X			X
Non-Hodgkin lymphoma	Female	Cumulative Cigarettes (10 Years)		X			X	
Non-Hodgkin lymphoma	Female	Socio-demographic Index			X			X
Non-Hodgkin lymphoma	Female	Smoking Prevalence		X			X	
Non-Hodgkin lymphoma	Female	LDI (I$ per capita)			X			X
Non-Hodgkin lymphoma	Female	Health System Access 2 (unitless)	X				X	
Non-Hodgkin lymphoma	Female	Total Fertility Rate			X			X
Non-Hodgkin lymphoma	Female	Alcohol (liters per capita)		X			X	
Non-Hodgkin lymphoma	Male	Cumulative Cigarettes (10 Years)		X			X	
Non-Hodgkin lymphoma	Male	Socio-demographic Index			X			X
Non-Hodgkin lymphoma	Male	Smoking Prevalence		X			X	
Non-Hodgkin lymphoma	Male	LDI (I$ per capita)			X			X
Non-Hodgkin lymphoma	Male	Health System Access 2 (unitless)	X			X		

© 2018 Fitzmaurice C et al. JAMA Oncology.

Cause	Sex	Covariate	GBD 2015			GBD 2016		
			Level 1	Level 2	Level 3	Level 1	Level 2	Level 3
Non-Hodgkin lymphoma	Male	Alcohol (liters per capita)		X			X	
Multiple myeloma	Female	Smoking Prevalence	X			X		
Multiple myeloma	Female	Sanitation (proportion with access)		X			X	
Multiple myeloma	Female	Tobacco (cigarettes per capita)	X			X		
Multiple myeloma	Female	Education (years per capita)			X			X
Multiple myeloma	Female	LDI (I$ per capita)			X			X
Multiple myeloma	Female	Socio-demographic Index			X			X
Multiple myeloma	Female	Alcohol (liters per capita)	X			X		
Multiple myeloma	Female	Mean BMI		X			X	
Multiple myeloma	Female	Improved Water Source (proportion with access)		X			X	
Multiple myeloma	Male	Smoking Prevalence	X			X		
Multiple myeloma	Male	Sanitation (proportion with access)		X			X	
Multiple myeloma	Male	Tobacco (cigarettes per capita)	X			X		
Multiple myeloma	Male	Education (years per capita)			X			X
Multiple myeloma	Male	LDI (I$ per capita)			X			X
Multiple myeloma	Male	Socio-demographic Index			X			X
Multiple myeloma	Male	Alcohol (liters per capita)	X			X		
Multiple myeloma	Male	Mean BMI		X			X	
Multiple myeloma	Male	Improved Water Source (proportion with access)		X			X	
Leukemia	Male	Cumulative Cigarettes (10 Years)	X				X	
Leukemia	Male	Cumulative Cigarettes (15 Years)	X				X	
Leukemia	Male	Socio-demographic Index			X			X
Leukemia	Male	Cumulative Cigarettes (5 Years)	X				X	
Leukemia	Male	Education (years per capita)			X			X
Leukemia	Male	LDI (I$ per capita)			X			X

© 2018 Fitzmaurice C et al. JAMA Oncology.

Cause	Sex	Covariate	GBD 2015			GBD 2016		
			Level 1	Level 2	Level 3	Level 1	Level 2	Level 3
Leukemia	Male	Health System Access 2 (unitless)	X				X	
Leukemia	Male	Log-transformed SEV scalar: Leukemia	X			X		
Leukemia	Male	Smoking Prevalence	X				X	
Leukemia	Male	Alcohol (liters per capita)		X			X	
Leukemia	Female	Alcohol (liters per capita)		X			X	
Leukemia	Female	Cumulative Cigarettes (10 Years)	X				X	
Leukemia	Female	Cumulative Cigarettes (15 Years)	X				X	
Leukemia	Female	Education (years per capita)			X			X
Leukemia	Female	Health System Access 2 (unitless)	X				X	
Leukemia	Female	Health System Access 2 (unitless)	X			X		
Leukemia	Female	LDI (I$ per capita)			X			X
Leukemia	Female	Smoking Prevalence	X				X	
Leukemia	Female	Log-transformed SEV scalar: Leukemia	X			X		
Leukemia	Female	Socio-demographic Index			X			X
Acute lymphoid leukemia	Male	Cumulative Cigarettes (10 Years)	X				X	
Acute lymphoid leukemia	Male	Socio-demographic Index			X			X
Acute lymphoid leukemia	Male	Cumulative Cigarettes (5 Years)	X				X	
Acute lymphoid leukemia	Male	Education (years per capita)			X			X
Acute lymphoid leukemia	Male	LDI (I$ per capita)			X			X
Acute lymphoid leukemia	Male	Health System Access 2 (unitless)	X				X	
Acute lymphoid leukemia	Male	Log-transformed SEV scalar: Leukemia	X			X		
Acute lymphoid leukemia	Male	Smoking Prevalence	X				X	
Acute lymphoid leukemia	Male	Alcohol (liters per capita)		X			X	
Acute lymphoid leukemia	Female	Cumulative Cigarettes (10 Years)	X				X	
Acute lymphoid leukemia	Female	Socio-demographic Index			X			X
Acute lymphoid leukemia	Female	Cumulative Cigarettes (5 Years)	X				X	
Acute lymphoid leukemia	Female	Education (years per capita)			X			X

© 2018 Fitzmaurice C et al. JAMA Oncology.

Cause	Sex	Covariate	GBD 2015			GBD 2016		
			Level 1	Level 2	Level 3	Level 1	Level 2	Level 3
Acute lymphoid leukemia	Female	LDI (I$ per capita)			X			X
Acute lymphoid leukemia	Female	Health System Access 2 (unitless)	X				X	
Acute lymphoid leukemia	Female	Log-transformed SEV scalar: Leukemia	X			X		
Acute lymphoid leukemia	Female	Smoking Prevalence	X				X	
Acute lymphoid leukemia	Female	Alcohol (liters per capita)		X			X	
Chronic lymphoid leukemia	Female	Cumulative Cigarettes (10 Years)	X				X	
Chronic lymphoid leukemia	Female	Socio-demographic Index			X			X
Chronic lymphoid leukemia	Female	Cumulative Cigarettes (5 Years)	X				X	
Chronic lymphoid leukemia	Female	Education (years per capita)			X			X
Chronic lymphoid leukemia	Female	LDI (I$ per capita)			X			X
Chronic lymphoid leukemia	Female	Health System Access 2 (unitless)	X				X	
Chronic lymphoid leukemia	Female	Log-transformed SEV scalar: Leukemia	X			X		
Chronic lymphoid leukemia	Female	Smoking Prevalence	X				X	
Chronic lymphoid leukemia	Female	Alcohol (liters per capita)		X			X	
Chronic lymphoid leukemia	Male	Cumulative Cigarettes (10 Years)	X				X	
Chronic lymphoid leukemia	Male	Socio-demographic Index			X			X
Chronic lymphoid leukemia	Male	Cumulative Cigarettes (5 Years)	X				X	
Chronic lymphoid leukemia	Male	Education (years per capita)			X			X
Chronic lymphoid leukemia	Male	LDI (I$ per capita)			X			X
Chronic lymphoid leukemia	Male	Health System Access 2 (unitless)	X				X	
Chronic lymphoid leukemia	Male	Log-transformed SEV scalar: Leukemia	X			X		
Chronic lymphoid leukemia	Male	Smoking Prevalence	X				X	
Chronic lymphoid leukemia	Male	Alcohol (liters per capita)		X			X	
Acute myeloid leukemia	Male	Cumulative Cigarettes (10 Years)	X				X	
Acute myeloid leukemia	Male	Socio-demographic Index			X			X
Acute myeloid leukemia	Male	Cumulative Cigarettes (5 Years)	X				X	
Acute myeloid leukemia	Male	Tobacco (cigarettes per capita)	X				X	

© 2018 Fitzmaurice C et al. JAMA Oncology.

Cause	Sex	Covariate	GBD 2015			GBD 2016		
			Level 1	Level 2	Level 3	Level 1	Level 2	Level 3
Acute myeloid leukemia	Male	Education (years per capita)			X			X
Acute myeloid leukemia	Male	LDI (I$ per capita)			X			X
Acute myeloid leukemia	Male	Health System Access 2 (unitless)	X				X	
Acute myeloid leukemia	Male	Health System Access 2 (unitless)	X			X		
Acute myeloid leukemia	Male	Log-transformed SEV scalar: Leukemia	X			X		
Acute myeloid leukemia	Male	Smoking Prevalence	X				X	
Acute myeloid leukemia	Male	Alcohol (liters per capita)		X			X	
Acute myeloid leukemia	Female	Cumulative Cigarettes (10 Years)	X				X	
Acute myeloid leukemia	Female	Socio-demographic Index			X			X
Acute myeloid leukemia	Female	Cumulative Cigarettes (5 Years)	X				X	
Acute myeloid leukemia	Female	Tobacco (cigarettes per capita)	X				X	
Acute myeloid leukemia	Female	Education (years per capita)			X			X
Acute myeloid leukemia	Female	LDI (I$ per capita)			X			X
Acute myeloid leukemia	Female	Health System Access 2 (unitless)	X				X	
Acute myeloid leukemia	Female	Log-transformed SEV scalar: Leukemia	X			X		
Acute myeloid leukemia	Female	Smoking Prevalence	X				X	
Acute myeloid leukemia	Female	Alcohol (liters per capita)		X			X	
Chronic myeloid leukemia	Male	Cumulative Cigarettes (10 Years)	X				X	
Chronic myeloid leukemia	Male	Socio-demographic Index			X			X
Chronic myeloid leukemia	Male	Cumulative Cigarettes (5 Years)	X				X	
Chronic myeloid leukemia	Male	Education (years per capita)			X			X
Chronic myeloid leukemia	Male	LDI (I$ per capita)			X			X
Chronic myeloid leukemia	Male	Health System Access 2 (unitless)	X				X	
Chronic myeloid leukemia	Male	Smoking Prevalence	X				X	
Chronic myeloid leukemia	Male	Alcohol (liters per capita)		X			X	
Chronic myeloid leukemia	Female	Cumulative Cigarettes (10 Years)	X				X	
Chronic myeloid leukemia	Female	Socio-demographic Index			X			X

© 2018 Fitzmaurice C et al. JAMA Oncology.

Cause	Sex	Covariate	GBD 2015			GBD 2016		
			Level 1	Level 2	Level 3	Level 1	Level 2	Level 3
Chronic myeloid leukemia	Female	Cumulative Cigarettes (5 Years)	X				X	
Chronic myeloid leukemia	Female	Education (years per capita)			X			X
Chronic myeloid leukemia	Female	LDI (I$ per capita)			X			X
Chronic myeloid leukemia	Female	Health System Access 2 (unitless)	X				X	
Chronic myeloid leukemia	Female	Smoking Prevalence	X				X	
Chronic myeloid leukemia	Female	Alcohol (liters per capita)		X			X	
Other neoplasms	Male	Tobacco (cigarettes per capita)	X			X		
Other neoplasms	Male	Education (years per capita)			X			X
Other neoplasms	Male	Health System Access 2 (unitless)		X			X	
Other neoplasms	Male	LDI (I$ per capita)			X			X
Other neoplasms	Male	Nuts & Seeds (kcal per capita)		X			X	
Other neoplasms	Male	Smoking Prevalence	X			X		
Other neoplasms	Male	Socio-demographic Index			X			X
Other neoplasms	Female	Tobacco (cigarettes per capita)	X			X		
Other neoplasms	Female	Education (years per capita)			X			X
Other neoplasms	Female	Health System Access 2 (unitless)		X			X	
Other neoplasms	Female	LDI (I$ per capita)			X			X
Other neoplasms	Female	Smoking Prevalence	X			X		
Other neoplasms	Female	Socio-demographic Index			X			X

eTable 10: Results for CODEm model testing

Cause	Sex	Age start	Age end	Predictive validity					
				RMSE in	RMSE out	Trend in	Trend out	Coverage in	Coverage out
Esophageal cancer [Data Rich]	Male	15-19 years	95+ years	0.198433	0.245255	0.161804	0.185832	0.998919	0.997968
Esophageal cancer [Data	Female	15-19	95+	0.215283	0.268253	0.179366	0.206173	0.998593	0.997497

© 2018 Fitzmaurice C et al. JAMA Oncology.

Cause	Sex	Age start	Age end	Predictive validity					
				RMSE in	RMSE out	Trend in	Trend out	Coverage in	Coverage out
Rich]		years	years						
Esophageal cancer [Global]	Female	15-19 years	95+ years	0.272331	0.449786	0.211737	0.217784	0.997501	0.980359
Esophageal cancer [Global]	Male	15-19 years	95+ years	0.238675	0.413533	0.185874	0.189452	0.997966	0.980274
Stomach cancer [Data Rich]	Male	15-19 years	95+ years	0.168624	0.204856	0.135969	0.153663	0.998632	0.997638
Stomach cancer [Data Rich]	Female	15-19 years	95+ years	0.174226	0.211966	0.142399	0.164469	0.998495	0.99713
Stomach cancer [Global]	Female	15-19 years	95+ years	0.204713	0.321435	0.162978	0.160719	0.99867	0.984612
Stomach cancer [Global]	Male	15-19 years	95+ years	0.196924	0.307854	0.153815	0.152673	0.998643	0.980242
Liver cancer [Global]	Male	5-9 years	95+ years	0.269051	0.454562	0.209164	0.210674	0.998031	0.986505
Liver cancer [Data Rich]	Male	5-9 years	95+ years	0.2413	0.325407	0.191496	0.224282	0.998065	0.996981
Liver cancer [Global]	Female	5-9 years	95+ years	0.277507	0.413467	0.216708	0.218733	0.998803	0.991523
Liver cancer [Data Rich]	Female	5-9 years	95+ years	0.232587	0.30673	0.193079	0.226226	0.998381	0.997116
Larynx cancer [Data Rich]	Male	15-19 years	95+ years	0.200641	0.252556	0.161284	0.192664	0.997858	0.997282
Larynx cancer [Data Rich]	Female	15-19 years	95+ years	0.30673	0.388496	0.253812	0.302646	0.975795	0.977395
Larynx cancer [Global]	Male	15-19 years	95+ years	0.234619	0.374157	0.181438	0.191236	0.998393	0.986868
Larynx cancer [Global]	Female	15-19 years	95+ years	0.356904	0.5464	0.283167	0.287904	0.98139	0.973441
Tracheal, bronchus, and lung cancer [Data Rich]	Female	15-19 years	95+ years	0.188871	0.231498	0.154034	0.180269	0.998449	0.997224

© 2018 Fitzmaurice C et al. JAMA Oncology.

Cause	Sex	Age start	Age end	Predictive validity					
				RMSE in	RMSE out	Trend in	Trend out	Coverage in	Coverage out
Tracheal, bronchus, and lung cancer [Data Rich]	Male	15-19 years	95+ years	0.181641	0.221799	0.146639	0.164353	0.99861	0.997327
Tracheal, bronchus, and lung cancer [Global]	Female	15-19 years	95+ years	0.225478	0.304297	0.174547	0.175022	0.998643	0.995915
Tracheal, bronchus, and lung cancer [Global]	Male	15-19 years	95+ years	0.212545	0.307851	0.166159	0.169413	0.998557	0.990068
Breast cancer [Data Rich]	Male	15-19 years	95+ years	0.339918	0.42271	0.265862	0.31327	0.97947	0.976519
Breast cancer [Data Rich]	Female	15-19 years	95+ years	0.192908	0.233432	0.155652	0.177959	0.996331	0.993547
Breast cancer [Global]	Female	15-19 years	95+ years	0.211224	0.287481	0.162086	0.167525	0.997149	0.990481
Breast cancer [Global]	Male	15-19 years	95+ years	0.423524	0.594604	0.313547	0.315621	0.979072	0.968179
Cervical cancer [Data Rich]	Female	15-19 years	95+ years	0.193564	0.239786	0.156019	0.182773	0.998702	0.997568
Cervical cancer [Global]	Female	15-19 years	95+ years	0.244551	0.350403	0.188472	0.186523	0.999189	0.990516
Uterine cancer [Global]	Female	15-19 years	95+ years	0.26113	0.405926	0.203732	0.203708	0.999474	0.993606
Uterine cancer [Data Rich]	Female	15-19 years	95+ years	0.215364	0.272806	0.172502	0.20594	0.999534	0.998942
Prostate cancer [Data Rich]	Male	15-19 years	95+ years	0.25085	0.303217	0.204423	0.237726	0.996921	0.994794
Prostate cancer [Global]	Male	15-19 years	95+ years	0.29197	0.370344	0.218901	0.21368	0.996743	0.986557
Colon and rectum cancer [Data Rich]	Male	15-19 years	95+ years	0.185598	0.224739	0.152051	0.172096	0.998745	0.997677
Colon and rectum cancer [Data Rich]	Female	15-19 years	95+ years	0.195509	0.240842	0.161929	0.192825	0.998011	0.996491
Colon and rectum cancer	Male	15-19	95+	0.208621	0.291093	0.166006	0.167229	0.999046	0.991509

© 2018 Fitzmaurice C et al. JAMA Oncology.

Cause	Sex	Age start	Age end	Predictive validity					
				RMSE in	RMSE out	Trend in	Trend out	Coverage in	Coverage out
[Global]		years	years						
Colon and rectum cancer [Global]	Female	15-19 years	95+ years	0.215863	0.294113	0.171992	0.168904	0.998465	0.990486
Lip and oral cavity cancer [Data Rich]	Male	15-19 years	95+ years	0.207908	0.252841	0.168893	0.183409	0.999097	0.998219
Lip and oral cavity cancer [Data Rich]	Female	15-19 years	95+ years	0.217054	0.273426	0.177172	0.204022	0.999549	0.999077
Lip and oral cavity cancer [Global]	Male	15-19 years	95+ years	0.252937	0.355882	0.20016	0.196201	0.998305	0.991385
Lip and oral cavity cancer [Global]	Female	15-19 years	95+ years	0.252204	0.349135	0.192715	0.197068	0.999507	0.995154
Nasopharynx cancer [Data Rich]	Female	5-9 years	95+ years	0.305983	0.418084	0.256857	0.342077	0.991357	0.991272
Nasopharynx cancer [Data Rich]	Male	5-9 years	95+ years	0.256041	0.343061	0.207344	0.249863	0.998424	0.997617
Nasopharynx cancer [Global]	Female	5-9 years	95+ years	0.361818	0.574134	0.287872	0.294643	0.990987	0.982361
Nasopharynx cancer [Global]	Male	5-9 years	95+ years	0.303468	0.507949	0.238899	0.243384	0.998489	0.987144
Other pharynx cancer [Data Rich]	Male	15-19 years	95+ years	0.243933	0.316171	0.196356	0.221139	0.99611	0.995076
Other pharynx cancer [Data Rich]	Female	15-19 years	95+ years	0.257668	0.327035	0.210857	0.248682	0.994188	0.993541
Other pharynx cancer [Global]	Male	15-19 years	95+ years	0.287334	0.46305	0.218922	0.209273	0.996444	0.987678
Other pharynx cancer [Global]	Female	15-19 years	95+ years	0.319571	0.475567	0.248676	0.243749	0.994634	0.987119
Gallbladder and biliary tract cancer [Data Rich]	Female	15-19 years	95+ years	0.203972	0.267017	0.165798	0.189961	0.995926	0.994112
Gallbladder and biliary tract cancer [Data Rich]	Male	15-19 years	95+ years	0.200502	0.247264	0.162741	0.182337	0.997554	0.996224

© 2018 Fitzmaurice C et al. JAMA Oncology.

Cause	Sex	Age start	Age end	Predictive validity					
				RMSE in	RMSE out	Trend in	Trend out	Coverage in	Coverage out
Gallbladder and biliary tract cancer [Global]	Female	15-19 years	95+ years	0.24119	0.382075	0.18688	0.183792	0.99678	0.982529
Gallbladder and biliary tract cancer [Global]	Male	15-19 years	95+ years	0.261043	0.375592	0.187773	0.184805	0.998613	0.989089
Pancreatic cancer [Data Rich]	Male	15-19 years	95+ years	0.173161	0.212802	0.140106	0.151654	0.998714	0.997757
Pancreatic cancer [Data Rich]	Female	15-19 years	95+ years	0.19234	0.240872	0.158471	0.180492	0.997995	0.996599
Pancreatic cancer [Global]	Male	15-19 years	95+ years	0.198254	0.284044	0.152835	0.151022	0.998854	0.995525
Pancreatic cancer [Global]	Female	15-19 years	95+ years	0.209814	0.286474	0.168889	0.166741	0.998889	0.996591
Malignant skin melanoma [Data Rich]	Male	15-19 years	95+ years	0.258707	0.324742	0.207811	0.237785	0.999191	0.998467
Malignant skin melanoma [Data Rich]	Female	15-19 years	95+ years	0.256129	0.301867	0.200431	0.216407	0.998728	0.997828
Malignant skin melanoma [Global]	Male	15-19 years	95+ years	0.296332	0.434735	0.225174	0.226047	0.998842	0.994037
Malignant skin melanoma [Global]	Female	15-19 years	95+ years	0.292432	0.42973	0.222704	0.226799	0.998281	0.991761
Non-melanoma skin cancer [Data Rich]	Male	15-19 years	95+ years	0.155711	0.239935	0.112916	0.133783	0.99981	0.999603
Non-melanoma skin cancer [Data Rich]	Female	15-19 years	95+ years	0.214155	0.309436	0.159857	0.183713	0.997395	0.997199
Non-melanoma skin cancer [Global]	Female	15-19 years	95+ years	0.291672	0.489846	0.209023	0.217962	0.994887	0.985998
Non-melanoma skin cancer [Global]	Male	15-19 years	95+ years	0.192737	0.330459	0.136252	0.14092	0.999232	0.995604
Ovarian cancer [Data Rich]	Female	15-19 years	95+ years	0.195794	0.246041	0.157071	0.173662	0.998842	0.997833
Ovarian cancer [Global]	Female	15-19	95+	0.218013	0.313157	0.166727	0.167835	0.999174	0.994091

© 2018 Fitzmaurice C et al. JAMA Oncology.

Cause	Sex	Age start	Age end	Predictive validity					
				RMSE in	RMSE out	Trend in	Trend out	Coverage in	Coverage out
		years	years						
Testicular cancer [Global]	Male	15-19 years	95+ years	0.328371	0.529164	0.255569	0.25659	0.999375	0.995125
Testicular cancer [Data Rich]	Male	15-19 years	95+ years	0.283022	0.371326	0.232189	0.243099	0.999645	0.999282
Kidney cancer [Data Rich]	Male	0-6 days	95+ years	0.241409	0.355526	0.19905	0.233492	0.999066	0.998706
Kidney cancer [Data Rich]	Female	0-6 days	95+ years	0.269859	0.399459	0.223989	0.265876	0.998886	0.997983
Kidney cancer [Global]	Male	0-6 days	95+ years	0.280428	0.40437	0.224896	0.23264	0.999225	0.994304
Kidney cancer [Global]	Female	0-6 days	95+ years	0.309672	0.434952	0.251863	0.260629	0.999065	0.992195
Bladder cancer [Data Rich]	Male	15-19 years	95+ years	0.238162	0.289199	0.193221	0.21405	0.998893	0.9979
Bladder cancer [Global]	Male	15-19 years	95+ years	0.259512	0.358356	0.20438	0.211875	0.998849	0.993972
Bladder cancer [Global]	Female	15-19 years	95+ years	0.279362	0.406151	0.217047	0.218843	0.995797	0.992228
Bladder cancer [Data Rich]	Female	15-19 years	95+ years	0.241778	0.308128	0.197425	0.236098	0.995139	0.99458
Brain and nervous system cancer [Data Rich]	Female	0-6 days	95+ years	0.233403	0.3046	0.177532	0.189069	0.998845	0.997948
Brain and nervous system cancer [Global]	Female	0-6 days	95+ years	0.302238	0.394989	0.217704	0.215738	0.998871	0.995489
Brain and nervous system cancer [Data Rich]	Male	0-6 days	95+ years	0.216468	0.277928	0.168084	0.183155	0.998868	0.997924
Brain and nervous system cancer [Global]	Male	0-6 days	95+ years	0.27435	0.374644	0.204471	0.20185	0.998951	0.996187
Thyroid cancer [Data Rich]	Female	10-14 years	95+ years	0.428862	0.514646	0.348096	0.339467	0.988448	0.986206

© 2018 Fitzmaurice C et al. JAMA Oncology.

Cause	Sex	Age start	Age end	Predictive validity					
				RMSE in	RMSE out	Trend in	Trend out	Coverage in	Coverage out
Thyroid cancer [Data Rich]	Male	10-14 years	95+ years	0.325439	0.400471	0.258836	0.251195	0.990913	0.991523
Thyroid cancer [Global]	Female	10-14 years	95+ years	0.452233	0.57687	0.360692	0.356627	0.990555	0.981127
Thyroid cancer [Global]	Male	10-14 years	95+ years	0.365192	0.48212	0.280751	0.28231	0.992984	0.989187
Mesothelioma [Data Rich]	Female	15-19 years	95+ years	0.196941	0.304164	0.152358	0.219033	0.998746	0.998454
Mesothelioma [Data Rich]	Male	15-19 years	95+ years	0.172967	0.285052	0.128708	0.171164	0.999971	0.999797
Mesothelioma [Global]	Male	15-19 years	95+ years	0.241276	0.464505	0.185143	0.181593	0.999543	0.989174
Mesothelioma [Global]	Female	15-19 years	95+ years	0.260519	0.427073	0.202264	0.205672	0.998568	0.995729
Hodgkin lymphoma [Data Rich]	Male	0-6 days	95+ years	0.350903	0.46589	0.27357	0.318263	0.99713	0.947085
Hodgkin lymphoma [Data Rich]	Female	0-6 days	95+ years	0.446449	0.618172	0.339263	0.421206	0.960309	0.961281
Hodgkin lymphoma [Global]	Male	0-6 days	95+ years	0.418144	0.544918	0.308703	0.308167	0.998369	0.99283
Hodgkin lymphoma [Global]	Female	0-6 days	95+ years	0.590103	0.786115	0.402028	0.406353	0.972256	0.963972
Non-Hodgkin lymphoma [Data Rich]	Male	0-6 days	95+ years	0.257262	0.319446	0.198048	0.224728	0.998985	0.998708
Non-Hodgkin lymphoma [Data Rich]	Female	0-6 days	95+ years	0.267228	0.346181	0.210521	0.242819	0.998808	0.998423
Non-Hodgkin lymphoma [Global]	Male	0-6 days	95+ years	0.294371	0.40601	0.217911	0.227594	0.998919	0.992808
Non-Hodgkin lymphoma [Global]	Female	0-6 days	95+ years	0.300954	0.416656	0.228722	0.233181	0.998752	0.993317
Multiple myeloma [Data	Male	15-19	95+	0.229224	0.274483	0.181391	0.198275	0.999263	0.998586

© 2018 Fitzmaurice C et al. JAMA Oncology.

Cause	Sex	Age start	Age end	Predictive validity					
				RMSE in	RMSE out	Trend in	Trend out	Coverage in	Coverage out
Rich]		years	years						
Multiple myeloma [Data Rich]	Female	15-19 years	95+ years	0.239102	0.290048	0.187379	0.216489	0.999277	0.998797
Multiple myeloma [Global]	Male	15-19 years	95+ years	0.273879	0.3742	0.206672	0.211225	0.999194	0.994302
Multiple myeloma [Global]	Female	15-19 years	95+ years	0.277053	0.38199	0.206454	0.209223	0.999174	0.996651
Leukemia [Data Rich]	Female	0-6 days	95+ years	0.232168	0.285674	0.190505	0.222964	0.997925	0.996086
Leukemia [Data Rich]	Male	0-6 days	95+ years	0.218797	0.271475	0.178922	0.207808	0.998391	0.99703
Leukemia [Global]	Female	0-6 days	95+ years	0.283536	0.361995	0.242014	0.242573	0.99865	0.993203
Leukemia [Global]	Male	0-6 days	95+ years	0.294539	0.379	0.275613	0.270439	0.997571	0.992988
Other leukemia [Data Rich]	Female	0-6 days	95+ years	0.3508	0.490285	0.278612	0.300191	0.996341	0.993081
Other leukemia [Data Rich]	Male	0-6 days	95+ years	0.282754	0.349516	0.239149	0.221327	0.998039	0.995322
Other leukemia [Global]	Female	0-6 days	95+ years	0.413955	0.570583	0.311448	0.31415	0.997038	0.983931
Other leukemia [Global]	Male	0-6 days	95+ years	0.34794	0.480636	0.275768	0.263252	0.998417	0.987829
Acute lymphoid leukemia [Data Rich]	Female	0-6 days	95+ years	0.245042	0.343861	0.199697	0.196121	0.999735	0.998677
Acute lymphoid leukemia [Data Rich]	Male	0-6 days	95+ years	0.232196	0.322463	0.192678	0.180934	0.999027	0.997797
Acute lymphoid leukemia [Global]	Female	0-6 days	95+ years	0.292299	0.430489	0.219397	0.219718	0.999584	0.995845
Acute lymphoid leukemia [Global]	Male	0-6 days	95+ years	0.258168	0.380972	0.20477	0.202271	0.999191	0.996156

© 2018 Fitzmaurice C et al. JAMA Oncology.

Cause	Sex	Age start	Age end	Predictive validity					
				RMSE in	RMSE out	Trend in	Trend out	Coverage in	Coverage out
Chronic lymphoid leukemia [Data Rich]	Male	15-19 years	95+ years	0.264066	0.422453	0.205004	0.200284	0.998068	0.99624
Chronic lymphoid leukemia [Data Rich]	Female	15-19 years	95+ years	0.229014	0.365306	0.185879	0.188733	0.997496	0.995669
Chronic lymphoid leukemia [Global]	Male	15-19 years	95+ years	0.281442	0.49744	0.21614	0.218752	0.997518	0.992235
Chronic lymphoid leukemia [Global]	Female	15-19 years	95+ years	0.268922	0.428058	0.20953	0.209509	0.997841	0.991753
Acute myeloid leukemia [Data Rich]	Female	0-6 days	95+ years	0.243902	0.359063	0.198629	0.202072	0.999145	0.99819
Acute myeloid leukemia [Data Rich]	Male	0-6 days	95+ years	0.232648	0.331949	0.175921	0.178595	0.999717	0.999322
Acute myeloid leukemia [Global]	Male	0-6 days	95+ years	0.265031	0.360267	0.184561	0.180444	0.99956	0.995375
Acute myeloid leukemia [Global]	Female	0-6 days	95+ years	0.281417	0.410413	0.213481	0.218102	0.999242	0.994128
Chronic myeloid leukemia [Data Rich]	Female	28-364 days	95+ years	0.257258	0.362538	0.219213	0.209664	0.999697	0.998442
Chronic myeloid leukemia [Global]	Female	28-364 days	95+ years	0.295483	0.439364	0.230637	0.214173	0.99951	0.996227
Chronic myeloid leukemia [Global]	Male	28-364 days	95+ years	0.27712	0.436653	0.219288	0.215423	0.999912	0.997674
Chronic myeloid leukemia [Data Rich]	Male	28-364 days	95+ years	0.247795	0.36944	0.21216	0.201106	0.999952	0.999142
Non-melanoma skin cancer (squamous-cell carcinoma) [Data Rich]	Male	28-364 days	95+ years	0.154952	0.235392	0.111747	0.134674	0.999782	0.999627
Non-melanoma skin cancer (squamous-cell carcinoma) [Data Rich]	Female	28-364 days	95+ years	0.21458	0.296797	0.160927	0.184583	0.997336	0.997268
Non-melanoma skin cancer	Male	28-364	95+	0.186211	0.307069	0.133081	0.13346	0.999225	0.993893

© 2018 Fitzmaurice C et al. JAMA Oncology.

Cause	Sex	Age start	Age end	Predictive validity					
				RMSE in	RMSE out	Trend in	Trend out	Coverage in	Coverage out
(squamous-cell carcinoma) [Global]		days	years						
Non-melanoma skin cancer (squamous-cell carcinoma) [Global]	Female	28-364 days	95+ years	0.27535	0.449328	0.203049	0.202074	0.996456	0.987022
Other neoplasms [Data Rich]	Male	0-6 days	95+ years	0.256396	0.315719	0.217052	0.239815	0.993394	0.988905
Other neoplasms [Data Rich]	Female	0-6 days	95+ years	0.266244	0.330966	0.224429	0.255028	0.993927	0.989754
Other neoplasms [Global]	Female	0-6 days	95+ years	0.287964	0.370172	0.237436	0.240632	0.995797	0.992799
Other neoplasms [Global]	Male	0-6 days	95+ years	0.277797	0.363343	0.227826	0.234498	0.995245	0.992186

© 2018 Fitzmaurice C et al. JAMA Oncology.

eTable 11: Percent change before and after CodCorrect by cancer for all ages, both sexes combined, 2016

**Cause**	**CodCorrect level**	**Percent change (%)**
Neoplasms	2	-1.19 (-2.61 to 0.59)
Esophageal cancer	3	-1.39 (-2.6 to 0.19)
Stomach cancer	3	-1.88 (-3.12 to -0.25)
Liver cancer	3	-1.2 (-2.69 to 1.12)
Liver cancer due to hepatitis B	4	-0.59 (-2.21 to 2.08)
Liver cancer due to hepatitis C	4	-2.59 (-3.98 to -0.86)
Liver cancer due to alcohol use	4	-1.87 (-3.7 to 0.33)
Liver cancer due to other causes	4	-0.65 (-2.07 to 1.69)
Larynx cancer	3	-0.77 (-2.33 to 1.03)
Tracheal, bronchus, and lung cancer	3	-2.23 (-3.42 to -0.62)
Breast cancer	3	-0.37 (-2.34 to 1.76)
Cervical cancer	3	3.5 (0.72 to 6.15)
Uterine cancer	3	0.05 (-2.11 to 1.94)
Prostate cancer	3	-0.98 (-2.71 to 0.95)
Colon and rectum cancer	3	-1.95 (-3.4 to -0.16)
Lip and oral cavity cancer	3	-0.39 (-2.3 to 1.54)
Nasopharynx cancer	3	0.26 (-1.36 to 3.21)
Other pharynx cancer	3	0.94 (-1.74 to 2.85)
Gallbladder and biliary tract cancer	3	-2.65 (-4.45 to -0.79)
Pancreatic cancer	3	-2.11 (-3.57 to -0.34)
Malignant skin melanoma	3	-0.7 (-2.07 to 0.99)
Non-melanoma skin cancer	3	-1.85 (-3.14 to -0.14)
Ovarian cancer	3	-0.37 (-2.32 to 1.5)
Testicular cancer	3	4.01 (1.79 to 7.54)
Kidney cancer	3	-1.83 (-3.18 to -0.23)
Bladder cancer	3	-2.19 (-3.56 to -0.52)
Brain and nervous system cancer	3	-0.24 (-1.96 to 2.3)
Thyroid cancer	3	0.67 (-1.23 to 2.44)
Mesothelioma	3	-1.14 (-2.49 to 0.61)
Hodgkin lymphoma	3	2.55 (0.15 to 5.06)
Non-Hodgkin lymphoma	3	-0.01 (-1.7 to 1.88)
Multiple myeloma	3	-1.25 (-2.68 to 0.36)
Leukemia	3	-0.42 (-2.46 to 2.27)
Other leukemia	4	2.9 (-3.46 to 13.65)
Acute lymphoid leukemia	4	1.81 (-5.35 to 11.97)
Chronic lymphoid leukemia	4	0.02 (-5.76 to 4.86)
Acute myeloid leukemia	4	1.35 (-3.24 to 7.78)

© 2018 Fitzmaurice C et al. JAMA Oncology.

Cause	CodCorrect level	Percent change (%)
Chronic myeloid leukemia	4	6.01 (-0.31 to 12.87)
Non-melanoma skin cancer (squamous-cell carcinoma)	4	-14.59 (-17.41 to -12.46)
Other neoplasms	3	0.34 (-1.28 to 2.57)

© 2018 Fitzmaurice C et al. JAMA Oncology.

eTable 12: Duration of four prevalence phases by cancer

	Diagnosis/ Treatment (months)	Remission	Disseminated/metastatic (months)	Note	Terminal (months)
Esophageal cancer	5[30]	Calculated based on remainder of time after attributing other sequelae.	4.6[31]	SEER Summary Stage 1997 (Distant site/node involved) 1995-2000	1 months
Stomach cancer	5.2[30]		3.88[31]	SEER Summary Stage 1997 (Distant site/node involved) 1995-2000	
Liver cancer	4		2.51[31]	SEER Summary Stage 1997 (Distant site/node involved) 1995-2000	
Larynx cancer	5.3[30]		8.84[31]	SEER Stage IVc	
Lung cancer	3.3[32]		4.51[31]	SEER Summary Stage 1997 (Distant site/node involved) 1995-2000	
Breast cancer	3[32]		17.7[31]	SEER Summary Stage 1997 (Distant site/node involved) 1995-2000	
Cervical cancer	4.8[30]		9.21[31]	SEER Summary Stage 1997 (Distant site/node involved) 1995-2000	
Uterine cancer	4.6[30]		11.6[31]	SEER Summary Stage 1997 (Distant site/node involved) 1995-2000	
Prostate cancer	4[32]		30.35[31]	SEER Summary Stage 1997 (Distant site/node involved) 1995-2000	
Colorectal cancer	4[32]		9.69[31]	SEER Summary Stage 1997 (Distant site/node involved) 1995-2000	
Oral cancer	5.3[30]		9.33[31]	SEER Stage IVc	
Nasopharyngeal cancer	5.3[30]		13.19[31]	SEER Stage IVc	
Cancer of other part of pharynx	5.3[30]		7.91[31]	SEER Stage IVc	
Gallbladder cancer	4		3.47[31]	SEER Summary Stage 1997 (Distant site/node involved) 1995-2000	
Pancreas cancer	4.1[30]		2.54[31]	SEER Summary Stage 1997 (Distant site/node involved) 1995-2000	

© 2018 Fitzmaurice C et al. JAMA Oncology.

	Diagnosis/ Treatment (months)	Remission	Disseminated/metastatic (months)	Note	Terminal (months)
Melanoma	2.9[33]		7.18[31]	SEER Summary Stage 1997 (Distant site/node involved) 1995-2000	
NMSC (squamous cell carcinoma)	2.9[33]		17[34]		
Ovarian cancer	3.2[32]		25.6[31]	SEER Summary Stage 1997 (Distant site/node involved) 1995-2000	
Testicular cancer	3.7[30]		19.47[31]	SEER Stage III	
Kidney cancer	5.3[30]		5.38[31]	SEER Summary Stage 1997 (Distant site/node involved) 1995-2000	
Bladder cancer	5.1[30]		5.8[31]	SEER Summary Stage 1997 (Distant site/node involved) 1995-2000	
Brain cancer	5		6.93[31]	SEER Median age standardized survival all patients, all years	
Thyroid cancer	3		19.39[31]	SEER Stage IVc	
Mesothelioma	4		7.75[31]	SEER Summary Stage 1997 (Distant site/node involved) 1995-2000	
Hodgkin lymphoma	3.7[32]		26[35]		
Non Hodgkin lymphoma	3.7[32]		7.7[35]		
Multiple myeloma	7[30]		36.82[31]	SEER Median age standardized survival all patients, all years	
Leukemia[30]	5		43.67[31]	SEER Median age standardized survival all patients, all years	
ALL	12		7.02[31]	SEER Median age standardized survival all patients, all years	
AML	6		4.6[31]	SEER Median age standardized survival all patients, all years	
CLL	6		48[36]		
CML	6		4.6[31]	SEER Median age standardized survival for AML (patients with CML die in blast	

© 2018 Fitzmaurice C et al. JAMA Oncology.

	Diagnosis/ Treatment (months)	Remission	Disseminated/metastatic (months)	Note	Terminal (months)
				crisis, which is treated like AML) all patients, all years	
Other leukemia	6		48[36]		
Other	4.4 (mean of other cancer durations)		15.81[31]	SEER Median age standardized survival all patients, all years	

© 2018 Fitzmaurice C et al. JAMA Oncology.

eTable 13: Disability weights

Health state	Lay description	Estimate	Uncertainty interval	
Cancer, diagnosis and primary therapy	Has pain, nausea, fatigue, weight loss and high anxiety.	0.288	0.193	0.399
Cancer, controlled phase	Has a chronic disease that requires medication every day and causes some worry but minimal interference with daily activities.	0.049	0.031	0.072
Cancer, metastatic	Has severe pain, extreme fatigue, weight loss and high anxiety.	0.451	0.307	0.600
Terminal phase, with medication	Has lost a lot of weight and regularly uses strong medication to avoid constant pain. The person has no appetite, feels nauseous, and needs to spend most of the day in bed.	0.540	0.377	0.687
Mastectomy	Had one of the breasts removed and sometimes has pain or swelling in the arms.	0.036	0.020	0.057
Stoma	Has a pouch attached to an opening in the belly to collect and empty stools.	0.095	0.063	0.131
Laryngectomy	Has difficulty speaking, and others find it difficult to understand.	0.051	0.032	0.078
Urinary incontinence	Cannot control urinating.	0.139	0.094	0.198
Impotence	Has difficulty in obtaining or maintaining an erection.	0.017	0.009	0.030

© 2018 Fitzmaurice C et al. JAMA Oncology.

eTable 14: Decomposition of trends in incidence globally, and by SDI quintile, both sexes, 2006 to 2016

Location		Incidence cases, No.		Expected incidence cases, 2016, No.		Change in incidence cases, 1990 to 2016, %			Overall change, %
		2006	2016	Given population growth alone	Given population growth and aging	Due to population growth	Due to change in age structure	Due to change in incidence rate	
Global	Neoplasms	13414991 (13035144 to 13851075)	17227632 (16712769 to 17802548)	15082343	17367274	12.4	17	-1	28.4
High SDI	Neoplasms	5844162 (5583309 to 6160012)	7031745 (6704656 to 7398378)	6156321	7080101	5.3	15.8	-0.8	20.3
High-middle SDI	Neoplasms	2550090 (2507380 to 2604682)	3349475 (3258731 to 3460355)	2833196	3248231	11.1	16.3	4	31.3
Middle SDI	Neoplasms	3335555 (3252321 to 3409296)	4598970 (4478183 to 4725368)	3580523	4413440	7.3	25	5.6	37.9
Low-middle SDI	Neoplasms	1346658 (1304402 to 1385677)	1784107 (1744975 to 1823028)	1570501	1776791	16.6	15.3	0.5	32.5
Low SDI	Neoplasms	335133 (302282 to 354854)	442185 (395504 to 463281)	443392	453927	32.3	3.1	-3.5	31.9
Global	Lip and oral cavity cancer	287037 (283253 to 291135)	381974 (370715 to 392125)	322712	369080	12.4	16.2	4.5	33.1
High SDI	Lip and oral cavity cancer	71667 (70595 to 72790)	85014 (81846 to 88201)	75494	85567	5.3	14.1	-0.8	18.6

© 2018 Fitzmaurice C et al. JAMA Oncology.

Location		Incidence cases, No.		Expected incidence cases, 2016, No.		Change in incidence cases, 1990 to 2016, %			Overall change, %
		2006	2016	Given population growth alone	Given population growth and aging	Due to population growth	Due to change in age structure	Due to change in incidence rate	
High-middle SDI	Lip and oral cavity cancer	40566 (39454 to 41788)	49913 (47666 to 52321)	45069	52170	11.1	17.5	-5.6	23
Middle SDI	Lip and oral cavity cancer	67999 (66996 to 69014)	98889 (94382 to 101678)	72993	90221	7.3	25.3	12.7	45.4
Low-middle SDI	Lip and oral cavity cancer	100832 (98310 to 103446)	140674 (133355 to 146157)	117592	133925	16.6	16.2	6.7	39.5
Low SDI	Lip and oral cavity cancer	7624 (7309 to 8080)	10550 (10205 to 10931)	10086	10368	32.3	3.7	2.4	38.4
Global	Nasopharynx cancer	88213 (85305 to 90956)	95608 (90817 to 101115)	99177	107805	12.4	9.8	-13.8	8.4
High SDI	Nasopharynx cancer	10108 (9808 to 10434)	10103 (9621 to 10742)	10648	11427	5.3	7.7	-13.1	0
High-middle SDI	Nasopharynx cancer	23257 (21902 to 24667)	24346 (21962 to 26853)	25838	28216	11.1	10.2	-16.6	4.7
Middle SDI	Nasopharynx cancer	37082 (35912 to 38292)	39310 (36836 to 41715)	39805	45313	7.3	14.9	-16.2	6
Low-middle SDI	Nasopharynx cancer	14512 (13320 to 15513)	17671 (16747 to 18542)	16924	18750	16.6	12.6	-7.4	21.8

© 2018 Fitzmaurice C et al. JAMA Oncology.

Location		Incidence cases, No.		Expected incidence cases, 2016, No.		Change in incidence cases, 1990 to 2016, %			Overall change, %
		2006	2016	Given population growth alone	Given population growth and aging	Due to population growth	Due to change in age structure	Due to change in incidence rate	
Low SDI	Nasopharynx cancer	2970 (2534 to 3470)	3811 (3373 to 4408)	3928	4012	32.3	2.8	-6.8	28.3
Global	Other pharynx cancer	124249 (121736 to 126963)	169552 (159409 to 175514)	139692	160851	12.4	17	7	36.5
High SDI	Other pharynx cancer	35013 (34405 to 35596)	44631 (42482 to 45851)	36883	41224	5.3	12.4	9.7	27.5
High-middle SDI	Other pharynx cancer	17545 (16978 to 18134)	23861 (22788 to 24968)	19492	22871	11.1	19.3	5.6	36
Middle SDI	Other pharynx cancer	21785 (21224 to 22321)	32723 (29186 to 34705)	23385	29246	7.3	26.9	16	50.2
Low-middle SDI	Other pharynx cancer	48308 (46042 to 50836)	66970 (60455 to 71169)	56338	64686	16.6	17.3	4.7	38.6
Low SDI	Other pharynx cancer	2748 (2532 to 3045)	3516 (3297 to 3855)	3635	3721	32.3	3.1	-7.5	27.9
Global	Esophageal cancer	413137 (407522 to 418815)	442599 (433254 to 456192)	464485	540391	12.4	18.4	-23.7	7.1
High SDI	Esophageal cancer	72614 (71745 to 73451)	86401 (81501 to 90840)	76492	88543	5.3	16.6	-3	19

© 2018 Fitzmaurice C et al. JAMA Oncology.

Location		Incidence cases, No.		Expected incidence cases, 2016, No.		Change in incidence cases, 1990 to 2016, %			Overall change, %
		2006	2016	Given population growth alone	Given population growth and aging	Due to population growth	Due to change in age structure	Due to change in incidence rate	
High-middle SDI	Esophageal cancer	75851 (73953 to 77630)	83187 (80195 to 87238)	84271	98643	11.1	18.9	-20.4	9.7
Middle SDI	Esophageal cancer	202143 (198105 to 206114)	197973 (192251 to 203503)	216988	277435	7.3	29.9	-39.3	-2.1
Low-middle SDI	Esophageal cancer	46543 (45586 to 47579)	54670 (53263 to 56187)	54279	62523	16.6	17.7	-16.9	17.5
Low SDI	Esophageal cancer	12164 (11791 to 12531)	16670 (16047 to 17426)	16093	16515	32.3	3.5	1.3	37
Global	Stomach cancer	1002458 (991851 to 1013372)	1156619 (1134360 to 1179828)	1127053	1307918	12.4	18	-15.1	15.4
High SDI	Stomach cancer	254241 (251114 to 257474)	292834 (286623 to 299363)	267820	314185	5.3	18.2	-8.4	15.2
High-middle SDI	Stomach cancer	218180 (213700 to 222909)	243582 (233757 to 253492)	242402	279723	11.1	17.1	-16.6	11.6
Middle SDI	Stomach cancer	383988 (377257 to 390982)	454079 (438498 to 472218)	412188	521010	7.3	28.3	-17.4	18.3
Low-middle SDI	Stomach cancer	122088 (119484 to 124655)	136618 (132677 to 140958)	142382	163468	16.6	17.3	-22	11.9

© 2018 Fitzmaurice C et al. JAMA Oncology.

Location		Incidence cases, No.		Expected incidence cases, 2016, No.		Change in incidence cases, 1990 to 2016, %			Overall change, %
		2006	2016	Given population growth alone	Given population growth and aging	Due to population growth	Due to change in age structure	Due to change in incidence rate	
Low SDI	Stomach cancer	20531 (19126 to 21663)	23595 (22200 to 24713)	27162	27907	32.3	3.6	-21	14.9
Global	Colon and rectum cancer	1282348 (1268339 to 1297474)	1715539 (1657957 to 1794936)	1441731	1687411	12.4	19.2	2.2	33.8
High SDI	Colon and rectum cancer	659083 (652434 to 665608)	792174 (769351 to 838150)	694286	813077	5.3	18	-3.2	20.2
High-middle SDI	Colon and rectum cancer	282411 (274326 to 288783)	383658 (367341 to 407234)	313764	363506	11.1	17.6	7.1	35.9
Middle SDI	Colon and rectum cancer	248440 (242662 to 253275)	404279 (385990 to 419500)	266686	334579	7.3	27.3	28.1	62.7
Low-middle SDI	Colon and rectum cancer	78300 (75914 to 82221)	112741 (106546 to 116574)	91315	104709	16.6	17.1	10.3	44
Low SDI	Colon and rectum cancer	16787 (15637 to 19181)	23337 (22545 to 24418)	22210	22888	32.3	4	2.7	39
Global	Liver cancer	732483 (701581 to 746688)	1007763 (953287 to 1042211)	823523	940885	12.4	16	9.1	37.6
High SDI	Liver cancer	138232 (135756 to 140834)	189298 (177696 to 197225)	145615	167624	5.3	15.9	15.7	36.9

© 2018 Fitzmaurice C et al. JAMA Oncology.

Location		Incidence cases, No.		Expected incidence cases, 2016, No.		Change in incidence cases, 1990 to 2016, %			Overall change, %
		2006	2016	Given population growth alone	Given population growth and aging	Due to population growth	Due to change in age structure	Due to change in incidence rate	
High-middle SDI	Liver cancer	130254 (125569 to 134793)	194089 (180351 to 207681)	144714	166017	11.1	16.4	21.6	49
Middle SDI	Liver cancer	357944 (340304 to 367714)	487481 (463750 to 508119)	384232	474882	7.3	25.3	3.5	36.2
Low-middle SDI	Liver cancer	73832 (67819 to 79856)	91647 (83575 to 97266)	86104	98131	16.6	16.3	-8.8	24.1
Low SDI	Liver cancer	23969 (21231 to 25947)	31746 (28098 to 33777)	31712	32518	32.3	3.4	-3.2	32.4
Global	Gallbladder and biliary tract cancer	152248 (141517 to 159461)	183711 (168547 to 193180)	171171	201386	12.4	19.8	-11.6	20.7
High SDI	Gallbladder and biliary tract cancer	61398 (58776 to 63059)	72183 (68266 to 76642)	64676	77365	5.3	20.7	-8.4	17.6
High-middle SDI	Gallbladder and biliary tract cancer	28458 (25935 to 29730)	32726 (28817 to 35200)	31616	37005	11.1	18.9	-15	15
Middle SDI	Gallbladder and biliary tract cancer	33669 (29849 to 36924)	42850 (37142 to 46736)	36141	45948	7.3	29.1	-9.2	27.3
Low-middle SDI	Gallbladder and biliary tract cancer	24983 (22141 to 27891)	31439 (27789 to 34896)	29135	33580	16.6	17.8	-8.6	25.8

© 2018 Fitzmaurice C et al. JAMA Oncology.

Location		Incidence cases, No.		Expected incidence cases, 2016, No.		Change in incidence cases, 1990 to 2016, %			Overall change, %
		2006	2016	Given population growth alone	Given population growth and aging	Due to population growth	Due to change in age structure	Due to change in incidence rate	
Low SDI	Gallbladder and biliary tract cancer	4052 (3431 to 4878)	4990 (4389 to 5771)	5360	5500	32.3	3.4	-12.6	23.1
Global	Pancreatic cancer	312537 (310229 to 314889)	417680 (405672 to 425430)	351381	413049	12.4	19.7	1.5	33.6
High SDI	Pancreatic cancer	147245 (145232 to 148989)	192036 (182535 to 198237)	155109	182964	5.3	18.9	6.2	30.4
High-middle SDI	Pancreatic cancer	68565 (67476 to 69638)	88260 (84925 to 90768)	76177	88654	11.1	18.2	-0.6	28.7
Middle SDI	Pancreatic cancer	62675 (61609 to 63869)	89108 (88045 to 90133)	67277	85532	7.3	29.1	5.7	42.2
Low-middle SDI	Pancreatic cancer	27893 (27369 to 28401)	39197 (38464 to 40066)	32529	37684	16.6	18.5	5.4	40.5
Low SDI	Pancreatic cancer	6933 (6771 to 7126)	9879 (9597 to 10169)	9172	9427	32.3	3.7	6.5	42.5
Global	Larynx cancer	153392 (151195 to 155828)	186987 (183907 to 191334)	172456	200231	12.4	18.1	-8.6	21.9
High SDI	Larynx cancer	45558 (44717 to 46472)	50478 (49151 to 51788)	47991	54665	5.3	14.6	-9.2	10.8

© 2018 Fitzmaurice C et al. JAMA Oncology.

Location		Incidence cases, No.		Expected incidence cases, 2016, No.		Change in incidence cases, 1990 to 2016, %			Overall change, %
		2006	2016	Given population growth alone	Given population growth and aging	Due to population growth	Due to change in age structure	Due to change in incidence rate	
High-middle SDI	Larynx cancer	38381 (37469 to 39385)	43618 (42199 to 45040)	42641	49872	11.1	18.8	-16.3	13.6
Middle SDI	Larynx cancer	35090 (34551 to 35651)	49224 (47652 to 51000)	37666	47770	7.3	28.8	4.1	40.3
Low-middle SDI	Larynx cancer	31326 (30006 to 32878)	39949 (38478 to 41915)	36533	42189	16.6	18.1	-7.2	27.5
Low SDI	Larynx cancer	4066 (3790 to 4431)	4862 (4564 to 5301)	5380	5506	32.3	3.1	-15.9	19.6
Global	Tracheal, bronchus, and lung cancer	1563344 (1545583 to 1579252)	2007770 (1958410 to 2055318)	1757652	2047220	12.4	18.5	-2.5	28.4
High SDI	Tracheal, bronchus, and lung cancer	618407 (611960 to 624620)	746752 (732332 to 763525)	651438	756467	5.3	17	-1.6	20.8
High-middle SDI	Tracheal, bronchus, and lung cancer	329399 (323215 to 335892)	402342 (387257 to 419756)	365968	424612	11.1	17.8	-6.8	22.1
Middle SDI	Tracheal, bronchus, and lung cancer	482624 (472274 to 491832)	667965 (641926 to 691611)	518068	657801	7.3	29	2.1	38.4
Low-middle SDI	Tracheal, bronchus, and lung cancer	114595 (110588 to 119003)	159990 (153662 to 165364)	133642	154112	16.6	17.9	5.1	39.6

© 2018 Fitzmaurice C et al. JAMA Oncology.

Location		Incidence cases, No.		Expected incidence cases, 2016, No.		Change in incidence cases, 1990 to 2016, %			Overall change, %
		2006	2016	Given population growth alone	Given population growth and aging	Due to population growth	Due to change in age structure	Due to change in incidence rate	
Low SDI	Tracheal, bronchus, and lung cancer	15268 (13904 to 17287)	22486 (21148 to 24302)	20200	20721	32.3	3.4	11.6	47.3
Global	Malignant skin melanoma	202984 (178679 to 226918)	281528 (243064 to 314164)	228213	258780	12.4	15.1	11.2	38.7
High SDI	Malignant skin melanoma	155350 (134742 to 174853)	211113 (179804 to 235108)	163647	179122	5.3	10	20.6	35.9
High-middle SDI	Malignant skin melanoma	31950 (28104 to 36834)	44383 (38783 to 50361)	35496	40099	11.1	14.4	13.4	38.9
Middle SDI	Malignant skin melanoma	11023 (9867 to 12291)	18691 (16328 to 20556)	11832	14011	7.3	19.8	42.4	69.6
Low-middle SDI	Malignant skin melanoma	3704 (3237 to 4245)	5763 (5121 to 6630)	4319	4886	16.6	15.3	23.7	55.6
Low SDI	Malignant skin melanoma	1847 (1548 to 2143)	2645 (2237 to 3015)	2443	2508	32.3	3.5	7.4	43.2
Global	Non-melanoma skin cancer	1353929 (998622 to 1788464)	1521185 (1108989 to 2008485)	1522209	1795065	12.4	20.2	-20.2	12.4
High SDI	Non-melanoma skin cancer	904058 (650656 to 1199302)	910232 (623182 to 1240450)	952347	1113532	5.3	17.8	-22.5	0.7

© 2018 Fitzmaurice C et al. JAMA Oncology.

Location		Incidence cases, No.		Expected incidence cases, 2016, No.		Change in incidence cases, 1990 to 2016, %			Overall change, %
		2006	2016	Given population growth alone	Given population growth and aging	Due to population growth	Due to change in age structure	Due to change in incidence rate	
High-middle SDI	Non-melanoma skin cancer	165696 (130926 to 214683)	226750 (176393 to 289391)	184091	213777	11.1	17.9	7.8	36.8
Middle SDI	Non-melanoma skin cancer	221182 (169545 to 290848)	295218 (227972 to 379945)	237425	301946	7.3	29.2	-3	33.5
Low-middle SDI	Non-melanoma skin cancer	47504 (33300 to 68778)	66253 (46450 to 95777)	55400	63398	16.6	16.8	6	39.5
Low SDI	Non-melanoma skin cancer	13588 (9119 to 20470)	19721 (13128 to 30031)	17977	18475	32.3	3.7	9.2	45.1
Global	Non-melanoma skin cancer (squamous-cell carcinoma)	606710 (381513 to 918728)	635051 (385690 to 922345)	682117	815484	12.4	22	-29.7	4.7
High SDI	Non-melanoma skin cancer (squamous-cell carcinoma)	451221 (277363 to 672768)	456001 (256933 to 667176)	475321	565708	5.3	20	-24.3	1.1
High-middle SDI	Non-melanoma skin cancer (squamous-cell carcinoma)	48593 (32494 to 86907)	60117 (43765 to 93928)	53987	63441	11.1	19.5	-6.8	23.7
Middle SDI	Non-melanoma skin cancer (squamous-cell carcinoma)	89383 (56899 to 142182)	95714 (66538 to 138977)	95946	125201	7.3	32.7	-33	7.1

© 2018 Fitzmaurice C et al. JAMA Oncology.

Location		Incidence cases, No.		Expected incidence cases, 2016, No.		Change in incidence cases, 1990 to 2016, %			Overall change, %
		2006	2016	Given population growth alone	Given population growth and aging	Due to population growth	Due to change in age structure	Due to change in incidence rate	
Low-middle SDI	Non-melanoma skin cancer (squamous-cell carcinoma)	12678 (9286 to 20211)	16961 (13333 to 22661)	14785	17240	16.6	19.4	-2.2	33.8
Low SDI	Non-melanoma skin cancer (squamous-cell carcinoma)	3193 (2614 to 4029)	4516 (3577 to 5726)	4223	4378	32.3	4.8	4.3	41.5
Global	Non-melanoma skin cancer (basal-cell carcinoma)	747220 (500135 to 1055698)	886134 (574092 to 1261531)	840091	979580	12.4	18.7	-12.5	18.6
High SDI	Non-melanoma skin cancer (basal-cell carcinoma)	452838 (286960 to 671515)	454231 (273336 to 687116)	477025	547824	5.3	15.6	-20.7	0.3
High-middle SDI	Non-melanoma skin cancer (basal-cell carcinoma)	117103 (91369 to 148773)	166633 (120372 to 222374)	130103	150336	11.1	17.3	13.9	42.3
Middle SDI	Non-melanoma skin cancer (basal-cell carcinoma)	131799 (93184 to 176447)	199504 (139154 to 267877)	141478	176745	7.3	26.8	17.3	51.4
Low-middle SDI	Non-melanoma skin cancer (basal-cell carcinoma)	34826 (21496 to 54930)	49292 (29545 to 78033)	40615	46157	16.6	15.9	9	41.5
Low SDI	Non-melanoma skin cancer (basal-cell carcinoma)	10396 (6078 to 17253)	15204 (8916 to 25134)	13753	14096	32.3	3.3	10.7	46.3

© 2018 Fitzmaurice C et al. JAMA Oncology.

Location		Incidence cases, No.		Expected incidence cases, 2016, No.		Change in incidence cases, 1990 to 2016, %			Overall change, %
		2006	2016	Given population growth alone	Given population growth and aging	Due to population growth	Due to change in age structure	Due to change in incidence rate	
Global	Breast cancer	1319274 (1285179 to 1352139)	1702217 (1628609 to 1801186)	1483246	1690669	12.4	15.7	0.9	29
High SDI	Breast cancer	634531 (623997 to 644279)	726622 (707997 to 760448)	668423	745249	5.3	12.1	-2.9	14.5
High-middle SDI	Breast cancer	261237 (251472 to 269066)	332276 (305903 to 360083)	290238	332291	11.1	16.1	0	27.2
Middle SDI	Breast cancer	271736 (254436 to 283258)	414854 (379649 to 448538)	291692	349643	7.3	21.3	24	52.7
Low-middle SDI	Breast cancer	127133 (116375 to 146272)	190102 (173698 to 226022)	148265	167725	16.6	15.3	17.6	49.5
Low SDI	Breast cancer	26373 (21876 to 32887)	37663 (34077 to 42226)	34891	35741	32.3	3.2	7.3	42.8
Global	Cervical cancer	469434 (388864 to 492090)	511169 (413969 to 542277)	527779	579003	12.4	10.9	-14.5	8.9
High SDI	Cervical cancer	63464 (61782 to 64936)	63061 (60702 to 66504)	66854	70406	5.3	5.6	-11.6	-0.6
High-middle SDI	Cervical cancer	76774 (71182 to 80347)	78705 (68459 to 85768)	85297	94484	11.1	12	-20.6	2.5

© 2018 Fitzmaurice C et al. JAMA Oncology.

Location		Incidence cases, No.		Expected incidence cases, 2016, No.		Change in incidence cases, 1990 to 2016, %			Overall change, %
		2006	2016	Given population growth alone	Given population growth and aging	Due to population growth	Due to change in age structure	Due to change in incidence rate	
Middle SDI	Cervical cancer	156970 (122826 to 164021)	173512 (131220 to 187481)	168498	195559	7.3	17.2	-14	10.5
Low-middle SDI	Cervical cancer	112841 (88616 to 125449)	124698 (99160 to 135478)	131598	147605	16.6	14.2	-20.3	10.5
Low SDI	Cervical cancer	58431 (41321 to 71478)	69662 (48584 to 82632)	77306	79305	32.3	3.4	-16.5	19.2
Global	Uterine cancer	297330 (290529 to 303992)	417353 (400753 to 441738)	334285	386447	12.4	17.5	10.4	40.4
High SDI	Uterine cancer	135573 (132594 to 138281)	188007 (180895 to 198633)	142813	162096	5.3	14.2	19.1	38.7
High-middle SDI	Uterine cancer	81840 (77176 to 86613)	116595 (107295 to 129213)	90925	105456	11.1	17.8	13.6	42.5
Middle SDI	Uterine cancer	57679 (55429 to 59583)	82586 (79035 to 87925)	61914	75761	7.3	24	11.8	43.2
Low-middle SDI	Uterine cancer	18625 (16938 to 19870)	25635 (24112 to 26973)	21720	25015	16.6	17.7	3.3	37.6
Low SDI	Uterine cancer	4753 (4296 to 5165)	6162 (5701 to 6599)	6288	6432	32.3	3	-5.7	29.6

© 2018 Fitzmaurice C et al. JAMA Oncology.

Location		Incidence cases, No.		Expected incidence cases, 2016, No.		Change in incidence cases, 1990 to 2016, %			Overall change, %
		2006	2016	Given population growth alone	Given population growth and aging	Due to population growth	Due to change in age structure	Due to change in incidence rate	
Global	Ovarian cancer	204292 (199800 to 208804)	253547 (242388 to 260154)	229683	260083	12.4	14.9	-3.2	24.1
High SDI	Ovarian cancer	78458 (76932 to 80010)	85509 (80303 to 89115)	82648	92895	5.3	13.1	-9.4	9
High-middle SDI	Ovarian cancer	44965 (43481 to 46476)	52717 (48738 to 55292)	49957	56503	11.1	14.6	-8.4	17.2
Middle SDI	Ovarian cancer	46339 (44967 to 48441)	64087 (61799 to 66011)	49742	59022	7.3	20	10.9	38.3
Low-middle SDI	Ovarian cancer	27996 (26748 to 29820)	41059 (39181 to 42815)	32649	36748	16.6	14.6	15.4	46.7
Low SDI	Ovarian cancer	7037 (5714 to 7451)	10636 (8689 to 11362)	9310	9522	32.3	3	15.8	51.1
Global	Prostate cancer	1024737 (941906 to 1133813)	1435742 (1293396 to 1618655)	1152101	1360610	12.4	20.3	7.3	40.1
High SDI	Prostate cancer	686576 (627394 to 774210)	899318 (836795 to 1065763)	723248	851446	5.3	18.7	7	31
High-middle SDI	Prostate cancer	152657 (143626 to 178410)	226521 (205520 to 255438)	169605	196941	11.1	17.9	19.4	48.4

© 2018 Fitzmaurice C et al. JAMA Oncology.

Location		Incidence cases, No.		Expected incidence cases, 2016, No.		Change in incidence cases, 1990 to 2016, %			Overall change, %
		2006	2016	Given population growth alone	Given population growth and aging	Due to population growth	Due to change in age structure	Due to change in incidence rate	
Middle SDI	Prostate cancer	119754 (101660 to 134274)	207679 (178146 to 237617)	128549	167105	7.3	32.2	33.9	73.4
Low-middle SDI	Prostate cancer	47737 (32584 to 55265)	74721 (51286 to 82781)	55671	65261	16.6	20.1	19.8	56.5
Low SDI	Prostate cancer	20214 (11526 to 23402)	29805 (16591 to 34971)	26743	27715	32.3	4.8	10.3	47.4
Global	Testicular cancer	51202 (50063 to 52400)	66833 (64487 to 69736)	57565	58744	12.4	2.3	15.8	30.5
High SDI	Testicular cancer	29422 (28417 to 30391)	34681 (32921 to 36935)	30993	29738	5.3	-4.3	16.8	17.9
High-middle SDI	Testicular cancer	11199 (10758 to 11673)	15610 (14831 to 16376)	12441	12739	11.1	2.7	25.6	39.4
Middle SDI	Testicular cancer	6982 (6768 to 7280)	11740 (11334 to 12177)	7494	7715	7.3	3.2	57.6	68.1
Low-middle SDI	Testicular cancer	3269 (3101 to 3471)	4198 (3965 to 4480)	3812	4035	16.6	6.8	5	28.4
Low SDI	Testicular cancer	538 (479 to 613)	651 (590 to 734)	711	730	32.3	3.5	-14.8	21

© 2018 Fitzmaurice C et al. JAMA Oncology.

Location		Incidence cases, No.		Expected incidence cases, 2016, No.		Change in incidence cases, 1990 to 2016, %			Overall change, %
		2006	2016	Given population growth alone	Given population growth and aging	Due to population growth	Due to change in age structure	Due to change in incidence rate	
Global	Kidney cancer	267959 (263949 to 271723)	342100 (330759 to 349934)	301263	343137	12.4	15.6	-0.4	27.7
High SDI	Kidney cancer	134191 (131951 to 136091)	160805 (154689 to 165708)	141358	160165	5.3	14	0.5	19.8
High-middle SDI	Kidney cancer	65561 (63121 to 68051)	81637 (77842 to 85447)	72839	82789	11.1	15.2	-1.8	24.5
Middle SDI	Kidney cancer	46725 (45882 to 47618)	67625 (65243 to 69419)	50156	59409	7.3	19.8	17.6	44.7
Low-middle SDI	Kidney cancer	17563 (16826 to 18529)	25876 (24799 to 26806)	20482	22559	16.6	11.8	18.9	47.3
Low SDI	Kidney cancer	5162 (4837 to 5561)	7308 (6413 to 8127)	6829	6865	32.3	0.7	8.6	41.6
Global	Bladder cancer	333876 (329918 to 337714)	437442 (426709 to 447912)	375373	441586	12.4	19.8	-1.2	31
High SDI	Bladder cancer	171627 (169324 to 173991)	213500 (206159 to 220038)	180794	213267	5.3	18.9	0.1	24.4
High-middle SDI	Bladder cancer	76815 (75044 to 78668)	96865 (93605 to 100135)	85342	99392	11.1	18.3	-3.3	26.1

© 2018 Fitzmaurice C et al. JAMA Oncology.

Location		Incidence cases, No.		Expected incidence cases, 2016, No.		Change in incidence cases, 1990 to 2016, %			Overall change, %
		2006	2016	Given population growth alone	Given population growth and aging	Due to population growth	Due to change in age structure	Due to change in incidence rate	
Middle SDI	Bladder cancer	56859 (55417 to 58408)	85371 (80381 to 87836)	61034	77034	7.3	28.1	14.7	50.1
Low-middle SDI	Bladder cancer	23940 (22710 to 25170)	34771 (32913 to 36384)	27918	32388	16.6	18.7	10	45.2
Low SDI	Bladder cancer	6011 (5650 to 6672)	8223 (7850 to 8585)	7952	8197	32.3	4.1	0.4	36.8
Global	Brain and nervous system cancer	240728 (218267 to 250141)	329673 (298926 to 348845)	270648	294405	12.4	9.9	14.7	36.9
High SDI	Brain and nervous system cancer	73372 (64706 to 77067)	92681 (74397 to 99558)	77290	83669	5.3	8.7	12.3	26.3
High-middle SDI	Brain and nervous system cancer	53766 (49382 to 56471)	79703 (72810 to 85313)	59734	65196	11.1	10.2	27	48.2
Middle SDI	Brain and nervous system cancer	74017 (63396 to 77735)	105724 (92431 to 114403)	79452	89642	7.3	13.8	21.7	42.8
Low-middle SDI	Brain and nervous system cancer	32304 (28325 to 35122)	41107 (37077 to 46399)	37673	40273	16.6	8	2.6	27.2
Low SDI	Brain and nervous system cancer	7154 (6123 to 8020)	9749 (8616 to 10810)	9465	9552	32.3	1.2	2.7	36.3

© 2018 Fitzmaurice C et al. JAMA Oncology.

Location		Incidence cases, No.		Expected incidence cases, 2016, No.		Change in incidence cases, 1990 to 2016, %			Overall change, %
		2006	2016	Given population growth alone	Given population growth and aging	Due to population growth	Due to change in age structure	Due to change in incidence rate	
Global	Thyroid cancer	159182 (155761 to 162055)	238232 (229319 to 252919)	178967	198574	12.4	12.3	24.9	49.7
High SDI	Thyroid cancer	79105 (75684 to 80968)	100420 (95385 to 106771)	83330	88717	5.3	6.8	14.8	26.9
High-middle SDI	Thyroid cancer	31707 (30627 to 32953)	51421 (48060 to 56019)	35226	39101	11.1	12.2	38.9	62.2
Middle SDI	Thyroid cancer	30899 (29960 to 32409)	58429 (55309 to 63050)	33168	38341	7.3	16.7	65	89.1
Low-middle SDI	Thyroid cancer	14673 (13495 to 15739)	23790 (22883 to 24752)	17111	19077	16.6	13.4	32.1	62.1
Low SDI	Thyroid cancer	3141 (2309 to 3508)	4096 (3041 to 4476)	4155	4254	32.3	3.1	-5	30.4
Global	Mesothelioma	27239 (26149 to 28554)	34760 (32505 to 36461)	30625	35308	12.4	17.2	-2	27.6
High SDI	Mesothelioma	14661 (14322 to 15044)	18331 (17021 to 19127)	15443	17942	5.3	17	2.6	25
High-middle SDI	Mesothelioma	4430 (4281 to 4795)	5477 (5233 to 5712)	4921	5625	11.1	15.9	-3.4	23.6

© 2018 Fitzmaurice C et al. JAMA Oncology.

Location		Incidence cases, No.		Expected incidence cases, 2016, No.		Change in incidence cases, 1990 to 2016, %			Overall change, %
		2006	2016	Given population growth alone	Given population growth and aging	Due to population growth	Due to change in age structure	Due to change in incidence rate	
Middle SDI	Mesothelioma	4494 (4293 to 4787)	6172 (5790 to 6477)	4823	5849	7.3	22.8	7.2	37.3
Low-middle SDI	Mesothelioma	3004 (2474 to 3759)	3954 (3472 to 4607)	3503	3980	16.6	15.9	-0.9	31.6
Low SDI	Mesothelioma	710 (532 to 957)	884 (692 to 1111)	939	962	32.3	3.2	-11.1	24.5
Global	Hodgkin lymphoma	66457 (59597 to 73768)	73323 (65969 to 82334)	74716	78106	12.4	5.1	-7.2	10.3
High SDI	Hodgkin lymphoma	27927 (26983 to 32248)	31252 (30055 to 36939)	29418	29537	5.3	0.4	6.1	11.9
High-middle SDI	Hodgkin lymphoma	12666 (11577 to 13611)	15026 (13159 to 16002)	14072	14570	11.1	3.9	3.6	18.6
Middle SDI	Hodgkin lymphoma	10696 (8591 to 12248)	12784 (10404 to 14780)	11482	12696	7.3	11.4	0.8	19.5
Low-middle SDI	Hodgkin lymphoma	11911 (9511 to 15176)	10845 (8943 to 14387)	13890	14545	16.6	5.5	-31.1	-8.9
Low SDI	Hodgkin lymphoma	3610 (2506 to 4141)	3697 (2526 to 4377)	4776	4836	32.3	1.7	-31.6	2.4

© 2018 Fitzmaurice C et al. JAMA Oncology.

Location		Incidence cases, No.		Expected incidence cases, 2016, No.		Change in incidence cases, 1990 to 2016, %			Overall change, %
		2006	2016	Given population growth alone	Given population growth and aging	Due to population growth	Due to change in age structure	Due to change in incidence rate	
Global	Non-Hodgkin lymphoma	319078 (304377 to 333823)	461164 (427602 to 482023)	358736	407003	12.4	15.1	17	44.5
High SDI	Non-Hodgkin lymphoma	174320 (165667 to 187535)	239770 (225951 to 264248)	183631	208999	5.3	14.6	17.7	37.5
High-middle SDI	Non-Hodgkin lymphoma	45827 (44492 to 49419)	75451 (70037 to 79706)	50914	57261	11.1	13.8	39.7	64.6
Middle SDI	Non-Hodgkin lymphoma	51095 (49421 to 54637)	82157 (73987 to 85368)	54847	64608	7.3	19.1	34.3	60.8
Low-middle SDI	Non-Hodgkin lymphoma	33254 (28538 to 35457)	44491 (38207 to 46667)	38781	42420	16.6	10.9	6.2	33.8
Low SDI	Non-Hodgkin lymphoma	14979 (11132 to 16732)	19777 (15076 to 22196)	19817	19935	32.3	0.8	-1.1	32
Global	Multiple myeloma	100349 (88210 to 111447)	138509 (121000 to 155480)	112821	131674	12.4	18.8	6.8	38
High SDI	Multiple myeloma	57019 (47672 to 64672)	73721 (61316 to 86813)	60064	69849	5.3	17.2	6.8	29.3
High-middle SDI	Multiple myeloma	17075 (15720 to 19458)	25301 (22033 to 27636)	18970	21966	11.1	17.5	19.5	48.2

© 2018 Fitzmaurice C et al. JAMA Oncology.

Location		Incidence cases, No.		Expected incidence cases, 2016, No.		Change in incidence cases, 1990 to 2016, %			Overall change, %
		2006	2016	Given population growth alone	Given population growth and aging	Due to population growth	Due to change in age structure	Due to change in incidence rate	
Middle SDI	Multiple myeloma	14774 (13742 to 16855)	23273 (21136 to 26947)	15858	19848	7.3	27	23.2	57.5
Low-middle SDI	Multiple myeloma	8841 (7574 to 9612)	12525 (11005 to 13977)	10310	11848	16.6	17.4	7.7	41.7
Low SDI	Multiple myeloma	2895 (2135 to 3337)	4052 (3129 to 4528)	3830	3924	32.3	3.3	4.4	40
Global	Leukemia	370482 (344033 to 385104)	466802 (423070 to 488508)	416529	454618	12.4	10.3	3.3	26
High SDI	Leukemia	132201 (130035 to 134403)	173244 (166069 to 180076)	139262	159298	5.3	15.2	10.5	31
High-middle SDI	Leukemia	72840 (65301 to 76218)	103552 (86306 to 112000)	80926	87543	11.1	9.1	22	42.2
Middle SDI	Leukemia	99326 (88729 to 104334)	117494 (102068 to 126403)	106620	114552	7.3	8	3	18.3
Low-middle SDI	Leukemia	53456 (47824 to 61474)	57464 (52287 to 64593)	62341	65453	16.6	5.8	-14.9	7.5
Low SDI	Leukemia	12619 (10697 to 15696)	15096 (13290 to 17299)	16695	16828	32.3	1.1	-13.7	19.6

© 2018 Fitzmaurice C et al. JAMA Oncology.

Location		Incidence cases, No.		Expected incidence cases, 2016, No.		Change in incidence cases, 1990 to 2016, %			Overall change, %
		2006	2016	Given population growth alone	Given population growth and aging	Due to population growth	Due to change in age structure	Due to change in incidence rate	
Global	Acute lymphoid leukemia	58114 (55305 to 65858)	75996 (66466 to 80296)	65336	65594	12.4	0.4	17.9	30.8
High SDI	Acute lymphoid leukemia	14425 (13814 to 15077)	18820 (16919 to 19988)	15195	15308	5.3	0.8	24.3	30.5
High-middle SDI	Acute lymphoid leukemia	10165 (9379 to 11295)	16905 (13770 to 18701)	11293	11240	11.1	-0.5	55.7	66.3
Middle SDI	Acute lymphoid leukemia	18389 (17027 to 20694)	24007 (19493 to 25872)	19739	19914	7.3	1	22.3	30.6
Low-middle SDI	Acute lymphoid leukemia	12442 (10838 to 15886)	12873 (11684 to 15223)	14509	14392	16.6	-0.9	-12.2	3.5
Low SDI	Acute lymphoid leukemia	2619 (2186 to 3822)	3126 (2806 to 3935)	3464	3443	32.3	-0.8	-12.1	19.4
Global	Chronic lymphoid leukemia	76290 (72538 to 83117)	105341 (98310 to 113339)	85771	99912	12.4	18.5	7.1	38.1
High SDI	Chronic lymphoid leukemia	43112 (40240 to 46605)	57151 (53105 to 62599)	45414	53577	5.3	18.9	8.3	32.6
High-middle SDI	Chronic lymphoid leukemia	16357 (15581 to 17600)	23399 (21163 to 25086)	18173	20860	11.1	16.4	15.5	43

© 2018 Fitzmaurice C et al. JAMA Oncology.

Location		Incidence cases, No.		Expected incidence cases, 2016, No.		Change in incidence cases, 1990 to 2016, %			Overall change, %
		2006	2016	Given population growth alone	Given population growth and aging	Due to population growth	Due to change in age structure	Due to change in incidence rate	
Middle SDI	Chronic lymphoid leukemia	10551 (9716 to 12303)	16444 (14746 to 17988)	11326	13297	7.3	18.7	29.8	55.8
Low-middle SDI	Chronic lymphoid leukemia	5633 (4689 to 6876)	7493 (6504 to 9027)	6568	7451	16.6	15.7	0.7	33
Low SDI	Chronic lymphoid leukemia	972 (803 to 1253)	1314 (1122 to 1615)	1286	1319	32.3	3.4	-0.5	35.2
Global	Acute myeloid leukemia	77255 (71056 to 82068)	102968 (91146 to 107607)	86857	95023	12.4	10.6	10.3	33.3
High SDI	Acute myeloid leukemia	34500 (30802 to 36370)	46847 (40087 to 49064)	36342	41189	5.3	14	16.4	35.8
High-middle SDI	Acute myeloid leukemia	14693 (12678 to 15678)	20771 (16706 to 22450)	16324	17483	11.1	7.9	22.4	41.4
Middle SDI	Acute myeloid leukemia	14616 (13409 to 16937)	18645 (17277 to 21227)	15689	16718	7.3	7	13.2	27.6
Low-middle SDI	Acute myeloid leukemia	11306 (9768 to 13557)	13829 (12170 to 15837)	13184	13723	16.6	4.8	0.9	22.3
Low SDI	Acute myeloid leukemia	2381 (2050 to 3163)	3222 (2762 to 3853)	3150	3165	32.3	0.6	2.4	35.3

© 2018 Fitzmaurice C et al. JAMA Oncology.

Location		Incidence cases, No.		Expected incidence cases, 2016, No.		Change in incidence cases, 1990 to 2016, %			Overall change, %
		2006	2016	Given population growth alone	Given population growth and aging	Due to population growth	Due to change in age structure	Due to change in incidence rate	
Global	Chronic myeloid leukemia	30436 (27806 to 32275)	32363 (29258 to 34323)	34219	38358	12.4	13.6	-19.7	6.3
High SDI	Chronic myeloid leukemia	11587 (10833 to 12122)	12095 (10946 to 12780)	12206	13930	5.3	14.9	-15.8	4.4
High-middle SDI	Chronic myeloid leukemia	5851 (5100 to 6133)	5942 (5088 to 6371)	6500	7259	11.1	13	-22.5	1.6
Middle SDI	Chronic myeloid leukemia	5449 (4982 to 5919)	5907 (5378 to 6465)	5849	6701	7.3	15.6	-14.6	8.4
Low-middle SDI	Chronic myeloid leukemia	6237 (5132 to 7633)	6852 (5772 to 8191)	7273	8031	16.6	12.2	-18.9	9.9
Low SDI	Chronic myeloid leukemia	1489 (1233 to 1814)	1780 (1505 to 2125)	1970	2020	32.3	3.4	-16.2	19.5
Global	Other leukemia	128387 (108979 to 135155)	150134 (127058 to 161348)	144344	155730	12.4	8.9	-4.4	16.9
High SDI	Other leukemia	28577 (27172 to 30154)	38330 (35108 to 40548)	30103	35292	5.3	18.2	10.6	34.1
High-middle SDI	Other leukemia	25774 (20477 to 27855)	36536 (26921 to 41204)	28635	30700	11.1	8	22.6	41.8

© 2018 Fitzmaurice C et al. JAMA Oncology.

Location		Incidence cases, No.		Expected incidence cases, 2016, No.		Change in incidence cases, 1990 to 2016, %			Overall change, %
		2006	2016	Given population growth alone	Given population growth and aging	Due to population growth	Due to change in age structure	Due to change in incidence rate	
Middle SDI	Other leukemia	50321 (39849 to 52523)	52491 (42662 to 58493)	54016	57920	7.3	7.8	-10.8	4.3
Low-middle SDI	Other leukemia	17840 (14760 to 20335)	16417 (14407 to 17990)	20805	21853	16.6	5.9	-30.5	-8
Low SDI	Other leukemia	5158 (3864 to 6632)	5654 (4599 to 6821)	6823	6879	32.3	1.1	-23.8	9.6
Global	Other neoplasms	495012 (468793 to 503028)	750253 (681869 to 772487)	556537	617221	12.4	12.3	26.9	51.6
High SDI	Other neoplasms	178743 (174575 to 182261)	247574 (232971 to 258019)	188289	211053	5.3	12.7	20.4	38.5
High-middle SDI	Other neoplasms	90220 (86651 to 93086)	151901 (140796 to 159276)	100236	111193	11.1	12.1	45.1	68.4
Middle SDI	Other neoplasms	121568 (113816 to 124335)	207696 (188189 to 214137)	130495	150948	7.3	16.8	46.7	70.8
Low-middle SDI	Other neoplasms	75690 (66747 to 79046)	105289 (85134 to 109563)	88271	95805	16.6	10	12.5	39.1
Low SDI	Other neoplasms	28958 (24756 to 31115)	36918 (32102 to 39377)	38312	39049	32.3	2.5	-7.4	27.5

© 2018 Fitzmaurice C et al. JAMA Oncology.

© 2018 Fitzmaurice C et al. JAMA Oncology.

eTable 15: Contribution of YLDs and YLLs to DALYs by cancer, global, both sexes, 2016

**Cause**	**Contribution YLLs (%)**	**Contribution YLDs (%)**
Neoplasm	98	2
Lip and Oral Cavity Cancer	97	3
Nasopharynx Cancer	98	2
Other Pharynx Cancer	98	2
Esophageal Cancer	99	1
Stomach Cancer	98	2
Colon and Rectum Cancer	97	3
Liver Cancer	99	1
Gallbladder and Biliary Tract Cancer	99	1
Pancreatic Cancer	99	1
Larynx Cancer	97	3
Tracheal, Bronchus, and Lung Cancer	99	1
Malignant Skin Melanoma	94	6
Non-Melanoma Skin Cancer	97	3
Non-Melanoma Skin Cancer (Squamous-Cell Carcinoma)	97	3
Non-Melanoma Skin Cancer (Basal-Cell Carcinoma)	0	100
Breast Cancer	95	5
Cervical Cancer	97	3
Uterine Cancer	93	7
Ovarian Cancer	97	3
Prostate Cancer	91	9
Testicular Cancer	94	6
Kidney Cancer	96	4
Bladder Cancer	95	5
Brain and Nervous System Cancer	99	1
Thyroid Cancer	93	7
Mesothelioma	98	2

© 2018 Fitzmaurice C et al. JAMA Oncology.

Cause	Contribution YLLs (%)	Contribution YLDs (%)
Hodgkin Lymphoma	97	3
Non-Hodgkin Lymphoma	98	2
Multiple Myeloma	97	3
Leukemia	98	2
Acute Lymphoid Leukemia	99	1
Chronic Lymphoid Leukemia	92	8
Acute Myeloid Leukemia	99	1
Chronic Myeloid Leukemia	98	2
Other Leukemia	98	2
Other Neoplasms	98	2

eTable 16: Probability of developing cancer within selected age intervals, global, and by SDI quintile, by sex, 2006-2016 in % (odds)

		Birth to age 49		Age 50 to 59		Age 60 to 69		Age 70 to 79		Age 30 to 70		Birth to age 79	
**Location/ SDI quintile**	**Cancer**	**Male**	**Female**	**Male**	**Female**	**Male**	**Female**	**Male**	**Female**	**Male**	**Female**	**Male**	**Female**
**Global**	Neoplasms	2.43 (1 in 41)	3.40 (1 in 29)	4.40 (1 in 23)	3.99 (1 in 25)	10.52 (1 in 10)	6.52 (1 in 15)	17.43 (1 in 6)	9.34 (1 in 11)	30.80 (1 in 3)	21.06 (1 in 5)	31.09 (1 in 3)	21.40 (1 in 5)
**Global**	Lip and oral cavity cancer	0.10 (1 in 991)	0.07 (1 in 1417)	0.16 (1 in 629)	0.08 (1 in 1251)	0.25 (1 in 394)	0.13 (1 in 780)	0.33 (1 in 304)	0.17 (1 in 580)	0.83 (1 in 120)	0.44 (1 in 227)	0.84 (1 in 119)	0.45 (1 in 222)
**Global**	Nasopharynx cancer	0.06 (1 in 1584)	0.02 (1 in 4302)	0.04 (1 in 2456)	0.01 (1 in 7131)	0.05 (1 in 1853)	0.02 (1 in 6522)	0.05 (1 in 1906)	0.02 (1 in 6654)	0.20 (1 in 501)	0.06 (1 in 1567)	0.21 (1 in 476)	0.07 (1 in 1479)
**Global**	Other pharynx cancer	0.04 (1 in 2391)	0.02 (1 in 5535)	0.11 (1 in 936)	0.03 (1 in 3434)	0.17 (1 in 606)	0.04 (1 in 2299)	0.16 (1 in 645)	0.04 (1 in 2289)	0.47 (1 in 215)	0.13 (1 in 757)	0.47 (1 in 214)	0.13 (1 in 745)

© 2018 Fitzmaurice C et al. JAMA Oncology.

		Birth to age 49		Age 50 to 59		Age 60 to 69		Age 70 to 79		Age 30 to 70		Birth to age 79	
**Location/ SDI quintile**	**Cancer**	**Male**	**Female**	**Male**	**Female**	**Male**	**Female**	**Male**	**Female**	**Male**	**Female**	**Male**	**Female**
**Global**	Esophageal cancer	0.06 (1 in 1680)	0.02 (1 in 4025)	0.18 (1 in 562)	0.05 (1 in 1899)	0.44 (1 in 227)	0.13 (1 in 758)	0.64 (1 in 156)	0.21 (1 in 480)	1.31 (1 in 76)	0.42 (1 in 241)	1.31 (1 in 76)	0.42 (1 in 240)
**Global**	Stomach cancer	0.15 (1 in 656)	0.11 (1 in 944)	0.37 (1 in 268)	0.16 (1 in 620)	0.97 (1 in 103)	0.35 (1 in 283)	1.63 (1 in 61)	0.63 (1 in 157)	3.09 (1 in 32)	1.24 (1 in 81)	3.10 (1 in 32)	1.25 (1 in 80)
**Global**	Colon and rectum cancer	0.20 (1 in 504)	0.16 (1 in 618)	0.44 (1 in 228)	0.33 (1 in 306)	1.13 (1 in 88)	0.69 (1 in 144)	2.09 (1 in 48)	1.29 (1 in 77)	3.80 (1 in 26)	2.45 (1 in 41)	3.81 (1 in 26)	2.46 (1 in 41)
**Global**	Liver cancer	0.31 (1 in 323)	0.08 (1 in 1272)	0.50 (1 in 202)	0.13 (1 in 756)	0.82 (1 in 122)	0.27 (1 in 370)	1.05 (1 in 95)	0.42 (1 in 236)	2.63 (1 in 38)	0.89 (1 in 112)	2.65 (1 in 38)	0.90 (1 in 111)
**Global**	Gallbladder and biliary tract cancer	0.01 (1 in 7431)	0.02 (1 in 4590)	0.03 (1 in 3257)	0.04 (1 in 2348)	0.08 (1 in 1208)	0.10 (1 in 1020)	0.17 (1 in 577)	0.18 (1 in 570)	0.30 (1 in 334)	0.34 (1 in 297)	0.30 (1 in 333)	0.34 (1 in 296)
**Global**	Pancreatic cancer	0.04 (1 in 2561)	0.02 (1 in 4154)	0.11 (1 in 945)	0.07 (1 in 1497)	0.26 (1 in 380)	0.18 (1 in 550)	0.47 (1 in 212)	0.37 (1 in 270)	0.88 (1 in 114)	0.64 (1 in 156)	0.88 (1 in 114)	0.64 (1 in 156)
**Global**	Larynx cancer	0.03 (1 in 2922)	0.01 (1 in 12880)	0.12 (1 in 857)	0.02 (1 in 6489)	0.22 (1 in 445)	0.03 (1 in 3760)	0.27 (1 in 373)	0.03 (1 in 2909)	0.64 (1 in 156)	0.08 (1 in 1199)	0.64 (1 in 156)	0.08 (1 in 1189)
**Global**	Tracheal, bronchus, and lung cancer	0.19 (1 in 529)	0.11 (1 in 904)	0.66 (1 in 152)	0.28 (1 in 357)	1.80 (1 in 55)	0.67 (1 in 149)	3.09 (1 in 32)	1.14 (1 in 88)	5.63 (1 in 18)	2.18 (1 in 46)	5.64 (1 in 18)	2.18 (1 in 46)

© 2018 Fitzmaurice C et al. JAMA Oncology.

		Birth to age 49		Age 50 to 59		Age 60 to 69		Age 70 to 79		Age 30 to 70		Birth to age 79	
**Location/ SDI quintile**	**Cancer**	**Male**	**Female**	**Male**	**Female**	**Male**	**Female**	**Male**	**Female**	**Male**	**Female**	**Male**	**Female**
**Global**	Malignant skin melanoma	0.07 (1 in 1474)	0.08 (1 in 1252)	0.08 (1 in 1242)	0.06 (1 in 1577)	0.15 (1 in 664)	0.10 (1 in 1044)	0.25 (1 in 394)	0.14 (1 in 721)	0.54 (1 in 184)	0.36 (1 in 275)	0.55 (1 in 181)	0.38 (1 in 265)
**Global**	Non-melanoma skin cancer	0.16 (1 in 625)	0.17 (1 in 579)	0.34 (1 in 297)	0.27 (1 in 376)	0.92 (1 in 108)	0.56 (1 in 177)	1.81 (1 in 55)	1.00 (1 in 100)	3.18 (1 in 31)	1.97 (1 in 51)	3.20 (1 in 31)	1.99 (1 in 50)
**Global**	Non-melanoma skin cancer (squamous-cell carcinoma)	0.03 (1 in 3572)	0.03 (1 in 3609)	0.11 (1 in 879)	0.06 (1 in 1807)	0.45 (1 in 222)	0.18 (1 in 546)	0.98 (1 in 102)	0.40 (1 in 253)	1.57 (1 in 64)	0.66 (1 in 152)	1.57 (1 in 64)	0.66 (1 in 151)
**Global**	Non-melanoma skin cancer (basal-cell carcinoma)	0.13 (1 in 757)	0.14 (1 in 690)	0.22 (1 in 448)	0.21 (1 in 475)	0.47 (1 in 211)	0.38 (1 in 262)	0.83 (1 in 120)	0.61 (1 in 164)	1.64 (1 in 61)	1.32 (1 in 76)	1.65 (1 in 61)	1.34 (1 in 75)
**Global**	Breast cancer	0.01 (1 in 15229)	0.99 (1 in 101)	0.01 (1 in 8639)	1.11 (1 in 90)	0.03 (1 in 3987)	1.41 (1 in 71)	0.03 (1 in 3108)	1.53 (1 in 66)	0.07 (1 in 1339)	4.91 (1 in 20)	0.08 (1 in 1327)	4.95 (1 in 20)
**Global**	Cervical cancer	NA	0.49 (1 in 203)	NA	0.33 (1 in 306)	NA	0.31 (1 in 322)	NA	0.25 (1 in 406)	NA	1.33 (1 in 75)	NA	1.37 (1 in 73)
**Global**	Uterine cancer	NA	0.14 (1 in 715)	NA	0.32 (1 in 310)	NA	0.47 (1 in 215)	NA	0.45 (1 in 221)	NA	1.37 (1 in 73)	NA	1.37 (1 in 73)

© 2018 Fitzmaurice C et al. JAMA Oncology.

		Birth to age 49		Age 50 to 59		Age 60 to 69		Age 70 to 79		Age 30 to 70		Birth to age 79	
**Location/ SDI quintile**	**Cancer**	**Male**	**Female**	**Male**	**Female**	**Male**	**Female**	**Male**	**Female**	**Male**	**Female**	**Male**	**Female**
**Global**	Ovarian cancer	NA	0.15 (1 in 661)	NA	0.16 (1 in 622)	NA	0.21 (1 in 476)	NA	0.24 (1 in 408)	NA	0.74 (1 in 135)	NA	0.76 (1 in 131)
**Global**	Prostate cancer	0.06 (1 in 1609)	NA	0.46 (1 in 219)	NA	1.99 (1 in 50)	NA	3.90 (1 in 26)	NA	6.30 (1 in 16)	NA	6.30 (1 in 16)	NA
**Global**	Testicular cancer	0.10 (1 in 1010)	NA	0.01 (1 in 7006)	NA	0.01 (1 in 8191)	NA	0.01 (1 in 7480)	NA	0.10 (1 in 1018)	NA	0.14 (1 in 720)	NA
**Global**	Kidney cancer	0.07 (1 in 1373)	0.05 (1 in 1963)	0.13 (1 in 768)	0.06 (1 in 1590)	0.24 (1 in 424)	0.12 (1 in 823)	0.36 (1 in 276)	0.19 (1 in 522)	0.79 (1 in 127)	0.41 (1 in 241)	0.80 (1 in 125)	0.43 (1 in 235)
**Global**	Bladder cancer	0.05 (1 in 1961)	0.02 (1 in 5319)	0.14 (1 in 740)	0.04 (1 in 2679)	0.38 (1 in 266)	0.09 (1 in 1169)	0.77 (1 in 130)	0.18 (1 in 561)	1.32 (1 in 76)	0.32 (1 in 315)	1.33 (1 in 75)	0.32 (1 in 313)
**Global**	Brain and nervous system cancer	0.13 (1 in 777)	0.12 (1 in 858)	0.09 (1 in 1174)	0.07 (1 in 1428)	0.14 (1 in 706)	0.11 (1 in 924)	0.18 (1 in 555)	0.14 (1 in 716)	0.49 (1 in 204)	0.39 (1 in 255)	0.53 (1 in 187)	0.43 (1 in 231)
**Global**	Thyroid cancer	0.05 (1 in 1833)	0.14 (1 in 738)	0.06 (1 in 1752)	0.10 (1 in 992)	0.06 (1 in 1694)	0.12 (1 in 860)	0.07 (1 in 1427)	0.11 (1 in 923)	0.23 (1 in 430)	0.44 (1 in 226)	0.24 (1 in 416)	0.46 (1 in 217)
**Global**	Mesotheliom a	0.01 (1 in 18522)	0.00 (1 in 25827)	0.01 (1 in 8363)	0.00 (1 in 21314)	0.03 (1 in 3877)	0.01 (1 in 10536)	0.06 (1 in 1740)	0.02 (1 in 6006)	0.10 (1 in 1000)	0.03 (1 in 2938)	0.10 (1 in 994)	0.03 (1 in 2882)

© 2018 Fitzmaurice C et al. JAMA Oncology.

		Birth to age 49		Age 50 to 59		Age 60 to 69		Age 70 to 79		Age 30 to 70		Birth to age 79	
**Location/ SDI quintile**	**Cancer**	**Male**	**Female**	**Male**	**Female**	**Male**	**Female**	**Male**	**Female**	**Male**	**Female**	**Male**	**Female**
**Global**	Hodgkin lymphoma	0.05 (1 in 2107)	0.03 (1 in 3000)	0.02 (1 in 5211)	0.01 (1 in 11537)	0.03 (1 in 3979)	0.01 (1 in 8661)	0.03 (1 in 3848)	0.01 (1 in 7387)	0.10 (1 in 1031)	0.05 (1 in 1957)	0.12 (1 in 849)	0.07 (1 in 1491)
**Global**	Non-Hodgkin lymphoma	0.12 (1 in 833)	0.08 (1 in 1249)	0.13 (1 in 752)	0.09 (1 in 1147)	0.25 (1 in 404)	0.17 (1 in 579)	0.42 (1 in 241)	0.28 (1 in 352)	0.88 (1 in 114)	0.60 (1 in 166)	0.91 (1 in 110)	0.62 (1 in 161)
**Global**	Multiple myeloma	0.02 (1 in 6032)	0.01 (1 in 8471)	0.04 (1 in 2595)	0.03 (1 in 3424)	0.09 (1 in 1175)	0.07 (1 in 1503)	0.16 (1 in 619)	0.11 (1 in 876)	0.30 (1 in 333)	0.22 (1 in 454)	0.30 (1 in 332)	0.22 (1 in 451)
**Global**	Leukemia	0.14 (1 in 715)	0.10 (1 in 965)	0.10 (1 in 967)	0.07 (1 in 1442)	0.22 (1 in 458)	0.13 (1 in 793)	0.39 (1 in 260)	0.22 (1 in 462)	0.78 (1 in 128)	0.47 (1 in 215)	0.84 (1 in 118)	0.51 (1 in 194)
**Global**	Acute lymphoid leukemia	0.04 (1 in 2737)	0.02 (1 in 4118)	0.01 (1 in 8345)	0.01 (1 in 12545)	0.02 (1 in 5365)	0.01 (1 in 9229)	0.02 (1 in 4311)	0.01 (1 in 7435)	0.07 (1 in 1514)	0.04 (1 in 2503)	0.09 (1 in 1107)	0.06 (1 in 1769)
**Global**	Chronic lymphoid leukemia	0.01 (1 in 7641)	0.01 (1 in 7918)	0.03 (1 in 3493)	0.02 (1 in 5467)	0.07 (1 in 1417)	0.04 (1 in 2497)	0.13 (1 in 779)	0.07 (1 in 1385)	0.24 (1 in 421)	0.14 (1 in 711)	0.24 (1 in 416)	0.14 (1 in 699)
**Global**	Acute myeloid leukemia	0.03 (1 in 3171)	0.02 (1 in 4096)	0.02 (1 in 4480)	0.02 (1 in 6387)	0.05 (1 in 2189)	0.03 (1 in 3495)	0.08 (1 in 1177)	0.05 (1 in 1863)	0.17 (1 in 586)	0.11 (1 in 896)	0.18 (1 in 542)	0.12 (1 in 818)
**Global**	Chronic myeloid leukemia	0.01 (1 in 8306)	0.01 (1 in 11844)	0.01 (1 in 10981)	0.01 (1 in 15080)	0.01 (1 in 7652)	0.01 (1 in 11693)	0.03 (1 in 3948)	0.01 (1 in 7198)	0.06 (1 in 1765)	0.04 (1 in 2826)	0.06 (1 in 1680)	0.04 (1 in 2666)
**Global**	Other leukemia	0.05 (1 in 2139)	0.03 (1 in 2948)	0.03 (1 in 3186)	0.02 (1 in 4807)	0.07 (1 in 1420)	0.04 (1 in 2629)	0.12 (1 in 808)	0.06 (1 in 1578)	0.25 (1 in 400)	0.14 (1 in 717)	0.27 (1 in 367)	0.16 (1 in 641)

© 2018 Fitzmaurice C et al. JAMA Oncology.

		Birth to age 49		Age 50 to 59		Age 60 to 69		Age 70 to 79		Age 30 to 70		Birth to age 79	
**Location/ SDI quintile**	**Cancer**	**Male**	**Female**	**Male**	**Female**	**Male**	**Female**	**Male**	**Female**	**Male**	**Female**	**Male**	**Female**
**Global**	Other neoplasms	0.23 (1 in 440)	0.21 (1 in 467)	0.17 (1 in 574)	0.14 (1 in 703)	0.33 (1 in 307)	0.23 (1 in 433)	0.57 (1 in 175)	0.39 (1 in 259)	1.22 (1 in 82)	0.90 (1 in 111)	1.29 (1 in 77)	0.97 (1 in 103)
**High-middle SDI**	Neoplasms	2.84 (1 in 35)	3.86 (1 in 26)	5.00 (1 in 20)	4.32 (1 in 23)	11.47 (1 in 9)	6.82 (1 in 15)	17.67 (1 in 6)	8.99 (1 in 11)	32.36 (1 in 3)	21.55 (1 in 5)	32.72 (1 in 3)	21.99 (1 in 5)
**High-middle SDI**	Lip and oral cavity cancer	0.06 (1 in 1663)	0.03 (1 in 2875)	0.14 (1 in 723)	0.04 (1 in 2283)	0.22 (1 in 446)	0.07 (1 in 1362)	0.26 (1 in 391)	0.11 (1 in 873)	0.67 (1 in 149)	0.26 (1 in 382)	0.68 (1 in 148)	0.27 (1 in 376)
**High-middle SDI**	Nasopharynx cancer	0.10 (1 in 1014)	0.03 (1 in 3268)	0.06 (1 in 1748)	0.02 (1 in 6004)	0.07 (1 in 1360)	0.02 (1 in 5310)	0.07 (1 in 1485)	0.02 (1 in 5773)	0.28 (1 in 356)	0.08 (1 in 1274)	0.30 (1 in 337)	0.08 (1 in 1199)
**High-middle SDI**	Other pharynx cancer	0.03 (1 in 3209)	0.01 (1 in 14579)	0.11 (1 in 924)	0.01 (1 in 8194)	0.16 (1 in 626)	0.02 (1 in 5699)	0.11 (1 in 945)	0.02 (1 in 6207)	0.40 (1 in 248)	0.05 (1 in 1925)	0.40 (1 in 247)	0.05 (1 in 1897)
**High-middle SDI**	Esophageal cancer	0.05 (1 in 1932)	0.02 (1 in 5688)	0.19 (1 in 532)	0.04 (1 in 2421)	0.47 (1 in 213)	0.12 (1 in 821)	0.67 (1 in 148)	0.20 (1 in 494)	1.38 (1 in 73)	0.38 (1 in 262)	1.38 (1 in 73)	0.38 (1 in 261)
**High-middle SDI**	Stomach cancer	0.16 (1 in 617)	0.11 (1 in 885)	0.43 (1 in 231)	0.18 (1 in 541)	1.19 (1 in 84)	0.42 (1 in 240)	1.90 (1 in 53)	0.74 (1 in 136)	3.62 (1 in 28)	1.43 (1 in 70)	3.63 (1 in 28)	1.44 (1 in 69)
**High-middle SDI**	Colon and rectum cancer	0.24 (1 in 422)	0.20 (1 in 497)	0.54 (1 in 184)	0.41 (1 in 241)	1.45 (1 in 69)	0.87 (1 in 115)	2.57 (1 in 39)	1.49 (1 in 67)	4.71 (1 in 21)	2.94 (1 in 34)	4.73 (1 in 21)	2.95 (1 in 34)
**High-middle SDI**	Liver cancer	0.37 (1 in 267)	0.08 (1 in 1293)	0.54 (1 in 186)	0.13 (1 in 796)	0.84 (1 in 118)	0.25 (1 in 398)	1.05 (1 in 95)	0.37 (1 in 269)	2.76 (1 in 36)	0.82 (1 in 123)	2.78 (1 in 36)	0.82 (1 in 121)

© 2018 Fitzmaurice C et al. JAMA Oncology.

		Birth to age 49		Age 50 to 59		Age 60 to 69		Age 70 to 79		Age 30 to 70		Birth to age 79	
**Location/ SDI quintile**	**Cancer**	**Male**	**Female**	**Male**	**Female**	**Male**	**Female**	**Male**	**Female**	**Male**	**Female**	**Male**	**Female**
**High-middle SDI**	Gallbladder and biliary tract cancer	0.01 (1 in 8122)	0.02 (1 in 5625)	0.03 (1 in 3363)	0.04 (1 in 2490)	0.08 (1 in 1227)	0.10 (1 in 960)	0.16 (1 in 634)	0.18 (1 in 543)	0.28 (1 in 357)	0.34 (1 in 290)	0.28 (1 in 356)	0.35 (1 in 289)
**High-middle SDI**	Pancreatic cancer	0.06 (1 in 1816)	0.03 (1 in 3422)	0.15 (1 in 672)	0.08 (1 in 1236)	0.34 (1 in 296)	0.21 (1 in 472)	0.51 (1 in 195)	0.38 (1 in 264)	1.05 (1 in 95)	0.70 (1 in 143)	1.05 (1 in 95)	0.70 (1 in 143)
**High-middle SDI**	Larynx cancer	0.04 (1 in 2349)	0.01 (1 in 13840)	0.17 (1 in 599)	0.01 (1 in 6941)	0.31 (1 in 326)	0.02 (1 in 4187)	0.33 (1 in 305)	0.03 (1 in 3631)	0.84 (1 in 119)	0.07 (1 in 1385)	0.84 (1 in 119)	0.07 (1 in 1369)
**High-middle SDI**	Tracheal, bronchus, and lung cancer	0.23 (1 in 430)	0.12 (1 in 835)	0.89 (1 in 112)	0.27 (1 in 368)	2.31 (1 in 43)	0.58 (1 in 173)	3.44 (1 in 29)	0.85 (1 in 118)	6.72 (1 in 15)	1.80 (1 in 56)	6.73 (1 in 15)	1.81 (1 in 55)
**High-middle SDI**	Malignant skin melanoma	0.07 (1 in 1533)	0.08 (1 in 1252)	0.07 (1 in 1499)	0.06 (1 in 1632)	0.11 (1 in 917)	0.10 (1 in 1044)	0.16 (1 in 638)	0.13 (1 in 772)	0.39 (1 in 258)	0.35 (1 in 284)	0.40 (1 in 252)	0.37 (1 in 273)
**High-middle SDI**	Non-melanoma skin cancer (squamous-cell carcinoma)	0.02 (1 in 4062)	0.02 (1 in 4316)	0.07 (1 in 1416)	0.03 (1 in 3242)	0.24 (1 in 408)	0.07 (1 in 1393)	0.52 (1 in 193)	0.15 (1 in 677)	0.85 (1 in 117)	0.27 (1 in 368)	0.86 (1 in 117)	0.27 (1 in 366)
**High-middle SDI**	Non-melanoma skin cancer (basal-cell carcinoma)	0.11 (1 in 882)	0.13 (1 in 755)	0.21 (1 in 467)	0.21 (1 in 475)	0.50 (1 in 202)	0.43 (1 in 235)	0.89 (1 in 112)	0.72 (1 in 138)	1.69 (1 in 59)	1.47 (1 in 68)	1.70 (1 in 59)	1.48 (1 in 67)

© 2018 Fitzmaurice C et al. JAMA Oncology.

		Birth to age 49		Age 50 to 59		Age 60 to 69		Age 70 to 79		Age 30 to 70		Birth to age 79	
**Location/ SDI quintile**	**Cancer**	**Male**	**Female**	**Male**	**Female**	**Male**	**Female**	**Male**	**Female**	**Male**	**Female**	**Male**	**Female**
**High-middle SDI**	Breast cancer	0.01 (1 in 16887)	1.13 (1 in 88)	0.01 (1 in 9983)	1.17 (1 in 85)	0.02 (1 in 4511)	1.42 (1 in 71)	0.03 (1 in 3480)	1.39 (1 in 72)	0.07 (1 in 1513)	4.97 (1 in 20)	0.07 (1 in 1496)	5.02 (1 in 20)
**High-middle SDI**	Cervical cancer	NA	0.43 (1 in 235)	NA	0.25 (1 in 395)	NA	0.24 (1 in 415)	NA	0.21 (1 in 486)	NA	1.09 (1 in 92)	NA	1.12 (1 in 89)
**High-middle SDI**	Uterine cancer	NA	0.23 (1 in 429)	NA	0.50 (1 in 198)	NA	0.67 (1 in 149)	NA	0.55 (1 in 180)	NA	1.94 (1 in 52)	NA	1.95 (1 in 51)
**High-middle SDI**	Ovarian cancer	NA	0.18 (1 in 564)	NA	0.19 (1 in 539)	NA	0.24 (1 in 418)	NA	0.25 (1 in 407)	NA	0.82 (1 in 122)	NA	0.84 (1 in 118)
**High-middle SDI**	Prostate cancer	0.07 (1 in 1492)	NA	0.39 (1 in 255)	NA	1.72 (1 in 58)	NA	3.45 (1 in 29)	NA	5.54 (1 in 18)	NA	5.55 (1 in 18)	NA
**High-middle SDI**	Testicular cancer	0.13 (1 in 748)	NA	0.02 (1 in 6624)	NA	0.02 (1 in 5655)	NA	0.02 (1 in 4851)	NA	0.13 (1 in 780)	NA	0.19 (1 in 535)	NA
**High-middle SDI**	Kidney cancer	0.10 (1 in 958)	0.07 (1 in 1376)	0.19 (1 in 520)	0.09 (1 in 1141)	0.33 (1 in 307)	0.17 (1 in 593)	0.39 (1 in 255)	0.22 (1 in 446)	1.00 (1 in 100)	0.53 (1 in 188)	1.01 (1 in 99)	0.55 (1 in 181)
**High-middle SDI**	Bladder cancer	0.06 (1 in 1588)	0.02 (1 in 4868)	0.18 (1 in 542)	0.04 (1 in 2558)	0.52 (1 in 193)	0.09 (1 in 1135)	0.98 (1 in 103)	0.18 (1 in 568)	1.73 (1 in 58)	0.32 (1 in 311)	1.73 (1 in 58)	0.32 (1 in 309)
**High-middle SDI**	Brain and nervous system	0.19 (1 in 529)	0.19 (1 in 527)	0.11 (1 in 910)	0.10 (1 in 1025)	0.18 (1 in 564)	0.14 (1 in 718)	0.21 (1 in 473)	0.16 (1 in 630)	0.61 (1 in 163)	0.51 (1 in 196)	0.69 (1 in 146)	0.58 (1 in 171)

© 2018 Fitzmaurice C et al. JAMA Oncology.

		Birth to age 49		Age 50 to 59		Age 60 to 69		Age 70 to 79		Age 30 to 70		Birth to age 79	
**Location/ SDI quintile**	**Cancer**	**Male**	**Female**	**Male**	**Female**	**Male**	**Female**	**Male**	**Female**	**Male**	**Female**	**Male**	**Female**
	cancer												
**High-middle SDI**	Thyroid cancer	0.07 (1 in 1484)	0.19 (1 in 524)	0.06 (1 in 1798)	0.12 (1 in 802)	0.05 (1 in 2027)	0.13 (1 in 752)	0.05 (1 in 1959)	0.11 (1 in 948)	0.21 (1 in 473)	0.53 (1 in 190)	0.22 (1 in 448)	0.55 (1 in 181)
**High-middle SDI**	Mesothelioma	0.01 (1 in 14905)	0.00 (1 in 22172)	0.01 (1 in 8210)	0.01 (1 in 15635)	0.02 (1 in 5808)	0.01 (1 in 8585)	0.03 (1 in 3386)	0.02 (1 in 5731)	0.06 (1 in 1543)	0.04 (1 in 2548)	0.07 (1 in 1524)	0.04 (1 in 2500)
**High-middle SDI**	Hodgkin lymphoma	0.06 (1 in 1740)	0.05 (1 in 2046)	0.02 (1 in 4507)	0.01 (1 in 12074)	0.03 (1 in 3502)	0.01 (1 in 9919)	0.03 (1 in 3984)	0.01 (1 in 8950)	0.11 (1 in 939)	0.05 (1 in 1911)	0.13 (1 in 750)	0.08 (1 in 1276)
**High-middle SDI**	Non-Hodgkin lymphoma	0.13 (1 in 759)	0.09 (1 in 1060)	0.13 (1 in 760)	0.09 (1 in 1127)	0.22 (1 in 457)	0.16 (1 in 619)	0.31 (1 in 327)	0.22 (1 in 463)	0.75 (1 in 133)	0.54 (1 in 187)	0.79 (1 in 127)	0.56 (1 in 179)
**High-middle SDI**	Multiple myeloma	0.02 (1 in 4624)	0.01 (1 in 7037)	0.04 (1 in 2294)	0.03 (1 in 3060)	0.09 (1 in 1120)	0.07 (1 in 1450)	0.14 (1 in 721)	0.10 (1 in 1051)	0.29 (1 in 345)	0.21 (1 in 479)	0.29 (1 in 342)	0.21 (1 in 474)
**High-middle SDI**	Leukemia	0.20 (1 in 505)	0.15 (1 in 678)	0.15 (1 in 686)	0.09 (1 in 1065)	0.28 (1 in 362)	0.15 (1 in 647)	0.40 (1 in 249)	0.21 (1 in 474)	0.92 (1 in 109)	0.53 (1 in 189)	1.02 (1 in 98)	0.61 (1 in 165)
**High-middle SDI**	Acute lymphoid leukemia	0.05 (1 in 2023)	0.03 (1 in 2952)	0.01 (1 in 6993)	0.01 (1 in 10607)	0.02 (1 in 4641)	0.01 (1 in 8178)	0.03 (1 in 3982)	0.01 (1 in 7386)	0.08 (1 in 1322)	0.04 (1 in 2277)	0.11 (1 in 906)	0.07 (1 in 1448)
**High-middle SDI**	Chronic lymphoid leukemia	0.02 (1 in 4904)	0.02 (1 in 5422)	0.04 (1 in 2292)	0.03 (1 in 3749)	0.09 (1 in 1099)	0.05 (1 in 1917)	0.13 (1 in 763)	0.08 (1 in 1307)	0.28 (1 in 356)	0.17 (1 in 588)	0.29 (1 in 350)	0.17 (1 in 576)

© 2018 Fitzmaurice C et al. JAMA Oncology.

		Birth to age 49		Age 50 to 59		Age 60 to 69		Age 70 to 79		Age 30 to 70		Birth to age 79	
**Location/ SDI quintile**	**Cancer**	**Male**	**Female**	**Male**	**Female**	**Male**	**Female**	**Male**	**Female**	**Male**	**Female**	**Male**	**Female**
**High-middle SDI**	Acute myeloid leukemia	0.04 (1 in 2340)	0.04 (1 in 2686)	0.03 (1 in 3657)	0.02 (1 in 4854)	0.05 (1 in 2199)	0.03 (1 in 3127)	0.07 (1 in 1464)	0.04 (1 in 2292)	0.16 (1 in 612)	0.12 (1 in 868)	0.18 (1 in 544)	0.13 (1 in 750)
**High-middle SDI**	Chronic myeloid leukemia	0.01 (1 in 8011)	0.01 (1 in 12011)	0.01 (1 in 9951)	0.01 (1 in 13435)	0.01 (1 in 6856)	0.01 (1 in 10317)	0.02 (1 in 4127)	0.01 (1 in 7676)	0.06 (1 in 1714)	0.04 (1 in 2765)	0.06 (1 in 1630)	0.04 (1 in 2598)
**High-middle SDI**	Other leukemia	0.07 (1 in 1368)	0.05 (1 in 2013)	0.05 (1 in 1979)	0.03 (1 in 3361)	0.10 (1 in 964)	0.05 (1 in 2060)	0.15 (1 in 654)	0.06 (1 in 1550)	0.34 (1 in 291)	0.17 (1 in 602)	0.38 (1 in 263)	0.19 (1 in 520)
**High-middle SDI**	Other neoplasms	0.27 (1 in 364)	0.29 (1 in 350)	0.20 (1 in 491)	0.16 (1 in 624)	0.37 (1 in 270)	0.25 (1 in 398)	0.59 (1 in 170)	0.38 (1 in 262)	1.34 (1 in 75)	0.97 (1 in 103)	1.43 (1 in 70)	1.07 (1 in 93)
**High SDI**	Neoplasms	4.06 (1 in 25)	5.73 (1 in 17)	7.47 (1 in 13)	6.82 (1 in 15)	17.53 (1 in 6)	10.93 (1 in 9)	27.52 (1 in 4)	15.03 (1 in 7)	46.51 (1 in 2)	33.02 (1 in 3)	46.94 (1 in 2)	33.51 (1 in 3)
**High SDI**	Lip and oral cavity cancer	0.10 (1 in 1045)	0.04 (1 in 2256)	0.19 (1 in 524)	0.06 (1 in 1618)	0.28 (1 in 352)	0.10 (1 in 1010)	0.34 (1 in 296)	0.15 (1 in 648)	0.90 (1 in 111)	0.35 (1 in 283)	0.91 (1 in 110)	0.36 (1 in 279)
**High SDI**	Nasopharynx cancer	0.03 (1 in 3169)	0.01 (1 in 8988)	0.02 (1 in 4097)	0.01 (1 in 12361)	0.03 (1 in 3255)	0.01 (1 in 16647)	0.04 (1 in 2758)	0.01 (1 in 17432)	0.12 (1 in 846)	0.03 (1 in 3519)	0.12 (1 in 814)	0.03 (1 in 3230)
**High SDI**	Other pharynx cancer	0.05 (1 in 2022)	0.01 (1 in 9395)	0.17 (1 in 604)	0.03 (1 in 3370)	0.23 (1 in 442)	0.04 (1 in 2233)	0.17 (1 in 580)	0.04 (1 in 2641)	0.61 (1 in 163)	0.12 (1 in 818)	0.61 (1 in 163)	0.12 (1 in 814)
**High SDI**	Esophageal cancer	0.05 (1 in 1898)	0.01 (1 in 9011)	0.17 (1 in 588)	0.03 (1 in 3493)	0.38 (1 in 266)	0.06 (1 in 1626)	0.55 (1 in 182)	0.10 (1 in 955)	1.14 (1 in 88)	0.21 (1 in 487)	1.14 (1 in 87)	0.21 (1 in 486)

© 2018 Fitzmaurice C et al. JAMA Oncology.

		Birth to age 49		Age 50 to 59		Age 60 to 69		Age 70 to 79		Age 30 to 70		Birth to age 79	
**Location/ SDI quintile**	**Cancer**	**Male**	**Female**	**Male**	**Female**	**Male**	**Female**	**Male**	**Female**	**Male**	**Female**	**Male**	**Female**
**High SDI**	Stomach cancer	0.13 (1 in 775)	0.10 (1 in 1000)	0.30 (1 in 328)	0.13 (1 in 748)	0.79 (1 in 126)	0.28 (1 in 353)	1.66 (1 in 60)	0.62 (1 in 161)	2.86 (1 in 35)	1.13 (1 in 89)	2.86 (1 in 35)	1.13 (1 in 88)
**High SDI**	Colon and rectum cancer	0.35 (1 in 286)	0.30 (1 in 332)	0.90 (1 in 111)	0.62 (1 in 161)	2.11 (1 in 47)	1.21 (1 in 82)	3.66 (1 in 27)	2.15 (1 in 47)	6.86 (1 in 15)	4.21 (1 in 24)	6.87 (1 in 15)	4.22 (1 in 24)
**High SDI**	Liver cancer	0.13 (1 in 769)	0.04 (1 in 2673)	0.35 (1 in 282)	0.08 (1 in 1261)	0.64 (1 in 156)	0.18 (1 in 550)	1.04 (1 in 96)	0.40 (1 in 249)	2.14 (1 in 47)	0.69 (1 in 144)	2.15 (1 in 47)	0.70 (1 in 143)
**High SDI**	Gallbladder and biliary tract cancer	0.01 (1 in 7005)	0.01 (1 in 7787)	0.04 (1 in 2690)	0.03 (1 in 2890)	0.13 (1 in 770)	0.11 (1 in 895)	0.30 (1 in 335)	0.25 (1 in 408)	0.48 (1 in 209)	0.40 (1 in 248)	0.48 (1 in 209)	0.40 (1 in 248)
**High SDI**	Pancreatic cancer	0.05 (1 in 1841)	0.03 (1 in 2932)	0.18 (1 in 570)	0.11 (1 in 921)	0.44 (1 in 225)	0.30 (1 in 332)	0.82 (1 in 122)	0.63 (1 in 158)	1.49 (1 in 67)	1.07 (1 in 93)	1.49 (1 in 67)	1.07 (1 in 93)
**High SDI**	Larynx cancer	0.03 (1 in 2997)	0.01 (1 in 15179)	0.14 (1 in 705)	0.02 (1 in 4782)	0.26 (1 in 378)	0.03 (1 in 3072)	0.31 (1 in 318)	0.03 (1 in 2939)	0.75 (1 in 133)	0.09 (1 in 1072)	0.75 (1 in 133)	0.09 (1 in 1063)
**High SDI**	Tracheal, bronchus, and lung cancer	0.19 (1 in 537)	0.16 (1 in 623)	0.83 (1 in 120)	0.54 (1 in 184)	2.31 (1 in 43)	1.22 (1 in 82)	4.16 (1 in 24)	1.91 (1 in 52)	7.33 (1 in 14)	3.78 (1 in 26)	7.33 (1 in 14)	3.78 (1 in 26)
**High SDI**	Malignant skin melanoma	0.34 (1 in 292)	0.42 (1 in 236)	0.31 (1 in 319)	0.24 (1 in 420)	0.52 (1 in 194)	0.29 (1 in 346)	0.73 (1 in 137)	0.34 (1 in 293)	1.83 (1 in 55)	1.20 (1 in 83)	1.89 (1 in 53)	1.29 (1 in 78)

© 2018 Fitzmaurice C et al. JAMA Oncology.

		Birth to age 49		Age 50 to 59		Age 60 to 69		Age 70 to 79		Age 30 to 70		Birth to age 79	
**Location/ SDI quintile**	**Cancer**	**Male**	**Female**	**Male**	**Female**	**Male**	**Female**	**Male**	**Female**	**Male**	**Female**	**Male**	**Female**
**High SDI**	Non-melanoma skin cancer (squamous-cell carcinoma)	0.08 (1 in 1319)	0.09 (1 in 1120)	0.38 (1 in 262)	0.18 (1 in 549)	1.32 (1 in 76)	0.57 (1 in 176)	2.42 (1 in 41)	1.05 (1 in 96)	4.14 (1 in 24)	1.87 (1 in 54)	4.15 (1 in 24)	1.88 (1 in 53)
**High SDI**	Non-melanoma skin cancer (basal-cell carcinoma)	0.38 (1 in 264)	0.40 (1 in 249)	0.55 (1 in 182)	0.50 (1 in 199)	1.02 (1 in 98)	0.79 (1 in 127)	1.55 (1 in 65)	1.06 (1 in 94)	3.41 (1 in 29)	2.67 (1 in 37)	3.46 (1 in 29)	2.73 (1 in 37)
**High SDI**	Breast cancer	0.01 (1 in 10052)	1.93 (1 in 52)	0.02 (1 in 5862)	2.22 (1 in 45)	0.04 (1 in 2686)	2.89 (1 in 35)	0.05 (1 in 1904)	2.93 (1 in 34)	0.11 (1 in 870)	9.55 (1 in 10)	0.12 (1 in 857)	9.61 (1 in 10)
**High SDI**	Cervical cancer	NA	0.37 (1 in 270)	NA	0.18 (1 in 546)	NA	0.18 (1 in 564)	NA	0.16 (1 in 609)	NA	0.85 (1 in 117)	NA	0.89 (1 in 112)
**High SDI**	Uterine cancer	NA	0.25 (1 in 394)	NA	0.66 (1 in 152)	NA	0.98 (1 in 102)	NA	0.89 (1 in 113)	NA	2.74 (1 in 36)	NA	2.75 (1 in 36)
**High SDI**	Ovarian cancer	NA	0.21 (1 in 487)	NA	0.24 (1 in 425)	NA	0.34 (1 in 296)	NA	0.40 (1 in 247)	NA	1.15 (1 in 87)	NA	1.18 (1 in 85)
**High SDI**	Prostate cancer	0.19 (1 in 522)	NA	1.47 (1 in 68)	NA	5.27 (1 in 19)	NA	8.15 (1 in 12)	NA	14.43 (1 in 7)	NA	14.44 (1 in 7)	NA

© 2018 Fitzmaurice C et al. JAMA Oncology.

		Birth to age 49		Age 50 to 59		Age 60 to 69		Age 70 to 79		Age 30 to 70		Birth to age 79	
**Location/ SDI quintile**	**Cancer**	**Male**	**Female**	**Male**	**Female**	**Male**	**Female**	**Male**	**Female**	**Male**	**Female**	**Male**	**Female**
**High SDI**	Testicular cancer	0.43 (1 in 232)	NA	0.04 (1 in 2226)	NA	0.02 (1 in 4538)	NA	0.01 (1 in 7198)	NA	0.33 (1 in 306)	NA	0.51 (1 in 195)	NA
**High SDI**	Kidney cancer	0.16 (1 in 639)	0.10 (1 in 1029)	0.29 (1 in 349)	0.13 (1 in 760)	0.52 (1 in 194)	0.25 (1 in 408)	0.75 (1 in 133)	0.38 (1 in 263)	1.69 (1 in 59)	0.83 (1 in 120)	1.70 (1 in 59)	0.85 (1 in 117)
**High SDI**	Bladder cancer	0.08 (1 in 1227)	0.04 (1 in 2800)	0.27 (1 in 375)	0.07 (1 in 1388)	0.71 (1 in 140)	0.16 (1 in 618)	1.39 (1 in 72)	0.32 (1 in 316)	2.43 (1 in 41)	0.58 (1 in 172)	2.44 (1 in 41)	0.58 (1 in 171)
**High SDI**	Brain and nervous system cancer	0.21 (1 in 478)	0.18 (1 in 552)	0.12 (1 in 808)	0.10 (1 in 1045)	0.20 (1 in 503)	0.15 (1 in 669)	0.26 (1 in 384)	0.20 (1 in 503)	0.71 (1 in 140)	0.55 (1 in 181)	0.79 (1 in 127)	0.62 (1 in 160)
**High SDI**	Thyroid cancer	0.15 (1 in 658)	0.31 (1 in 324)	0.14 (1 in 701)	0.21 (1 in 466)	0.14 (1 in 697)	0.24 (1 in 424)	0.13 (1 in 761)	0.19 (1 in 516)	0.55 (1 in 182)	0.92 (1 in 109)	0.57 (1 in 176)	0.95 (1 in 105)
**High SDI**	Mesotheliom a	0.01 (1 in 17019)	0.00 (1 in 29776)	0.02 (1 in 4678)	0.01 (1 in 15619)	0.07 (1 in 1415)	0.02 (1 in 6542)	0.15 (1 in 675)	0.03 (1 in 3403)	0.25 (1 in 407)	0.05 (1 in 1853)	0.25 (1 in 407)	0.05 (1 in 1837)
**High SDI**	Hodgkin lymphoma	0.16 (1 in 611)	0.13 (1 in 759)	0.04 (1 in 2577)	0.02 (1 in 4956)	0.04 (1 in 2561)	0.02 (1 in 4695)	0.04 (1 in 2625)	0.02 (1 in 4407)	0.21 (1 in 487)	0.13 (1 in 792)	0.28 (1 in 358)	0.20 (1 in 511)
**High SDI**	Non-Hodgkin lymphoma	0.26 (1 in 380)	0.18 (1 in 565)	0.32 (1 in 314)	0.22 (1 in 455)	0.57 (1 in 176)	0.40 (1 in 250)	0.94 (1 in 106)	0.64 (1 in 156)	2.03 (1 in 49)	1.40 (1 in 71)	2.08 (1 in 48)	1.43 (1 in 70)

© 2018 Fitzmaurice C et al. JAMA Oncology.

		Birth to age 49		Age 50 to 59		Age 60 to 69		Age 70 to 79		Age 30 to 70		Birth to age 79	
**Location/ SDI quintile**	**Cancer**	**Male**	**Female**	**Male**	**Female**	**Male**	**Female**	**Male**	**Female**	**Male**	**Female**	**Male**	**Female**
**High SDI**	Multiple myeloma	0.03 (1 in 3203)	0.02 (1 in 5284)	0.09 (1 in 1149)	0.06 (1 in 1715)	0.19 (1 in 535)	0.13 (1 in 766)	0.36 (1 in 278)	0.24 (1 in 409)	0.66 (1 in 151)	0.45 (1 in 222)	0.66 (1 in 151)	0.45 (1 in 221)
**High SDI**	Leukemia	0.16 (1 in 634)	0.11 (1 in 883)	0.17 (1 in 582)	0.11 (1 in 927)	0.41 (1 in 245)	0.22 (1 in 456)	0.76 (1 in 132)	0.41 (1 in 242)	1.42 (1 in 71)	0.80 (1 in 125)	1.49 (1 in 67)	0.85 (1 in 117)
**High SDI**	Acute lymphoid leukemia	0.06 (1 in 1727)	0.04 (1 in 2560)	0.01 (1 in 6914)	0.01 (1 in 9897)	0.02 (1 in 4729)	0.01 (1 in 7857)	0.03 (1 in 3906)	0.02 (1 in 6025)	0.07 (1 in 1347)	0.05 (1 in 2101)	0.12 (1 in 840)	0.08 (1 in 1274)
**High SDI**	Chronic lymphoid leukemia	0.02 (1 in 5718)	0.01 (1 in 7791)	0.07 (1 in 1499)	0.03 (1 in 2909)	0.17 (1 in 589)	0.08 (1 in 1270)	0.30 (1 in 335)	0.15 (1 in 656)	0.55 (1 in 182)	0.28 (1 in 362)	0.55 (1 in 181)	0.28 (1 in 360)
**High SDI**	Acute myeloid leukemia	0.04 (1 in 2272)	0.04 (1 in 2706)	0.05 (1 in 2115)	0.04 (1 in 2790)	0.11 (1 in 915)	0.07 (1 in 1458)	0.20 (1 in 492)	0.12 (1 in 801)	0.39 (1 in 257)	0.25 (1 in 393)	0.40 (1 in 248)	0.27 (1 in 376)
**High SDI**	Chronic myeloid leukemia	0.02 (1 in 6406)	0.01 (1 in 10839)	0.02 (1 in 6421)	0.01 (1 in 10660)	0.02 (1 in 4405)	0.01 (1 in 7608)	0.05 (1 in 2012)	0.02 (1 in 4060)	0.10 (1 in 992)	0.05 (1 in 1829)	0.10 (1 in 966)	0.06 (1 in 1774)
**High SDI**	Other leukemia	0.02 (1 in 4400)	0.02 (1 in 6584)	0.03 (1 in 3591)	0.02 (1 in 5472)	0.09 (1 in 1167)	0.05 (1 in 2156)	0.18 (1 in 546)	0.10 (1 in 1048)	0.31 (1 in 322)	0.17 (1 in 589)	0.32 (1 in 313)	0.18 (1 in 571)
**High SDI**	Other neoplasms	0.36 (1 in 275)	0.40 (1 in 252)	0.21 (1 in 470)	0.22 (1 in 462)	0.40 (1 in 248)	0.34 (1 in 296)	0.83 (1 in 121)	0.60 (1 in 166)	1.67 (1 in 60)	1.41 (1 in 71)	1.80 (1 in 56)	1.54 (1 in 65)
**Low-middle SDI**	Neoplasms	1.54 (1 in 65)	2.39 (1 in 42)	2.22 (1 in 45)	2.54 (1 in 39)	4.58 (1 in 22)	3.55 (1 in 28)	6.92 (1 in 14)	4.42 (1 in 23)	14.25 (1 in 7)	12.04 (1 in 8)	14.49 (1 in 7)	12.30 (1 in 8)

© 2018 Fitzmaurice C et al. JAMA Oncology.

		Birth to age 49		Age 50 to 59		Age 60 to 69		Age 70 to 79		Age 30 to 70		Birth to age 79	
**Location/ SDI quintile**	**Cancer**	**Male**	**Female**	**Male**	**Female**	**Male**	**Female**	**Male**	**Female**	**Male**	**Female**	**Male**	**Female**
**Low-middle SDI**	Lip and oral cavity cancer	0.18 (1 in 562)	0.15 (1 in 655)	0.23 (1 in 440)	0.18 (1 in 558)	0.36 (1 in 278)	0.29 (1 in 350)	0.46 (1 in 218)	0.33 (1 in 305)	1.20 (1 in 83)	0.92 (1 in 108)	1.22 (1 in 82)	0.94 (1 in 106)
**Low-middle SDI**	Nasopharynx cancer	0.04 (1 in 2235)	0.02 (1 in 6663)	0.04 (1 in 2762)	0.01 (1 in 7275)	0.05 (1 in 2171)	0.02 (1 in 6556)	0.04 (1 in 2278)	0.02 (1 in 6322)	0.16 (1 in 616)	0.06 (1 in 1745)	0.17 (1 in 586)	0.06 (1 in 1672)
**Low-middle SDI**	Other pharynx cancer	0.07 (1 in 1350)	0.04 (1 in 2319)	0.15 (1 in 673)	0.07 (1 in 1455)	0.26 (1 in 378)	0.11 (1 in 930)	0.31 (1 in 328)	0.12 (1 in 844)	0.79 (1 in 127)	0.33 (1 in 301)	0.79 (1 in 127)	0.34 (1 in 296)
**Low-middle SDI**	Esophageal cancer	0.05 (1 in 1912)	0.03 (1 in 3059)	0.10 (1 in 963)	0.06 (1 in 1746)	0.21 (1 in 481)	0.09 (1 in 1055)	0.29 (1 in 341)	0.15 (1 in 645)	0.65 (1 in 153)	0.34 (1 in 297)	0.66 (1 in 153)	0.34 (1 in 295)
**Low-middle SDI**	Stomach cancer	0.12 (1 in 855)	0.11 (1 in 921)	0.21 (1 in 475)	0.15 (1 in 684)	0.45 (1 in 221)	0.28 (1 in 356)	0.70 (1 in 143)	0.44 (1 in 228)	1.46 (1 in 68)	0.96 (1 in 104)	1.47 (1 in 68)	0.97 (1 in 103)
**Low-middle SDI**	Colon and rectum cancer	0.10 (1 in 960)	0.09 (1 in 1116)	0.14 (1 in 723)	0.15 (1 in 674)	0.32 (1 in 312)	0.25 (1 in 404)	0.52 (1 in 193)	0.44 (1 in 225)	1.06 (1 in 94)	0.92 (1 in 109)	1.08 (1 in 93)	0.93 (1 in 108)
**Low-middle SDI**	Liver cancer	0.13 (1 in 767)	0.05 (1 in 1988)	0.18 (1 in 568)	0.08 (1 in 1289)	0.32 (1 in 311)	0.15 (1 in 668)	0.45 (1 in 223)	0.24 (1 in 423)	1.06 (1 in 95)	0.51 (1 in 197)	1.07 (1 in 93)	0.51 (1 in 195)
**Low-middle SDI**	Gallbladder and biliary tract cancer	0.02 (1 in 6129)	0.04 (1 in 2634)	0.03 (1 in 3083)	0.07 (1 in 1513)	0.06 (1 in 1559)	0.11 (1 in 921)	0.09 (1 in 1140)	0.12 (1 in 803)	0.20 (1 in 500)	0.34 (1 in 298)	0.20 (1 in 499)	0.34 (1 in 297)
**Low-middle SDI**	Pancreatic cancer	0.02 (1 in 4054)	0.02 (1 in 5629)	0.06 (1 in 1730)	0.04 (1 in 2419)	0.13 (1 in 770)	0.10 (1 in 964)	0.21 (1 in 466)	0.18 (1 in 561)	0.43 (1 in 235)	0.34 (1 in 294)	0.43 (1 in 234)	0.34 (1 in 293)

© 2018 Fitzmaurice C et al. JAMA Oncology.

		Birth to age 49		Age 50 to 59		Age 60 to 69		Age 70 to 79		Age 30 to 70		Birth to age 79	
**Location/ SDI quintile**	**Cancer**	**Male**	**Female**	**Male**	**Female**	**Male**	**Female**	**Male**	**Female**	**Male**	**Female**	**Male**	**Female**
**Low-middle SDI**	Larynx cancer	0.04 (1 in 2344)	0.01 (1 in 8241)	0.12 (1 in 859)	0.02 (1 in 5070)	0.22 (1 in 453)	0.03 (1 in 3205)	0.24 (1 in 418)	0.04 (1 in 2248)	0.62 (1 in 162)	0.11 (1 in 936)	0.62 (1 in 162)	0.11 (1 in 930)
**Low-middle SDI**	Tracheal, bronchus, and lung cancer	0.13 (1 in 791)	0.07 (1 in 1460)	0.32 (1 in 309)	0.11 (1 in 948)	0.74 (1 in 136)	0.22 (1 in 465)	1.20 (1 in 84)	0.39 (1 in 259)	2.36 (1 in 42)	0.77 (1 in 130)	2.37 (1 in 42)	0.77 (1 in 129)
**Low-middle SDI**	Malignant skin melanoma	0.01 (1 in 12368)	0.01 (1 in 14093)	0.01 (1 in 12581)	0.01 (1 in 16213)	0.01 (1 in 8906)	0.01 (1 in 9428)	0.02 (1 in 6231)	0.02 (1 in 5864)	0.04 (1 in 2387)	0.04 (1 in 2543)	0.04 (1 in 2309)	0.04 (1 in 2444)
**Low-middle SDI**	Non-melanoma skin cancer (squamous-cell carcinoma)	0.01 (1 in 8518)	0.01 (1 in 11341)	0.02 (1 in 5323)	0.01 (1 in 9766)	0.06 (1 in 1638)	0.02 (1 in 4230)	0.13 (1 in 788)	0.04 (1 in 2328)	0.22 (1 in 463)	0.08 (1 in 1188)	0.22 (1 in 458)	0.09 (1 in 1168)
**Low-middle SDI**	Non-melanoma skin cancer (basal-cell carcinoma)	0.06 (1 in 1721)	0.06 (1 in 1738)	0.07 (1 in 1514)	0.06 (1 in 1622)	0.12 (1 in 864)	0.10 (1 in 1005)	0.18 (1 in 569)	0.14 (1 in 706)	0.41 (1 in 247)	0.35 (1 in 285)	0.42 (1 in 241)	0.36 (1 in 278)
**Low-middle SDI**	Breast cancer	0.01 (1 in 19160)	0.62 (1 in 161)	0.01 (1 in 11729)	0.61 (1 in 164)	0.02 (1 in 5998)	0.63 (1 in 159)	0.02 (1 in 5234)	0.61 (1 in 164)	0.05 (1 in 2037)	2.41 (1 in 41)	0.05 (1 in 2020)	2.45 (1 in 41)
**Low-middle SDI**	Cervical cancer	NA	0.48 (1 in 207)	NA	0.38 (1 in 263)	NA	0.37 (1 in 267)	NA	0.27 (1 in 368)	NA	1.47 (1 in 68)	NA	1.50 (1 in 67)

© 2018 Fitzmaurice C et al. JAMA Oncology.

		Birth to age 49		Age 50 to 59		Age 60 to 69		Age 70 to 79		Age 30 to 70		Birth to age 79	
**Location/ SDI quintile**	**Cancer**	**Male**	**Female**	**Male**	**Female**	**Male**	**Female**	**Male**	**Female**	**Male**	**Female**	**Male**	**Female**
**Low-middle SDI**	Uterine cancer	NA	0.05 (1 in 2120)	NA	0.10 (1 in 1041)	NA	0.14 (1 in 730)	NA	0.13 (1 in 792)	NA	0.40 (1 in 248)	NA	0.41 (1 in 246)
**Low-middle SDI**	Ovarian cancer	NA	0.13 (1 in 792)	NA	0.13 (1 in 746)	NA	0.15 (1 in 663)	NA	0.14 (1 in 700)	NA	0.53 (1 in 187)	NA	0.55 (1 in 181)
**Low-middle SDI**	Prostate cancer	0.02 (1 in 5364)	NA	0.11 (1 in 938)	NA	0.53 (1 in 190)	NA	1.15 (1 in 87)	NA	1.79 (1 in 56)	NA	1.79 (1 in 56)	NA
**Low-middle SDI**	Testicular cancer	0.02 (1 in 4905)	NA	0.00 (1 in 27489)	NA	0.00 (1 in 20556)	NA	0.01 (1 in 13863)	NA	0.03 (1 in 3535)	NA	0.04 (1 in 2770)	NA
**Low-middle SDI**	Kidney cancer	0.03 (1 in 3050)	0.03 (1 in 3861)	0.05 (1 in 2185)	0.03 (1 in 3849)	0.07 (1 in 1378)	0.04 (1 in 2557)	0.09 (1 in 1088)	0.04 (1 in 2262)	0.24 (1 in 421)	0.13 (1 in 774)	0.24 (1 in 412)	0.14 (1 in 740)
**Low-middle SDI**	Bladder cancer	0.03 (1 in 3478)	0.01 (1 in 8843)	0.06 (1 in 1621)	0.02 (1 in 4529)	0.15 (1 in 646)	0.05 (1 in 2066)	0.26 (1 in 377)	0.08 (1 in 1206)	0.51 (1 in 197)	0.16 (1 in 611)	0.51 (1 in 196)	0.16 (1 in 608)
**Low-middle SDI**	Brain and nervous system cancer	0.08 (1 in 1326)	0.06 (1 in 1757)	0.05 (1 in 1971)	0.04 (1 in 2654)	0.07 (1 in 1350)	0.05 (1 in 1923)	0.08 (1 in 1264)	0.06 (1 in 1579)	0.25 (1 in 396)	0.19 (1 in 529)	0.28 (1 in 358)	0.21 (1 in 477)
**Low-middle SDI**	Thyroid cancer	0.02 (1 in 5734)	0.07 (1 in 1503)	0.02 (1 in 5573)	0.05 (1 in 2193)	0.02 (1 in 4256)	0.05 (1 in 2006)	0.03 (1 in 3281)	0.05 (1 in 1940)	0.09 (1 in 1161)	0.20 (1 in 500)	0.09 (1 in 1120)	0.21 (1 in 469)

© 2018 Fitzmaurice C et al. JAMA Oncology.

		Birth to age 49		Age 50 to 59		Age 60 to 69		Age 70 to 79		Age 30 to 70		Birth to age 79	
**Location/ SDI quintile**	**Cancer**	**Male**	**Female**	**Male**	**Female**	**Male**	**Female**	**Male**	**Female**	**Male**	**Female**	**Male**	**Female**
**Low-middle SDI**	Mesothelioma	0.00 (1 in 21896)	0.00 (1 in 25655)	0.01 (1 in 11490)	0.00 (1 in 29155)	0.01 (1 in 8098)	0.01 (1 in 19678)	0.02 (1 in 4850)	0.01 (1 in 13703)	0.05 (1 in 2188)	0.02 (1 in 5250)	0.05 (1 in 2163)	0.02 (1 in 5075)
**Low-middle SDI**	Hodgkin lymphoma	0.03 (1 in 3664)	0.01 (1 in 7537)	0.01 (1 in 7807)	0.01 (1 in 17100)	0.02 (1 in 5760)	0.01 (1 in 10637)	0.02 (1 in 5204)	0.01 (1 in 10258)	0.06 (1 in 1564)	0.03 (1 in 3076)	0.08 (1 in 1304)	0.04 (1 in 2614)
**Low-middle SDI**	Non-Hodgkin lymphoma	0.07 (1 in 1391)	0.05 (1 in 2153)	0.06 (1 in 1744)	0.04 (1 in 2473)	0.10 (1 in 974)	0.08 (1 in 1318)	0.13 (1 in 749)	0.09 (1 in 1152)	0.34 (1 in 294)	0.23 (1 in 426)	0.36 (1 in 274)	0.25 (1 in 401)
**Low-middle SDI**	Multiple myeloma	0.01 (1 in 9611)	0.01 (1 in 10870)	0.02 (1 in 5376)	0.02 (1 in 5568)	0.03 (1 in 2877)	0.04 (1 in 2580)	0.05 (1 in 2048)	0.04 (1 in 2387)	0.11 (1 in 896)	0.11 (1 in 938)	0.11 (1 in 888)	0.11 (1 in 928)
**Low-middle SDI**	Leukemia	0.10 (1 in 993)	0.08 (1 in 1329)	0.05 (1 in 1864)	0.05 (1 in 2212)	0.09 (1 in 1058)	0.07 (1 in 1451)	0.14 (1 in 727)	0.09 (1 in 1075)	0.34 (1 in 296)	0.25 (1 in 399)	0.39 (1 in 259)	0.28 (1 in 354)
**Low-middle SDI**	Acute lymphoid leukemia	0.03 (1 in 3606)	0.02 (1 in 5643)	0.01 (1 in 12144)	0.01 (1 in 17092)	0.01 (1 in 8182)	0.01 (1 in 13324)	0.01 (1 in 6916)	0.01 (1 in 11603)	0.04 (1 in 2254)	0.03 (1 in 3476)	0.06 (1 in 1597)	0.04 (1 in 2519)
**Low-middle SDI**	Chronic lymphoid leukemia	0.00 (1 in 20261)	0.01 (1 in 11572)	0.01 (1 in 10696)	0.01 (1 in 9670)	0.02 (1 in 4956)	0.02 (1 in 4453)	0.03 (1 in 3398)	0.03 (1 in 3745)	0.06 (1 in 1599)	0.07 (1 in 1500)	0.06 (1 in 1566)	0.07 (1 in 1468)
**Low-middle SDI**	Acute myeloid leukemia	0.03 (1 in 3623)	0.02 (1 in 5288)	0.01 (1 in 7175)	0.01 (1 in 11580)	0.02 (1 in 4631)	0.01 (1 in 8682)	0.03 (1 in 3795)	0.02 (1 in 5623)	0.08 (1 in 1295)	0.05 (1 in 2058)	0.09 (1 in 1118)	0.06 (1 in 1759)
**Low-middle SDI**	Chronic myeloid leukemia	0.01 (1 in 7443)	0.01 (1 in 9410)	0.01 (1 in 11961)	0.01 (1 in 12692)	0.01 (1 in 8664)	0.01 (1 in 10928)	0.02 (1 in 6429)	0.01 (1 in 9347)	0.05 (1 in 2189)	0.04 (1 in 2783)	0.05 (1 in 2046)	0.04 (1 in 2608)

© 2018 Fitzmaurice C et al. JAMA Oncology.

		Birth to age 49		Age 50 to 59		Age 60 to 69		Age 70 to 79		Age 30 to 70		Birth to age 79	
**Location/ SDI quintile**	**Cancer**	**Male**	**Female**	**Male**	**Female**	**Male**	**Female**	**Male**	**Female**	**Male**	**Female**	**Male**	**Female**
**Low-middle SDI**	Other leukemia	0.03 (1 in 3704)	0.02 (1 in 5162)	0.01 (1 in 7251)	0.01 (1 in 7994)	0.03 (1 in 3449)	0.02 (1 in 5470)	0.05 (1 in 1926)	0.03 (1 in 3418)	0.11 (1 in 919)	0.07 (1 in 1404)	0.12 (1 in 822)	0.08 (1 in 1259)
**Low-middle SDI**	Other neoplasms	0.15 (1 in 659)	0.12 (1 in 811)	0.13 (1 in 751)	0.10 (1 in 1020)	0.23 (1 in 427)	0.16 (1 in 625)	0.32 (1 in 314)	0.21 (1 in 479)	0.78 (1 in 128)	0.55 (1 in 183)	0.83 (1 in 120)	0.59 (1 in 170)
**Low SDI**	Neoplasms	1.41 (1 in 71)	2.67 (1 in 37)	1.81 (1 in 55)	2.62 (1 in 38)	3.95 (1 in 25)	3.84 (1 in 26)	6.73 (1 in 15)	4.38 (1 in 23)	13.03 (1 in 8)	12.59 (1 in 8)	13.27 (1 in 8)	12.85 (1 in 8)
**Low SDI**	Lip and oral cavity cancer	0.04 (1 in 2362)	0.05 (1 in 2121)	0.06 (1 in 1573)	0.05 (1 in 1862)	0.10 (1 in 995)	0.11 (1 in 905)	0.18 (1 in 543)	0.12 (1 in 857)	0.38 (1 in 260)	0.32 (1 in 311)	0.39 (1 in 256)	0.33 (1 in 305)
**Low SDI**	Nasopharynx cancer	0.03 (1 in 2875)	0.01 (1 in 7745)	0.03 (1 in 3588)	0.02 (1 in 6527)	0.04 (1 in 2470)	0.01 (1 in 6973)	0.03 (1 in 3672)	0.01 (1 in 13533)	0.12 (1 in 814)	0.05 (1 in 2120)	0.13 (1 in 767)	0.05 (1 in 2002)
**Low SDI**	Other pharynx cancer	0.01 (1 in 7367)	0.01 (1 in 7311)	0.03 (1 in 3640)	0.02 (1 in 4714)	0.05 (1 in 2048)	0.03 (1 in 3102)	0.05 (1 in 2036)	0.04 (1 in 2745)	0.14 (1 in 724)	0.10 (1 in 980)	0.14 (1 in 720)	0.10 (1 in 966)
**Low SDI**	Esophageal cancer	0.07 (1 in 1431)	0.03 (1 in 3517)	0.14 (1 in 698)	0.06 (1 in 1761)	0.25 (1 in 393)	0.14 (1 in 713)	0.42 (1 in 237)	0.23 (1 in 437)	0.88 (1 in 113)	0.45 (1 in 221)	0.89 (1 in 113)	0.45 (1 in 220)
**Low SDI**	Stomach cancer	0.09 (1 in 1160)	0.07 (1 in 1342)	0.15 (1 in 667)	0.11 (1 in 949)	0.32 (1 in 317)	0.22 (1 in 447)	0.56 (1 in 178)	0.31 (1 in 319)	1.10 (1 in 91)	0.71 (1 in 141)	1.11 (1 in 90)	0.72 (1 in 140)
**Low SDI**	Colon and rectum cancer	0.08 (1 in 1313)	0.07 (1 in 1342)	0.12 (1 in 856)	0.13 (1 in 768)	0.24 (1 in 415)	0.23 (1 in 430)	0.46 (1 in 217)	0.43 (1 in 234)	0.88 (1 in 113)	0.86 (1 in 117)	0.89 (1 in 112)	0.86 (1 in 116)

© 2018 Fitzmaurice C et al. JAMA Oncology.

		Birth to age 49		Age 50 to 59		Age 60 to 69		Age 70 to 79		Age 30 to 70		Birth to age 79	
**Location/ SDI quintile**	**Cancer**	**Male**	**Female**	**Male**	**Female**	**Male**	**Female**	**Male**	**Female**	**Male**	**Female**	**Male**	**Female**
**Low SDI**	Liver cancer	0.19 (1 in 515)	0.09 (1 in 1065)	0.23 (1 in 431)	0.12 (1 in 841)	0.39 (1 in 259)	0.24 (1 in 412)	0.56 (1 in 177)	0.31 (1 in 323)	1.34 (1 in 74)	0.75 (1 in 133)	1.37 (1 in 73)	0.76 (1 in 131)
**Low SDI**	Gallbladder and biliary tract cancer	0.01 (1 in 12076)	0.02 (1 in 5020)	0.02 (1 in 4355)	0.04 (1 in 2517)	0.04 (1 in 2354)	0.09 (1 in 1155)	0.06 (1 in 1539)	0.10 (1 in 979)	0.14 (1 in 723)	0.25 (1 in 404)	0.14 (1 in 721)	0.25 (1 in 403)
**Low SDI**	Pancreatic cancer	0.03 (1 in 3703)	0.02 (1 in 4461)	0.06 (1 in 1720)	0.05 (1 in 2015)	0.14 (1 in 731)	0.12 (1 in 849)	0.23 (1 in 443)	0.20 (1 in 499)	0.45 (1 in 225)	0.39 (1 in 257)	0.45 (1 in 224)	0.39 (1 in 257)
**Low SDI**	Larynx cancer	0.02 (1 in 5206)	0.01 (1 in 14172)	0.06 (1 in 1751)	0.01 (1 in 8394)	0.12 (1 in 840)	0.02 (1 in 5008)	0.12 (1 in 832)	0.02 (1 in 4183)	0.31 (1 in 318)	0.06 (1 in 1602)	0.32 (1 in 317)	0.06 (1 in 1592)
**Low SDI**	Tracheal, bronchus, and lung cancer	0.08 (1 in 1297)	0.04 (1 in 2324)	0.18 (1 in 548)	0.08 (1 in 1228)	0.40 (1 in 249)	0.18 (1 in 544)	0.68 (1 in 148)	0.27 (1 in 365)	1.33 (1 in 75)	0.58 (1 in 173)	1.33 (1 in 75)	0.58 (1 in 172)
**Low SDI**	Malignant skin melanoma	0.01 (1 in 11122)	0.01 (1 in 10030)	0.01 (1 in 9260)	0.02 (1 in 5841)	0.03 (1 in 3801)	0.04 (1 in 2769)	0.04 (1 in 2797)	0.04 (1 in 2850)	0.08 (1 in 1237)	0.10 (1 in 1032)	0.08 (1 in 1222)	0.10 (1 in 1018)
**Low SDI**	Non-melanoma skin cancer (squamous-cell carcinoma)	0.02 (1 in 5534)	0.01 (1 in 10644)	0.02 (1 in 4111)	0.01 (1 in 8504)	0.07 (1 in 1534)	0.03 (1 in 3838)	0.13 (1 in 757)	0.05 (1 in 2051)	0.24 (1 in 424)	0.09 (1 in 1060)	0.24 (1 in 418)	0.10 (1 in 1042)
**Low SDI**	Non-melanoma skin cancer	0.08 (1 in 1209)	0.07 (1 in 1365)	0.10 (1 in 1012)	0.08 (1 in 1282)	0.15 (1 in 661)	0.11 (1 in 907)	0.21 (1 in 470)	0.14 (1 in 706)	0.53 (1 in 188)	0.39 (1 in 256)	0.54 (1 in 184)	0.40 (1 in 248)

© 2018 Fitzmaurice C et al. JAMA Oncology.

		Birth to age 49		Age 50 to 59		Age 60 to 69		Age 70 to 79		Age 30 to 70		Birth to age 79	
**Location/ SDI quintile**	**Cancer**	**Male**	**Female**	**Male**	**Female**	**Male**	**Female**	**Male**	**Female**	**Male**	**Female**	**Male**	**Female**
	(basal-cell carcinoma)												
**Low SDI**	Breast cancer	0.01 (1 in 16426)	0.48 (1 in 210)	0.01 (1 in 13787)	0.48 (1 in 210)	0.01 (1 in 6678)	0.55 (1 in 181)	0.02 (1 in 5509)	0.51 (1 in 196)	0.05 (1 in 2177)	1.98 (1 in 51)	0.05 (1 in 2152)	2.00 (1 in 50)
**Low SDI**	Cervical cancer	NA	1.04 (1 in 96)	NA	0.80 (1 in 124)	NA	0.87 (1 in 115)	NA	0.61 (1 in 163)	NA	3.23 (1 in 31)	NA	3.29 (1 in 30)
**Low SDI**	Uterine cancer	NA	0.04 (1 in 2471)	NA	0.08 (1 in 1200)	NA	0.15 (1 in 661)	NA	0.15 (1 in 660)	NA	0.42 (1 in 236)	NA	0.43 (1 in 235)
**Low SDI**	Ovarian cancer	NA	0.12 (1 in 853)	NA	0.14 (1 in 738)	NA	0.15 (1 in 650)	NA	0.18 (1 in 549)	NA	0.57 (1 in 175)	NA	0.59 (1 in 170)
**Low SDI**	Prostate cancer	0.03 (1 in 3858)	NA	0.17 (1 in 605)	NA	0.92 (1 in 109)	NA	2.08 (1 in 48)	NA	3.16 (1 in 32)	NA	3.16 (1 in 32)	NA
**Low SDI**	Testicular cancer	0.01 (1 in 9844)	NA	0.00 (1 in 33316)	NA	0.00 (1 in 22247)	NA	0.01 (1 in 16688)	NA	0.02 (1 in 5007)	NA	0.02 (1 in 4229)	NA
**Low SDI**	Kidney cancer	0.03 (1 in 3890)	0.03 (1 in 3997)	0.04 (1 in 2788)	0.03 (1 in 3740)	0.05 (1 in 1925)	0.04 (1 in 2816)	0.07 (1 in 1437)	0.04 (1 in 2681)	0.18 (1 in 564)	0.12 (1 in 847)	0.18 (1 in 547)	0.12 (1 in 803)
**Low SDI**	Bladder cancer	0.03 (1 in 3864)	0.02 (1 in 6406)	0.05 (1 in 2020)	0.03 (1 in 3135)	0.13 (1 in 787)	0.07 (1 in 1453)	0.26 (1 in 385)	0.10 (1 in 964)	0.46 (1 in 217)	0.22 (1 in 457)	0.46 (1 in 217)	0.22 (1 in 455)

© 2018 Fitzmaurice C et al. JAMA Oncology.

		Birth to age 49		Age 50 to 59		Age 60 to 69		Age 70 to 79		Age 30 to 70		Birth to age 79	
**Location/ SDI quintile**	**Cancer**	**Male**	**Female**	**Male**	**Female**	**Male**	**Female**	**Male**	**Female**	**Male**	**Female**	**Male**	**Female**
**Low SDI**	Brain and nervous system cancer	0.05 (1 in 1882)	0.04 (1 in 2336)	0.04 (1 in 2275)	0.03 (1 in 3223)	0.07 (1 in 1458)	0.05 (1 in 2116)	0.07 (1 in 1341)	0.06 (1 in 1627)	0.22 (1 in 450)	0.17 (1 in 596)	0.24 (1 in 417)	0.18 (1 in 548)
**Low SDI**	Thyroid cancer	0.01 (1 in 10628)	0.03 (1 in 2934)	0.01 (1 in 7548)	0.04 (1 in 2651)	0.02 (1 in 4285)	0.04 (1 in 2306)	0.03 (1 in 3734)	0.06 (1 in 1776)	0.07 (1 in 1409)	0.17 (1 in 596)	0.07 (1 in 1374)	0.17 (1 in 584)
**Low SDI**	Mesothelioma	0.00 (1 in 28725)	0.00 (1 in 22394)	0.01 (1 in 15857)	0.00 (1 in 21367)	0.01 (1 in 9895)	0.01 (1 in 14591)	0.02 (1 in 5851)	0.01 (1 in 12092)	0.04 (1 in 2734)	0.02 (1 in 4203)	0.04 (1 in 2704)	0.02 (1 in 4121)
**Low SDI**	Hodgkin lymphoma	0.03 (1 in 3524)	0.02 (1 in 5466)	0.01 (1 in 7369)	0.01 (1 in 11450)	0.02 (1 in 4897)	0.02 (1 in 6589)	0.02 (1 in 4693)	0.01 (1 in 8192)	0.07 (1 in 1376)	0.05 (1 in 2101)	0.08 (1 in 1195)	0.05 (1 in 1838)
**Low SDI**	Non-Hodgkin lymphoma	0.12 (1 in 856)	0.09 (1 in 1170)	0.07 (1 in 1510)	0.05 (1 in 2143)	0.11 (1 in 913)	0.09 (1 in 1120)	0.13 (1 in 777)	0.10 (1 in 984)	0.37 (1 in 267)	0.29 (1 in 343)	0.42 (1 in 238)	0.32 (1 in 310)
**Low SDI**	Multiple myeloma	0.01 (1 in 8332)	0.01 (1 in 6947)	0.02 (1 in 5356)	0.03 (1 in 3582)	0.05 (1 in 2108)	0.07 (1 in 1497)	0.07 (1 in 1536)	0.05 (1 in 1910)	0.14 (1 in 703)	0.16 (1 in 624)	0.14 (1 in 699)	0.16 (1 in 620)
**Low SDI**	Leukemia	0.08 (1 in 1311)	0.07 (1 in 1530)	0.05 (1 in 2173)	0.04 (1 in 2289)	0.09 (1 in 1136)	0.07 (1 in 1406)	0.13 (1 in 762)	0.11 (1 in 946)	0.31 (1 in 326)	0.26 (1 in 387)	0.34 (1 in 293)	0.29 (1 in 350)
**Low SDI**	Acute lymphoid leukemia	0.02 (1 in 6033)	0.01 (1 in 8391)	0.01 (1 in 13249)	0.00 (1 in 20382)	0.01 (1 in 11518)	0.01 (1 in 14516)	0.01 (1 in 9171)	0.01 (1 in 18208)	0.03 (1 in 3011)	0.02 (1 in 4625)	0.04 (1 in 2288)	0.03 (1 in 3425)

© 2018 Fitzmaurice C et al. JAMA Oncology.

		Birth to age 49		Age 50 to 59		Age 60 to 69		Age 70 to 79		Age 30 to 70		Birth to age 79	
**Location/ SDI quintile**	**Cancer**	**Male**	**Female**	**Male**	**Female**	**Male**	**Female**	**Male**	**Female**	**Male**	**Female**	**Male**	**Female**
**Low SDI**	Chronic lymphoid leukemia	0.00 (1 in 56848)	0.00 (1 in 20048)	0.00 (1 in 20469)	0.01 (1 in 15700)	0.02 (1 in 5989)	0.02 (1 in 4295)	0.02 (1 in 4107)	0.03 (1 in 3669)	0.05 (1 in 2123)	0.06 (1 in 1641)	0.05 (1 in 2097)	0.06 (1 in 1616)
**Low SDI**	Acute myeloid leukemia	0.02 (1 in 5444)	0.01 (1 in 7176)	0.01 (1 in 8942)	0.01 (1 in 16332)	0.02 (1 in 5274)	0.01 (1 in 12839)	0.02 (1 in 5419)	0.02 (1 in 6245)	0.06 (1 in 1698)	0.04 (1 in 2699)	0.07 (1 in 1494)	0.04 (1 in 2280)
**Low SDI**	Chronic myeloid leukemia	0.01 (1 in 8098)	0.01 (1 in 8162)	0.01 (1 in 15804)	0.01 (1 in 11920)	0.01 (1 in 8582)	0.01 (1 in 10857)	0.01 (1 in 8395)	0.01 (1 in 7493)	0.04 (1 in 2598)	0.04 (1 in 2458)	0.04 (1 in 2368)	0.04 (1 in 2315)
**Low SDI**	Other leukemia	0.03 (1 in 3674)	0.02 (1 in 4491)	0.02 (1 in 6219)	0.02 (1 in 5585)	0.03 (1 in 3122)	0.02 (1 in 4173)	0.07 (1 in 1524)	0.04 (1 in 2292)	0.13 (1 in 775)	0.10 (1 in 1015)	0.14 (1 in 710)	0.11 (1 in 928)
**Low SDI**	Other neoplasms	0.26 (1 in 378)	0.19 (1 in 518)	0.14 (1 in 706)	0.11 (1 in 934)	0.21 (1 in 481)	0.15 (1 in 646)	0.31 (1 in 327)	0.20 (1 in 488)	0.85 (1 in 118)	0.60 (1 in 166)	0.92 (1 in 109)	0.66 (1 in 152)
**Middle SDI**	Neoplasms	2.46 (1 in 41)	3.13 (1 in 32)	4.06 (1 in 25)	3.34 (1 in 30)	9.17 (1 in 11)	5.21 (1 in 19)	14.35 (1 in 7)	7.18 (1 in 14)	26.88 (1 in 4)	17.25 (1 in 6)	27.20 (1 in 4)	17.62 (1 in 6)
**Middle SDI**	Lip and oral cavity cancer	0.08 (1 in 1261)	0.05 (1 in 2165)	0.13 (1 in 788)	0.05 (1 in 1820)	0.21 (1 in 475)	0.09 (1 in 1062)	0.30 (1 in 328)	0.15 (1 in 681)	0.71 (1 in 140)	0.34 (1 in 298)	0.72 (1 in 139)	0.34 (1 in 293)
**Middle SDI**	Nasopharynx cancer	0.08 (1 in 1304)	0.03 (1 in 3066)	0.05 (1 in 2211)	0.02 (1 in 6349)	0.07 (1 in 1520)	0.02 (1 in 5007)	0.07 (1 in 1479)	0.02 (1 in 4323)	0.24 (1 in 413)	0.09 (1 in 1161)	0.26 (1 in 392)	0.09 (1 in 1094)
**Middle SDI**	Other pharynx cancer	0.03 (1 in 3710)	0.01 (1 in 10236)	0.06 (1 in 1578)	0.02 (1 in 6270)	0.09 (1 in 1118)	0.02 (1 in 4317)	0.09 (1 in 1067)	0.03 (1 in 3906)	0.27 (1 in 367)	0.07 (1 in 1363)	0.27 (1 in 366)	0.07 (1 in 1343)

© 2018 Fitzmaurice C et al. JAMA Oncology.

		Birth to age 49		Age 50 to 59		Age 60 to 69		Age 70 to 79		Age 30 to 70		Birth to age 79	
**Location/ SDI quintile**	**Cancer**	**Male**	**Female**	**Male**	**Female**	**Male**	**Female**	**Male**	**Female**	**Male**	**Female**	**Male**	**Female**
**Middle SDI**	Esophageal cancer	0.07 (1 in 1490)	0.03 (1 in 3622)	0.22 (1 in 447)	0.07 (1 in 1473)	0.62 (1 in 161)	0.21 (1 in 486)	0.94 (1 in 107)	0.34 (1 in 292)	1.84 (1 in 54)	0.64 (1 in 156)	1.84 (1 in 54)	0.64 (1 in 156)
**Middle SDI**	Stomach cancer	0.19 (1 in 524)	0.11 (1 in 917)	0.50 (1 in 200)	0.18 (1 in 553)	1.36 (1 in 73)	0.42 (1 in 236)	2.13 (1 in 47)	0.73 (1 in 136)	4.12 (1 in 24)	1.43 (1 in 70)	4.13 (1 in 24)	1.44 (1 in 69)
**Middle SDI**	Colon and rectum cancer	0.20 (1 in 491)	0.15 (1 in 659)	0.36 (1 in 276)	0.26 (1 in 386)	0.88 (1 in 113)	0.54 (1 in 185)	1.47 (1 in 68)	0.92 (1 in 109)	2.88 (1 in 35)	1.85 (1 in 54)	2.90 (1 in 35)	1.86 (1 in 54)
**Middle SDI**	Liver cancer	0.48 (1 in 208)	0.11 (1 in 904)	0.76 (1 in 131)	0.20 (1 in 509)	1.25 (1 in 80)	0.41 (1 in 243)	1.48 (1 in 68)	0.60 (1 in 167)	3.89 (1 in 26)	1.30 (1 in 77)	3.92 (1 in 26)	1.31 (1 in 76)
**Middle SDI**	Gallbladder and biliary tract cancer	0.01 (1 in 7843)	0.02 (1 in 5801)	0.03 (1 in 3598)	0.03 (1 in 2867)	0.07 (1 in 1489)	0.08 (1 in 1234)	0.13 (1 in 777)	0.14 (1 in 719)	0.24 (1 in 425)	0.27 (1 in 369)	0.24 (1 in 423)	0.27 (1 in 368)
**Middle SDI**	Pancreatic cancer	0.04 (1 in 2717)	0.02 (1 in 4539)	0.08 (1 in 1207)	0.05 (1 in 1837)	0.19 (1 in 513)	0.13 (1 in 748)	0.31 (1 in 324)	0.24 (1 in 421)	0.62 (1 in 161)	0.45 (1 in 224)	0.62 (1 in 161)	0.45 (1 in 224)
**Middle SDI**	Larynx cancer	0.03 (1 in 3604)	0.01 (1 in 17124)	0.09 (1 in 1145)	0.01 (1 in 9074)	0.18 (1 in 570)	0.02 (1 in 4445)	0.23 (1 in 437)	0.03 (1 in 2878)	0.52 (1 in 193)	0.07 (1 in 1363)	0.52 (1 in 193)	0.07 (1 in 1350)
**Middle SDI**	Tracheal, bronchus, and lung cancer	0.23 (1 in 438)	0.12 (1 in 804)	0.70 (1 in 143)	0.27 (1 in 368)	1.98 (1 in 50)	0.66 (1 in 151)	3.32 (1 in 30)	1.11 (1 in 90)	6.10 (1 in 16)	2.14 (1 in 47)	6.11 (1 in 16)	2.15 (1 in 46)

© 2018 Fitzmaurice C et al. JAMA Oncology.

		Birth to age 49		Age 50 to 59		Age 60 to 69		Age 70 to 79		Age 30 to 70		Birth to age 79	
**Location/ SDI quintile**	**Cancer**	**Male**	**Female**	**Male**	**Female**	**Male**	**Female**	**Male**	**Female**	**Male**	**Female**	**Male**	**Female**
**Middle SDI**	Malignant skin melanoma	0.02 (1 in 4734)	0.02 (1 in 5399)	0.02 (1 in 5136)	0.01 (1 in 7787)	0.02 (1 in 4792)	0.02 (1 in 5367)	0.03 (1 in 3177)	0.03 (1 in 3306)	0.09 (1 in 1122)	0.08 (1 in 1323)	0.09 (1 in 1076)	0.08 (1 in 1246)
**Middle SDI**	Non-melanoma skin cancer (squamous-cell carcinoma)	0.02 (1 in 4317)	0.02 (1 in 4629)	0.06 (1 in 1604)	0.03 (1 in 3052)	0.23 (1 in 444)	0.08 (1 in 1221)	0.51 (1 in 197)	0.17 (1 in 587)	0.81 (1 in 123)	0.30 (1 in 329)	0.82 (1 in 123)	0.31 (1 in 326)
**Middle SDI**	Non-melanoma skin cancer (basal-cell carcinoma)	0.11 (1 in 944)	0.12 (1 in 801)	0.17 (1 in 602)	0.16 (1 in 620)	0.34 (1 in 292)	0.27 (1 in 372)	0.59 (1 in 169)	0.42 (1 in 239)	1.19 (1 in 84)	0.96 (1 in 105)	1.20 (1 in 83)	0.97 (1 in 103)
**Middle SDI**	Breast cancer	0.01 (1 in 15063)	0.89 (1 in 112)	0.01 (1 in 8447)	0.88 (1 in 113)	0.02 (1 in 4106)	0.94 (1 in 107)	0.02 (1 in 4089)	0.89 (1 in 112)	0.07 (1 in 1501)	3.51 (1 in 28)	0.07 (1 in 1486)	3.56 (1 in 28)
**Middle SDI**	Cervical cancer	NA	0.48 (1 in 209)	NA	0.34 (1 in 295)	NA	0.33 (1 in 306)	NA	0.28 (1 in 351)	NA	1.39 (1 in 72)	NA	1.42 (1 in 70)
**Middle SDI**	Uterine cancer	NA	0.13 (1 in 764)	NA	0.22 (1 in 461)	NA	0.23 (1 in 442)	NA	0.19 (1 in 532)	NA	0.75 (1 in 133)	NA	0.76 (1 in 132)
**Middle SDI**	Ovarian cancer	NA	0.14 (1 in 694)	NA	0.13 (1 in 783)	NA	0.15 (1 in 680)	NA	0.15 (1 in 648)	NA	0.54 (1 in 184)	NA	0.57 (1 in 175)

© 2018 Fitzmaurice C et al. JAMA Oncology.

		Birth to age 49		Age 50 to 59		Age 60 to 69		Age 70 to 79		Age 30 to 70		Birth to age 79	
**Location/ SDI quintile**	**Cancer**	**Male**	**Female**	**Male**	**Female**	**Male**	**Female**	**Male**	**Female**	**Male**	**Female**	**Male**	**Female**
**Middle SDI**	Prostate cancer	0.04 (1 in 2366)	NA	0.20 (1 in 501)	NA	0.83 (1 in 120)	NA	1.90 (1 in 53)	NA	2.94 (1 in 34)	NA	2.95 (1 in 34)	NA
**Middle SDI**	Testicular cancer	0.05 (1 in 1942)	NA	0.01 (1 in 16097)	NA	0.01 (1 in 11812)	NA	0.01 (1 in 7482)	NA	0.05 (1 in 1892)	NA	0.08 (1 in 1258)	NA
**Middle SDI**	Kidney cancer	0.06 (1 in 1611)	0.05 (1 in 2070)	0.08 (1 in 1235)	0.04 (1 in 2467)	0.12 (1 in 832)	0.07 (1 in 1479)	0.18 (1 in 563)	0.09 (1 in 1127)	0.43 (1 in 233)	0.23 (1 in 434)	0.44 (1 in 227)	0.24 (1 in 408)
**Middle SDI**	Bladder cancer	0.05 (1 in 1885)	0.02 (1 in 5801)	0.10 (1 in 1034)	0.03 (1 in 3640)	0.24 (1 in 422)	0.05 (1 in 1855)	0.43 (1 in 232)	0.11 (1 in 927)	0.81 (1 in 123)	0.20 (1 in 490)	0.82 (1 in 122)	0.21 (1 in 485)
**Middle SDI**	Brain and nervous system cancer	0.14 (1 in 729)	0.13 (1 in 763)	0.08 (1 in 1270)	0.07 (1 in 1497)	0.13 (1 in 750)	0.10 (1 in 981)	0.16 (1 in 617)	0.12 (1 in 805)	0.46 (1 in 219)	0.37 (1 in 271)	0.51 (1 in 196)	0.42 (1 in 236)
**Middle SDI**	Thyroid cancer	0.05 (1 in 2138)	0.11 (1 in 884)	0.04 (1 in 2350)	0.07 (1 in 1430)	0.03 (1 in 3040)	0.07 (1 in 1378)	0.05 (1 in 1901)	0.07 (1 in 1489)	0.17 (1 in 604)	0.30 (1 in 328)	0.17 (1 in 572)	0.32 (1 in 310)
**Middle SDI**	Mesotheliom a	0.01 (1 in 18184)	0.00 (1 in 27415)	0.01 (1 in 10448)	0.00 (1 in 27486)	0.01 (1 in 11013)	0.01 (1 in 14209)	0.02 (1 in 6495)	0.01 (1 in 10076)	0.04 (1 in 2579)	0.02 (1 in 4257)	0.04 (1 in 2529)	0.02 (1 in 4124)
**Middle SDI**	Hodgkin lymphoma	0.02 (1 in 4282)	0.01 (1 in 7342)	0.01 (1 in 8087)	0.00 (1 in 22076)	0.02 (1 in 5178)	0.01 (1 in 15519)	0.02 (1 in 5002)	0.01 (1 in 11877)	0.06 (1 in 1575)	0.03 (1 in 3890)	0.08 (1 in 1333)	0.03 (1 in 3029)

© 2018 Fitzmaurice C et al. JAMA Oncology.

		Birth to age 49		Age 50 to 59		Age 60 to 69		Age 70 to 79		Age 30 to 70		Birth to age 79	
**Location/ SDI quintile**	**Cancer**	**Male**	**Female**	**Male**	**Female**	**Male**	**Female**	**Male**	**Female**	**Male**	**Female**	**Male**	**Female**
**Middle SDI**	Non-Hodgkin lymphoma	0.09 (1 in 1114)	0.06 (1 in 1810)	0.09 (1 in 1100)	0.05 (1 in 2036)	0.15 (1 in 686)	0.09 (1 in 1156)	0.19 (1 in 527)	0.12 (1 in 855)	0.49 (1 in 205)	0.29 (1 in 344)	0.52 (1 in 194)	0.31 (1 in 325)
**Middle SDI**	Multiple myeloma	0.01 (1 in 7487)	0.01 (1 in 11106)	0.03 (1 in 3957)	0.02 (1 in 5366)	0.05 (1 in 2103)	0.04 (1 in 2776)	0.07 (1 in 1473)	0.05 (1 in 2049)	0.15 (1 in 658)	0.11 (1 in 901)	0.15 (1 in 649)	0.11 (1 in 890)
**Middle SDI**	Leukemia	0.16 (1 in 619)	0.12 (1 in 817)	0.08 (1 in 1195)	0.05 (1 in 1851)	0.15 (1 in 681)	0.08 (1 in 1189)	0.21 (1 in 482)	0.11 (1 in 883)	0.51 (1 in 195)	0.31 (1 in 327)	0.60 (1 in 167)	0.37 (1 in 268)
**Middle SDI**	Acute lymphoid leukemia	0.04 (1 in 2411)	0.03 (1 in 3500)	0.01 (1 in 8094)	0.01 (1 in 12800)	0.02 (1 in 4945)	0.01 (1 in 9063)	0.03 (1 in 3730)	0.01 (1 in 7074)	0.07 (1 in 1368)	0.04 (1 in 2423)	0.10 (1 in 992)	0.06 (1 in 1625)
**Middle SDI**	Chronic lymphoid leukemia	0.02 (1 in 6260)	0.01 (1 in 6973)	0.02 (1 in 6286)	0.01 (1 in 8513)	0.03 (1 in 3457)	0.02 (1 in 5413)	0.04 (1 in 2519)	0.02 (1 in 4293)	0.10 (1 in 1048)	0.06 (1 in 1562)	0.10 (1 in 995)	0.07 (1 in 1474)
**Middle SDI**	Acute myeloid leukemia	0.03 (1 in 3492)	0.02 (1 in 4592)	0.01 (1 in 7422)	0.01 (1 in 12542)	0.02 (1 in 5106)	0.01 (1 in 8893)	0.03 (1 in 3624)	0.02 (1 in 6063)	0.07 (1 in 1343)	0.05 (1 in 2186)	0.09 (1 in 1120)	0.06 (1 in 1740)
**Middle SDI**	Chronic myeloid leukemia	0.01 (1 in 10822)	0.01 (1 in 17210)	0.01 (1 in 16172)	0.00 (1 in 26184)	0.01 (1 in 14588)	0.00 (1 in 23024)	0.01 (1 in 9392)	0.01 (1 in 17703)	0.03 (1 in 3277)	0.02 (1 in 5626)	0.03 (1 in 3037)	0.02 (1 in 5097)
**Middle SDI**	Other leukemia	0.07 (1 in 1505)	0.05 (1 in 1925)	0.04 (1 in 2793)	0.02 (1 in 4410)	0.07 (1 in 1404)	0.04 (1 in 2561)	0.10 (1 in 974)	0.05 (1 in 1863)	0.24 (1 in 414)	0.14 (1 in 727)	0.28 (1 in 362)	0.17 (1 in 598)
**Middle SDI**	Other neoplasms	0.21 (1 in 469)	0.20 (1 in 508)	0.17 (1 in 595)	0.12 (1 in 804)	0.32 (1 in 316)	0.20 (1 in 511)	0.50 (1 in 199)	0.30 (1 in 333)	1.12 (1 in 89)	0.74 (1 in 135)	1.20 (1 in 84)	0.81 (1 in 123)

© 2018 Fitzmaurice C et al. JAMA Oncology.

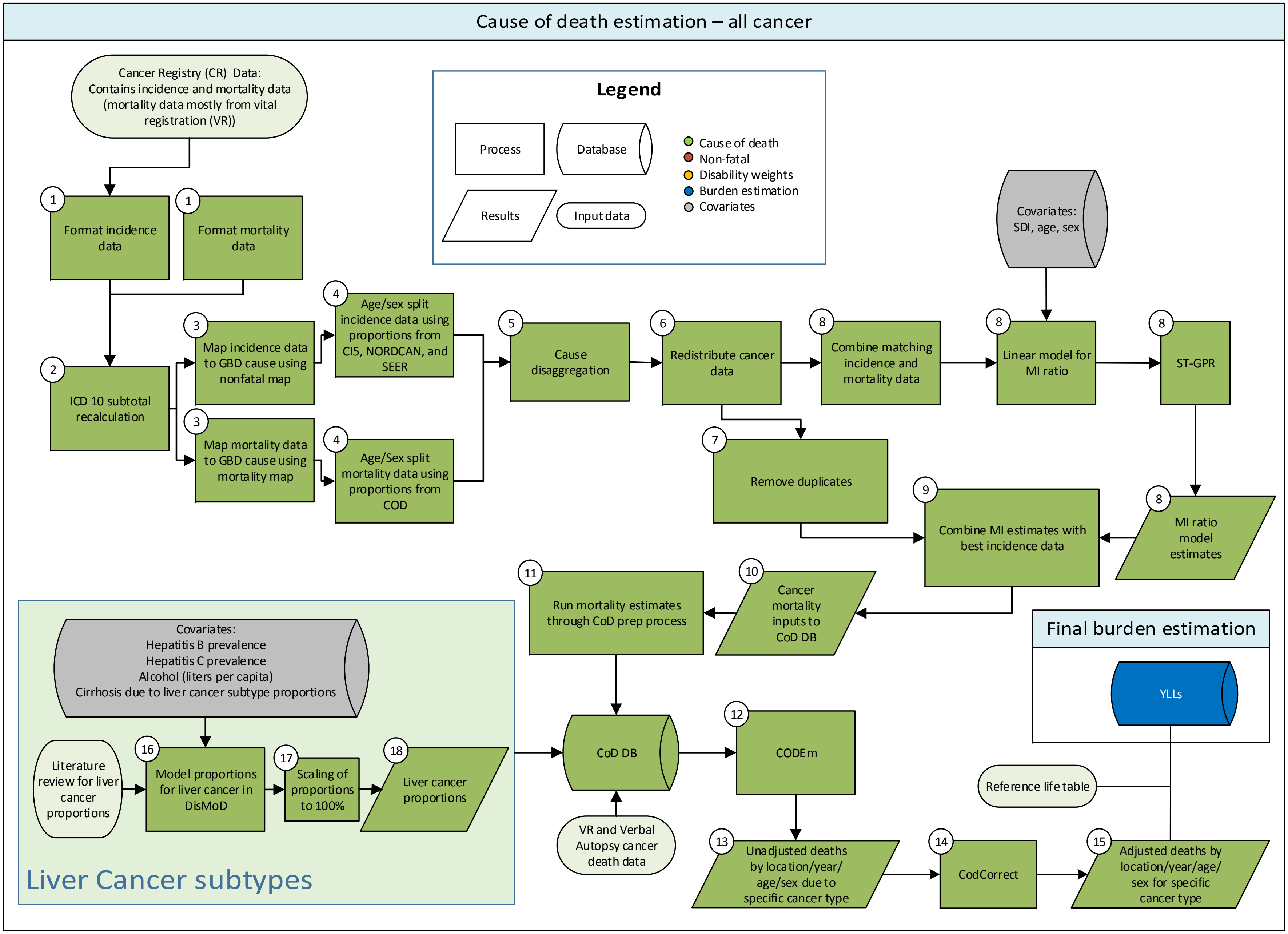

eFigure 1: Flowchart GBD cancer mortality, YLL estimation

© 2018 Fitzmaurice C et al. JAMA Oncology.

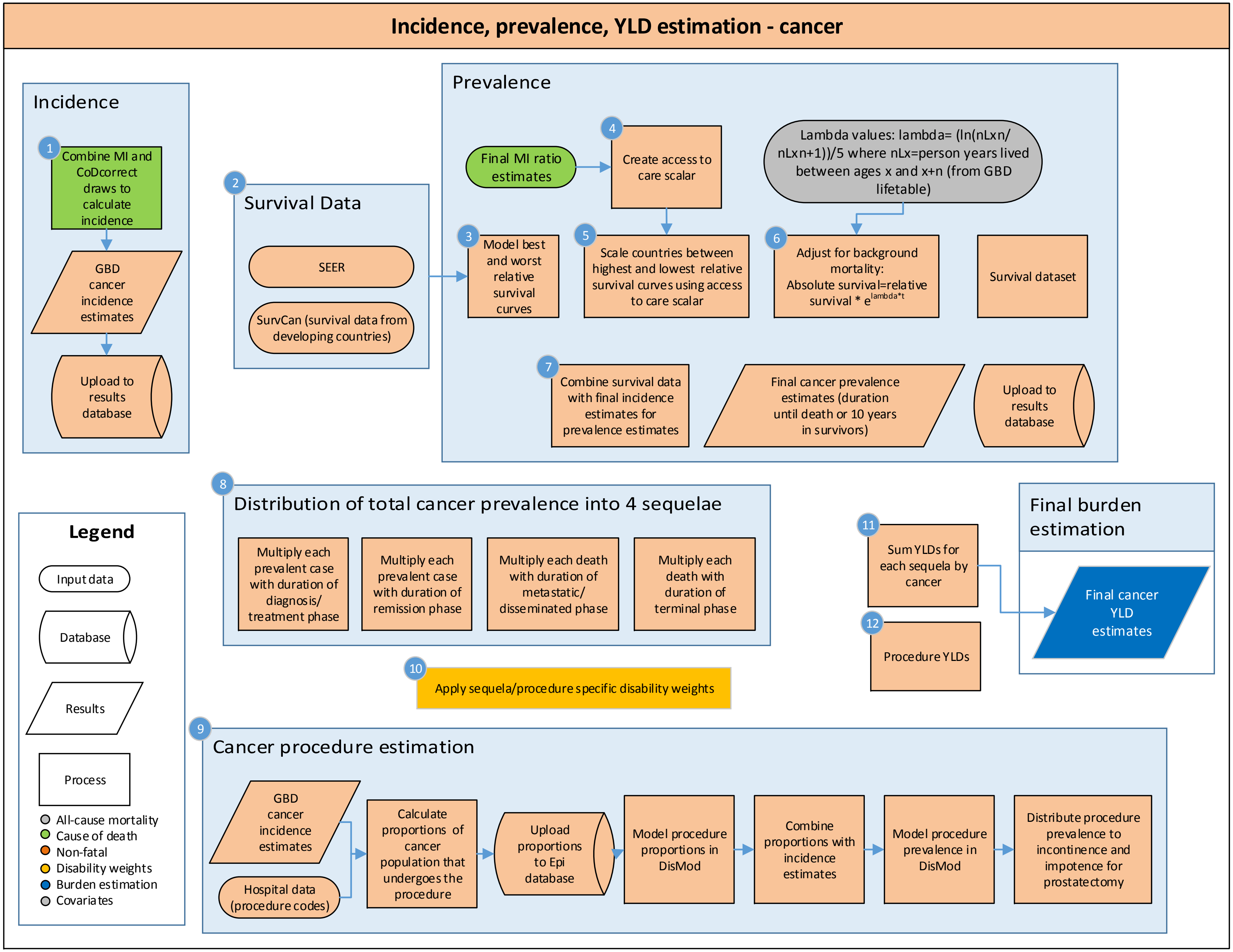

eFigure 2: Flowchart GBD cancer incidence, prevalence, YLD estimation

© 2018 Fitzmaurice C et al. JAMA Oncology.

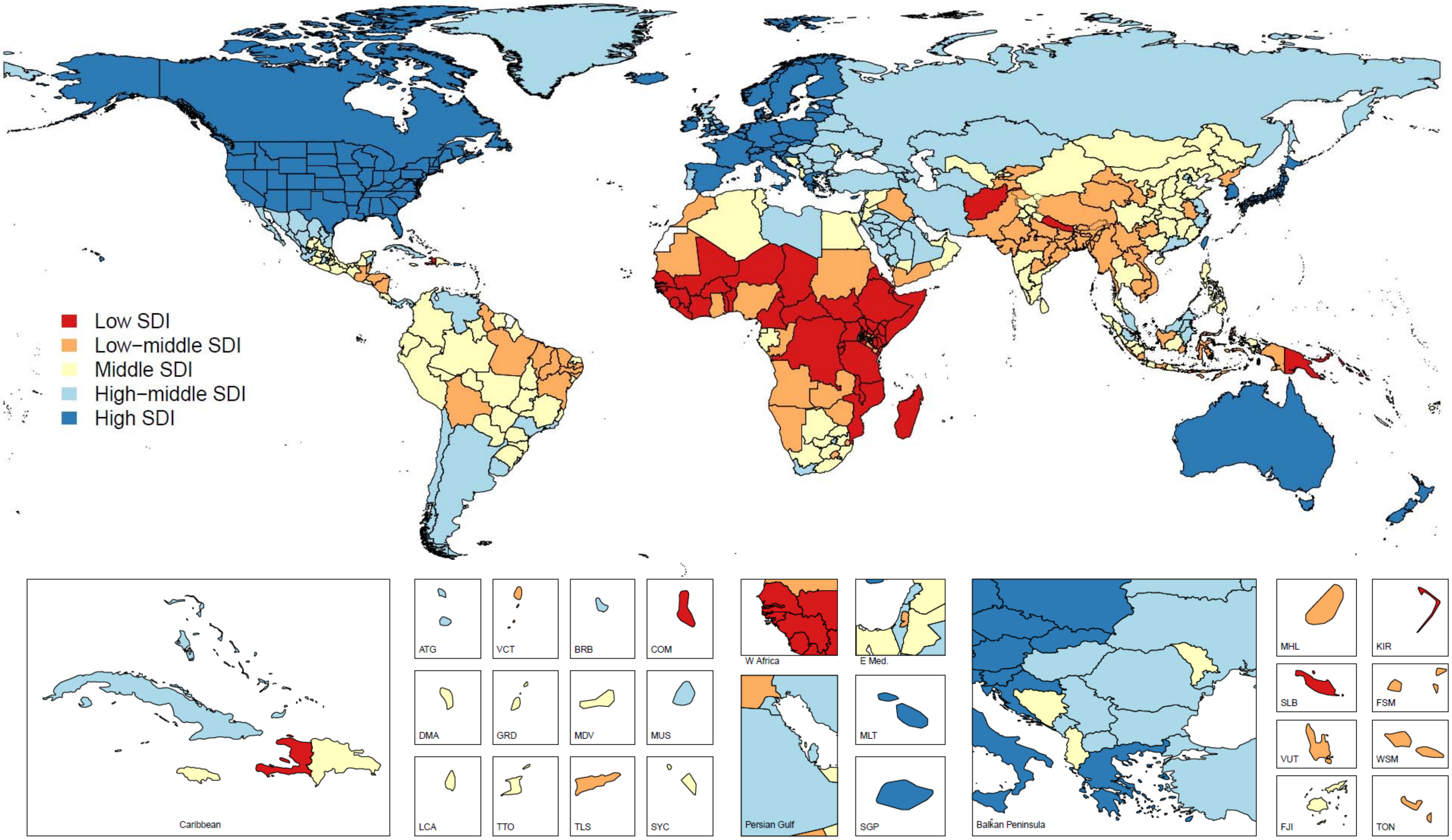

eFigure 3: Sociodemographic Index quintiles, 2016

© 2018 Fitzmaurice C et al. JAMA Oncology.

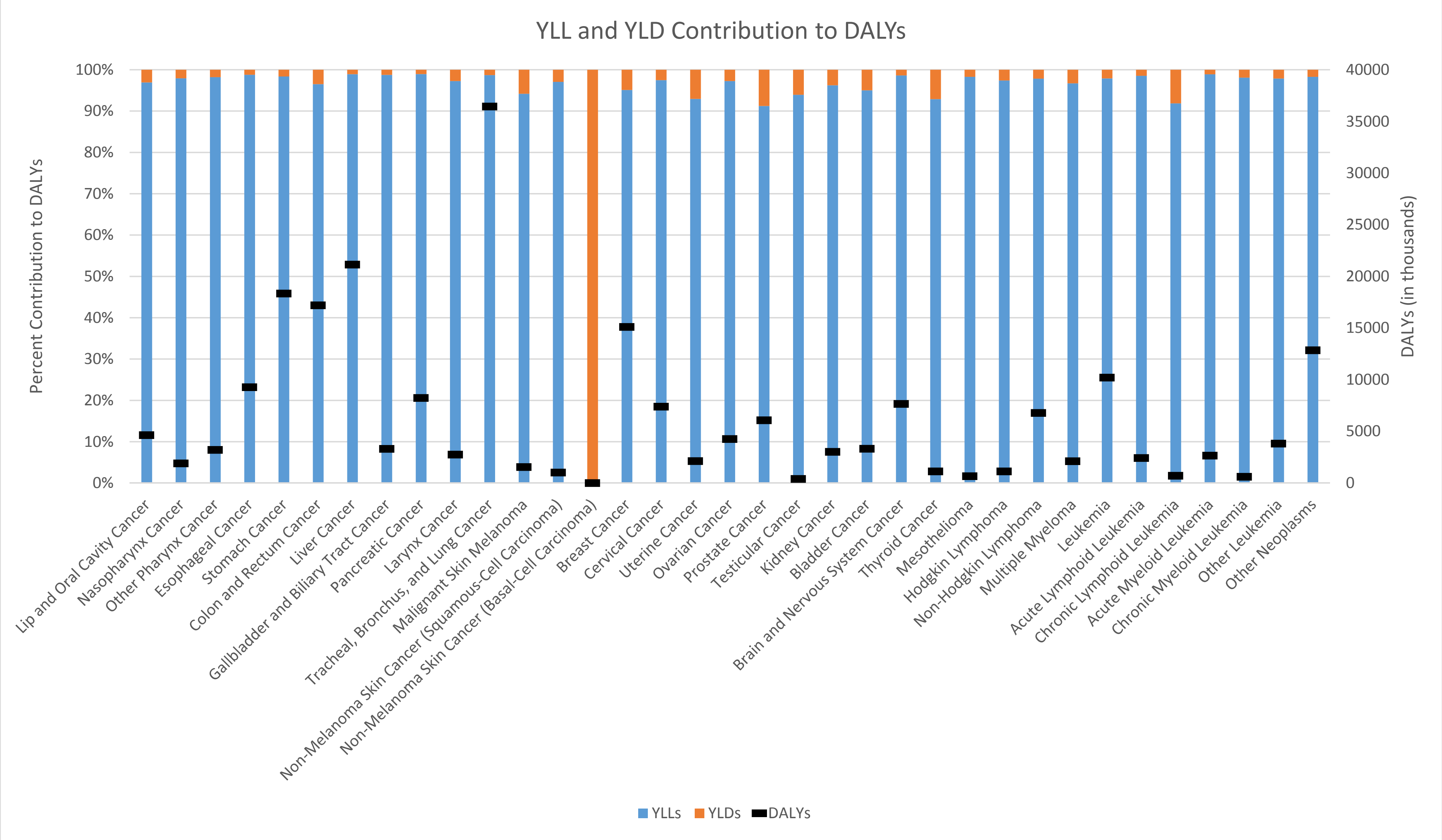

eFigure 4: Contribution of YLDs and YLLs to DALYs by cancer, global, both sexes, 2016

© 2018 Fitzmaurice C et al. JAMA Oncology.

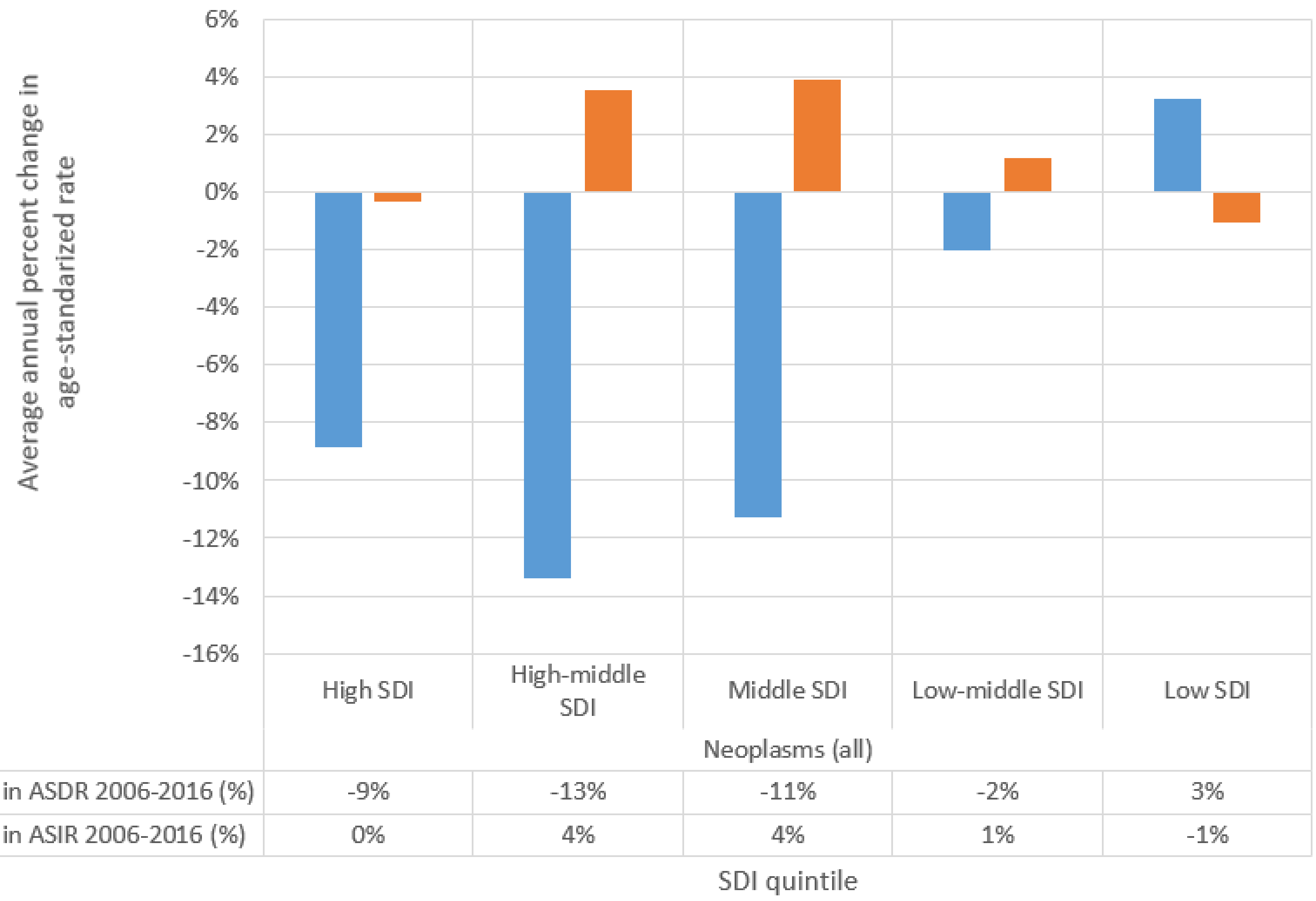

	High SDI	High-middle SDI	Middle SDI	Low-middle SDI	Low SDI
Change in ASDR 2006-2016 (%)	-9%	-13%	-11%	-2%	3%
Change in ASIR 2006-2016 (%)	0%	4%	4%	1%	-1%

eFigure 5: Average annual percent change in age-standardized death and incidence rates between 2006 and 2016 for both sexes, by SDI quintile, all neoplasms

© 2018 Fitzmaurice C et al. JAMA Oncology.

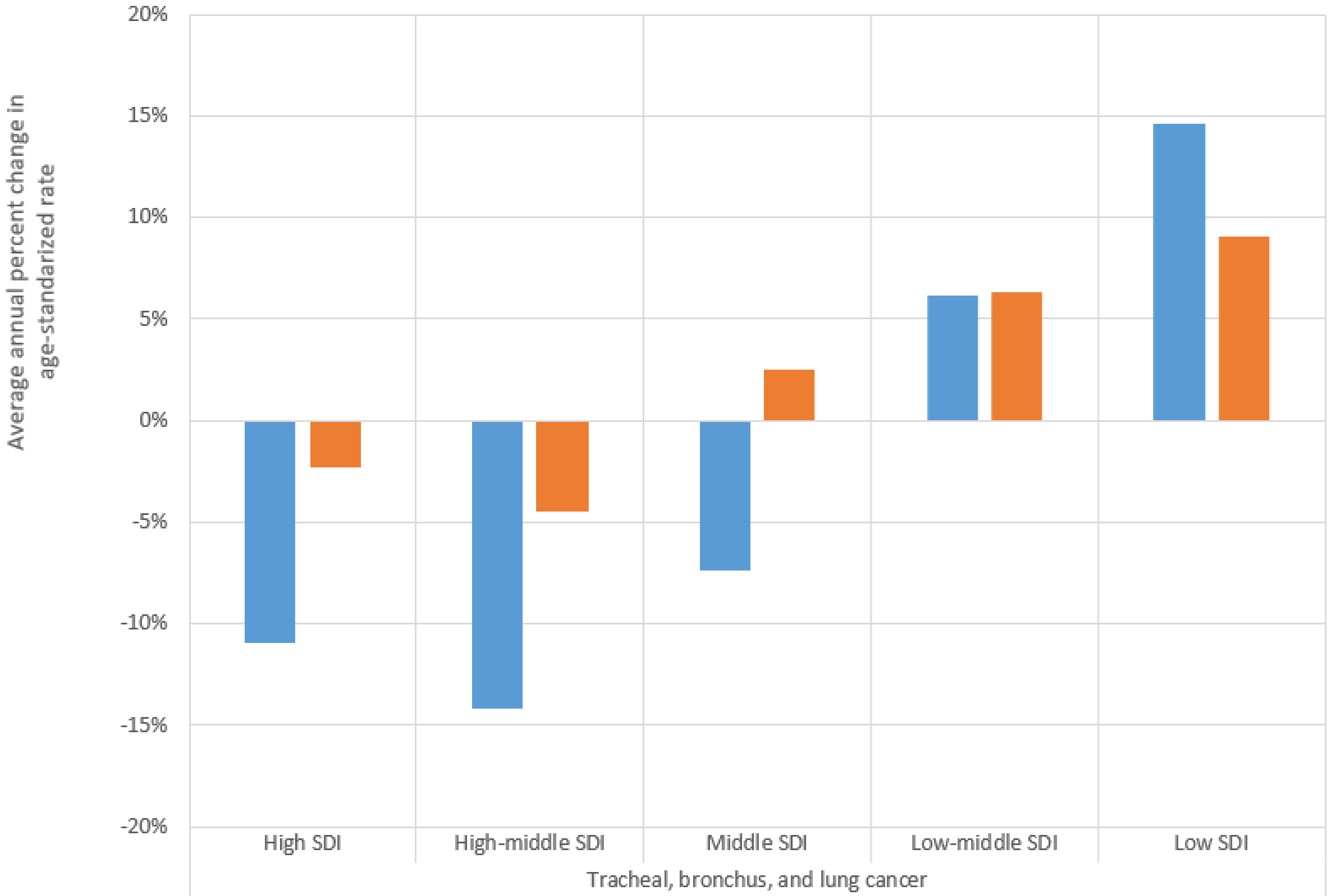

	High SDI	High-middle SDI	Middle SDI	Low-middle SDI	Low SDI
Change in ASDR 2006-2016 (%)	-11%	-14%	-7%	6%	15%
Change in ASIR 2006-2016 (%)	-2%	-5%	3%	6%	9%

SDI quintile

eFigure 6: Average annual percent change in age-standardized death and incidence rates between 2006 and 2016 for both sexes, by SDI quintile, tracheal, bronchus, and lung cancer

© 2018 Fitzmaurice C et al. JAMA Oncology.

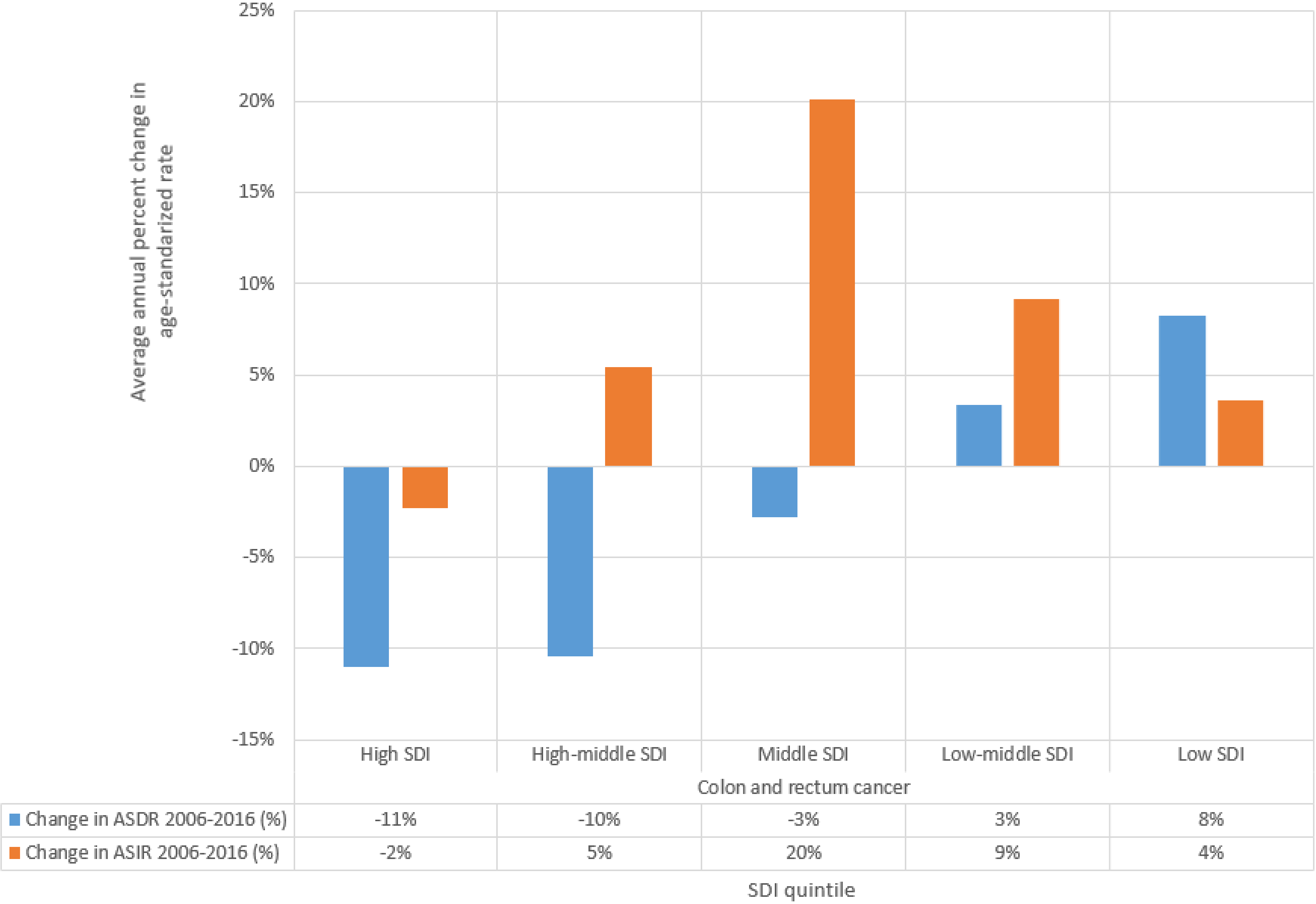

eFigure 7: Average annual percent change in age-standardized death and incidence rates between 2006 and 2016 for both sexes, by SDI quintile, colon and rectum cancer

© 2018 Fitzmaurice C et al. JAMA Oncology.

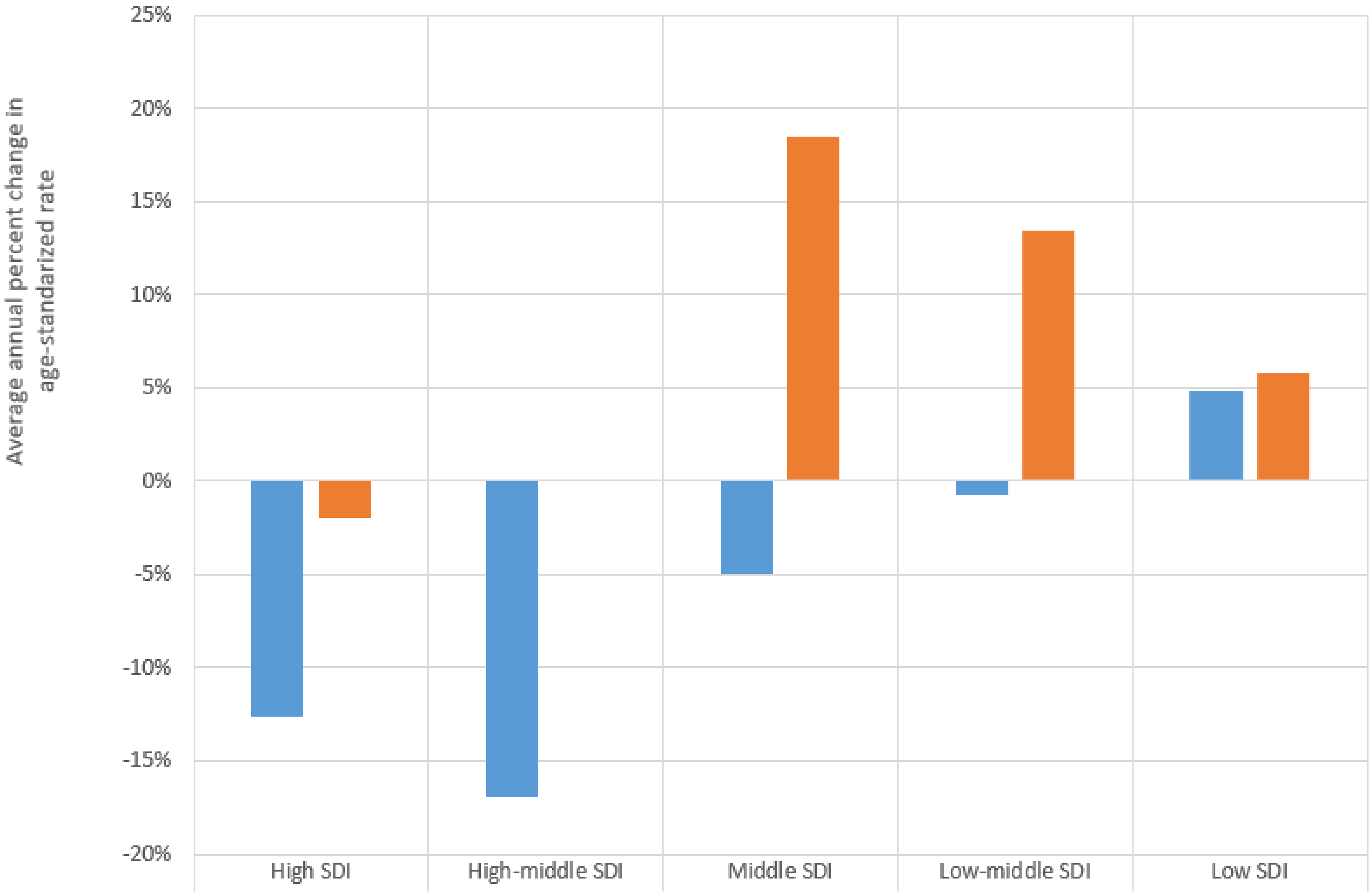

Breast cancer	High SDI	High-middle SDI	Middle SDI	Low-middle SDI	Low SDI
Change in ASDR 2006-2016 (%)	-13%	-17%	-5%	-1%	5%
Change in ASIR 2006-2016 (%)	-2%	0%	19%	13%	6%

SDI quintile

eFigure 8: Average annual percent change in age-standardized death and incidence rates between 2006 and 2016 for both sexes, by SDI quintile, breast cancer

© 2018 Fitzmaurice C et al. JAMA Oncology.

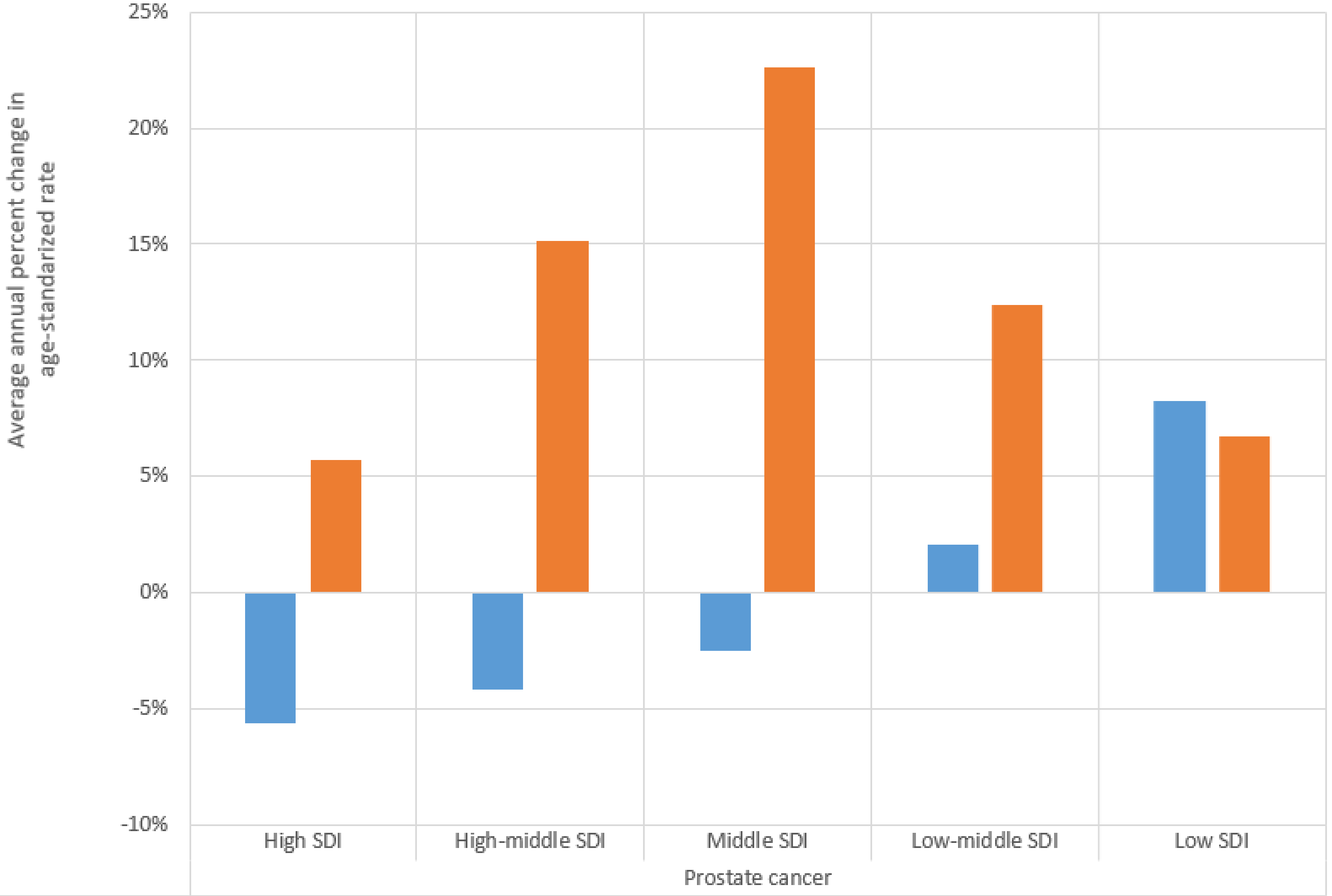

	High SDI	High-middle SDI	Middle SDI	Low-middle SDI	Low SDI
Change in ASDR 2006-2016 (%)	-6%	-4%	-3%	2%	8%
Change in ASIR 2006-2016 (%)	6%	15%	23%	12%	7%

eFigure 9: Average annual percent change in age-standardized death and incidence rates between 2006 and 2016, by SDI quintile, prostate cancer

© 2018 Fitzmaurice C et al. JAMA Oncology.

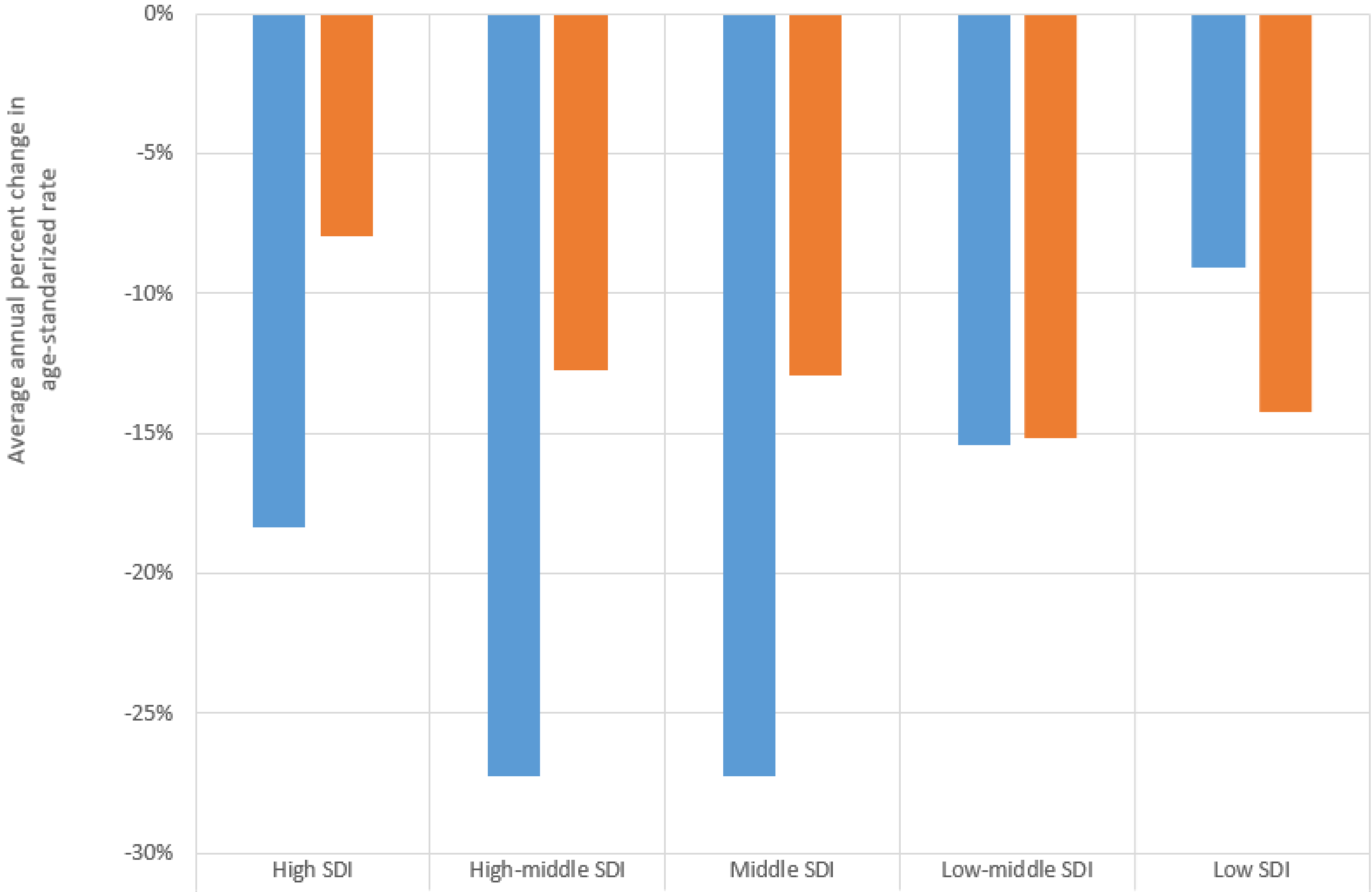

	High SDI	High-middle SDI	Middle SDI	Low-middle SDI	Low SDI
Change in ASDR 2006-2016 (%)	-18%	-27%	-27%	-15%	-9%
Change in ASIR 2006-2016 (%)	-8%	-13%	-13%	-15%	-14%

SDI quintile

eFigure 10: Average annual percent change in age-standardized death and incidence rates between 2006 and 2016 for both sexes, by SDI quintile, stomach cancer

© 2018 Fitzmaurice C et al. JAMA Oncology.

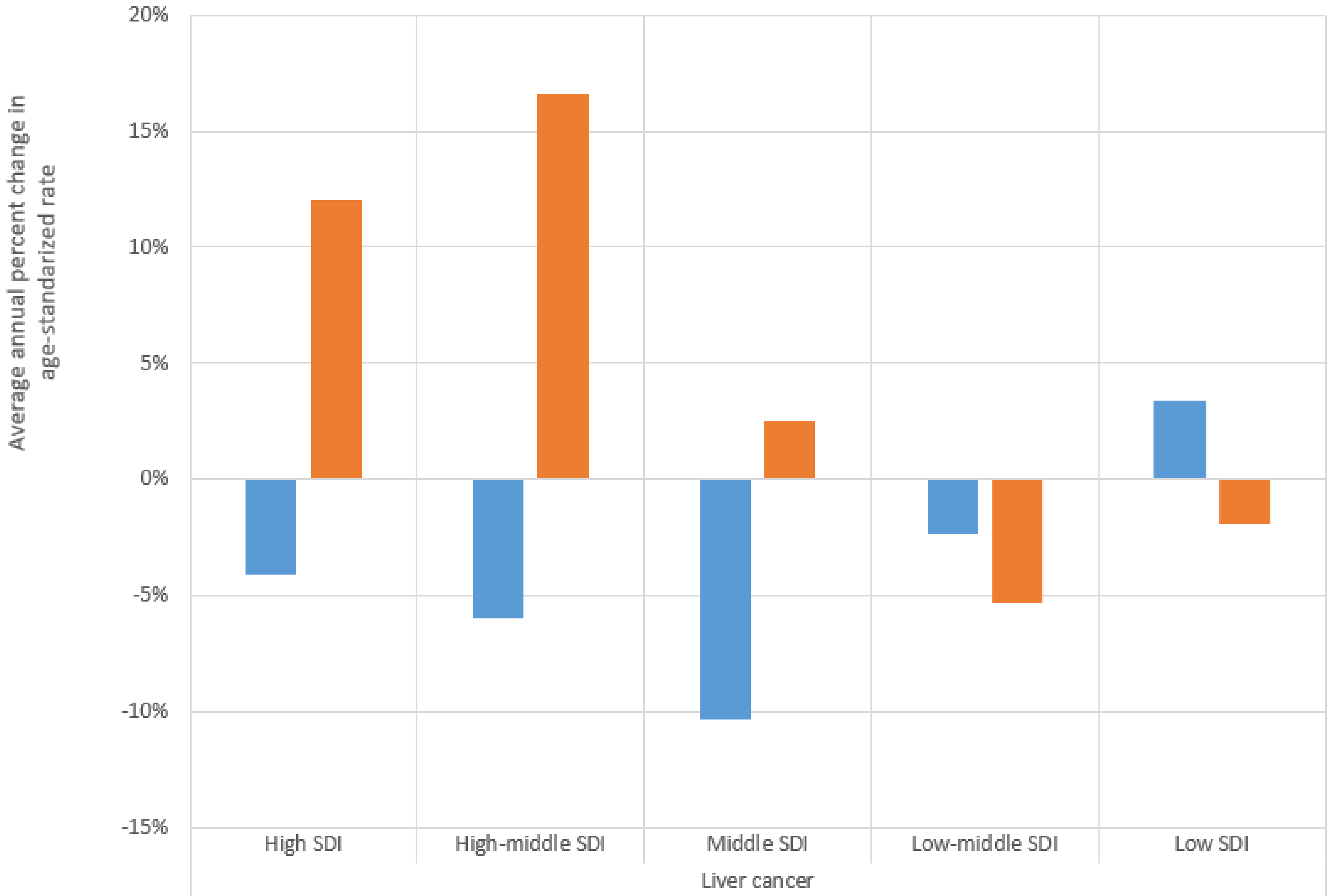

	High SDI	High-middle SDI	Middle SDI	Low-middle SDI	Low SDI
Change in ASDR 2006-2016 (%)	-4%	-6%	-10%	-2%	3%
Change in ASIR 2006-2016 (%)	12%	17%	3%	-5%	-2%

SDI quintile

eFigure 11: Average annual percent change in age-standardized death and incidence rates between 2006 and 2016 for both sexes, by SDI quintile, liver cancer

© 2018 Fitzmaurice C et al. JAMA Oncology.

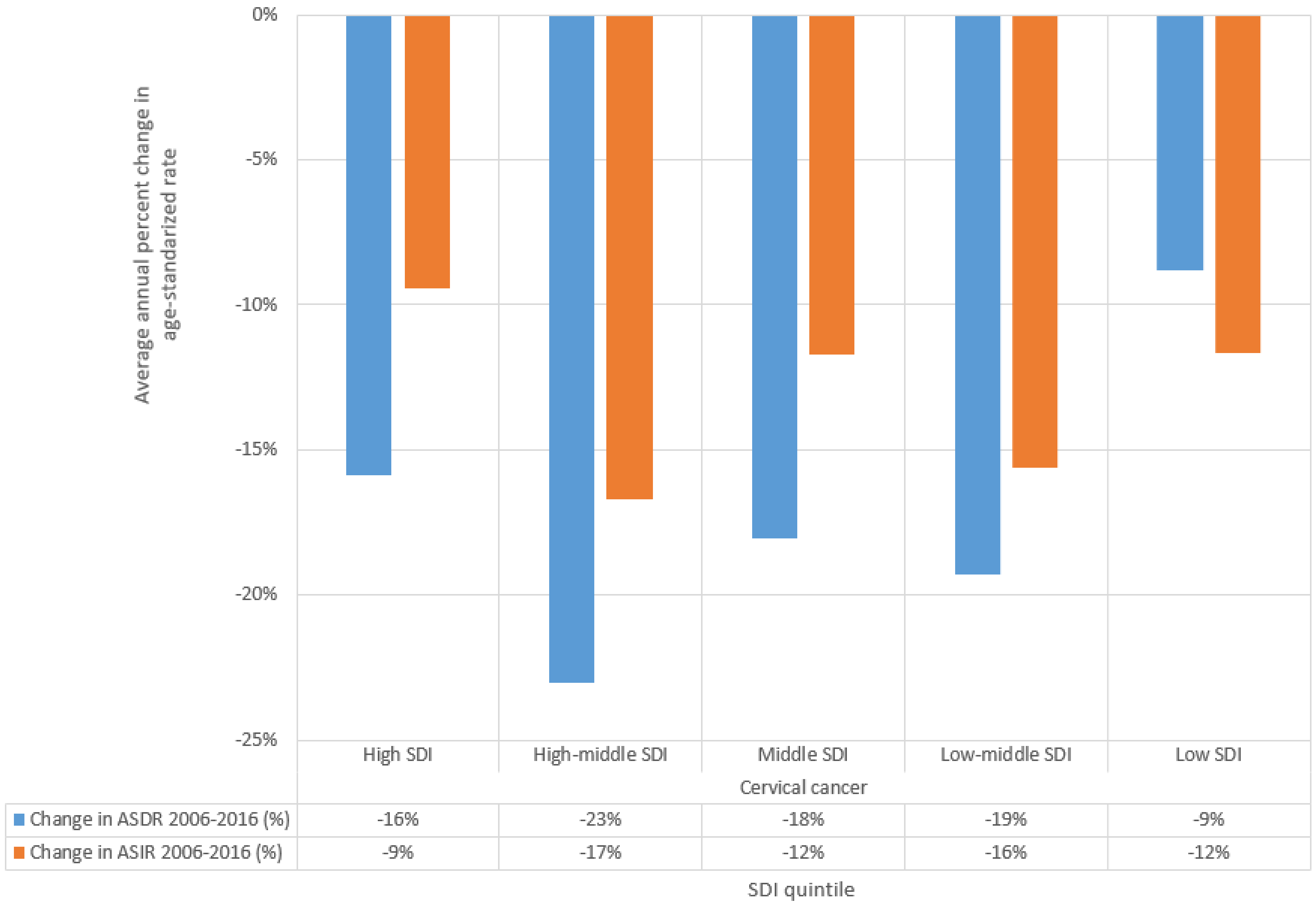

eFigure 12: Average annual percent change in age-standardized death and incidence rates between 2006 and 2016, by SDI quintile, cervical cancer

© 2018 Fitzmaurice C et al. JAMA Oncology.

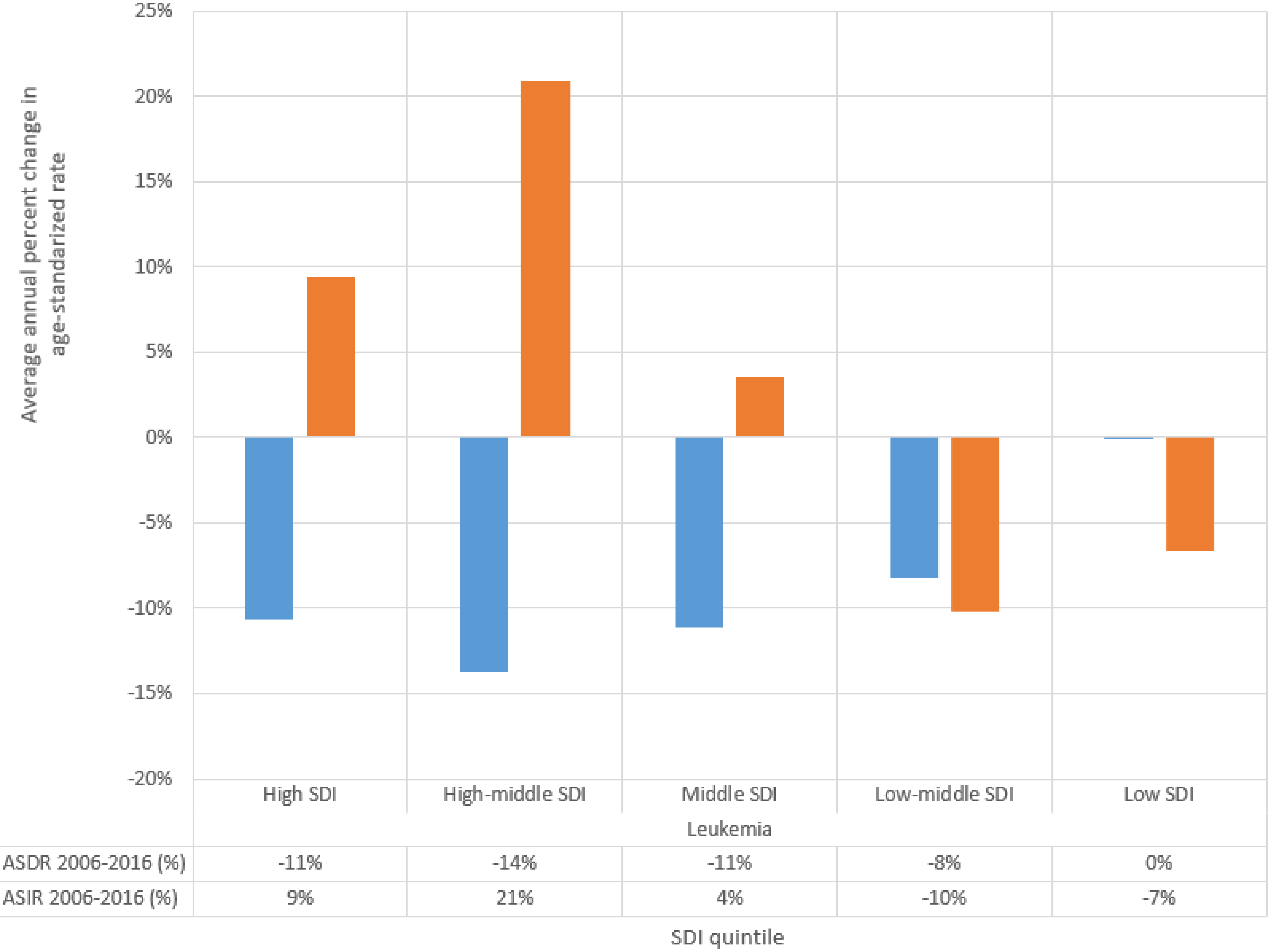

	High SDI	High-middle SDI	Middle SDI	Low-middle SDI	Low SDI
Change in ASDR 2006-2016 (%)	-11%	-14%	-11%	-8%	0%
Change in ASIR 2006-2016 (%)	9%	21%	4%	-10%	-7%

eFigure 13: Average annual percent change in age-standardized death and incidence rates between 2006 and 2016 for both sexes, by SDI quintile, leukemia

© 2018 Fitzmaurice C et al. JAMA Oncology.

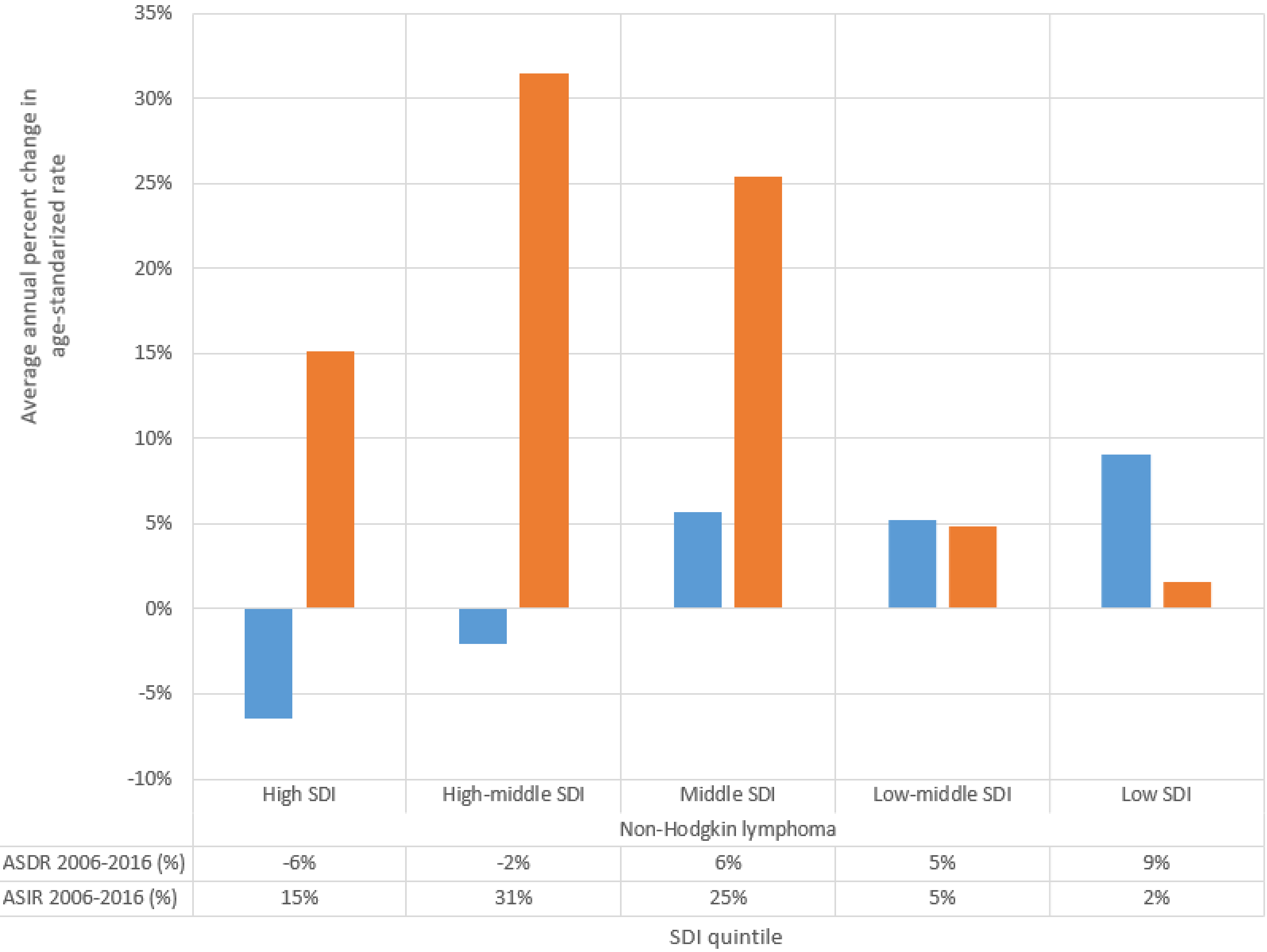

	High SDI	High-middle SDI	Middle SDI	Low-middle SDI	Low SDI
Change in ASDR 2006-2016 (%)	-6%	-2%	6%	5%	9%
Change in ASIR 2006-2016 (%)	15%	31%	25%	5%	2%

eFigure 14: Average annual percent change in age-standardized death and incidence rates between 2006 and 2016 for both sexes, by SDI quintile, non-Hodgkin lymphoma

© 2018 Fitzmaurice C et al. JAMA Oncology.

Country	Tracheal, bronchus, and lung cancer	Colon and rectum cancer	Breast cancer	Non-melanoma skin cancer	Prostate cancer	Stomach cancer	Liver cancer	Cervical cancer	Leukemia	Non-Hodgkin lymphoma	Esophageal cancer	Bladder cancer	Pancreatic cancer	Uterine cancer	Lip and oral cavity cancer	Kidney cancer	Brain and nervous system cancer	Malignant skin melanoma	Ovarian cancer	Thyroid cancer	Larynx cancer	Gallbladder and biliary tract cancer	Other pharynx cancer	Other leukemia	Multiple myeloma	Chronic lymphoid leukemia	Acute myeloid leukemia	Nasopharynx cancer	Acute lymphoid leukemia	Hodgkin lymphoma	Testicular cancer	Mesothelioma	Chronic myeloid leukemia
Global	1	2	3	4	5	6	7	9	10	11	12	13	14	15	16	17	18	19	20	21	22	23	24	25	26	27	28	29	30	31	32	33	34
High SDI	4	3	5	1	2	6	12	23	14	8	18	9	11	13	20	15	17	10	19	16	25	22	27	28	21	24	26	34	31	30	29	32	33
High-middle SDI	1	2	3	5	6	4	7	16	10	17	13	11	12	9	20	14	15	21	18	19	22	24	27	23	25	28	29	26	30	32	31	34	33
Middle SDI	1	5	4	6	8	3	2	10	11	17	9	15	14	16	13	18	12	28	19	20	22	23	25	21	27	30	29	24	26	31	32	33	34
Low-middle SDI	2	6	1	11	9	4	8	5	12	14	13	19	18	22	3	21	15	32	16	23	17	20	10	25	28	30	26	24	27	29	33	34	31
Low SDI	8	7	2	10	5	6	4	1	12	9	11	17	15	19	14	18	16	30	13	23	22	21	27	20	24	32	28	25	29	26	34	33	31
South Asia	3	7	2	21	12	5	13	6	10	15	9	18	19	22	1	23	17	33	14	20	11	16	8	29	26	30	25	24	27	28	32	34	31
India	6	8	1	19	11	5	13	4	10	16	9	21	18	22	2	23	17	32	15	20	12	14	7	29	26	30	25	24	27	28	33	34	31
Pakistan	3	4	2	31	15	12	14	18	13	8	6	7	21	17	1	22	16	33	9	19	10	20	11	28	23	32	25	27	26	24	29	34	30
Bangladesh	1	3	4	24	11	6	9	7	14	17	12	19	13	23	2	21	15	34	18	20	10	16	8	29	26	30	25	22	27	28	33	32	31
Nepal	5	7	1	28	14	2	11	3	10	17	8	19	13	23	4	21	18	33	15	20	16	12	9	27	26	30	25	22	24	29	34	32	31
Bhutan	4	5	2	26	11	7	9	6	12	14	10	20	15	25	1	21	13	32	16	19	17	18	8	29	27	30	22	23	24	28	34	33	31
East Asia	1	4	5	8	10	3	2	12	9	15	6	16	13	14	18	20	11	29	23	21	22	24	30	17	27	26	28	19	25	31	32	33	34
China	1	4	5	8	10	3	2	12	9	15	6	17	13	14	19	20	11	29	23	21	22	24	30	16	26	27	28	18	25	31	32	33	34
North Korea	1	5	7	23	16	2	3	6	8	17	4	13	10	18	19	15	11	30	20	26	22	21	31	12	28	25	27	14	24	29	34	32	33
Taiwan	3	1	4	20	7	5	2	15	12	11	9	10	16	13	8	17	21	32	22	14	26	23	18	27	28	25	29	19	24	31	30	34	33
Southeast Asia	2	3	1	12	6	7	4	5	11	13	22	17	14	18	9	16	19	30	10	15	25	23	21	24	28	34	27	20	26	29	31	33	32
Indonesia	5	3	1	8	4	7	9	2	19	11	25	18	13	12	14	17	15	31	10	16	22	21	23	24	26	34	29	20	28	27	30	32	33
Philippines	2	3	1	20	6	13	4	5	8	16	25	23	19	14	10	12	17	30	9	11	24	28	26	15	27	31	21	18	22	33	32	34	29
Vietnam	1	2	4	13	15	5	3	9	19	10	12	18	14	20	6	22	21	31	16	11	23	24	7	30	27	33	26	17	25	28	29	34	32
Thailand	2	4	3	16	7	10	1	5	9	22	18	12	15	24	6	19	17	30	14	23	20	8	26	13	27	34	31	21	25	28	32	29	33
Myanmar	1	3	4	13	6	5	7	2	9	16	22	14	12	17	10	15	18	31	11	23	21	24	26	19	28	33	27	20	25	29	34	32	30
Malaysia	3	2	1	19	5	10	7	8	6	9	23	14	17	18	15	16	21	33	12	13	25	28	26	22	27	31	24	11	20	29	30	34	32
Sri Lanka	4	3	1	19	8	9	12	11	5	14	10	17	15	20	2	7	16	33	13	21	27	24	18	22	28	34	26	25	23	29	31	30	32

© 2018 Fitzmaurice C et al. JAMA Oncology.

Country	Tracheal, bronchus, and lung cancer	Colon and rectum cancer	Breast cancer	Non-melanoma skin cancer	Prostate cancer	Stomach cancer	Liver cancer	Cervical cancer	Leukemia	Non-Hodgkin lymphoma	Esophageal cancer	Bladder cancer	Pancreatic cancer	Uterine cancer	Lip and oral cavity cancer	Kidney cancer	Brain and nervous system cancer	Malignant skin melanoma	Ovarian cancer	Thyroid cancer	Larynx cancer	Gallbladder and biliary tract cancer	Other pharynx cancer	Other leukemia	Multiple myeloma	Chronic lymphoid leukemia	Acute myeloid leukemia	Nasopharynx cancer	Acute lymphoid leukemia	Hodgkin lymphoma	Testicular cancer	Mesothelioma	Chronic myeloid leukemia
Cambodia	1	4	2	13	10	5	8	3	7	14	21	18	12	20	9	17	16	31	11	24	22	23	26	15	29	33	27	19	25	28	32	34	30
Laos	1	4	2	15	9	5	6	3	7	13	21	18	14	20	10	17	16	31	12	25	22	23	26	11	29	32	27	19	24	28	33	34	30
Mauritius	4	2	1	16	3	6	14	7	13	18	17	11	12	9	8	15	22	31	10	20	21	23	25	19	26	32	29	24	27	28	30	34	33
Timor-Leste	1	4	2	14	6	8	9	3	7	12	21	18	13	20	10	17	16	30	11	24	25	22	26	15	28	32	27	19	23	29	34	33	31
Maldives	2	4	1	12	7	16	6	5	8	14	19	9	15	23	3	17	18	29	11	13	22	25	31	20	24	32	26	27	21	33	34	28	30
Seychelles	6	3	2	20	1	13	14	4	8	9	18	7	15	23	5	16	19	30	12	27	10	28	11	21	24	33	26	22	25	29	31	34	32
North Africa and Middle East	2	3	1	9	4	5	8	18	7	12	21	6	13	19	22	16	11	28	17	15	20	24	33	14	25	29	23	30	27	26	31	34	32
Egypt	4	5	3	15	8	10	1	18	7	13	17	2	11	16	21	14	12	30	9	26	22	24	31	23	25	29	20	32	27	19	34	33	28
Iran	4	5	1	7	3	2	10	21	6	18	13	11	16	19	22	17	8	27	20	15	14	28	33	12	29	23	24	34	25	26	30	31	32
Turkey	1	3	2	4	5	6	15	21	8	12	28	7	9	14	25	13	10	19	16	18	17	27	33	26	20	24	22	31	29	32	23	30	34
Algeria	3	2	1	10	4	6	17	9	11	7	26	13	12	20	24	18	15	28	19	8	22	14	29	21	25	31	27	16	30	23	33	34	32
Iraq	2	7	1	16	13	10	11	18	3	9	25	6	12	21	20	14	5	33	15	19	17	26	32	8	29	30	23	28	24	22	31	34	27
Sudan	2	5	1	7	11	6	3	12	4	13	18	14	15	19	20	16	10	32	17	23	26	21	31	9	27	30	22	29	24	25	33	34	28
Morocco	2	3	1	10	5	9	20	4	16	7	23	11	13	17	19	21	15	29	12	8	14	24	27	22	26	34	28	18	30	25	33	31	32
Afghanistan	3	5	1	8	16	2	10	7	4	12	13	15	17	18	23	22	11	34	21	27	20	19	31	6	29	30	28	26	25	14	33	32	24
Saudi Arabia	7	2	1	12	5	15	4	20	8	6	24	11	13	17	19	14	9	30	18	3	25	23	31	16	22	33	26	27	29	21	28	34	32
Yemen	2	5	1	6	13	4	8	10	3	12	19	15	14	18	21	17	11	34	16	25	26	20	31	7	29	30	24	28	23	22	32	33	27
Syria	4	5	2	6	8	10	9	20	1	15	25	14	12	22	23	19	7	29	16	28	24	27	30	3	26	18	13	31	17	33	34	32	21
Tunisia	2	3	1	9	5	8	23	17	11	6	28	4	13	14	18	15	16	27	19	10	12	21	29	25	22	31	26	20	30	24	33	34	32
United Arab Emirates	5	2	1	4	3	14	12	16	10	6	25	11	15	20	19	13	8	26	23	9	21	30	29	18	22	33	28	27	32	24	17	34	31
Jordan	3	2	1	12	6	11	18	20	4	7	25	9	13	15	19	14	10	27	16	17	23	21	29	5	22	30	28	24	31	33	26	32	34
Libya	3	2	1	19	5	12	13	16	8	6	28	4	11	20	24	14	10	30	17	9	15	22	33	21	23	29	26	25	27	18	31	34	32
Lebanon	4	5	1	8	2	11	21	23	9	6	29	3	17	13	22	18	12	24	14	10	15	25	32	19	20	30	27	33	28	16	26	34	31
Palestine	1	3	2	9	10	8	7	18	4	13	25	14	12	16	19	15	5	31	17	27	26	23	32	6	21	30	20	29	22	24	33	34	28
Oman	3	2	1	11	4	9	8	16	7	6	19	13	14	23	15	10	12	27	20	22	24	29	34	17	18	31	21	33	26	25	28	32	30

© 2018 Fitzmaurice C et al. JAMA Oncology.

Country	Tracheal, bronchus, and lung cancer	Colon and rectum cancer	Breast cancer	Non-melanoma skin cancer	Prostate cancer	Stomach cancer	Liver cancer	Cervical cancer	Leukemia	Non-Hodgkin lymphoma	Esophageal cancer	Bladder cancer	Pancreatic cancer	Uterine cancer	Lip and oral cavity cancer	Kidney cancer	Brain and nervous system cancer	Malignant skin melanoma	Ovarian cancer	Thyroid cancer	Larynx cancer	Gallbladder and biliary tract cancer	Other pharynx cancer	Other leukemia	Multiple myeloma	Chronic lymphoid leukemia	Acute myeloid leukemia	Nasopharynx cancer	Acute lymphoid leukemia	Hodgkin lymphoma	Testicular cancer	Mesothelioma	Chronic myeloid leukemia
Kuwait	8	2	1	17	3	18	12	19	5	4	27	9	14	6	21	13	11	25	15	10	28	29	31	16	23	30	22	32	20	26	24	34	33
Qatar	4	2	1	3	5	12	7	19	6	9	22	14	13	16	21	10	11	25	17	15	24	30	33	18	26	29	20	32	27	28	23	34	31
Bahrain	3	2	1	13	4	12	16	18	7	9	24	5	11	15	20	8	17	34	14	10	23	29	28	19	21	30	25	26	27	22	32	31	33
Western Europe	5	2	3	4	1	9	15	25	12	8	20	7	11	13	19	14	16	10	17	21	24	23	26	28	18	22	27	34	33	30	29	31	32
Germany	4	2	3	5	1	7	15	25	14	8	21	11	10	13	18	12	17	9	16	22	27	19	23	30	20	24	28	33	34	29	26	31	32
United Kingdom	3	5	4	1	2	12	18	25	7	8	15	9	13	11	21	14	20	10	16	24	27	31	28	23	17	19	22	34	32	29	30	26	33
France	5	3	2	4	1	14	12	25	10	8	19	7	11	15	17	13	16	9	18	22	23	28	20	26	21	24	27	33	34	30	29	31	32
Italy	4	2	3	5	1	7	12	24	15	9	26	6	11	10	21	13	16	14	20	17	22	19	29	27	18	23	25	34	33	28	30	31	32
England	3	5	4	1	2	13	17	26	7	8	14	9	12	11	20	15	22	10	18	24	27	31	28	23	16	19	21	34	32	29	30	25	33
Spain	3	1	5	2	4	8	13	26	11	9	24	6	12	10	18	16	15	14	20	23	17	22	28	25	19	21	27	32	30	29	31	34	33
Netherlands	4	2	3	5	1	10	20	27	11	9	16	8	13	12	23	14	15	7	17	18	26	24	31	21	19	30	22	34	32	29	25	28	33
Belgium	3	4	1	5	2	11	16	26	10	8	19	7	13	12	18	14	15	9	17	21	23	28	27	24	20	22	25	34	33	29	31	30	32
Greece	3	5	2	1	4	7	12	22	8	17	29	6	9	13	25	14	10	15	16	26	18	21	32	19	23	24	27	31	33	20	28	34	30
Portugal	5	1	3	4	2	6	15	18	14	9	19	8	11	10	17	16	12	13	20	24	25	22	21	28	23	26	27	30	34	29	31	33	32
Sweden	5	3	2	4	1	14	18	20	13	9	22	8	11	10	23	15	12	6	16	24	28	21	27	29	17	19	25	34	32	30	26	31	33
South East England	5	4	3	1	2	14	19	26	7	8	13	9	12	11	22	15	21	10	17	25	27	31	29	23	16	18	20	34	32	28	30	24	33
Greater London	4	5	3	1	2	13	16	28	8	7	14	9	12	11	23	15	20	10	19	18	27	32	26	24	17	21	22	34	31	25	29	30	33
Austria	4	3	2	12	1	6	14	23	11	9	25	5	7	13	21	15	18	8	16	19	28	20	24	29	22	17	26	34	32	30	27	33	31
Switzerland	5	4	3	2	1	12	15	28	10	8	19	9	11	13	17	14	16	7	18	22	26	25	24	33	21	20	23	34	31	29	27	30	32
Israel	4	3	1	2	5	11	17	21	12	9	25	7	6	15	22	14	13	8	16	18	24	26	34	28	20	19	23	31	32	27	29	30	33
North West England	2	4	5	1	3	11	16	26	8	9	14	7	13	12	20	15	22	10	17	28	27	31	24	23	18	19	21	34	32	29	30	25	33
East of England	5	4	3	1	2	13	19	26	7	8	15	9	12	11	20	14	22	10	17	25	28	31	29	23	16	18	21	34	32	27	30	24	33
West Midlands	3	4	5	1	2	12	17	24	8	9	14	7	13	10	22	15	21	11	16	25	26	27	29	23	18	19	20	34	32	30	31	28	33
Denmark	3	2	4	7	1	15	20	24	8	10	18	5	12	13	19	14	9	6	16	23	27	28	22	26	21	17	25	34	33	30	29	31	32
Finland	5	4	2	3	1	15	17	25	14	6	22	13	9	12	21	11	10	8	16	19	30	23	26	28	18	20	24	34	32	27	29	31	33

© 2018 Fitzmaurice C et al. JAMA Oncology.

Country	Tracheal, bronchus, and lung cancer	Colon and rectum cancer	Breast cancer	Non-melanoma skin cancer	Prostate cancer	Stomach cancer	Liver cancer	Cervical cancer	Leukemia	Non-Hodgkin lymphoma	Esophageal cancer	Bladder cancer	Pancreatic cancer	Uterine cancer	Lip and oral cavity cancer	Kidney cancer	Brain and nervous system cancer	Malignant skin melanoma	Ovarian cancer	Thyroid cancer	Larynx cancer	Gallbladder and biliary tract cancer	Other pharynx cancer	Other leukemia	Multiple myeloma	Chronic lymphoid leukemia	Acute myeloid leukemia	Nasopharynx cancer	Acute lymphoid leukemia	Hodgkin lymphoma	Testicular cancer	Mesothelioma	Chronic myeloid leukemia
South West England	5	3	4	1	2	14	19	26	8	7	13	9	12	11	21	15	22	10	17	25	27	31	28	23	16	18	20	34	32	29	30	24	33
Yorkshire and the Humber	2	4	5	1	3	10	17	24	7	9	15	8	13	11	21	14	20	12	18	27	26	31	29	23	16	19	22	34	32	28	30	25	33
Scotland	1	4	2	5	3	12	19	23	7	8	13	11	14	15	16	10	18	6	17	29	26	30	24	25	21	20	22	33	32	27	28	31	34
Norway	5	2	4	3	1	15	23	19	11	10	22	8	14	12	24	13	9	6	16	21	29	26	27	28	17	18	25	34	32	30	20	33	31
East Midlands	3	4	5	1	2	13	17	24	7	9	14	8	12	11	20	15	22	10	18	27	26	30	29	23	16	19	21	34	32	28	31	25	33
Ireland	5	4	3	1	2	11	20	18	9	8	17	10	12	15	23	14	13	6	16	22	25	29	30	28	19	21	26	34	31	24	27	32	33
Wales	4	3	2	5	1	10	20	23	8	9	15	11	13	14	22	12	17	6	16	30	27	25	26	31	19	18	21	34	33	24	28	29	32
North East England	2	4	5	1	3	11	16	25	7	8	15	9	13	14	18	10	23	12	20	28	22	30	27	26	17	19	24	34	32	29	31	21	33
Northern Ireland	4	3	2	5	1	9	20	24	10	7	17	12	13	14	19	11	15	6	16	23	27	28	30	31	18	22	26	34	32	21	25	29	33
Cyprus	5	3	2	4	1	12	16	25	8	9	30	7	14	10	21	17	13	11	15	19	28	27	33	24	20	18	22	34	29	26	23	31	32
Luxembourg	4	2	1	5	3	13	16	28	9	8	23	10	14	12	21	22	11	6	17	15	24	30	25	18	20	19	26	34	31	29	27	33	32
Malta	5	3	2	4	1	11	19	24	14	9	23	6	10	8	18	12	16	13	15	17	21	29	30	33	20	26	22	31	34	25	27	28	32
Iceland	3	4	2	5	1	14	19	25	13	10	18	12	15	16	21	11	7	8	20	9	29	27	32	24	17	22	23	34	28	26	30	33	31
Andorra	5	3	2	4	1	11	19	24	10	7	18	9	13	15	22	14	12	8	16	21	28	27	30	31	20	17	23	34	32	26	29	25	33
Western SSA	9	7	2	8	4	6	1	3	11	10	14	17	13	19	18	16	12	28	15	32	25	21	27	24	22	30	20	31	26	23	34	33	29
Nigeria	8	6	1	7	3	12	2	4	11	9	14	22	15	17	20	16	10	27	13	32	23	21	29	25	24	30	19	28	26	18	34	33	31
Ghana	12	10	3	6	4	8	2	1	13	5	19	18	7	16	15	14	11	29	17	33	23	20	25	24	22	28	21	31	27	30	34	32	26
Cameroon	6	8	3	9	5	4	1	2	10	11	13	16	12	19	18	15	14	31	17	28	22	21	25	24	23	30	20	32	26	29	34	33	27
Cote d'Ivoire	7	10	2	9	1	8	4	3	11	5	23	15	12	21	14	17	16	25	13	26	20	19	24	27	18	31	22	32	29	28	34	33	30
Niger	8	11	6	7	5	3	2	1	9	10	12	16	13	22	17	15	14	32	19	30	25	21	28	20	24	29	18	31	23	26	33	34	27
Burkina Faso	10	6	4	7	8	3	2	1	13	9	11	17	12	18	20	15	14	31	16	29	24	19	25	22	23	32	21	30	27	26	33	34	28
Mali	9	8	4	7	11	3	1	2	10	12	17	6	14	22	20	13	15	27	16	26	28	18	31	21	23	30	19	32	25	24	33	34	29
Senegal	6	9	3	5	7	4	2	1	10	11	13	17	12	19	18	15	14	31	16	30	24	21	26	22	23	29	20	32	25	28	34	33	27
Chad	7	10	4	8	6	3	2	1	9	11	13	16	12	20	17	15	14	32	19	31	25	22	27	21	24	29	18	30	23	26	33	34	28
Guinea	5	9	3	7	8	4	1	2	11	14	18	12	13	20	10	16	15	22	17	27	24	19	23	30	25	31	21	33	26	28	32	34	29

© 2018 Fitzmaurice C et al. JAMA Oncology.

Country	Tracheal, bronchus, and lung cancer	Colon and rectum cancer	Breast cancer	Non-melanoma skin cancer	Prostate cancer	Stomach cancer	Liver cancer	Cervical cancer	Leukemia	Non-Hodgkin lymphoma	Esophageal cancer	Bladder cancer	Pancreatic cancer	Uterine cancer	Lip and oral cavity cancer	Kidney cancer	Brain and nervous system cancer	Malignant skin melanoma	Ovarian cancer	Thyroid cancer	Larynx cancer	Gallbladder and biliary tract cancer	Other pharynx cancer	Other leukemia	Multiple myeloma	Chronic lymphoid leukemia	Acute myeloid leukemia	Nasopharynx cancer	Acute lymphoid leukemia	Hodgkin lymphoma	Testicular cancer	Mesothelioma	Chronic myeloid leukemia
Benin	5	9	3	8	6	4	2	1	10	11	13	16	12	19	18	15	14	32	17	30	24	21	26	22	23	29	20	31	25	28	34	33	27
Togo	5	8	3	9	6	4	2	1	10	11	14	16	12	19	18	15	13	31	17	29	23	21	27	22	24	30	20	32	25	28	33	34	26
Sierra Leone	7	8	3	9	6	4	2	1	10	11	13	17	12	19	18	15	14	32	16	29	23	21	26	22	24	30	20	31	25	28	34	33	27
Liberia	9	7	3	8	5	4	1	2	10	13	12	17	11	19	18	15	14	31	16	30	24	21	26	22	23	28	20	32	25	29	34	33	27
Mauritania	7	9	3	8	4	5	1	2	12	11	16	17	10	19	18	14	13	29	15	26	24	21	27	23	22	28	20	31	25	32	34	33	30
The Gambia	6	8	3	5	12	9	1	2	11	7	17	20	10	19	16	15	14	31	13	25	28	21	24	23	18	30	22	32	26	27	34	33	29
Guinea-Bissau	6	7	4	9	5	3	2	1	10	12	11	15	13	19	17	16	14	32	18	30	22	20	25	23	24	29	21	31	27	28	34	33	26
Cape Verde	4	8	7	12	1	2	5	3	13	16	6	15	9	18	10	14	17	30	21	31	22	25	24	20	23	29	19	28	26	33	32	34	27
Sao Tome and Principe	3	6	5	7	8	4	15	2	12	11	13	9	10	18	21	14	17	30	16	28	23	19	27	26	22	29	20	31	24	33	34	32	25
Eastern Sub-Saharan Africa	11	7	4	8	3	10	6	1	13	5	9	17	15	19	14	18	16	24	12	20	25	28	26	27	22	32	30	21	29	23	34	33	31
Ethiopia	10	7	4	8	2	9	6	1	15	5	12	17	14	18	13	19	16	24	11	21	25	27	26	28	22	32	30	20	29	23	34	33	31
Tanzania	10	7	5	8	2	9	6	1	14	4	12	18	13	19	17	16	15	23	11	20	24	28	25	27	21	32	30	22	29	26	34	33	31
Kenya	12	7	5	2	6	4	8	1	11	10	9	19	13	25	15	20	16	30	14	28	17	24	26	29	21	32	23	18	22	27	33	34	31
Uganda	9	8	3	13	2	10	6	1	12	5	4	21	17	18	14	16	15	25	11	22	26	30	27	24	23	32	29	20	28	19	33	34	31
Mozambique	8	6	5	7	12	4	2	1	13	14	10	18	15	19	16	17	11	23	9	20	24	26	28	25	21	31	29	32	27	22	34	33	30
Madagascar	12	6	4	8	3	10	7	1	13	5	9	19	15	18	14	17	16	24	11	20	25	29	27	26	22	32	30	21	28	23	33	34	31
Malawi	12	9	6	11	7	13	8	2	15	4	3	5	17	21	16	10	19	18	14	20	28	24	29	23	22	32	27	31	25	26	33	34	30
Zambia	12	6	5	9	3	8	7	1	18	4	10	17	16	19	13	15	14	25	11	20	23	27	26	28	21	33	30	22	29	24	32	34	31
South Sudan	11	7	5	10	3	9	6	1	13	4	8	20	14	18	15	17	16	25	12	21	26	28	27	24	23	32	30	19	29	22	34	33	31
Rwanda	12	7	3	8	4	11	6	1	14	5	10	18	15	19	13	17	16	23	9	20	26	29	24	28	21	32	30	22	27	25	34	33	31
Burundi	11	7	4	10	3	9	6	1	13	5	8	19	16	17	14	18	15	26	12	22	25	29	24	27	23	32	30	21	28	20	34	33	31
Somalia	13	6	3	10	4	7	8	1	12	5	9	18	15	16	14	19	17	26	11	21	25	28	27	24	23	32	30	22	29	20	33	34	31
Eritrea	12	6	3	8	4	9	7	1	13	5	10	19	16	17	14	18	15	24	11	20	25	29	27	26	23	32	30	21	28	22	34	33	31
Djibouti	12	5	4	9	2	11	7	1	15	6	8	18	13	19	14	16	17	23	10	21	24	27	26	28	20	32	30	22	29	25	34	33	31
Comoros	12	6	4	8	2	11	7	1	14	5	9	18	13	19	15	17	16	23	10	20	25	29	26	28	21	32	30	22	27	24	34	33	31

© 2018 Fitzmaurice C et al. JAMA Oncology.

Country	Tracheal, bronchus, and lung cancer	Colon and rectum cancer	Breast cancer	Non-melanoma skin cancer	Prostate cancer	Stomach cancer	Liver cancer	Cervical cancer	Leukemia	Non-Hodgkin lymphoma	Esophageal cancer	Bladder cancer	Pancreatic cancer	Uterine cancer	Lip and oral cavity cancer	Kidney cancer	Brain and nervous system cancer	Malignant skin melanoma	Ovarian cancer	Thyroid cancer	Larynx cancer	Gallbladder and biliary tract cancer	Other pharynx cancer	Other leukemia	Multiple myeloma	Chronic lymphoid leukemia	Acute myeloid leukemia	Nasopharynx cancer	Acute lymphoid leukemia	Hodgkin lymphoma	Testicular cancer	Mesothelioma	Chronic myeloid leukemia
High-income North America	4	5	3	1	2	15	16	23	12	6	21	11	13	8	17	10	18	7	20	14	24	30	29	26	19	22	25	34	31	28	27	32	33
United States	4	5	3	1	2	16	15	22	12	6	21	11	13	8	17	10	18	7	20	14	24	30	28	25	19	23	27	34	31	29	26	32	33
Canada	2	4	3	6	1	11	17	23	9	5	21	8	13	12	22	14	15	10	19	18	26	25	30	27	20	16	24	34	31	28	29	32	33
Central Latin America	8	5	3	1	2	6	10	7	9	15	26	18	13	12	22	11	16	24	14	17	23	21	32	28	25	29	27	33	19	30	20	34	31
Mexico	8	5	3	1	2	6	11	7	10	15	27	19	13	12	22	9	18	23	14	17	25	21	33	29	24	30	26	34	20	28	16	32	31
Colombia	7	4	3	1	2	5	10	8	9	13	22	17	11	14	20	15	16	23	12	19	25	18	32	27	24	28	26	33	21	30	29	34	31
Venezuela	6	5	3	2	1	8	13	7	11	15	24	16	12	9	18	10	19	27	14	20	17	23	30	31	21	29	26	33	22	28	25	34	32
Guatemala	9	8	6	1	5	2	4	3	7	17	21	22	14	11	19	12	15	30	18	25	26	20	29	13	27	24	23	28	16	32	31	34	33
Honduras	9	7	6	1	2	3	10	5	4	18	29	24	12	8	16	20	17	31	14	32	25	15	23	27	26	21	19	28	11	30	34	33	22
El Salvador	7	6	4	1	3	5	9	2	8	18	23	20	12	10	19	15	13	32	14	26	25	17	27	16	24	22	29	28	21	30	33	34	31
Nicaragua	8	6	4	1	3	5	7	2	10	15	26	21	12	18	19	13	11	30	14	22	23	16	32	20	27	25	24	31	17	33	28	34	29
Costa Rica	7	3	4	2	1	5	11	8	9	13	27	15	12	10	20	14	17	19	16	21	26	23	32	33	18	28	25	30	22	29	24	34	31
Panama	8	3	2	6	1	5	12	7	10	14	26	18	13	9	19	11	15	24	16	17	22	23	30	29	20	28	25	32	21	31	27	34	33
Tropical Latin America	5	4	3	1	2	6	11	8	14	18	10	16	9	17	13	15	12	22	20	24	19	21	23	27	25	30	26	33	29	31	28	32	34
Brazil	5	4	3	1	2	6	11	8	14	18	10	16	9	17	13	15	12	21	20	24	19	22	23	27	25	30	26	33	29	31	28	32	34
Paraguay	5	4	1	9	3	7	16	2	8	15	13	19	10	11	17	12	18	26	14	20	21	22	27	23	29	30	24	33	25	31	28	34	32
Eastern Europe	3	1	2	7	4	5	18	12	13	17	22	10	11	6	16	8	19	14	15	20	21	26	23	31	25	24	28	32	29	27	30	33	34
Russia	3	1	2	8	5	6	17	12	14	18	22	10	11	4	16	7	20	13	15	19	21	27	23	31	26	24	30	34	28	25	29	33	32
Ukraine	3	1	2	4	6	5	22	9	14	19	23	7	10	11	15	12	17	16	13	21	18	24	20	27	26	25	28	30	33	29	31	32	34
Belarus	3	1	4	2	5	6	23	11	9	16	24	13	12	8	17	7	19	14	15	20	18	25	21	33	26	22	27	31	28	29	30	34	32
Moldova	4	1	3	2	6	5	7	9	19	20	23	13	8	11	15	10	16	21	18	22	14	27	17	24	25	30	29	26	31	28	32	33	34
Lithuania	3	2	4	5	1	7	19	20	10	13	23	12	11	6	22	9	17	15	14	18	21	27	25	30	24	16	26	34	29	28	31	33	32
Latvia	1	2	5	4	3	8	19	21	11	13	23	9	10	6	20	7	16	12	14	18	22	28	25	30	24	17	26	34	31	27	29	33	32
Estonia	4	2	5	3	1	6	18	17	9	10	25	11	12	7	21	8	15	13	14	20	23	27	24	30	22	19	26	34	29	28	31	33	32
High-income Asia	3	1	6	16	4	2	5	18	14	9	12	11	7	15	19	17	20	27	21	13	24	10	25	26	22	31	23	34	28	32	29	30	33

© 2018 Fitzmaurice C et al. JAMA Oncology.

Country	Tracheal, bronchus, and lung cancer	Colon and rectum cancer	Breast cancer	Non-melanoma skin cancer	Prostate cancer	Stomach cancer	Liver cancer	Cervical cancer	Leukemia	Non-Hodgkin lymphoma	Esophageal cancer	Bladder cancer	Pancreatic cancer	Uterine cancer	Lip and oral cavity cancer	Kidney cancer	Brain and nervous system cancer	Malignant skin melanoma	Ovarian cancer	Thyroid cancer	Larynx cancer	Gallbladder and biliary tract cancer	Other pharynx cancer	Other leukemia	Multiple myeloma	Chronic lymphoid leukemia	Acute myeloid leukemia	Nasopharynx cancer	Acute lymphoid leukemia	Hodgkin lymphoma	Testicular cancer	Mesothelioma	Chronic myeloid leukemia
Pacific																																	
Japan	3	1	5	14	4	2	6	20	13	9	12	11	7	15	18	16	21	27	19	17	24	10	25	26	22	31	23	34	28	32	29	30	33
South Korea	3	1	5	20	6	2	4	14	13	11	17	12	9	18	21	16	15	26	19	7	22	10	28	27	23	29	24	32	25	31	33	34	30
Singapore	3	1	2	18	4	6	5	16	11	8	20	14	10	9	21	12	17	27	15	13	24	23	29	28	25	31	22	19	26	30	32	34	33
Brunei	3	2	1	18	4	5	9	8	10	6	30	15	21	17	13	16	11	27	14	12	28	29	23	31	25	33	22	26	20	24	19	34	32
Central SSA	7	9	2	3	6	8	4	1	11	12	10	17	13	20	15	18	14	28	16	27	24	23	30	19	25	33	22	29	21	26	32	34	31
DRC	8	9	2	3	6	7	4	1	10	12	11	17	13	20	15	19	14	29	16	27	24	23	30	18	25	33	22	28	21	26	32	34	31
Angola	6	7	2	4	8	9	3	1	11	12	10	18	13	19	16	17	15	26	14	28	23	25	29	20	24	32	21	27	22	30	34	33	31
CAR	4	5	2	6	9	3	7	1	11	12	10	15	13	20	14	19	17	30	16	27	21	22	29	18	23	34	26	28	25	24	32	33	31
Congo	7	6	2	5	4	10	3	1	12	13	9	15	11	19	18	17	16	27	14	25	22	24	28	21	20	33	23	29	26	30	34	32	31
Gabon	8	5	1	6	3	11	4	2	17	12	9	13	10	19	16	14	18	23	15	22	21	25	27	26	20	32	24	29	28	30	33	34	31
Equatorial Guinea	7	6	1	5	3	16	4	2	17	10	9	14	11	19	15	13	18	21	12	20	23	27	25	26	22	33	24	29	28	30	31	34	32
Central Europe	2	1	4	3	5	8	17	14	12	13	25	6	10	7	19	11	18	15	16	22	20	23	24	30	27	21	28	33	31	29	26	34	32
Poland	1	2	3	4	5	9	23	17	10	13	24	6	11	7	19	12	16	15	14	21	20	22	25	31	26	18	28	34	30	29	27	33	32
Romania	2	1	5	4	3	6	11	7	14	18	24	8	12	10	15	13	17	21	19	22	16	25	20	30	27	23	28	32	31	29	26	34	33
Czech Republic	4	2	5	1	3	13	18	19	8	14	23	7	11	12	20	6	21	10	16	22	27	17	25	29	24	15	28	34	31	30	26	33	32
Hungary	2	1	4	3	5	10	20	18	12	15	24	6	7	9	13	8	21	14	17	25	19	23	16	29	27	22	28	32	30	31	26	34	33
Serbia	1	2	3	4	5	9	14	11	16	17	25	6	10	8	20	12	13	15	19	22	18	21	24	30	29	26	27	32	31	28	23	34	33
Bulgaria	4	1	2	3	5	7	13	10	14	11	26	8	9	6	20	16	15	19	17	21	18	24	22	30	29	25	28	33	31	27	23	34	32
Slovakia	2	1	3	4	5	7	20	15	9	12	25	10	11	8	21	6	22	13	18	26	27	17	19	31	23	16	28	32	30	29	24	34	33
Croatia	2	1	3	5	4	6	16	18	13	14	26	8	10	7	19	9	12	15	17	22	23	21	24	30	25	20	27	34	32	28	29	31	33
Bosnia and Herzegovina	1	2	4	3	5	6	8	14	16	20	22	9	7	13	21	10	11	19	15	24	18	17	25	30	26	23	27	33	31	29	28	34	32
Albania	1	6	4	2	3	5	8	14	12	19	21	22	10	13	18	11	9	23	17	20	15	24	25	16	26	33	29	30	31	27	28	32	34
Macedonia	1	3	2	4	5	6	10	14	17	19	25	8	9	7	20	18	13	12	16	21	15	22	26	30	28	23	29	33	31	27	24	34	32
Slovenia	3	1	4	5	2	7	16	21	9	6	24	14	12	11	20	13	22	8	18	25	27	19	17	29	23	15	28	34	30	31	26	33	32

© 2018 Fitzmaurice C et al. JAMA Oncology.

Country	Tracheal, bronchus, and lung cancer	Colon and rectum cancer	Breast cancer	Non-melanoma skin cancer	Prostate cancer	Stomach cancer	Liver cancer	Cervical cancer	Leukemia	Non-Hodgkin lymphoma	Esophageal cancer	Bladder cancer	Pancreatic cancer	Uterine cancer	Lip and oral cavity cancer	Kidney cancer	Brain and nervous system cancer	Malignant skin melanoma	Ovarian cancer	Thyroid cancer	Larynx cancer	Gallbladder and biliary tract cancer	Other pharynx cancer	Other leukemia	Multiple myeloma	Chronic lymphoid leukemia	Acute myeloid leukemia	Nasopharynx cancer	Acute lymphoid leukemia	Hodgkin lymphoma	Testicular cancer	Mesothelioma	Chronic myeloid leukemia
Montenegro	1	4	2	3	5	9	12	14	13	19	23	7	6	10	20	15	11	17	16	18	8	24	28	32	29	22	27	34	30	25	26	33	31
Central Asia	3	5	2	1	11	4	7	6	13	19	9	16	15	10	18	12	14	23	17	21	20	26	25	22	32	30	24	31	27	28	29	34	33
Uzbekistan	5	6	2	1	16	3	8	4	10	15	12	17	18	11	14	13	7	28	19	29	20	32	22	23	31	27	21	26	24	25	30	34	33
Kazakhstan	3	4	2	1	9	5	11	6	16	21	8	15	12	7	17	10	18	22	14	19	20	24	23	31	30	26	25	32	27	28	29	34	33
Azerbaijan	3	5	2	1	6	4	7	9	12	21	8	15	16	13	22	11	14	25	17	20	18	23	29	19	31	30	26	32	28	27	24	34	33
Tajikistan	6	9	3	1	15	2	7	11	8	18	4	17	14	20	16	12	10	25	13	29	24	23	26	19	31	27	22	30	21	28	32	34	33
Kyrgyzstan	4	7	3	1	16	2	6	5	10	19	14	18	12	15	17	9	13	26	11	22	23	25	24	21	31	28	20	29	27	32	30	34	33
Turkmenistan	5	8	2	1	14	4	10	3	12	21	6	17	20	19	13	9	11	28	15	18	24	25	22	23	29	32	27	31	30	26	16	34	33
Georgia	3	5	2	1	7	4	11	9	13	19	21	8	14	6	16	12	18	20	17	22	15	23	27	24	30	28	29	32	31	25	26	33	34
Armenia	3	4	2	1	6	5	8	11	14	18	22	7	10	12	19	16	13	26	15	21	17	23	29	20	28	25	24	34	27	31	32	30	33
Mongolia	4	8	7	3	17	2	1	6	14	18	5	20	12	11	15	10	13	31	16	22	21	19	26	30	27	25	23	29	24	32	34	33	28
Southern SSA	5	6	3	1	4	10	9	2	12	16	7	11	13	18	14	17	19	21	15	23	20	26	27	22	24	34	25	31	30	29	32	28	33
South Africa	5	6	3	1	4	14	9	2	10	18	8	12	13	17	11	16	21	20	15	23	19	26	28	22	24	34	25	30	31	29	32	27	33
Zimbabwe	8	7	3	12	2	6	4	1	15	9	5	11	14	16	19	18	17	23	13	21	22	27	31	25	20	32	24	29	26	28	33	34	30
Namibia	8	6	1	3	4	14	12	5	10	9	20	17	13	21	7	18	16	11	15	23	19	27	22	28	25	34	24	30	29	31	33	26	32
Botswana	5	8	2	4	3	11	9	1	12	18	7	14	13	17	10	16	20	21	15	23	19	26	27	22	24	34	25	28	30	31	32	29	33
Lesotho	2	8	3	4	5	9	10	1	11	21	6	14	13	19	12	18	20	24	17	28	15	23	32	16	22	34	25	27	30	29	33	26	31
Swaziland	2	8	4	3	5	10	9	1	12	20	6	16	13	21	11	14	17	23	15	26	18	25	29	19	22	33	24	27	28	31	34	30	32
Southern Latin America	4	3	1	6	2	5	17	7	15	13	16	12	10	14	22	8	21	20	19	24	23	11	31	26	25	28	27	34	30	29	18	32	33
Argentina	4	2	1	6	3	7	17	5	14	15	16	11	10	13	20	8	22	21	18	24	23	12	31	25	26	28	27	34	30	29	19	32	33
Chile	5	3	2	6	1	4	15	8	17	13	18	12	11	16	24	9	23	21	19	20	25	7	31	27	22	29	26	34	28	30	14	33	32
Uruguay	4	3	1	5	2	6	23	9	14	13	15	10	8	16	18	7	21	20	17	26	19	12	28	25	22	24	29	32	33	30	27	34	31
Andean Latin America	7	4	6	1	3	2	8	5	10	12	26	21	14	11	20	13	15	25	17	18	27	16	28	19	23	31	24	34	22	30	29	33	32
Peru	7	4	5	2	1	3	8	6	10	13	26	21	14	11	19	12	15	25	17	18	27	16	28	20	22	30	24	34	23	31	29	33	32
Ecuador	8	4	5	1	2	3	9	6	10	12	26	22	14	11	20	13	15	25	17	16	27	18	29	21	24	32	23	33	19	30	28	34	31

© 2018 Fitzmaurice C et al. JAMA Oncology.

Country	Tracheal, bronchus, and lung cancer	Colon and rectum cancer	Breast cancer	Non-melanoma skin cancer	Prostate cancer	Stomach cancer	Liver cancer	Cervical cancer	Leukemia	Non-Hodgkin lymphoma	Esophageal cancer	Bladder cancer	Pancreatic cancer	Uterine cancer	Lip and oral cavity cancer	Kidney cancer	Brain and nervous system cancer	Malignant skin melanoma	Ovarian cancer	Thyroid cancer	Larynx cancer	Gallbladder and biliary tract cancer	Other pharynx cancer	Other leukemia	Multiple myeloma	Chronic lymphoid leukemia	Acute myeloid leukemia	Nasopharynx cancer	Acute lymphoid leukemia	Hodgkin lymphoma	Testicular cancer	Mesothelioma	Chronic myeloid leukemia
Bolivia	7	5	6	2	3	1	9	4	8	12	23	20	13	11	21	15	16	25	18	17	27	14	28	19	24	32	26	34	22	30	29	33	31
Caribbean	4	2	3	6	1	9	11	8	10	13	20	12	14	7	16	17	18	25	19	23	15	26	24	22	21	29	28	31	30	27	33	34	32
Cuba	2	3	4	7	1	10	15	11	17	14	16	9	13	6	12	18	19	24	20	22	8	27	23	30	21	26	28	31	33	25	32	34	29
Haiti	5	6	2	8	3	4	11	1	7	12	19	20	14	13	15	16	18	32	17	29	22	21	27	10	23	31	26	28	24	25	34	33	30
Dominican Republic	5	3	2	4	1	9	8	7	10	16	21	18	13	12	11	17	14	29	19	24	20	27	22	15	23	30	26	25	28	31	33	34	32
Jamaica	4	3	2	11	1	7	15	5	9	10	19	13	14	8	22	17	23	25	12	21	20	24	29	16	18	27	26	28	30	32	33	34	31
Trinidad and Tobago	7	3	2	19	1	9	15	6	12	11	23	14	10	5	17	13	20	27	8	18	21	25	26	22	16	33	24	32	30	28	29	34	31
Guyana	8	4	2	6	1	10	12	3	11	15	23	18	14	7	16	13	19	26	9	20	22	24	30	17	21	29	32	31	25	27	34	33	28
Suriname	5	2	3	7	1	9	8	4	10	12	26	18	11	16	19	15	13	25	14	21	24	23	29	17	20	30	27	22	31	28	33	34	32
The Bahamas	7	3	2	9	1	8	16	6	11	10	19	21	17	5	14	15	23	22	12	20	18	24	25	26	13	27	28	30	32	29	34	33	31
Belize	4	5	3	6	2	8	9	1	10	16	21	18	12	11	17	13	15	26	19	25	20	22	28	14	23	32	30	29	24	27	34	31	33
Barbados	9	3	2	8	1	6	18	7	11	10	19	16	12	5	20	14	23	27	17	15	22	24	25	21	13	28	26	31	32	29	34	33	30
Saint Lucia	6	3	2	8	1	7	17	4	11	10	21	13	12	9	14	19	23	24	16	18	20	27	28	22	15	25	29	26	32	30	31	34	33
Saint Vincent and the Grenadines	7	3	2	6	1	9	13	4	11	10	22	16	14	8	12	19	20	24	15	23	17	26	25	18	21	27	28	29	32	30	34	33	31
Grenada	6	3	2	9	1	10	13	4	11	7	18	16	12	8	14	22	19	24	15	17	23	25	27	21	20	26	28	29	30	32	34	33	31
Antigua and Barbuda	8	3	2	5	1	6	13	7	10	11	24	16	14	9	20	18	22	21	12	15	23	25	26	17	19	33	27	28	29	30	31	34	32
Dominica	6	3	2	8	1	5	13	7	11	9	20	15	12	10	16	17	24	26	21	28	19	22	23	18	14	31	25	30	27	29	34	33	32
Australasia	6	3	5	1	2	10	16	26	8	9	22	11	12	14	18	13	21	4	23	19	30	29	31	32	17	15	20	34	24	28	27	25	33
Australia	6	3	5	1	2	10	16	26	9	7	22	11	12	14	18	13	21	4	23	19	29	30	31	33	17	15	20	34	24	28	27	25	32
New Zealand	6	3	4	1	2	11	19	25	8	9	17	14	13	12	20	10	21	5	22	23	31	26	32	28	16	15	18	34	24	29	27	30	33
Oceania	3	5	2	12	7	4	9	1	8	13	24	18	14	10	11	22	20	32	16	21	25	29	23	15	28	33	17	19	27	30	31	34	26
Papua New Guinea	3	6	2	12	9	4	8	1	5	14	24	21	15	10	11	22	19	31	17	29	23	27	20	13	28	34	18	16	26	30	32	33	25
Fiji	10	4	1	15	3	12	7	2	8	17	20	13	16	6	11	21	22	30	18	9	23	27	24	25	26	33	14	32	29	28	19	34	31
Solomon Islands	3	6	2	10	9	5	4	1	7	14	22	21	13	11	12	20	19	31	16	29	24	28	23	15	27	33	17	18	26	30	32	34	25

© 2018 Fitzmaurice C et al. JAMA Oncology.

Country	Tracheal, bronchus, and lung cancer	Colon and rectum cancer	Breast cancer	Non-melanoma skin cancer	Prostate cancer	Stomach cancer	Liver cancer	Cervical cancer	Leukemia	Non-Hodgkin lymphoma	Esophageal cancer	Bladder cancer	Pancreatic cancer	Uterine cancer	Lip and oral cavity cancer	Kidney cancer	Brain and nervous system cancer	Malignant skin melanoma	Ovarian cancer	Thyroid cancer	Larynx cancer	Gallbladder and biliary tract cancer	Other pharynx cancer	Other leukemia	Multiple myeloma	Chronic lymphoid leukemia	Acute myeloid leukemia	Nasopharynx cancer	Acute lymphoid leukemia	Hodgkin lymphoma	Testicular cancer	Mesothelioma	Chronic myeloid leukemia
Vanuatu	3	5	1	10	7	4	9	2	8	14	22	20	13	11	12	21	19	31	15	29	24	28	23	16	26	33	17	18	25	30	32	34	27
Samoa	7	3	1	6	5	4	9	2	10	14	26	18	12	11	17	19	16	20	13	15	29	27	25	22	24	33	23	21	30	32	28	34	31
Kiribati	3	8	2	10	13	6	4	7	9	11	15	25	14	23	1	12	16	31	17	32	24	28	22	18	29	33	20	21	27	30	19	34	26
Tonga	3	8	1	7	5	6	2	4	11	9	21	17	12	13	10	16	15	29	18	26	23	27	25	24	20	31	19	22	28	32	33	34	30
Federated States of Micronesia	2	4	1	11	5	6	8	3	9	14	24	16	13	10	12	19	18	30	15	25	22	29	23	20	26	33	17	21	28	32	31	34	27
Marshall Islands	3	4	1	12	9	5	6	2	8	13	22	19	14	10	11	16	18	30	17	25	24	29	23	20	26	33	15	21	27	31	32	34	28

Colors correspond to the ranking, with dark red as the most common cancer and dark green as the least common cancer for the location indicated. Rankings do not include the "other cancer" group. The numbers inside each box indicate the ranking. Abbreviations: SSA: Sub-Saharan Africa; DRC: Democratic Republic of Congo; CAR: Central African Republic

eFigure 15: Cancer ranking by total incidence based on global level for developing and developed regions and all countries, both sexes, 2016

Country	Tracheal, bronchus, and lung cancer	Stomach cancer	Colon and rectum cancer	Liver cancer	Breast cancer	Esophageal cancer	Pancreatic cancer	Prostate cancer	Leukemia	Cervical cancer	Non-Hodgkin lymphoma	Brain and nervous system cancer	Bladder cancer	Lip and oral cavity cancer	Ovarian cancer	Gallbladder and biliary tract cancer	Kidney cancer	Other pharynx cancer	Other leukemia	Larynx cancer	Multiple myeloma	Uterine cancer	Acute myeloid leukemia	Nasopharynx cancer	Malignant skin melanoma	Non-melanoma skin cancer	Acute lymphoid leukemia	Thyroid cancer	Chronic lymphoid leukemia	Mesothelioma	Hodgkin lymphoma	Chronic myeloid leukemia	Testicular cancer
Global	1	2	3	4	5	7	8	9	10	11	12	13	14	15	16	17	18	19	20	21	22	23	24	25	26	27	28	29	30	31	32	33	34
High SDI	1	5	2	7	4	12	3	6	9	21	10	15	11	20	14	16	13	25	22	26	17	23	18	30	19	28	32	29	24	27	33	31	34
High-middle SDI	1	3	2	4	5	7	6	9	10	14	13	11	12	18	15	17	16	26	20	19	22	21	23	24	25	27	28	30	29	32	31	33	34
Middle SDI	1	3	4	2	6	5	8	10	9	11	13	12	17	14	18	16	21	22	15	19	26	23	27	20	29	25	24	28	31	32	30	33	34
Low-middle SDI	1	2	5	6	3	10	13	11	12	8	14	15	19	7	18	17	27	9	20	16	24	21	22	23	31	29	25	26	32	33	28	30	34
Low SDI	7	6	8	2	4	9	12	5	11	1	10	13	15	16	14	19	25	23	18	20	21	17	28	26	29	30	27	22	32	33	24	31	34
South Asia	1	2	6	10	4	8	15	17	11	9	14	16	19	5	18	13	27	7	26	12	21	22	20	25	33	32	24	23	30	31	28	29	34

© 2018 Fitzmaurice C et al. JAMA Oncology.

Country	Tracheal, bronchus, and lung cancer	Stomach cancer	Colon and rectum cancer	Liver cancer	Breast cancer	Esophageal cancer	Pancreatic cancer	Prostate cancer	Leukemia	Cervical cancer	Non-Hodgkin lymphoma	Brain and nervous system cancer	Bladder cancer	Lip and oral cavity cancer	Ovarian cancer	Gallbladder and biliary tract cancer	Kidney cancer	Other pharynx cancer	Other leukemia	Larynx cancer	Multiple myeloma	Uterine cancer	Acute myeloid leukemia	Nasopharynx cancer	Malignant skin melanoma	Non-melanoma skin cancer	Acute lymphoid leukemia	Thyroid cancer	Chronic lymphoid leukemia	Mesothelioma	Hodgkin lymphoma	Chronic myeloid leukemia	Testicular cancer
India	3	1	7	10	4	8	14	17	11	9	15	16	19	5	18	13	27	6	26	12	21	25	20	23	33	32	24	22	30	31	28	29	34
Pakistan	2	8	5	11	1	6	19	16	9	20	7	15	14	3	13	17	27	12	26	10	21	18	24	28	31	34	22	23	32	33	25	29	30
Bangladesh	1	5	2	9	6	10	11	16	13	8	17	15	18	3	19	14	27	7	25	12	23	26	21	20	34	32	24	22	30	33	28	29	31
Nepal	2	1	5	10	4	7	13	17	11	6	15	16	19	8	18	12	27	9	26	14	25	21	23	24	33	31	22	20	30	32	28	29	34
Bhutan	2	3	4	7	6	8	12	11	10	13	14	15	20	5	18	16	25	9	26	17	22	27	19	23	32	33	21	24	31	30	28	29	34
East Asia	1	3	5	2	8	4	6	14	9	11	12	10	16	18	19	17	21	28	13	20	25	23	26	15	29	22	24	27	30	32	31	33	34
China	1	3	5	2	8	4	6	14	9	11	12	10	16	18	19	17	21	29	13	20	25	23	26	15	28	22	24	27	30	32	31	33	34
North Korea	1	2	5	3	7	4	10	17	6	9	13	12	14	21	20	16	23	31	11	18	26	19	25	15	30	24	22	27	29	32	28	33	34
Taiwan	1	4	3	2	7	5	6	10	13	14	11	19	12	8	20	18	16	17	21	24	22	23	25	15	29	27	26	28	30	31	32	33	34
Southeast Asia	1	5	3	2	4	15	9	10	8	7	11	12	20	13	14	16	23	18	17	22	27	21	28	19	30	26	24	25	34	31	29	32	33
Indonesia	2	3	4	6	1	23	9	8	13	7	10	11	14	17	12	15	19	20	22	21	25	16	29	18	30	26	27	24	34	31	28	32	33
Philippines	1	8	4	3	2	21	10	7	6	9	12	14	24	15	13	26	22	28	11	23	27	17	19	16	29	25	18	20	34	31	32	30	33
Vietnam	1	4	3	2	6	8	11	14	13	12	9	16	17	10	15	21	24	7	28	19	26	23	27	18	30	25	22	20	34	31	29	32	33
Thailand	2	7	3	1	4	13	9	14	6	8	18	15	17	12	16	5	23	24	10	20	27	25	30	19	29	22	21	26	34	28	31	32	33
Myanmar	1	4	2	6	5	19	9	12	8	3	11	13	17	14	10	18	22	24	15	20	26	16	27	21	30	28	23	25	33	32	29	31	34
Malaysia	1	6	2	4	3	14	9	11	5	12	8	16	18	19	13	23	21	26	15	22	24	27	20	10	29	28	17	25	33	32	31	30	34
Sri Lanka	1	7	5	9	3	6	10	11	2	18	12	13	23	8	20	17	14	19	15	27	25	26	22	21	31	24	16	28	34	30	29	32	33
Cambodia	1	4	3	6	2	16	9	10	8	5	11	14	17	13	15	20	24	22	12	18	27	19	28	21	31	26	23	25	34	33	29	30	32
Laos	1	3	4	5	2	16	10	11	6	7	12	13	17	14	15	18	24	23	9	22	29	19	25	20	31	28	21	26	34	32	27	30	33
Mauritius	1	4	2	8	3	12	7	6	9	10	17	18	15	11	13	19	20	24	14	21	22	16	26	23	30	27	25	28	33	32	29	31	34
Timor-Leste	1	4	2	6	3	16	10	9	8	7	11	13	17	15	14	18	23	24	12	22	28	21	26	19	30	27	20	25	34	32	29	31	33
Maldives	1	9	4	3	2	15	8	10	5	13	11	16	12	7	14	22	20	31	17	21	23	28	24	26	29	27	18	19	33	25	32	30	34
Seychelles	3	10	1	9	4	11	8	2	7	5	12	14	16	6	18	24	21	13	19	17	23	26	25	20	27	28	22	31	33	32	29	30	34
North Africa and Middle East	1	3	4	5	2	14	9	10	6	18	12	8	11	25	15	17	21	32	13	16	20	22	19	27	30	29	23	26	33	31	24	28	34
Egypt	2	9	5	1	4	14	8	12	7	23	13	10	3	26	11	18	22	28	15	20	19	24	16	33	31	29	21	27	30	32	17	25	34

© 2018 Fitzmaurice C et al. JAMA Oncology.

Country	Tracheal, bronchus, and lung cancer	Stomach cancer	Colon and rectum cancer	Liver cancer	Breast cancer	Esophageal cancer	Pancreatic cancer	Prostate cancer	Leukemia	Cervical cancer	Non-Hodgkin lymphoma	Brain and nervous system cancer	Bladder cancer	Lip and oral cavity cancer	Ovarian cancer	Gallbladder and biliary tract cancer	Kidney cancer	Other pharynx cancer	Other leukemia	Larynx cancer	Multiple myeloma	Uterine cancer	Acute myeloid leukemia	Nasopharynx cancer	Malignant skin melanoma	Non-melanoma skin cancer	Acute lymphoid leukemia	Thyroid cancer	Chronic lymphoid leukemia	Mesothelioma	Hodgkin lymphoma	Chronic myeloid leukemia	Testicular cancer
Iran	2	1	4	5	7	11	12	9	3	22	15	6	14	23	17	18	19	32	10	13	21	25	16	33	28	30	20	29	26	24	27	31	34
Turkey	1	3	2	10	6	19	4	7	5	23	11	8	12	27	14	20	18	33	17	16	15	21	13	29	24	25	22	30	28	26	32	31	34
Algeria	2	4	3	11	1	20	7	10	8	13	6	12	14	25	17	9	22	28	15	19	18	26	24	16	31	29	27	21	32	33	23	30	34
Iraq	2	10	6	11	1	21	12	13	3	24	8	5	9	23	15	25	16	29	7	14	26	20	18	28	32	30	22	19	34	33	17	27	31
Sudan	2	6	5	3	1	14	12	11	4	15	10	9	13	25	17	16	24	29	7	21	23	20	18	28	31	30	19	26	32	34	22	27	33
Morocco	1	6	3	15	2	18	8	7	10	9	5	11	13	23	14	20	25	26	16	12	21	22	27	17	30	28	29	19	34	31	24	32	33
Afghanistan	3	1	5	8	2	12	15	13	4	9	11	10	16	26	20	17	28	29	6	18	24	19	27	25	34	30	22	23	33	31	14	21	32
Saudi Arabia	3	10	2	1	4	14	7	12	5	22	8	6	13	19	16	15	17	26	11	23	18	27	20	24	31	30	25	21	32	33	29	28	34
Yemen	1	4	5	6	2	15	11	12	3	13	10	9	14	25	17	16	27	29	7	20	23	18	22	28	32	30	21	24	31	33	19	26	34
Syria	3	8	5	7	6	19	10	9	1	20	13	4	15	25	17	24	22	28	2	18	21	26	12	31	29	27	14	30	23	32	33	16	34
Tunisia	1	7	2	14	3	26	10	6	9	18	5	12	4	20	17	13	21	28	19	11	15	22	23	16	30	31	27	24	32	33	25	29	34
United Arab Emirates	1	9	2	5	3	13	10	12	6	20	8	4	14	19	22	21	16	25	11	18	15	31	17	23	28	32	26	27	34	33	24	29	30
Jordan	1	8	3	13	2	21	10	11	4	20	7	9	12	19	14	15	16	27	5	22	17	18	26	24	29	25	28	23	34	30	32	33	31
Libya	1	10	2	8	3	22	5	12	4	17	9	11	7	23	16	14	18	29	13	15	19	27	20	21	32	30	25	26	31	33	24	28	34
Lebanon	1	9	2	13	3	22	10	5	8	24	6	11	4	23	14	17	18	29	12	16	15	21	19	30	27	32	26	25	31	34	20	28	33
Palestine	1	8	2	7	3	19	11	10	4	24	12	5	13	25	15	18	16	32	6	23	17	14	20	29	30	27	22	26	31	34	21	28	33
Oman	1	8	2	4	6	13	10	11	5	20	7	9	16	18	19	21	15	31	12	23	14	26	17	33	28	29	22	30	32	27	24	25	34
Kuwait	2	10	3	5	1	17	7	12	4	21	8	9	13	22	14	19	15	26	11	24	20	16	18	27	30	28	23	25	32	29	31	33	34
Qatar	1	8	3	4	2	14	7	12	5	22	10	9	17	20	15	23	16	28	11	21	18	24	13	29	26	30	19	27	33	32	31	25	34
Bahrain	1	7	3	8	2	17	6	10	4	20	9	13	12	19	14	24	15	29	11	21	16	23	18	27	32	31	25	26	33	28	22	30	34
Western Europe	1	6	2	9	3	12	5	4	8	22	11	15	10	20	14	17	13	26	21	27	16	23	18	30	19	28	32	31	25	24	33	29	34
Germany	1	6	2	9	3	15	4	5	8	23	12	14	10	20	13	16	11	21	22	27	17	25	18	31	19	28	32	30	24	26	33	29	34
United Kingdom	1	7	2	13	4	6	5	3	8	23	11	15	10	21	12	20	14	27	25	28	16	22	17	30	19	26	32	31	24	18	33	29	34
France	1	9	2	6	3	12	5	4	7	23	11	16	10	18	14	20	13	21	17	27	15	24	19	30	22	28	32	31	26	25	33	29	34
Italy	1	4	2	6	3	18	5	7	9	25	11	14	8	19	15	13	12	28	21	24	16	26	17	31	20	27	32	30	22	23	33	29	34

© 2018 Fitzmaurice C et al. JAMA Oncology.

Country	Tracheal, bronchus, and lung cancer	Stomach cancer	Colon and rectum cancer	Liver cancer	Breast cancer	Esophageal cancer	Pancreatic cancer	Prostate cancer	Leukemia	Cervical cancer	Non-Hodgkin lymphoma	Brain and nervous system cancer	Bladder cancer	Lip and oral cavity cancer	Ovarian cancer	Gallbladder and biliary tract cancer	Kidney cancer	Other pharynx cancer	Other leukemia	Larynx cancer	Multiple myeloma	Uterine cancer	Acute myeloid leukemia	Nasopharynx cancer	Malignant skin melanoma	Non-melanoma skin cancer	Acute lymphoid leukemia	Thyroid cancer	Chronic lymphoid leukemia	Mesothelioma	Hodgkin lymphoma	Chronic myeloid leukemia	Testicular cancer
England	1	7	2	13	4	6	5	3	9	23	11	15	10	22	12	20	14	27	25	28	16	21	17	30	19	26	32	31	24	18	33	29	34
Spain	1	4	2	9	5	15	6	3	10	24	11	12	7	20	14	17	13	26	18	19	16	22	21	29	23	25	32	30	27	28	33	31	34
Netherlands	1	6	2	14	3	8	5	4	9	24	11	15	10	23	13	19	12	25	18	26	16	22	21	28	17	27	32	29	30	20	33	31	34
Belgium	1	7	2	12	3	11	5	4	8	22	10	15	9	19	14	21	13	25	17	26	16	23	18	29	20	28	32	31	27	24	33	30	34
Greece	1	6	2	7	3	21	5	4	8	20	17	10	9	26	13	15	14	33	12	19	16	22	18	28	23	24	32	30	25	31	27	29	34
Portugal	2	3	1	8	5	13	6	4	9	21	11	12	10	18	15	17	16	23	24	19	14	22	20	28	25	27	31	29	26	32	33	30	34
Sweden	1	7	2	13	5	18	4	3	8	20	10	14	9	23	11	16	12	26	24	30	17	21	19	32	15	27	31	29	22	25	33	28	34
South East England	1	9	2	15	4	6	5	3	8	24	11	14	10	22	12	20	13	27	25	29	17	21	16	30	19	26	33	31	23	18	32	28	34
Greater London	1	6	2	12	3	7	5	4	8	23	11	13	10	21	14	20	15	27	25	28	17	22	16	30	18	26	32	31	24	19	33	29	34
Austria	1	6	2	7	4	17	3	5	9	22	10	14	11	20	12	15	13	24	25	27	16	23	19	31	18	26	32	30	21	28	33	29	34
Switzerland	1	9	2	7	4	12	5	3	11	26	8	14	10	18	13	19	15	24	28	27	16	22	20	31	17	25	32	29	23	21	33	30	34
Israel	1	6	2	12	3	19	4	8	5	20	7	10	11	25	13	22	14	33	24	26	16	21	15	32	17	23	29	27	18	28	31	30	34
North West England	1	7	2	12	3	6	5	4	9	22	11	15	10	21	13	20	14	25	26	28	18	23	16	30	19	27	32	31	24	17	33	29	34
East of England	1	7	2	14	4	6	5	3	9	23	11	15	10	24	12	20	13	27	25	29	16	21	18	30	19	26	33	31	22	17	32	28	34
West Midlands	1	7	2	13	4	6	5	3	9	23	11	15	10	22	12	20	14	27	25	28	16	21	17	30	18	26	33	31	24	19	32	29	34
Denmark	1	9	2	15	4	12	5	3	6	21	13	10	7	20	11	19	14	22	26	27	17	24	18	30	16	28	31	33	23	25	32	29	34
Finland	1	6	2	9	5	16	3	4	10	24	8	13	14	21	12	17	11	26	25	29	15	19	20	33	18	27	31	28	22	23	32	30	34
South West England	1	7	2	14	4	6	5	3	9	24	11	15	10	23	12	21	13	27	26	29	16	20	17	30	18	25	32	31	22	19	33	28	34
Yorkshire and the Humber	1	6	2	12	4	7	5	3	9	21	11	15	10	23	14	20	13	27	25	28	16	22	17	30	19	26	32	31	24	18	33	29	34
Scotland	1	7	2	12	3	5	6	4	8	25	11	15	9	18	14	21	13	26	23	27	17	22	16	29	19	28	32	31	24	20	30	33	34
Norway	1	7	2	17	5	16	4	3	8	20	12	11	9	22	10	19	14	27	25	29	15	21	18	32	13	26	31	30	23	24	33	28	34
East Midlands	1	7	2	13	4	6	5	3	9	22	11	15	10	24	12	20	14	27	25	28	17	21	16	30	18	26	33	31	23	19	32	29	34
Ireland	1	6	2	14	3	7	5	4	8	21	10	12	15	22	11	19	13	27	20	26	16	24	18	30	17	25	31	32	23	28	33	29	34
Wales	1	6	2	14	4	7	5	3	8	23	11	15	10	22	12	20	13	27	26	28	17	21	16	30	18	25	31	33	24	19	32	29	34
North East England	1	6	2	11	4	7	5	3	9	22	12	16	10	21	14	20	13	28	25	26	17	24	18	30	19	27	32	31	23	15	33	29	34

© 2018 Fitzmaurice C et.al. JAMA Oncology.

Country	Tracheal, bronchus, and lung cancer	Stomach cancer	Colon and rectum cancer	Liver cancer	Breast cancer	Esophageal cancer	Pancreatic cancer	Prostate cancer	Leukemia	Cervical cancer	Non-Hodgkin lymphoma	Brain and nervous system cancer	Bladder cancer	Lip and oral cavity cancer	Ovarian cancer	Gallbladder and biliary tract cancer	Kidney cancer	Other pharynx cancer	Other leukemia	Larynx cancer	Multiple myeloma	Uterine cancer	Acute myeloid leukemia	Nasopharynx cancer	Malignant skin melanoma	Non-melanoma skin cancer	Acute lymphoid leukemia	Thyroid cancer	Chronic lymphoid leukemia	Mesothelioma	Hodgkin lymphoma	Chronic myeloid leukemia	Testicular cancer
Northern Ireland	1	6	2	14	3	7	5	4	12	22	8	15	13	20	9	21	10	27	28	26	16	23	19	29	17	25	33	32	24	18	31	30	34
Cyprus	1	6	2	11	3	21	5	4	7	23	9	12	10	25	13	15	16	33	18	27	14	19	17	32	20	24	28	29	22	26	30	31	34
Luxembourg	1	7	2	9	3	14	4	5	6	23	13	12	11	20	10	21	19	24	15	25	16	22	18	30	17	28	32	29	27	26	33	31	34
Malta	1	5	2	14	3	16	4	6	8	24	10	12	9	19	11	22	13	29	28	23	17	18	15	25	20	26	32	30	27	21	33	31	34
Iceland	1	7	2	14	5	11	4	3	10	23	13	8	12	21	16	20	6	29	19	27	15	24	18	32	17	30	31	22	26	25	33	28	34
Andorra	1	6	2	14	4	12	5	3	8	23	9	13	10	21	11	20	15	27	24	28	16	25	17	29	19	26	32	31	22	18	33	30	34
Western SSA	8	5	7	1	3	12	9	2	11	4	10	13	14	21	15	16	22	25	20	23	19	17	18	32	27	31	26	29	30	34	24	28	33
Nigeria	7	9	6	1	3	13	12	2	8	4	10	11	22	24	14	15	23	27	19	21	20	16	18	30	26	31	25	32	28	33	17	29	34
Ghana	9	6	8	1	3	13	5	4	12	2	7	11	15	18	16	17	21	24	22	23	19	14	20	32	28	27	25	33	30	31	29	26	34
Cameroon	3	2	7	1	6	10	9	5	11	4	12	13	14	20	17	15	19	24	23	21	22	16	18	32	27	29	25	30	31	33	28	26	34
Cote d'Ivoire	5	6	9	2	3	21	11	1	10	4	7	12	13	16	14	17	25	23	22	18	15	19	20	32	24	31	28	26	30	34	27	29	33
Niger	5	2	8	1	7	10	11	4	9	3	12	13	14	20	19	15	22	25	17	21	23	16	18	32	31	30	24	28	29	34	26	27	33
Burkina Faso	8	3	5	1	4	9	10	7	12	2	11	13	15	18	17	16	21	24	20	23	19	14	22	31	29	30	26	28	32	34	25	27	33
Mali	8	2	7	1	4	15	10	9	11	3	12	13	5	22	16	14	17	29	18	24	20	21	19	33	26	31	25	27	30	34	23	28	32
Senegal	4	3	8	1	5	11	9	6	10	2	12	13	14	20	15	17	22	25	19	23	21	16	18	32	28	31	24	29	30	34	27	26	33
Chad	4	3	8	1	7	12	10	5	9	2	11	13	14	20	19	15	21	25	16	23	22	17	18	32	29	31	24	28	30	34	26	27	33
Guinea	4	3	8	1	5	17	9	7	10	2	13	12	11	14	16	15	20	24	26	23	22	18	21	33	19	32	25	29	31	34	27	28	30
Benin	4	2	7	1	5	10	9	6	11	3	12	13	14	19	17	15	21	24	20	23	22	16	18	32	28	31	25	29	30	34	27	26	33
Togo	5	2	7	1	4	11	9	6	10	3	12	13	14	19	15	17	23	25	20	22	21	16	18	33	29	30	24	28	31	34	27	26	32
Sierra Leone	5	2	7	1	4	11	9	6	10	3	12	13	14	20	15	16	23	24	19	21	22	17	18	32	29	31	25	28	30	34	27	26	33
Liberia	8	2	6	1	4	10	9	5	11	3	12	13	14	20	15	17	21	24	18	23	22	16	19	32	30	31	25	29	28	33	27	26	34
Mauritania	6	4	8	1	2	12	9	5	11	3	10	13	15	21	14	17	19	25	22	23	20	16	18	32	26	31	24	27	29	33	30	28	34
The Gambia	5	6	8	1	4	13	7	11	10	2	9	12	18	20	14	16	22	23	21	26	15	17	19	32	29	30	24	25	31	34	27	28	33
Guinea-Bissau	5	2	6	1	4	9	10	7	11	3	12	13	14	20	17	16	23	24	18	19	22	15	21	32	30	29	25	28	31	34	27	26	33
Cape Verde	3	1	6	5	10	4	7	2	11	8	13	14	15	12	24	23	16	22	18	20	21	17	19	28	27	30	25	31	29	34	32	26	33

© 2018 Fitzmaurice C et al. JAMA Oncology.

Country	Tracheal, bronchus, and lung cancer	Stomach cancer	Colon and rectum cancer	Liver cancer	Breast cancer	Esophageal cancer	Pancreatic cancer	Prostate cancer	Leukemia	Cervical cancer	Non-Hodgkin lymphoma	Brain and nervous system cancer	Bladder cancer	Lip and oral cavity cancer	Ovarian cancer	Gallbladder and biliary tract cancer	Kidney cancer	Other pharynx cancer	Other leukemia	Larynx cancer	Multiple myeloma	Uterine cancer	Acute myeloid leukemia	Nasopharynx cancer	Malignant skin melanoma	Non-melanoma skin cancer	Acute lymphoid leukemia	Thyroid cancer	Chronic lymphoid leukemia	Mesothelioma	Hodgkin lymphoma	Chronic myeloid leukemia	Testicular cancer
Sao Tome and Principe	2	3	5	13	6	10	8	7	12	4	9	14	11	24	17	16	19	25	23	21	20	15	18	32	30	27	22	28	29	33	31	26	34
Eastern SSA	10	9	5	4	7	8	11	3	13	1	6	14	15	16	12	26	27	24	25	20	18	17	30	23	21	29	28	19	33	32	22	31	34
Ethiopia	9	8	4	5	6	10	12	2	13	1	7	14	17	15	11	25	28	24	26	22	18	16	30	20	21	27	29	19	33	32	23	31	34
Tanzania	9	7	5	6	8	10	11	1	13	3	4	14	15	18	12	27	24	23	26	21	17	16	30	22	19	28	29	20	33	32	25	31	34
Kenya	9	2	4	5	6	7	10	8	11	3	12	13	19	16	14	21	28	24	23	15	17	27	22	18	29	25	20	31	33	34	26	30	32
Uganda	9	10	7	6	4	3	13	1	11	2	5	14	17	16	12	29	27	25	20	24	19	15	30	22	23	28	26	21	33	34	18	31	32
Mozambique	7	3	5	1	6	8	12	10	14	2	13	9	15	17	11	25	24	28	23	22	18	16	29	33	21	26	27	19	31	32	20	30	34
Madagascar	10	9	4	7	5	8	12	3	11	1	6	14	16	17	13	26	29	25	23	24	18	15	30	21	22	27	28	19	34	33	20	31	32
Malawi	10	11	9	7	8	2	12	6	13	3	4	15	5	17	16	24	18	29	20	26	21	22	27	32	14	23	25	19	33	34	28	30	31
Zambia	10	7	5	6	8	9	11	3	14	1	4	13	16	15	12	27	25	23	28	19	17	18	30	24	21	26	29	20	34	33	22	31	32
South Sudan	10	9	5	6	8	4	12	2	11	1	7	15	16	17	13	26	27	25	21	24	20	14	30	18	23	28	29	19	32	34	22	31	33
Rwanda	10	9	7	6	4	8	12	3	13	1	5	14	15	17	11	28	26	22	25	23	18	16	30	21	19	29	27	20	33	32	24	31	34
Burundi	10	9	8	7	4	6	13	3	11	1	5	14	16	17	12	26	29	22	25	21	19	15	30	23	24	28	27	20	34	33	18	31	32
Somalia	10	6	5	9	4	7	13	3	11	1	8	15	16	19	12	25	29	26	21	23	20	14	30	22	24	28	27	17	33	34	18	31	32
Eritrea	10	8	6	7	4	9	13	3	12	1	5	14	16	18	11	26	28	25	24	23	17	15	30	22	21	29	27	19	34	33	20	31	32
Djibouti	10	9	4	6	7	8	11	1	13	2	5	14	15	16	12	27	23	24	26	22	17	18	30	21	20	28	29	19	32	33	25	31	34
Comoros	10	9	5	7	4	8	12	3	13	2	6	14	15	18	11	28	25	26	24	23	17	16	30	21	20	29	27	19	33	32	22	31	34
High-income North America	1	10	2	9	3	12	4	5	6	21	8	13	11	22	14	24	15	28	19	25	16	20	17	33	18	26	30	29	23	27	32	31	34
United States	1	12	2	9	3	11	4	5	6	21	8	13	10	22	14	25	15	27	19	24	16	20	17	33	18	26	30	29	23	28	32	31	34
Canada	1	6	2	12	3	11	5	4	8	21	7	14	10	20	15	17	13	28	22	27	16	23	19	31	18	26	30	29	24	25	33	32	34
Central Latin America	1	2	4	6	5	17	10	3	8	9	11	12	18	25	13	14	16	30	21	23	19	24	20	33	26	22	15	27	31	34	28	29	32
Mexico	1	2	4	6	5	18	10	3	8	9	11	12	17	24	14	16	13	33	22	23	19	25	20	34	27	21	15	26	32	31	28	30	29
Colombia	2	1	3	8	5	15	10	4	7	9	11	12	18	24	14	13	19	31	20	22	17	25	23	32	26	21	16	27	29	33	30	28	34
Venezuela	1	3	5	10	4	16	9	2	8	7	11	12	18	24	13	17	14	27	25	15	20	23	21	31	28	22	19	29	32	34	26	30	33
Guatemala	6	1	7	2	9	17	10	3	5	4	14	12	22	21	19	15	20	26	11	25	24	16	23	28	30	18	13	27	31	34	29	32	33

© 2018 Fitzmaurice C et.al. JAMA Oncology.

Country	Tracheal, bronchus, and lung cancer	Stomach cancer	Colon and rectum cancer	Liver cancer	Breast cancer	Esophageal cancer	Pancreatic cancer	Prostate cancer	Leukemia	Cervical cancer	Non-Hodgkin lymphoma	Brain and nervous system cancer	Bladder cancer	Lip and oral cavity cancer	Ovarian cancer	Gallbladder and biliary tract cancer	Kidney cancer	Other pharynx cancer	Other leukemia	Larynx cancer	Multiple myeloma	Uterine cancer	Acute myeloid leukemia	Nasopharynx cancer	Malignant skin melanoma	Non-melanoma skin cancer	Acute lymphoid leukemia	Thyroid cancer	Chronic lymphoid leukemia	Mesothelioma	Hodgkin lymphoma	Chronic myeloid leukemia	Testicular cancer
Honduras	3	1	6	5	8	26	9	4	2	7	15	14	25	20	16	12	27	19	21	22	24	11	17	30	31	28	10	32	23	33	29	18	34
El Salvador	3	1	4	7	8	18	9	5	6	2	14	11	19	22	16	13	20	27	12	23	21	15	26	29	32	24	17	31	25	33	28	30	34
Nicaragua	6	1	5	3	7	20	10	4	8	2	14	11	21	23	15	12	17	28	16	19	25	24	22	32	29	18	13	26	30	34	31	27	33
Costa Rica	4	1	2	6	5	20	9	3	8	11	10	12	14	24	15	18	17	30	26	23	13	21	19	31	25	22	16	32	29	34	28	27	33
Panama	2	4	3	8	5	19	10	1	6	9	12	11	21	23	14	18	15	25	22	24	16	17	20	30	27	26	13	28	29	34	32	31	33
Tropical Latin America	1	3	2	8	5	9	7	4	12	10	13	11	17	14	18	15	19	20	23	16	21	25	22	33	26	24	27	28	30	29	31	32	34
Brazil	1	2	3	8	5	9	7	4	12	11	13	10	17	14	18	15	19	20	24	16	21	25	22	33	26	23	27	28	30	29	31	32	34
Paraguay	1	6	2	11	4	10	9	3	8	5	12	13	23	17	15	14	18	25	16	20	26	19	21	34	28	24	22	27	30	33	29	31	32
Eastern Europe	1	3	2	8	4	14	5	6	10	15	19	13	12	16	9	22	11	21	25	18	23	17	24	31	20	26	28	29	27	32	30	33	34
Russia	1	3	2	8	4	12	5	7	11	15	19	14	13	17	10	22	9	21	27	18	23	16	24	33	20	25	28	29	26	32	30	31	34
Ukraine	1	3	2	13	4	15	5	6	8	10	21	12	11	14	9	18	16	19	23	17	25	22	24	28	20	27	29	31	26	30	32	33	34
Belarus	1	3	2	13	4	12	5	6	7	10	17	11	14	16	9	19	15	20	30	18	23	25	22	29	21	26	27	31	24	33	32	28	34
Moldova	1	3	2	5	4	20	6	8	12	9	17	10	14	15	16	23	18	13	22	11	25	19	26	27	21	24	28	30	31	33	29	34	32
Lithuania	1	3	2	14	5	13	6	4	10	15	16	12	11	17	7	22	9	24	27	18	21	19	23	33	20	26	29	28	25	32	31	30	34
Latvia	1	3	2	12	6	14	4	5	11	18	15	13	8	17	9	26	10	23	27	19	21	16	25	33	20	24	30	28	22	32	31	29	34
Estonia	1	3	2	12	6	16	4	5	8	15	14	13	11	18	10	22	7	24	27	25	19	21	20	33	17	26	29	28	23	32	31	30	34
High-income Asia Pacific	1	2	3	4	10	9	5	8	12	19	11	20	13	18	15	6	14	23	21	25	16	22	17	29	30	27	28	24	32	26	33	31	34
Japan	1	2	3	4	10	9	5	8	12	19	11	22	13	18	15	6	14	23	20	27	16	21	17	29	30	26	28	24	32	25	33	31	34
South Korea	1	3	4	2	8	9	5	10	11	15	12	16	13	21	17	6	14	24	20	23	18	26	19	28	29	25	27	22	32	31	33	30	34
Singapore	1	5	2	3	4	13	6	8	9	16	10	19	18	22	12	15	14	24	23	25	21	20	17	11	30	29	26	27	32	28	33	31	34
Brunei	1	5	2	4	3	21	9	11	8	10	7	12	20	14	13	16	17	22	24	26	19	25	15	18	27	30	23	28	34	32	31	29	33
Central SSA	4	7	8	2	3	9	11	6	10	1	12	13	14	18	15	19	22	27	17	21	20	16	24	30	29	25	23	26	34	33	28	31	32
DRC	7	4	8	2	3	9	11	5	10	1	12	13	14	18	15	19	25	28	17	21	20	16	23	30	29	24	22	26	34	33	27	31	32
Angola	4	8	6	2	3	9	11	7	10	1	12	13	15	16	14	20	22	26	18	21	19	17	23	30	27	25	24	28	34	32	29	31	33
CAR	4	2	5	6	3	8	11	7	10	1	12	15	13	16	18	20	24	28	17	19	21	14	26	30	29	22	25	27	34	33	23	31	32

© 2018 Fitzmaurice C et.al. JAMA Oncology.

Country	Tracheal, bronchus, and lung cancer	Stomach cancer	Colon and rectum cancer	Liver cancer	Breast cancer	Esophageal cancer	Pancreatic cancer	Prostate cancer	Leukemia	Cervical cancer	Non-Hodgkin lymphoma	Brain and nervous system cancer	Bladder cancer	Lip and oral cavity cancer	Ovarian cancer	Gallbladder and biliary tract cancer	Kidney cancer	Other pharynx cancer	Other leukemia	Larynx cancer	Multiple myeloma	Uterine cancer	Acute myeloid leukemia	Nasopharynx cancer	Malignant skin melanoma	Non-melanoma skin cancer	Acute lymphoid leukemia	Thyroid cancer	Chronic lymphoid leukemia	Mesothelioma	Hodgkin lymphoma	Chronic myeloid leukemia	Testicular cancer
Congo	4	9	5	2	3	8	10	6	12	1	11	14	15	17	13	19	21	26	20	22	18	16	23	30	27	25	24	28	33	32	29	31	34
Gabon	6	10	4	1	2	7	9	3	12	5	11	15	13	16	14	20	19	26	23	21	17	18	22	29	24	25	28	27	33	32	30	31	34
Equatorial Guinea	2	10	4	1	3	8	9	5	12	6	11	14	15	16	13	21	18	23	25	19	17	20	22	29	24	26	27	28	33	32	30	31	34
Central Europe	1	5	2	8	3	17	4	6	10	14	16	11	9	18	13	15	12	20	27	19	23	22	24	31	21	26	29	28	25	33	30	32	34
Poland	1	3	2	14	4	17	5	6	9	15	16	11	8	19	12	13	10	25	26	18	21	22	23	33	20	27	29	28	24	30	31	32	34
Romania	1	3	2	6	4	19	5	7	12	9	18	10	11	14	13	20	16	17	24	15	26	22	23	28	25	21	29	30	27	33	31	32	34
Czech Republic	1	7	2	10	4	16	3	5	9	17	15	14	12	18	13	11	6	23	24	26	19	21	22	33	20	27	30	28	25	31	29	32	34
Hungary	1	5	2	10	3	16	4	6	8	19	17	14	9	12	13	15	11	18	26	20	24	23	22	28	21	27	30	29	25	34	32	31	33
Serbia	1	6	2	8	3	18	4	5	11	12	16	9	10	19	13	15	14	24	27	17	25	22	20	31	21	23	29	30	26	34	28	32	33
Bulgaria	1	3	2	7	4	19	5	6	11	13	17	8	10	18	12	20	15	24	25	14	26	16	22	32	21	23	30	29	27	34	28	31	33
Slovakia	1	5	2	8	3	17	4	6	11	19	18	10	14	15	13	12	9	16	27	22	23	20	24	31	21	26	28	29	25	34	30	33	32
Croatia	1	4	2	7	3	16	6	5	10	19	14	9	11	17	13	15	12	22	27	20	21	23	24	33	18	26	31	29	25	28	32	30	34
Bosnia and Herzegovina	1	3	2	6	4	17	5	7	10	15	18	8	11	19	13	12	14	24	27	16	25	20	22	32	21	26	28	29	23	34	30	31	33
Albania	1	2	6	3	9	15	8	4	10	14	17	7	22	18	16	21	13	23	11	12	25	20	26	29	24	19	30	27	34	31	28	33	32
Macedonia	1	3	2	6	4	20	5	7	11	14	18	9	10	21	13	17	19	27	24	12	26	16	22	33	15	23	29	31	25	34	28	30	32
Slovenia	1	6	2	7	4	17	5	3	8	20	11	15	10	23	13	14	12	18	27	24	19	21	25	33	16	26	29	31	22	28	32	30	34
Montenegro	1	6	2	7	3	15	5	4	9	13	19	8	11	18	12	17	16	26	31	10	25	23	20	34	22	24	28	29	21	33	27	30	32
Central Asia	1	2	5	3	4	6	8	12	10	11	15	9	16	17	13	22	14	24	19	18	27	20	21	29	26	25	23	30	31	34	28	32	33
Uzbekistan	2	1	5	4	3	8	11	14	9	10	12	6	21	13	17	27	16	23	19	15	28	20	18	26	29	25	22	33	30	34	24	31	32
Kazakhstan	1	2	3	6	4	5	7	13	10	9	18	12	16	15	11	20	14	22	25	19	26	17	21	28	23	27	24	29	31	33	30	32	34
Azerbaijan	1	2	4	3	6	5	7	11	8	12	18	10	17	20	16	19	15	27	13	14	25	22	21	30	26	24	23	29	31	34	28	33	32
Tajikistan	4	1	6	5	8	2	10	11	7	13	15	9	16	17	12	21	19	24	14	23	28	22	20	29	25	26	18	30	31	34	27	33	32
Kyrgyzstan	2	1	6	3	4	9	8	13	10	7	17	11	18	15	12	21	14	25	19	23	27	16	20	29	26	22	24	28	32	34	30	33	31
Turkmenistan	3	1	8	6	4	2	11	16	10	9	18	7	21	13	14	17	12	19	15	22	23	26	20	25	28	27	24	31	32	34	29	33	30
Georgia	1	2	4	5	3	16	7	8	9	11	17	12	10	18	15	21	19	25	20	13	28	14	24	32	22	23	27	30	29	33	26	34	31

© 2018 Fitzmaurice C et.al. JAMA Oncology.

Country	Tracheal, bronchus, and lung cancer	Stomach cancer	Colon and rectum cancer	Liver cancer	Breast cancer	Esophageal cancer	Pancreatic cancer	Prostate cancer	Leukemia	Cervical cancer	Non-Hodgkin lymphoma	Brain and nervous system cancer	Bladder cancer	Lip and oral cavity cancer	Ovarian cancer	Gallbladder and biliary tract cancer	Kidney cancer	Other pharynx cancer	Other leukemia	Larynx cancer	Multiple myeloma	Uterine cancer	Acute myeloid leukemia	Nasopharynx cancer	Malignant skin melanoma	Non-melanoma skin cancer	Acute lymphoid leukemia	Thyroid cancer	Chronic lymphoid leukemia	Mesothelioma	Hodgkin lymphoma	Chronic myeloid leukemia	Testicular cancer
Armenia	1	3	4	5	2	19	6	8	11	12	17	9	10	21	13	20	16	28	15	14	25	18	22	31	29	23	24	27	30	26	33	32	34
Mongolia	3	2	7	1	8	4	9	17	11	6	12	10	23	15	13	14	16	27	25	20	26	18	19	30	32	21	22	24	29	33	31	28	34
Southern SSA	1	9	6	8	5	3	10	4	11	2	12	18	13	15	14	25	24	28	17	19	20	21	23	30	22	16	27	29	33	26	31	32	34
South Africa	1	11	6	8	4	3	9	5	10	2	15	18	17	12	14	24	21	27	13	19	20	23	22	29	25	16	28	31	33	26	30	32	34
Zimbabwe	8	5	7	3	6	4	11	2	13	1	9	15	12	24	14	25	27	29	22	19	18	16	23	31	20	17	26	21	33	32	28	30	34
Namibia	6	10	4	12	2	16	9	3	11	5	8	15	19	7	17	26	24	20	25	18	22	23	21	29	14	13	28	30	34	27	31	32	33
Botswana	1	9	8	7	6	3	10	4	11	2	13	15	19	12	16	25	23	26	17	18	20	24	21	28	22	14	29	31	34	27	30	32	33
Lesotho	1	6	7	9	4	3	11	5	10	2	18	17	16	14	19	22	23	30	12	15	21	20	24	28	25	13	29	32	34	26	27	31	33
Swaziland	1	9	7	8	6	3	11	4	10	2	16	15	18	12	19	24	23	27	14	17	20	21	22	28	25	13	26	31	34	29	30	32	33
Southern Latin America	1	3	2	11	4	10	6	5	12	9	14	16	15	22	17	8	13	29	18	21	19	20	23	34	24	25	26	27	28	31	32	33	30
Argentina	1	4	2	12	3	10	6	5	11	8	14	16	15	22	17	9	13	30	18	19	21	20	23	34	24	25	26	27	28	29	32	33	31
Chile	2	1	3	9	6	11	7	4	12	10	14	18	15	24	17	5	13	29	19	25	16	22	20	34	23	21	26	27	30	32	33	31	28
Uruguay	1	4	2	17	5	9	6	3	11	10	14	16	13	22	15	7	12	27	19	20	18	21	23	32	24	26	31	28	25	33	30	29	34
Andean Latin America	2	1	4	5	7	19	10	3	8	6	11	13	21	24	15	12	17	28	14	27	18	16	22	34	26	25	20	23	33	32	29	31	30
Peru	2	1	4	5	7	20	9	3	8	6	11	13	19	23	15	12	16	28	14	27	18	17	21	34	26	24	22	25	33	30	29	32	31
Ecuador	3	1	5	4	9	21	10	2	8	7	11	12	22	25	17	13	18	28	14	27	19	15	20	33	26	24	16	23	34	32	29	30	31
Bolivia	2	1	4	6	7	17	10	3	8	5	12	13	19	25	16	11	20	27	14	26	18	15	23	34	28	24	22	21	33	32	29	31	30
Caribbean	1	5	3	8	4	12	10	2	9	7	11	14	15	18	20	23	21	25	17	13	19	16	24	28	29	22	26	30	32	33	27	31	34
Cuba	1	6	3	8	4	10	7	2	12	13	15	14	11	17	20	22	21	23	25	9	18	16	24	30	29	19	31	32	28	33	27	26	34
Haiti	5	4	6	10	2	14	12	1	7	3	11	15	18	21	17	16	24	27	9	19	20	13	25	30	31	26	22	29	32	33	23	28	34
Dominican Republic	2	7	3	4	5	15	10	1	9	8	14	12	22	13	21	23	26	18	11	16	17	20	24	25	29	19	27	28	31	33	30	32	34
Jamaica	2	5	3	11	4	15	10	1	8	6	9	18	17	22	14	20	21	25	12	19	16	13	23	29	28	27	24	26	31	33	32	30	34
Trinidad and Tobago	4	8	2	11	3	18	7	1	9	6	12	16	19	20	10	23	17	24	15	22	14	13	21	28	29	27	25	26	32	33	31	30	34
Guyana	6	7	4	8	2	15	10	1	9	3	12	16	21	20	11	19	17	27	13	22	18	14	30	32	24	25	23	29	33	31	28	26	34
Suriname	2	8	1	6	4	21	9	3	10	5	12	11	17	19	13	16	18	29	14	22	15	20	23	24	26	27	30	25	32	33	28	31	34

© 2018 Fitzmaurice C et.al. JAMA Oncology.

Country	Tracheal, bronchus, and lung cancer	Stomach cancer	Colon and rectum cancer	Liver cancer	Breast cancer	Esophageal cancer	Pancreatic cancer	Prostate cancer	Leukemia	Cervical cancer	Non-Hodgkin lymphoma	Brain and nervous system cancer	Bladder cancer	Lip and oral cavity cancer	Ovarian cancer	Gallbladder and biliary tract cancer	Kidney cancer	Other pharynx cancer	Other leukemia	Larynx cancer	Multiple myeloma	Uterine cancer	Acute myeloid leukemia	Nasopharynx cancer	Malignant skin melanoma	Non-melanoma skin cancer	Acute lymphoid leukemia	Thyroid cancer	Chronic lymphoid leukemia	Mesothelioma	Hodgkin lymphoma	Chronic myeloid leukemia	Testicular cancer
The Bahamas	4	5	3	9	2	13	10	1	8	7	14	18	22	16	12	20	21	24	19	17	11	15	23	29	25	26	31	30	27	33	32	28	34
Belize	1	5	4	6	8	15	9	2	10	3	13	12	18	19	20	17	21	25	11	16	22	14	29	30	28	23	24	31	33	27	26	32	34
Barbados	4	5	2	12	3	14	7	1	8	9	11	17	18	21	16	20	19	24	15	23	10	13	22	28	26	27	31	25	30	33	32	29	34
Saint Lucia	3	5	4	11	2	13	8	1	9	7	10	20	15	17	16	22	21	24	14	19	12	18	23	26	27	29	30	25	28	34	31	32	33
Saint Vincent and the Grenadines	4	6	3	10	2	20	11	1	8	5	9	17	18	14	15	21	23	24	12	16	19	13	25	29	26	22	30	28	27	33	31	32	34
Grenada	4	8	2	11	3	12	9	1	10	7	6	16	19	18	17	20	23	25	14	21	15	13	22	30	26	27	29	24	28	32	33	31	34
Antigua and Barbuda	4	5	3	8	2	16	10	1	7	9	12	15	17	19	13	20	22	26	11	21	14	18	23	28	25	24	29	27	32	33	31	30	34
Dominica	2	3	5	9	4	14	8	1	7	11	10	23	15	16	19	18	21	22	13	17	12	20	24	28	27	26	25	32	31	33	29	30	34
Australasia	1	8	2	11	4	12	5	3	6	25	10	13	14	22	16	21	15	26	27	29	18	23	17	30	9	20	28	32	24	19	33	31	34
Australia	1	7	2	11	4	12	5	3	6	25	10	13	14	22	16	21	15	26	27	29	18	23	17	30	9	20	28	32	24	19	33	31	34
New Zealand	1	9	2	12	3	13	5	4	6	25	10	11	17	24	16	20	14	28	26	29	15	21	18	31	8	19	27	32	23	22	33	30	34
Oceania	1	3	6	5	2	17	11	9	8	4	12	16	22	14	19	24	29	18	13	21	23	10	15	20	31	25	27	28	34	33	30	26	32
Papua New Guinea	1	3	5	8	2	20	12	9	6	4	13	15	23	16	19	25	30	14	11	21	26	10	17	18	31	24	27	28	34	33	29	22	32
Fiji	6	9	4	3	1	15	11	8	7	2	13	16	17	14	19	23	26	22	18	24	20	12	10	29	27	28	25	21	34	33	32	30	31
Solomon Islands	1	4	6	3	2	15	11	9	8	5	12	17	22	14	19	26	28	18	13	21	24	10	16	20	31	23	27	29	34	33	30	25	32
Vanuatu	1	3	5	6	2	18	10	9	8	4	12	16	22	14	17	25	28	20	13	21	24	11	15	19	30	23	26	29	34	32	31	27	33
Samoa	4	2	3	7	1	21	8	5	10	9	11	14	18	25	13	26	27	23	16	28	19	12	20	24	15	22	30	17	34	33	32	29	31
Kiribati	1	5	7	3	2	12	10	13	8	11	9	14	26	4	16	25	15	18	17	20	28	21	19	23	30	27	29	32	34	33	31	24	22
Tonga	1	4	7	2	3	17	9	5	10	6	8	15	20	13	19	26	25	23	21	22	16	14	18	24	28	11	27	29	32	33	31	30	34
Federated States of Micronesia	1	4	3	5	2	18	10	7	9	8	12	14	22	15	17	25	28	21	16	20	19	11	13	23	30	24	26	29	34	32	31	27	33
Marshall Islands	1	3	4	5	2	17	10	9	7	8	11	15	23	16	21	26	28	20	14	19	22	12	13	18	29	25	24	30	34	33	31	27	32

Colors correspond to the ranking, with dark red as the most common cancer and dark green as the least common cancer for the location indicated. Rankings do not include the “other cancer” group. The numbers inside each box indicate the ranking. Abbreviations: SSA: Sub-Saharan Africa; DRC: Democratic Republic of Congo; CAR: Central African Republic

eFigure 16: Cancer ranking by total mortality based on global level for developing and developed regions and all countries, both sexes, 2016

© 2018 Fitzmaurice C et al. JAMA Oncology.

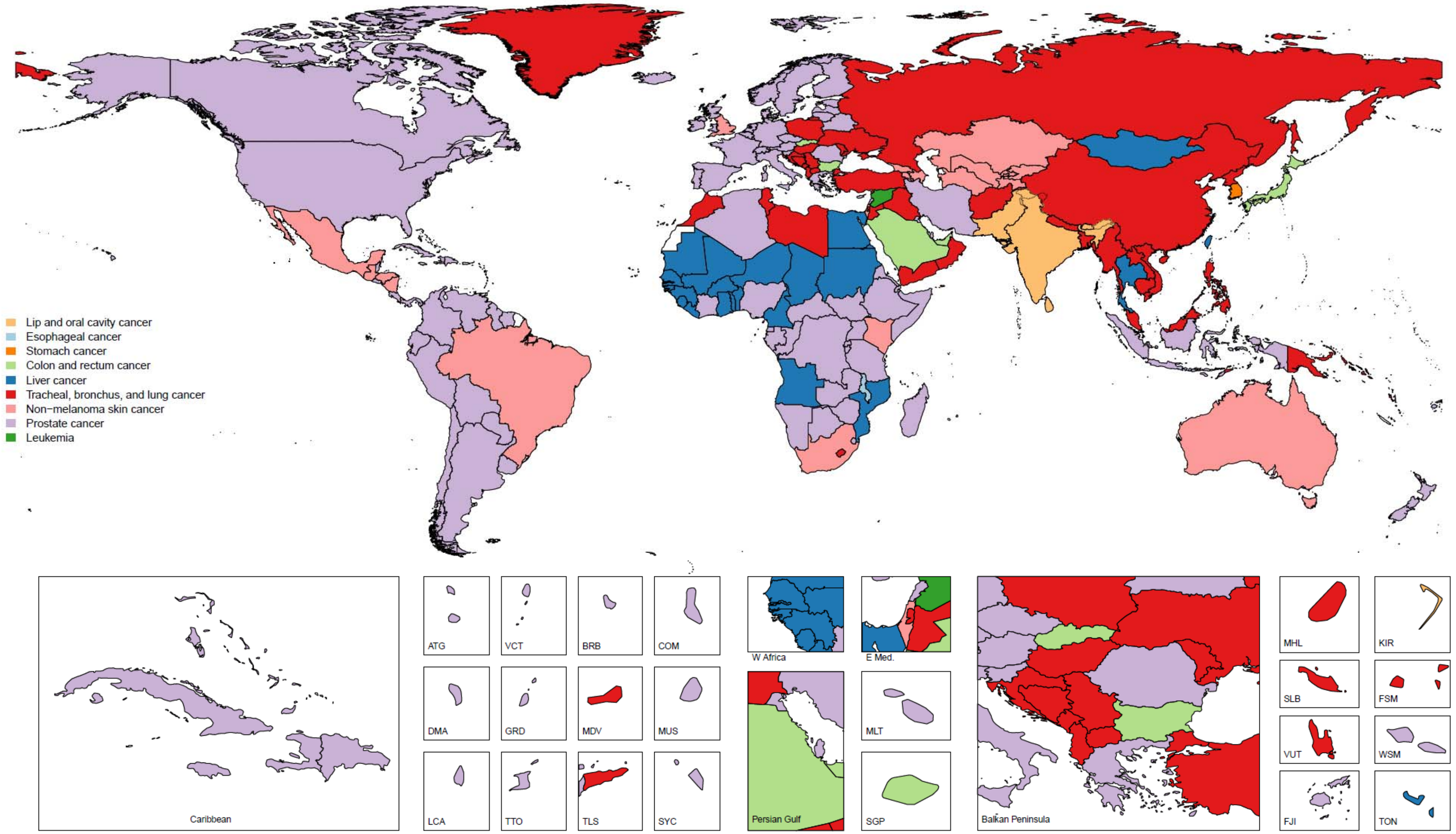

eFigure 17: Top-ranked cancers by absolute incident cases for all ages in males, 2016

© 2018 Fitzmaurice C et al. JAMA Oncology.

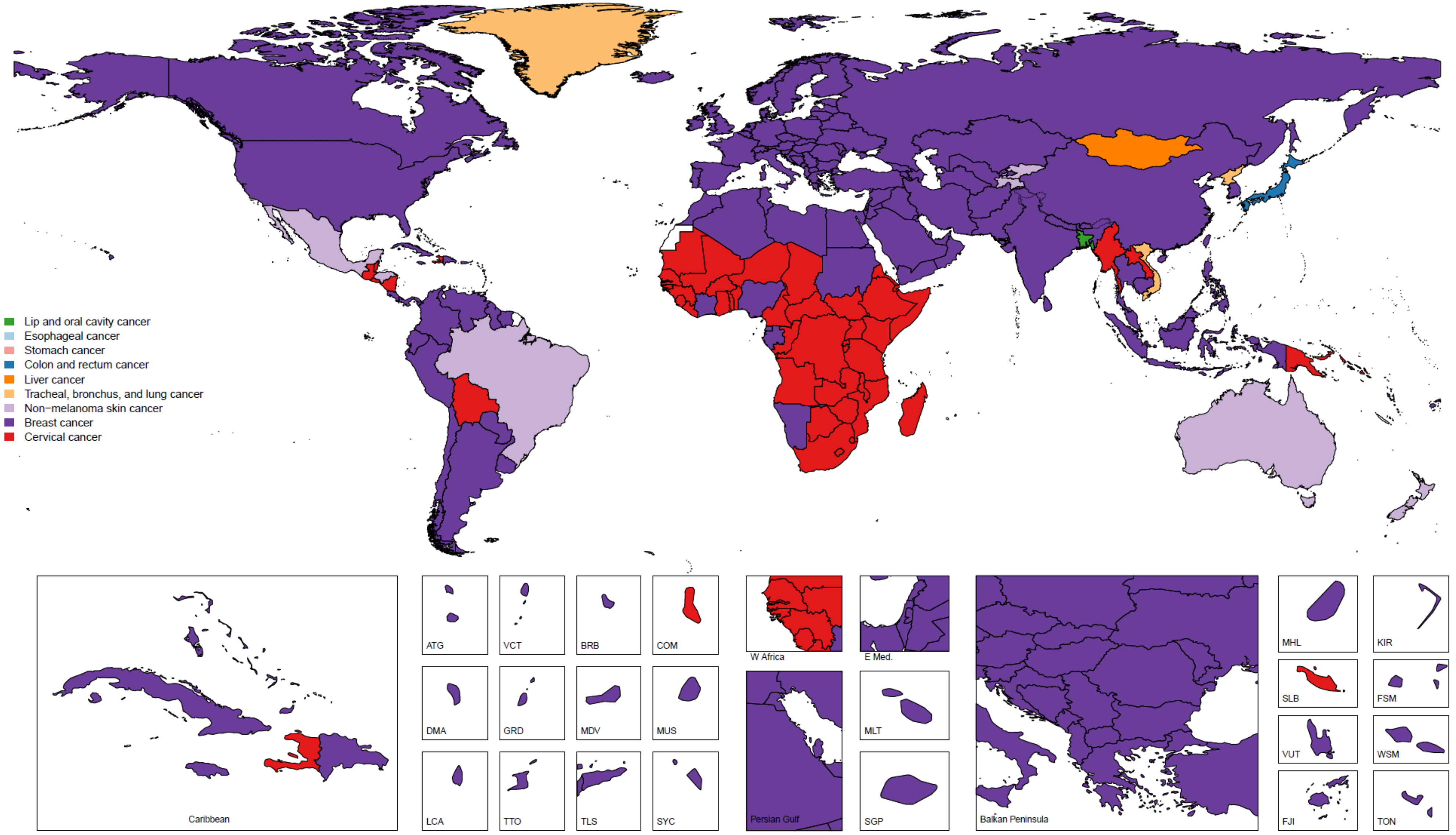

eFigure 18: Top-ranked cancers by absolute incident cases for all ages in females, 2016

© 2018 Fitzmaurice C et al. JAMA Oncology.

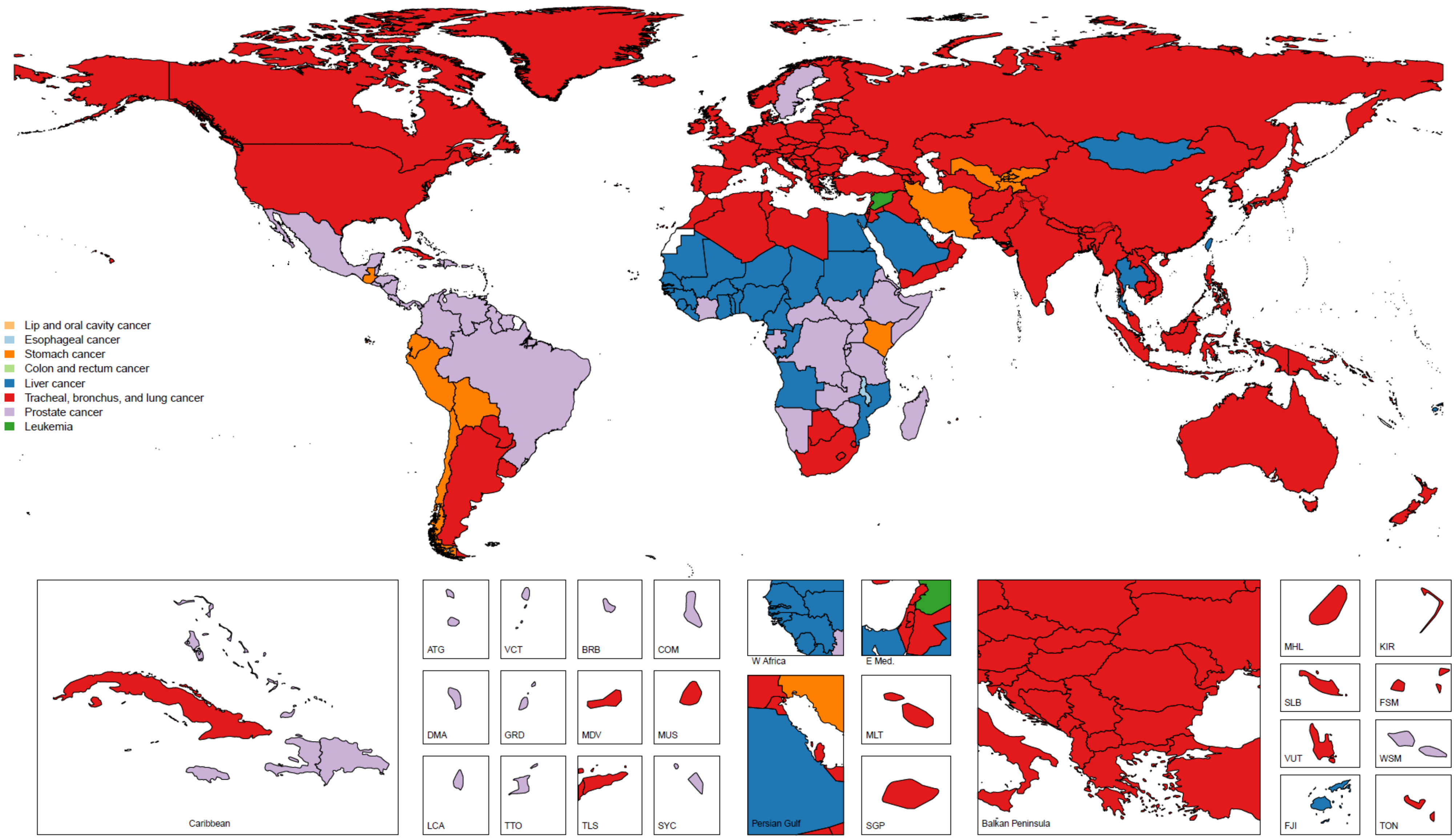

eFigure 19: Top-ranked cancers by absolute deaths for all ages in males, 2016

© 2018 Fitzmaurice C et al. JAMA Oncology.

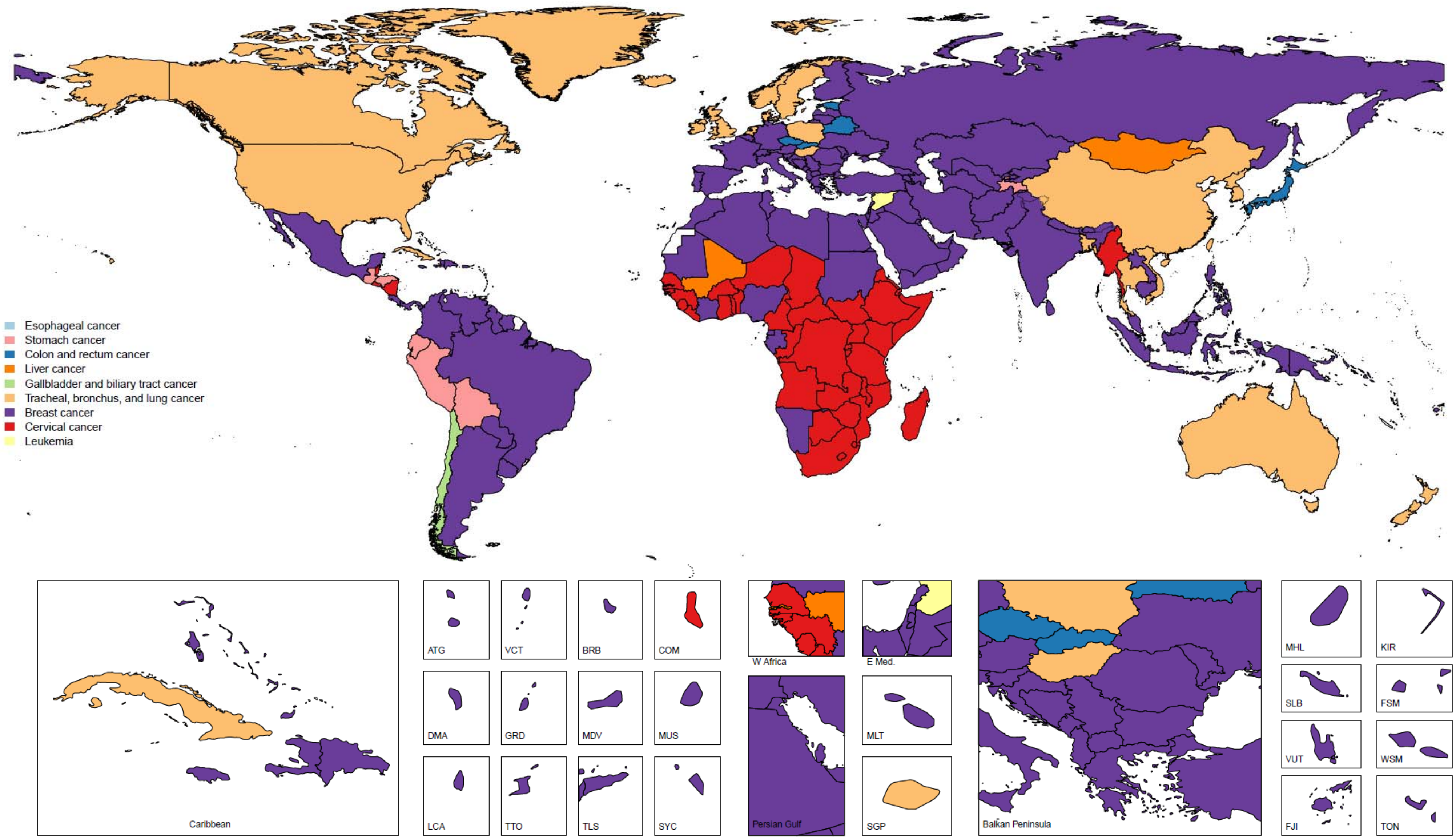

eFigure 20: Top-ranked cancers by absolute deaths for all ages in females, 2016

© 2018 Fitzmaurice C et al. JAMA Oncology.

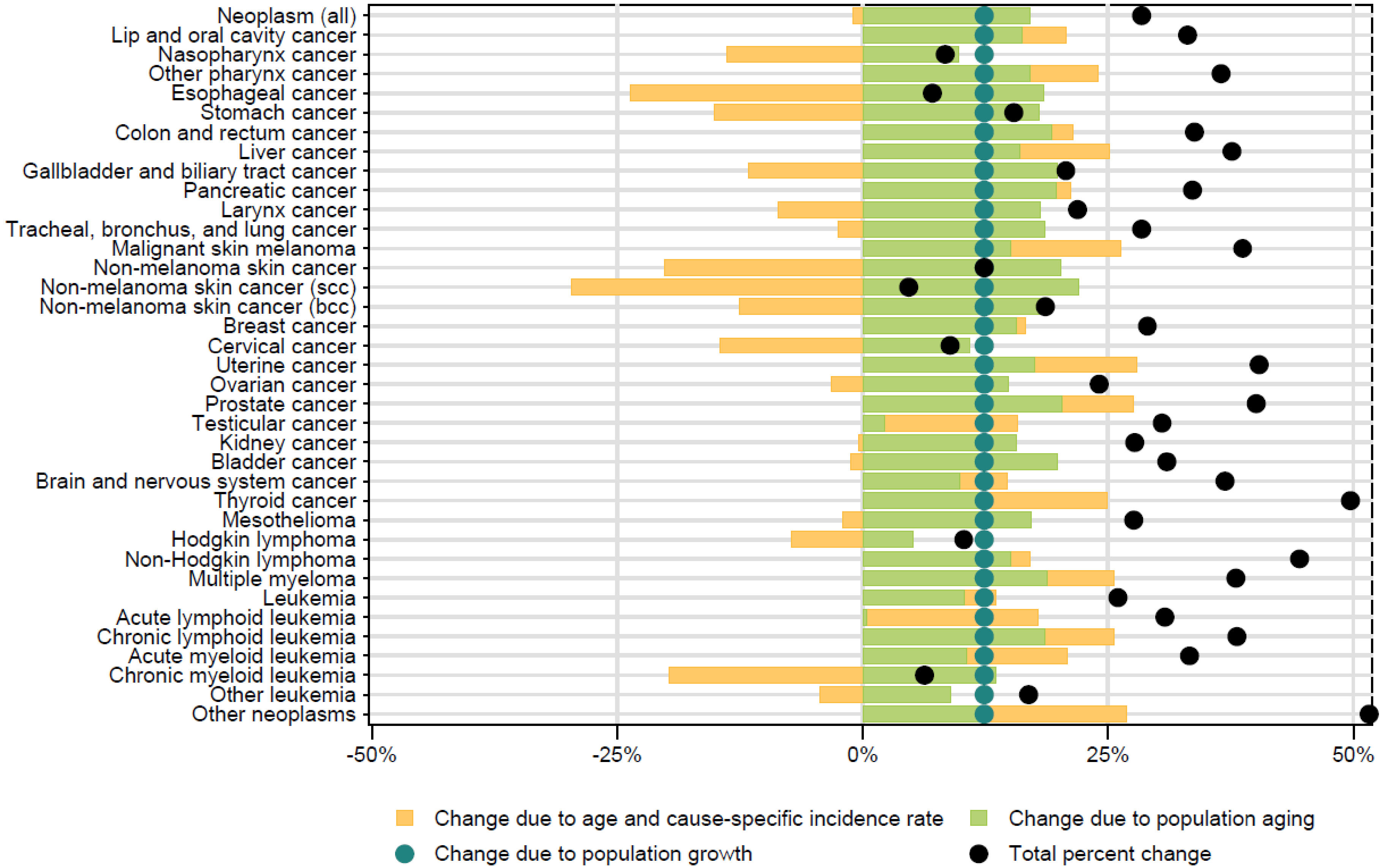

eFigure 21: Global Decomposition of Changes in Cancer Incident Cases due to Population Growth, Population Aging, and Changes in Age-specific Incidence Rates, Both Sexes, 2006 to 2016. Results are also presented in eTable 14. To estimate the effect of population growth we applied the population size of 2016 onto the rate, sex, and age structure of 2006. Since the global population grew by 12.4% between 2006 and 2016, and rates and age structure remained the same as in 2006, incidence due to all cancers increased by 12.4% in this counterfactual scenario. To estimate the effect of aging on incident cases we applied the age structure of 2016 onto the rate, sex distribution, and population size of 2006. The change in incident cases reported herein shows the proportion of the change in incident cases between 2006 and 2016 that can be attributed to the changing age structure of the population. To estimate the effect of changing incidence rates on the incident cases we applied the incidence rates for 2006 onto the population size and age structure of 2006. The change in incident cases reported herein shows the proportion of the change in incident cases between 2006 and 2016 that can be attributed to a change in incidence rates.

© 2018 Fitzmaurice C et al. JAMA Oncology.

For the following figures, the y-axes differ in scale between male and female graphs and by cancer.

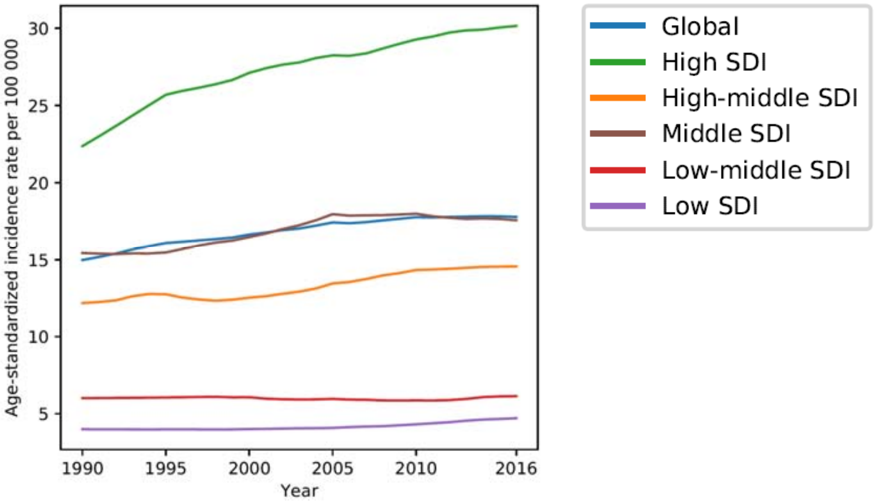

eFigure 22: Trends in Age-Standardized Incidence Rates for Tracheal, Bronchus, and Lung Cancer, 1990-2016, Female

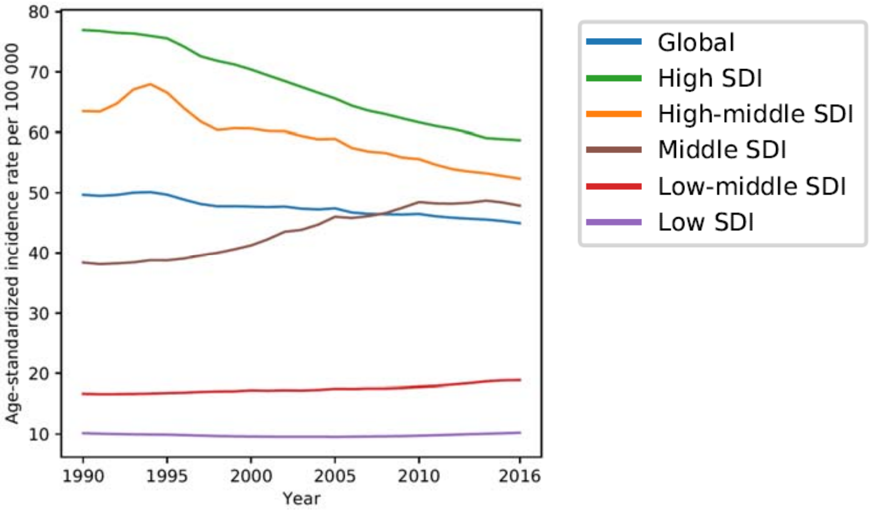

eFigure 23: Trends in Age-Standardized Incidence Rates for Tracheal, Bronchus, and Lung Cancer, 1990-2016, Male

© 2018 Fitzmaurice C et al. JAMA Oncology.

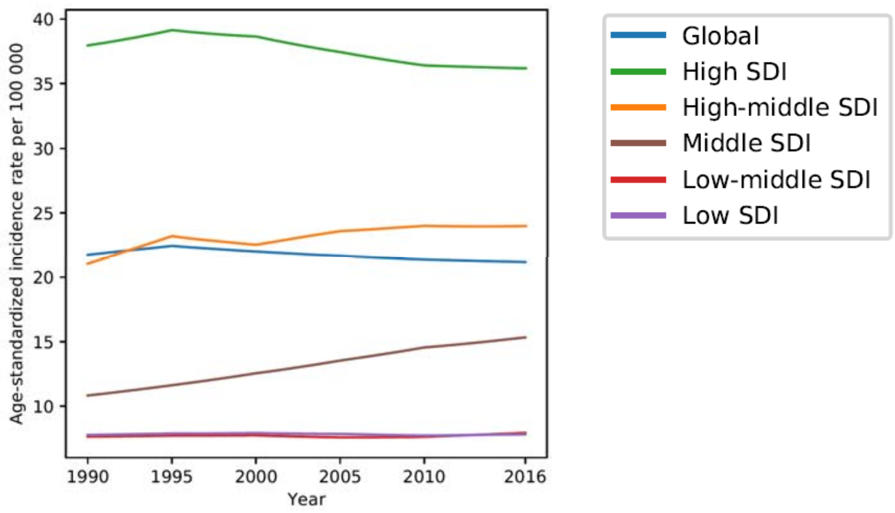

eFigure 24: Trends in Age-Standardized Incidence Rates for Colon and Rectum Cancer, 1990-2016, Female

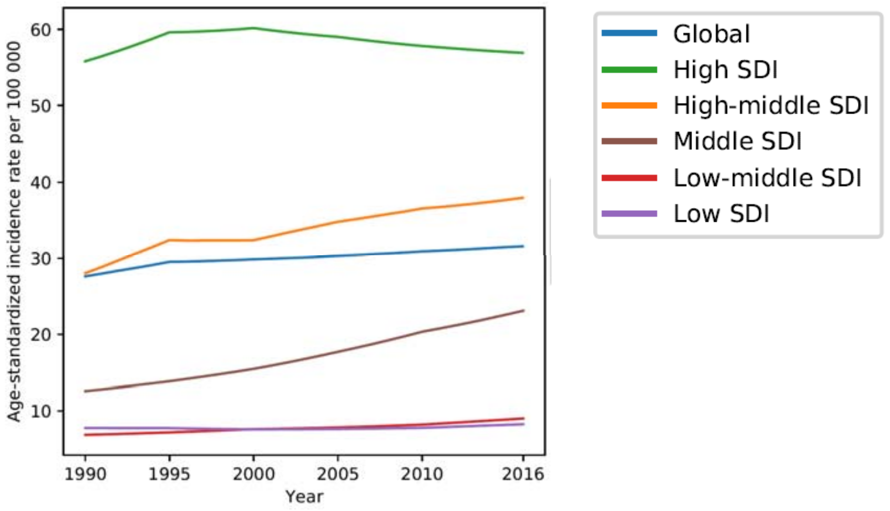

eFigure 25: Trends in Age-Standardized Incidence Rates for Colon and Rectum Cancer, 1990-2016, Male

© 2018 Fitzmaurice C et al. JAMA Oncology.

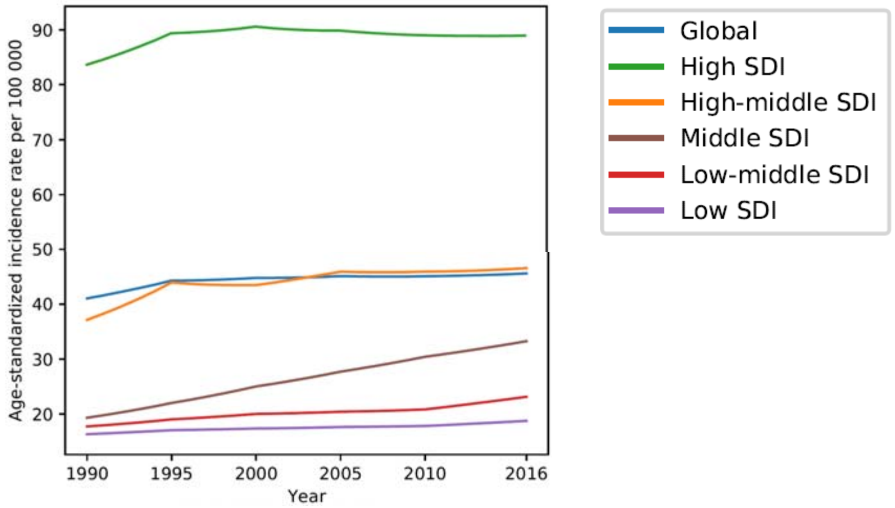

eFigure 26: Trends in Age-Standardized Incidence Rates for Breast Cancer, 1990-2016, Female

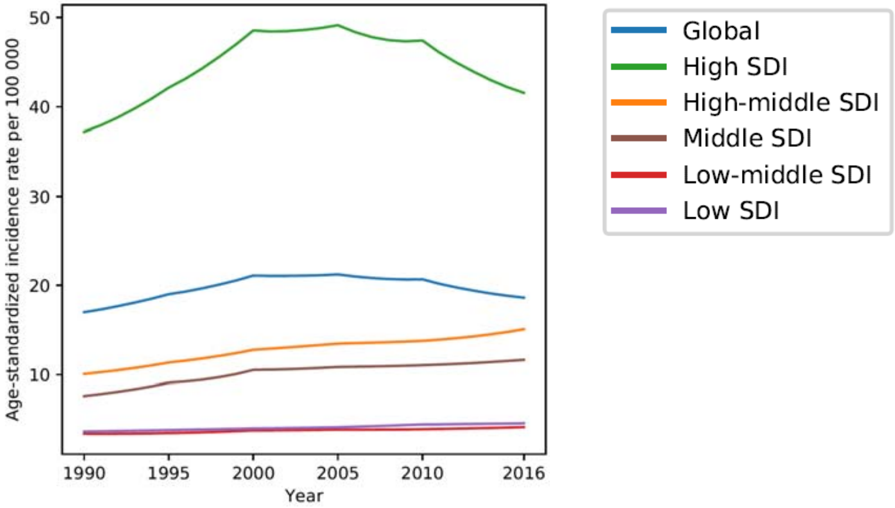

eFigure 27: Trends in Age-Standardized Incidence Rates for NMSC, 1990-2016, Female

© 2018 Fitzmaurice C et al. JAMA Oncology.

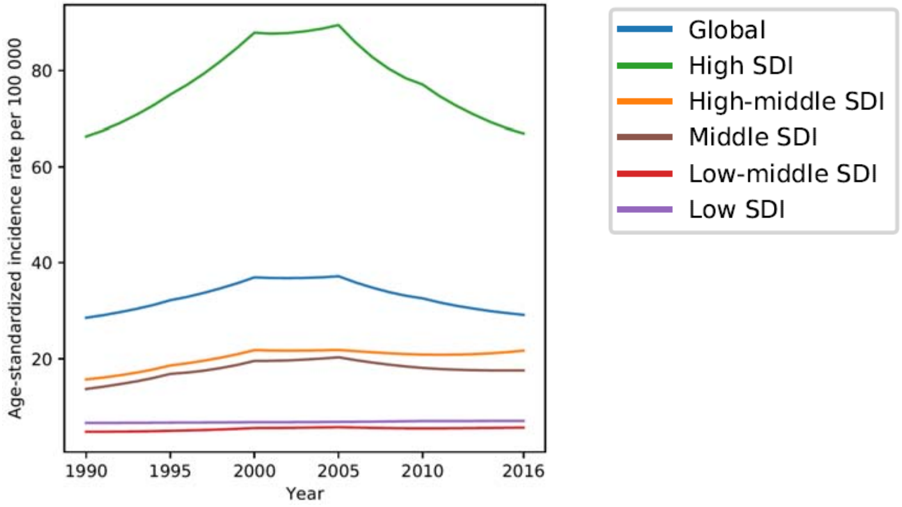

eFigure 28: Trends in Age-Standardized Incidence Rates for NMSC, 1990-2016, Male

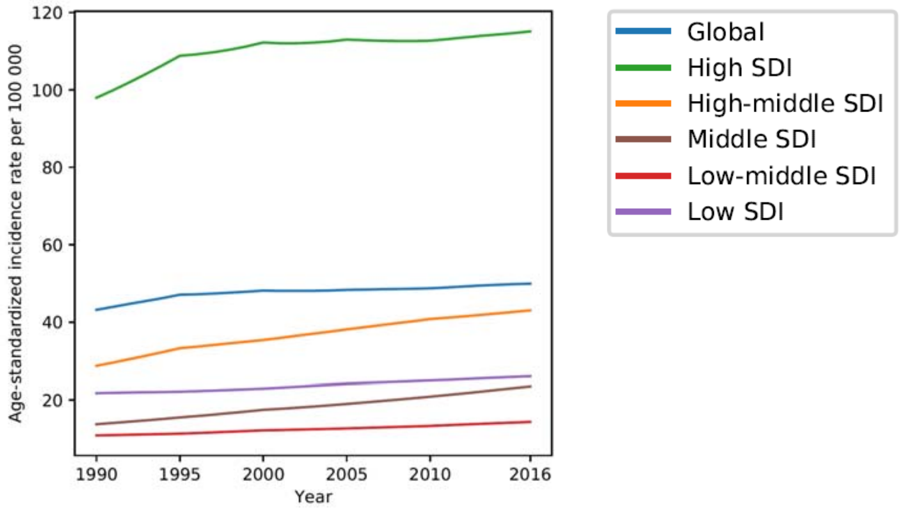

eFigure 29: Trends in Age-Standardized Incidence Rates for Prostate Cancer, 1990-2016, Male

© 2018 Fitzmaurice C et al. JAMA Oncology.

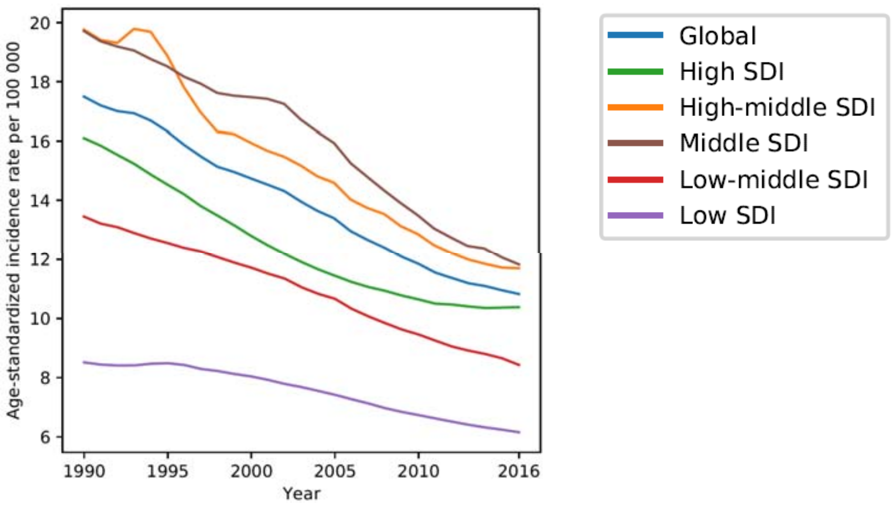

eFigure 30: Trends in Age-Standardized Incidence Rates for Stomach Cancer, 1990-2016, Female

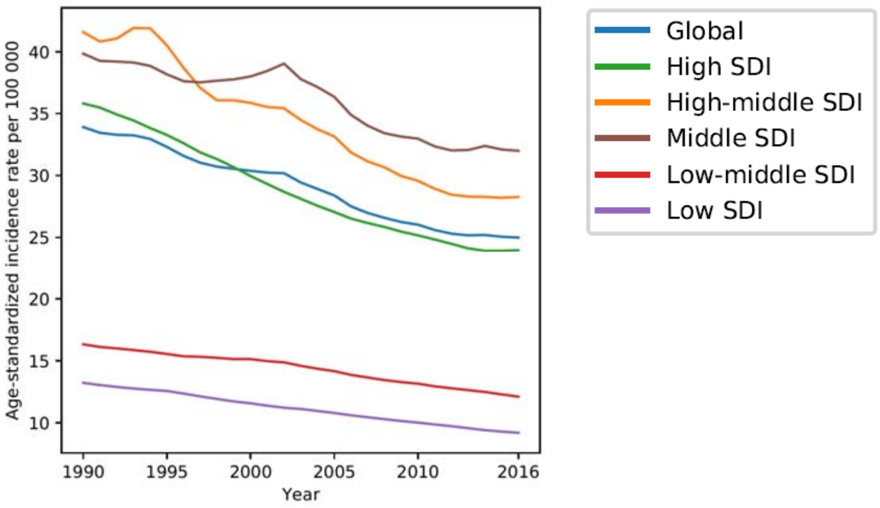

eFigure 31: Trends in Age-Standardized Incidence Rates for Stomach Cancer, 1990-2016, Male

© 2018 Fitzmaurice C et al. JAMA Oncology.

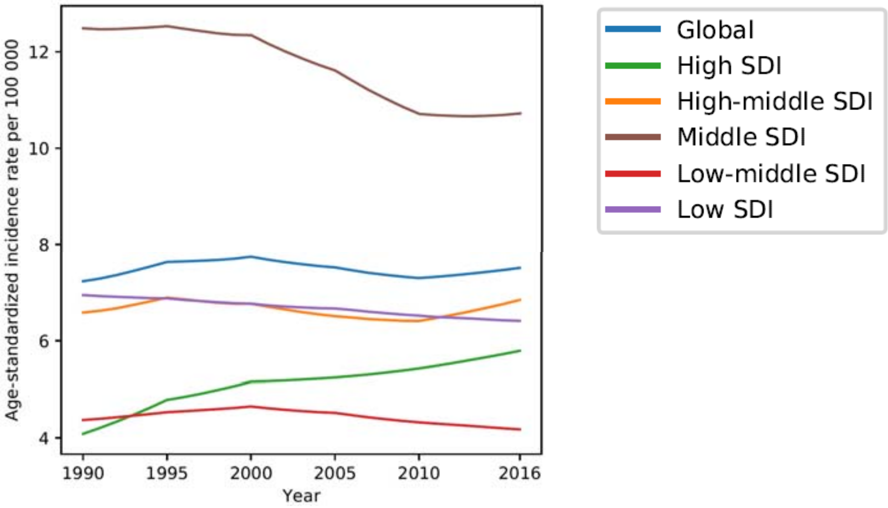

eFigure 32: Trends in Age-Standardized Incidence Rates for Liver Cancer, 1990-2016, Female

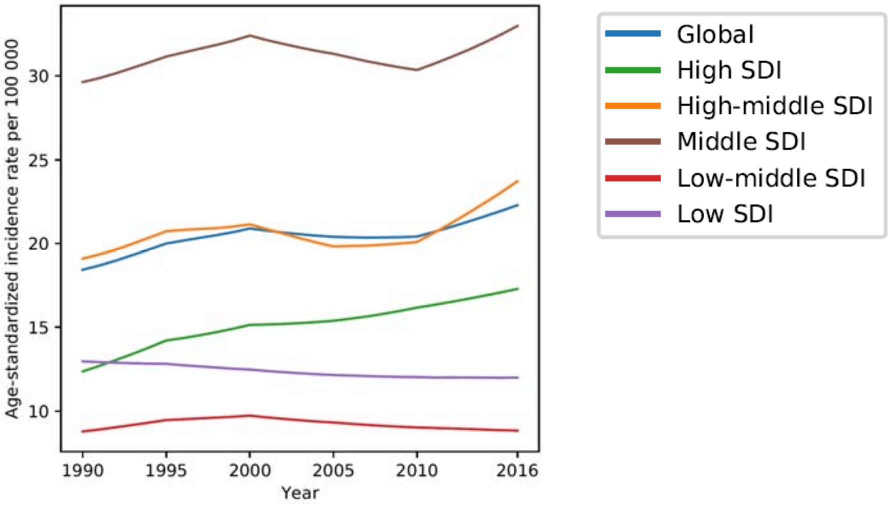

eFigure 33: Trends in Age-Standardized Incidence Rates for Liver Cancer, 1990-2016, Male

© 2018 Fitzmaurice C et al. JAMA Oncology.

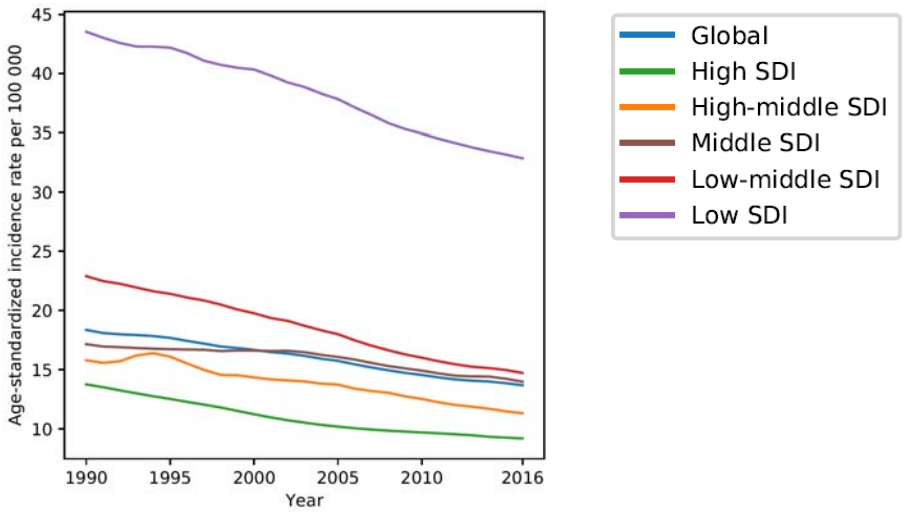

eFigure 34: Trends in Age-Standardized Incidence Rates for Cervical Cancer, 1990-2016, Female

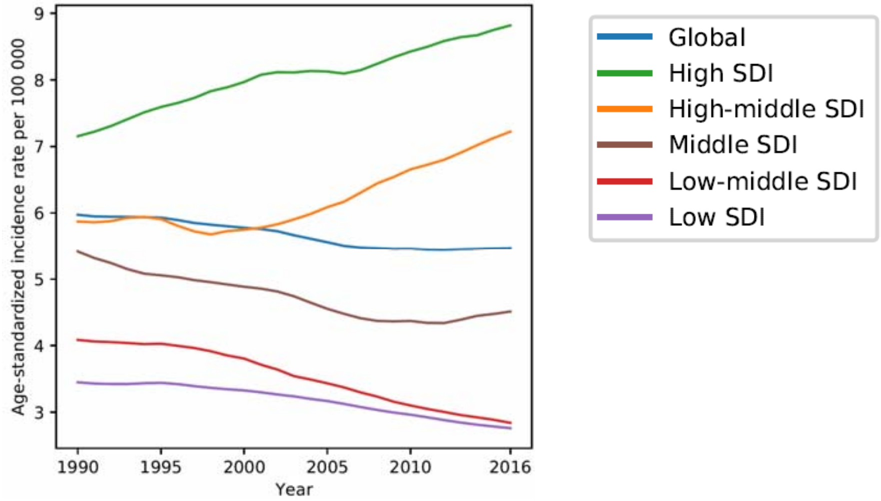

eFigure 35: Trends in Age-Standardized Incidence Rates for Leukemia, 1990-2016, Female

© 2018 Fitzmaurice C et al. JAMA Oncology.

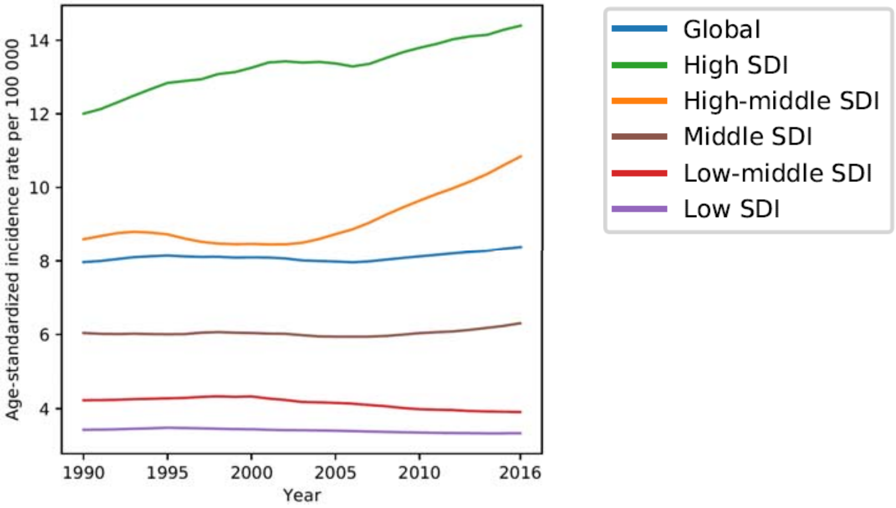

eFigure 36: Trends in Age-Standardized Incidence Rates for Leukemia, 1990-2016, Male

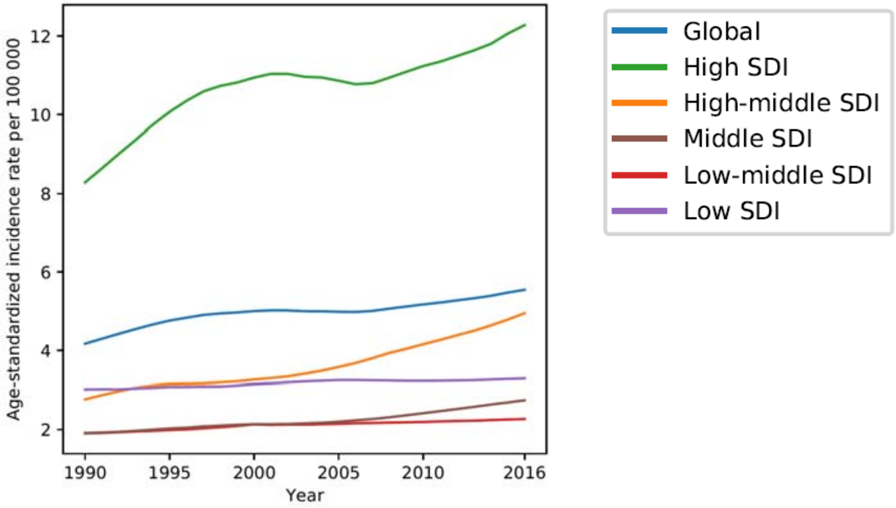

eFigure 37: Trends in Age-Standardized Incidence Rates for Non-Hodgkin lymphoma, 1990-2016, Female

© 2018 Fitzmaurice C et al. JAMA Oncology.

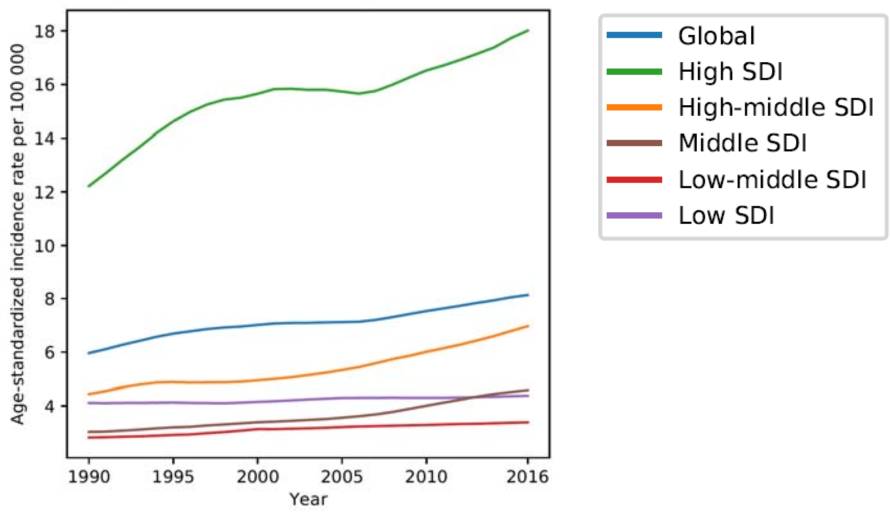

eFigure 38: Trends in Age-Standardized Incidence Rates for Non-Hodgkin lymphoma, 1990-2016, Male

© 2018 Fitzmaurice C et al. JAMA Oncology.

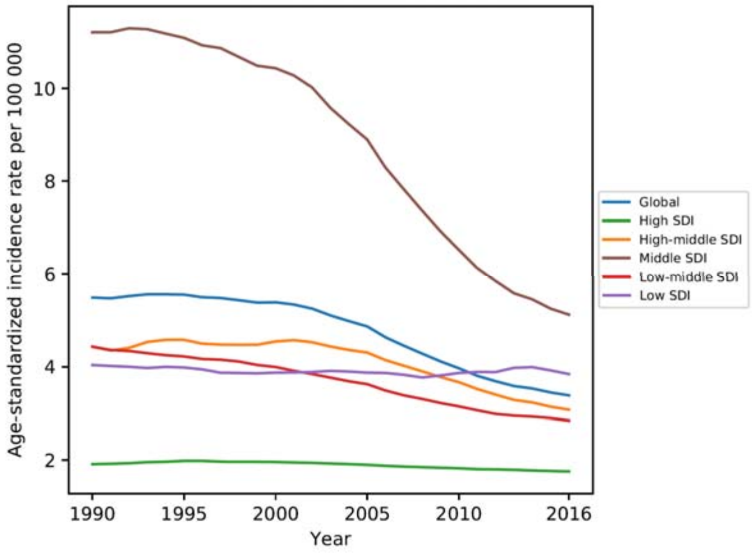

eFigure 39: Trends in Age-Standardized Incidence Rates for Esophageal Cancer, 1990-2016, Female

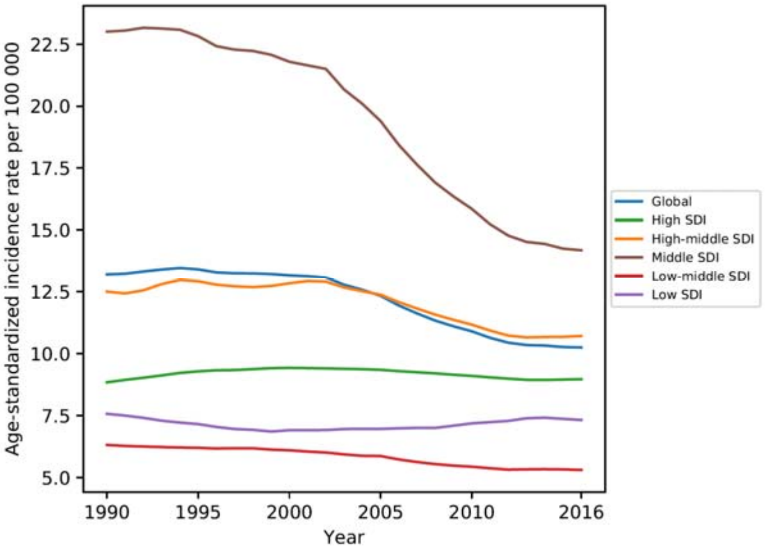

eFigure 40: Trends in Age-Standardized Incidence Rates for Esophageal Cancer, 1990-2016, Male

© 2018 Fitzmaurice C et al. JAMA Oncology.

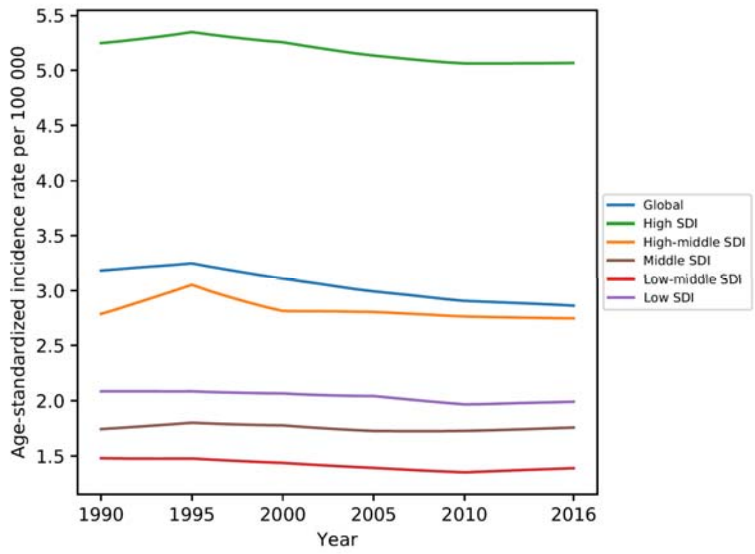

eFigure 41: Trends in Age-Standardized Incidence Rates for Bladder Cancer, 1990-2016, Female

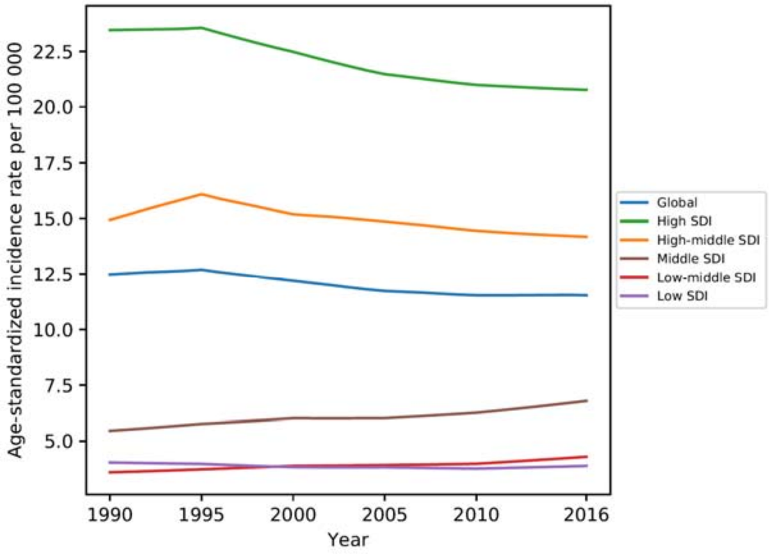

eFigure 42: Trends in Age-Standardized Incidence Rates for Bladder Cancer, 1990-2016, Male

© 2018 Fitzmaurice C et al. JAMA Oncology.

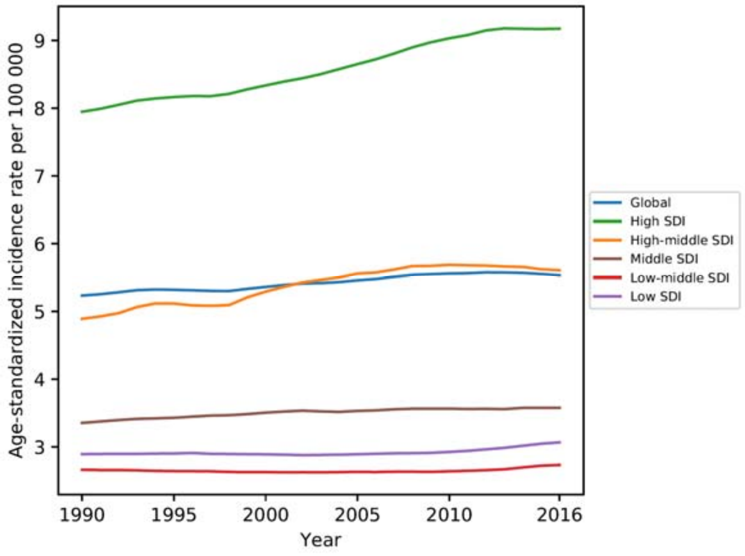

eFigure 43: Trends in Age-Standardized Incidence Rates for Pancreatic Cancer, 1990-2016, Female

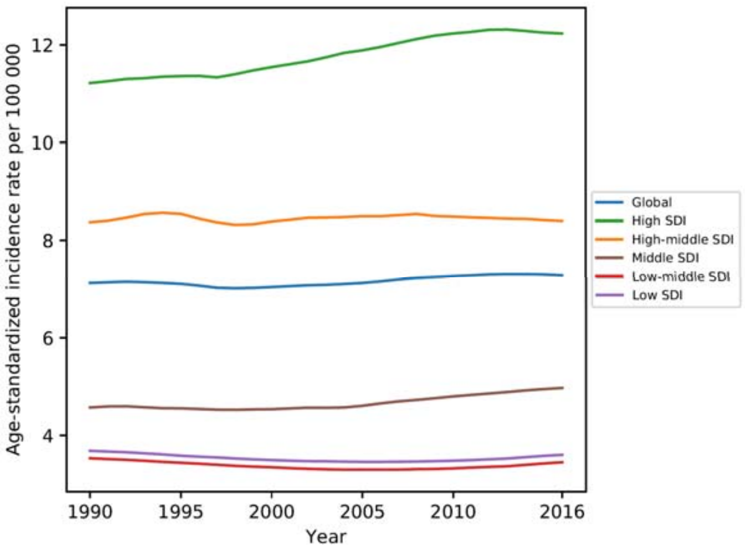

eFigure 44: Trends in Age-Standardized Incidence Rates for Pancreatic Cancer, 1990-2016, Male

© 2018 Fitzmaurice C et al. JAMA Oncology.

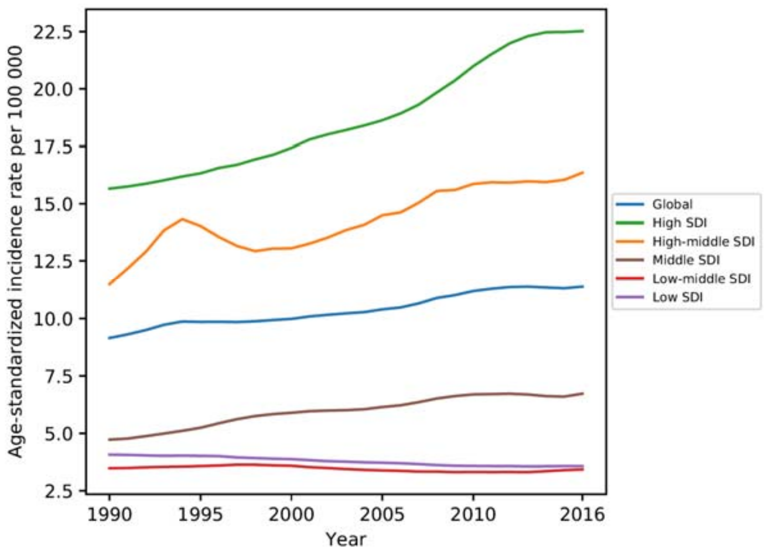

eFigure 45: Trends in Age-Standardized Incidence Rates for Uterine Cancer, 1990-2016, Female

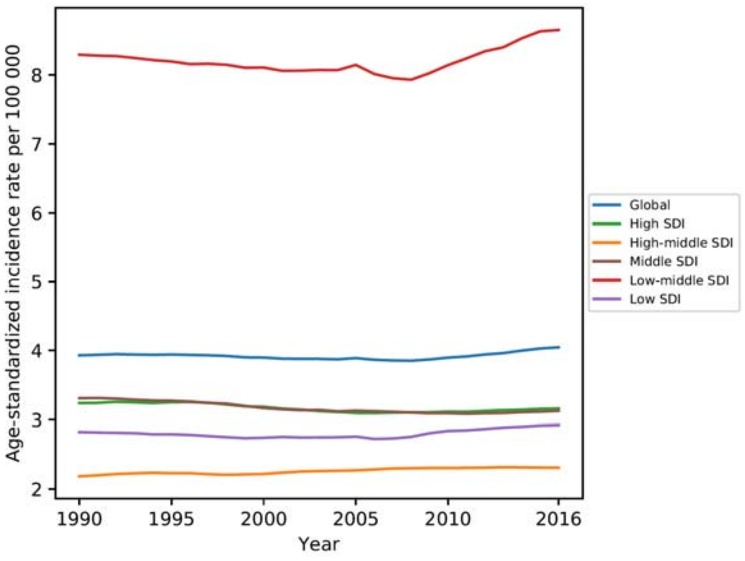

eFigure 46: Trends in Age-Standardized Incidence Rates for Lip and oral cavity Cancer, 1990-2016, Female

© 2018 Fitzmaurice C et al. JAMA Oncology.

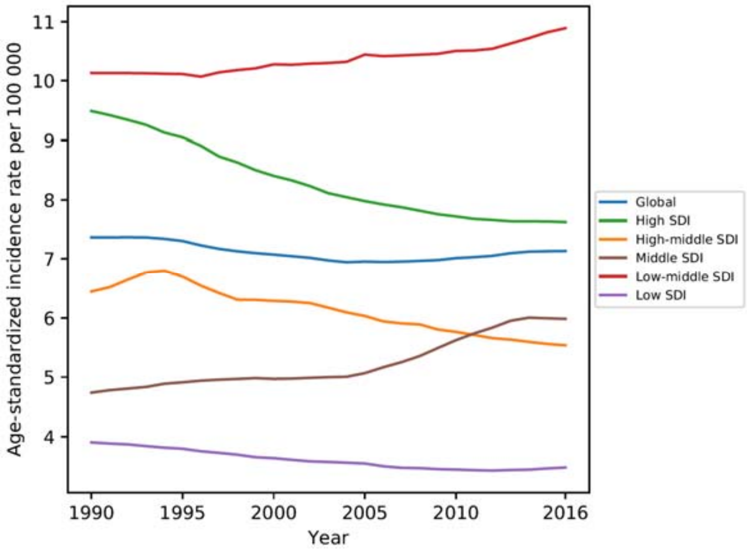

eFigure 47: Trends in Age-Standardized Incidence Rates for Lip and oral cavity Cancer, 1990-2016, Male

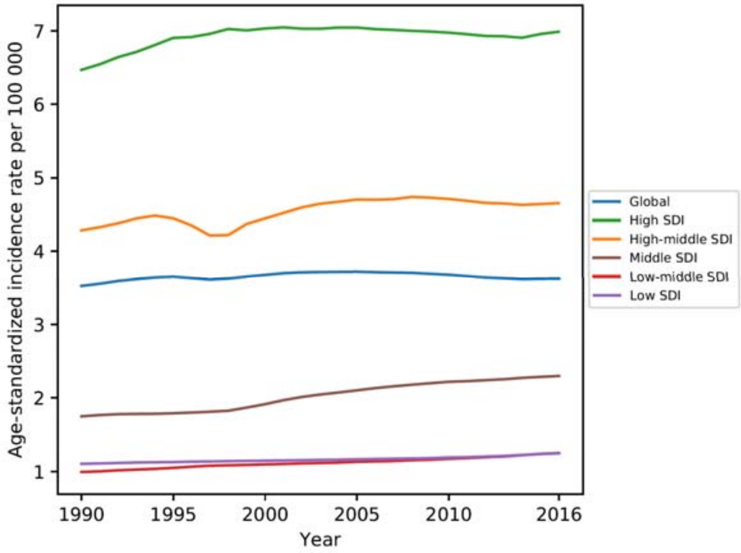

eFigure 48: Trends in Age-Standardized Incidence Rates for Kidney Cancer, 1990-2016, Female

© 2018 Fitzmaurice C et al. JAMA Oncology.

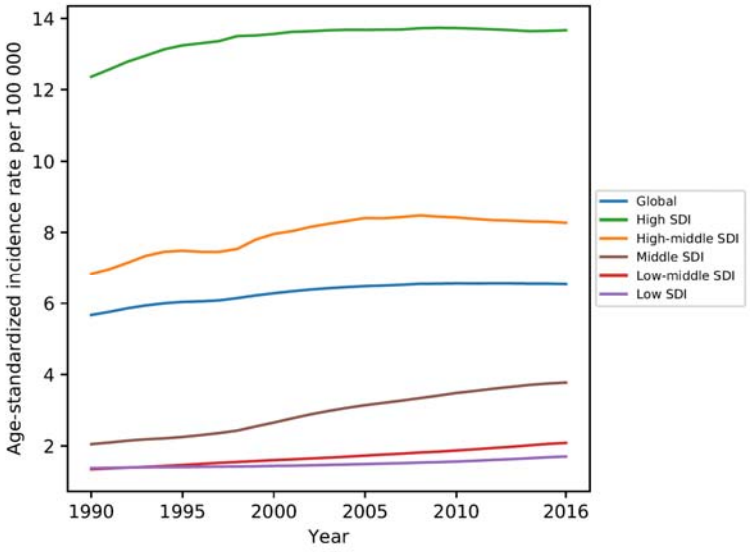

eFigure 49: Trends in Age-Standardized Incidence Rates for Kidney Cancer, 1990-2016, Male

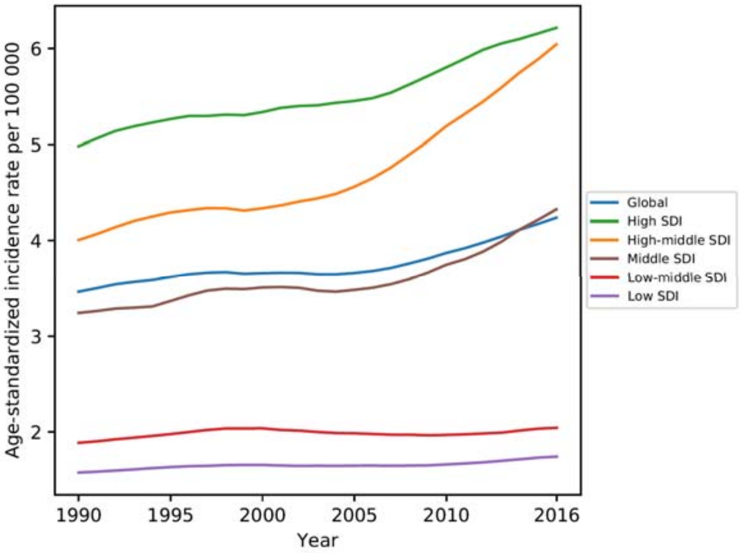

eFigure 50: Trends in Age-Standardized Incidence Rates for Brain and nervous system cancer, 1990-2016, Female

© 2018 Fitzmaurice C et al. JAMA Oncology.

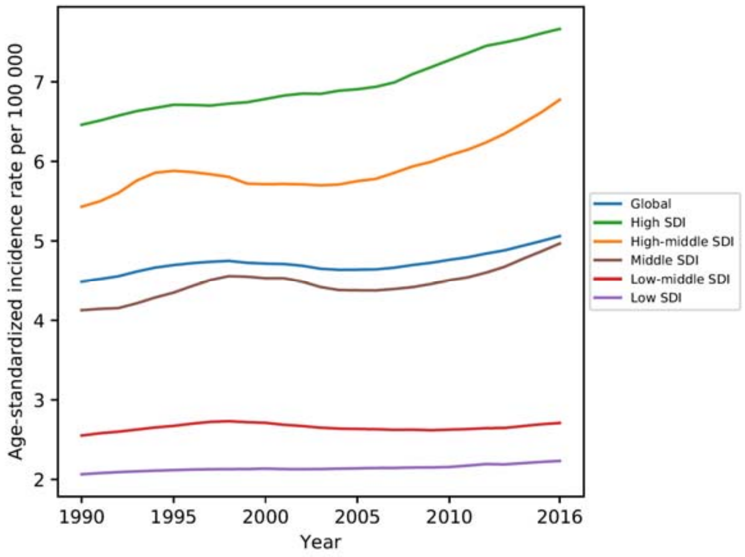

eFigure 51: Trends in Age-Standardized Incidence Rates for Brain and nervous system cancer, 1990-2016, Male

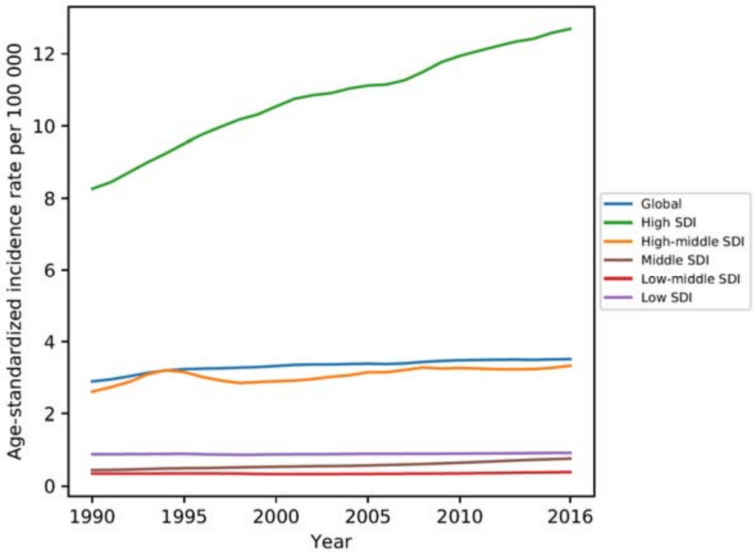

eFigure 52: Trends in Age-Standardized Incidence Rates for Melanoma, 1990-2016, Female

© 2018 Fitzmaurice C et al. JAMA Oncology.

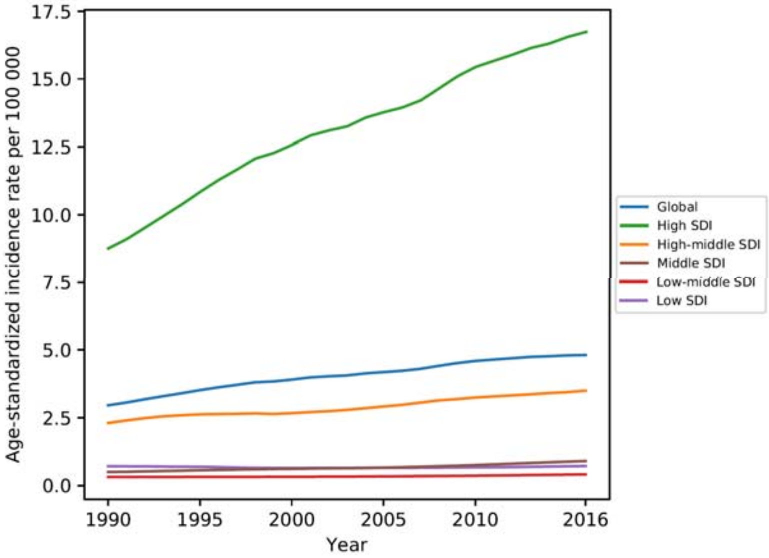

eFigure 53: Trends in Age-Standardized Incidence Rates for Melanoma, 1990-2016, Male

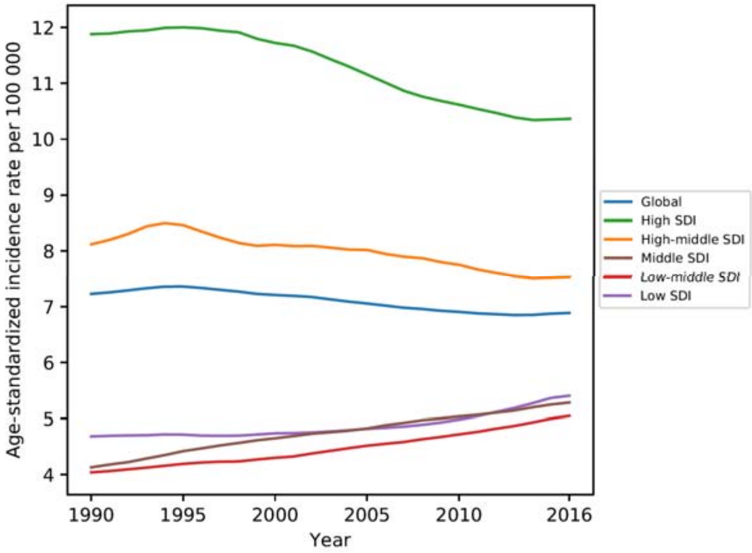

eFigure 54: Trends in Age-Standardized Incidence Rates for Ovarian Cancer, 1990-2016, Female

© 2018 Fitzmaurice C et al. JAMA Oncology.

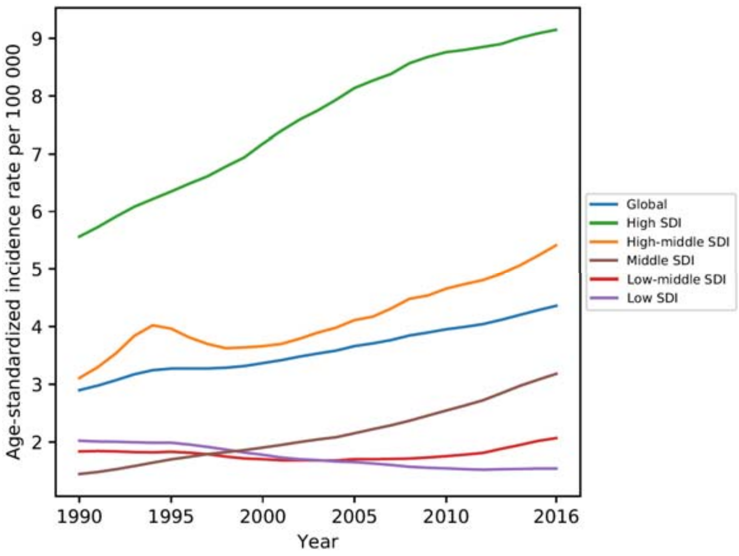

eFigure 55: Trends in Age-Standardized Incidence Rates for Thyroid Cancer, 1990-2016, Female

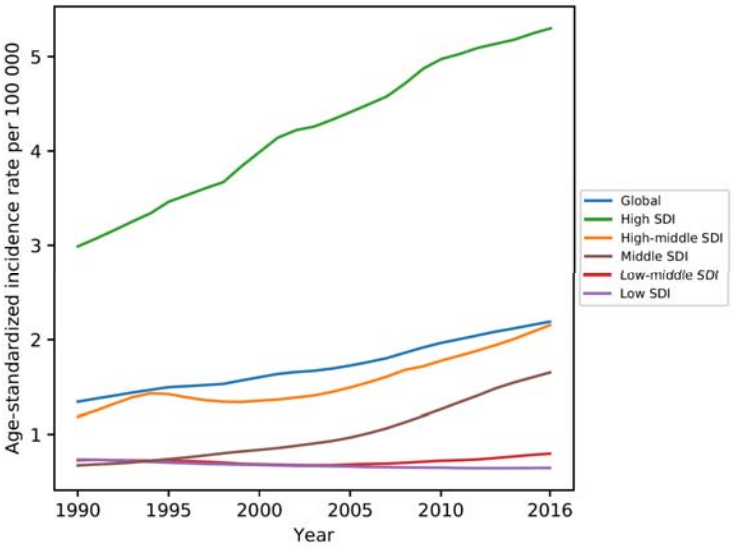

eFigure 56: Trends in Age-Standardized Incidence Rates for Thyroid Cancer, 1990-2016, Male

© 2018 Fitzmaurice C et al. JAMA Oncology.

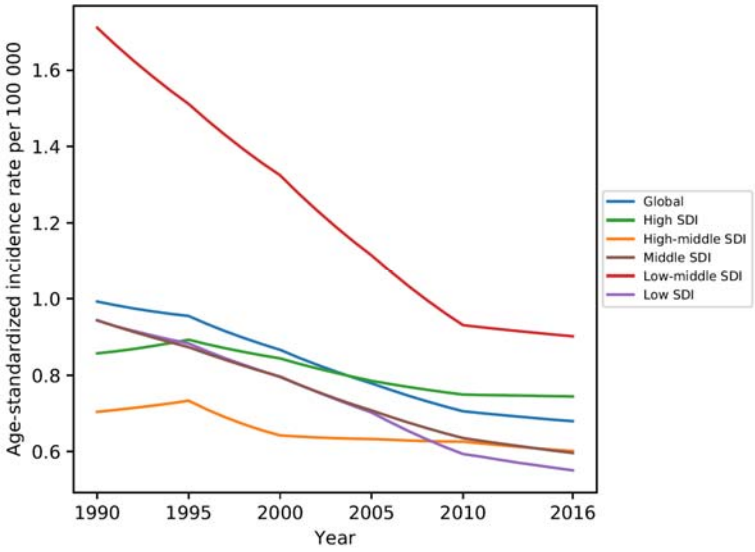

eFigure 57: Trends in Age-Standardized Incidence Rates for Larynx Cancer, 1990-2016, Female

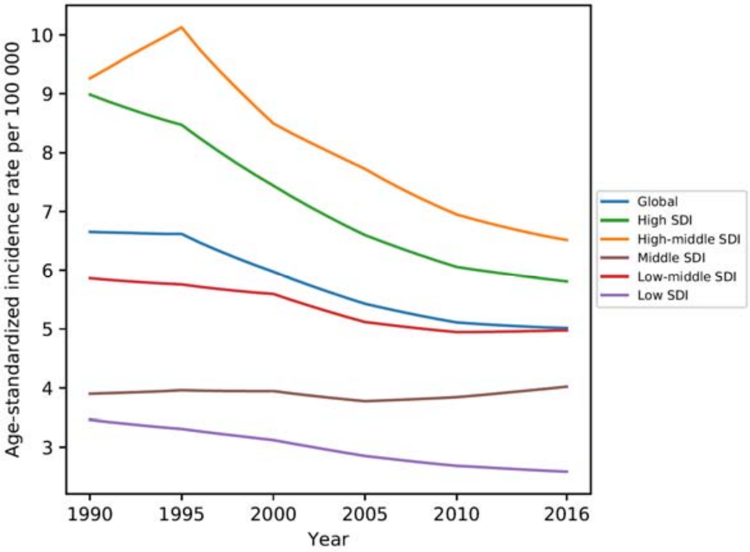

eFigure 58: Trends in Age-Standardized Incidence Rates for Larynx Cancer, 1990-2016, Male

© 2018 Fitzmaurice C et al. JAMA Oncology.

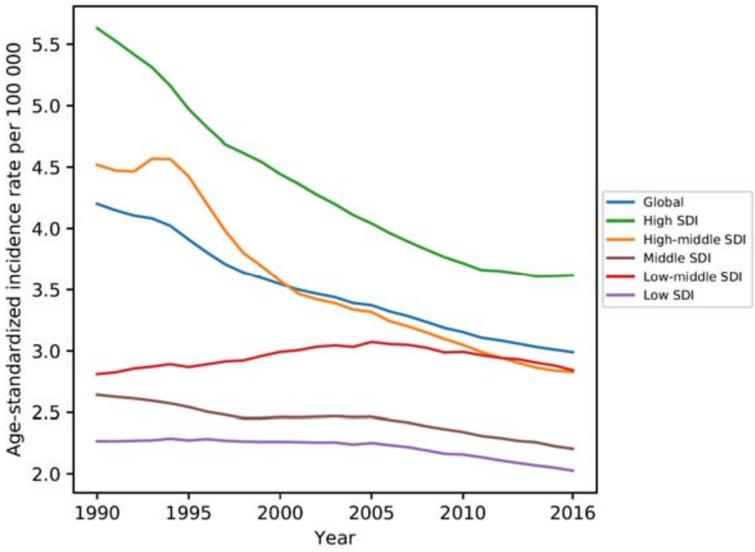

eFigure 59: Trends in Age-Standardized Incidence Rates for Gallbladder Cancer, 1990-2016, Female

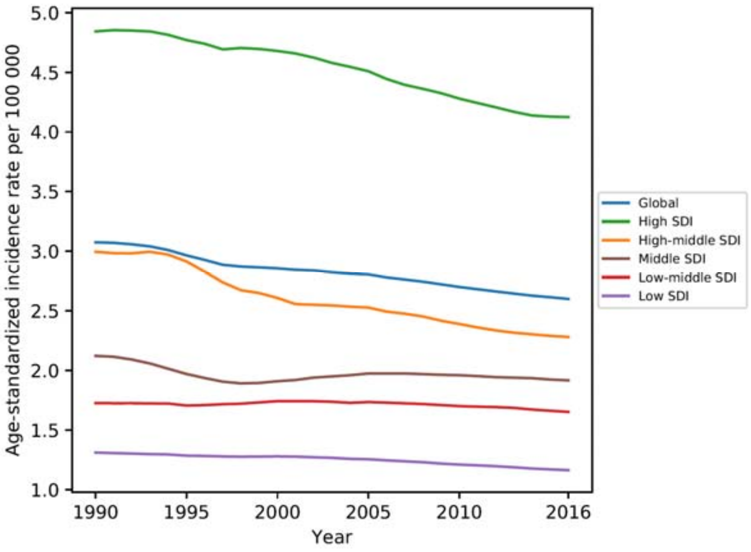

eFigure 60: Trends in Age-Standardized Incidence Rates for Gallbladder Cancer, 1990-2016, Male

© 2018 Fitzmaurice C et al. JAMA Oncology.

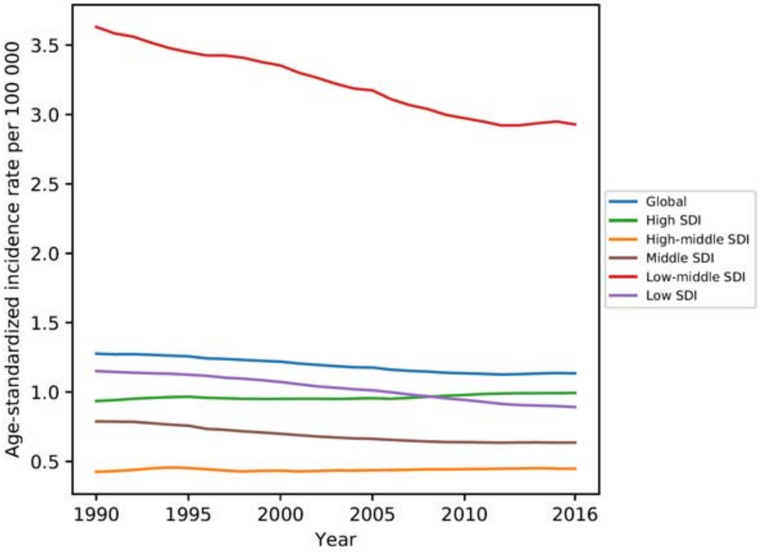

eFigure 61: Trends in Age-Standardized Incidence Rates for Other Pharynx Cancer, 1990-2016, Female

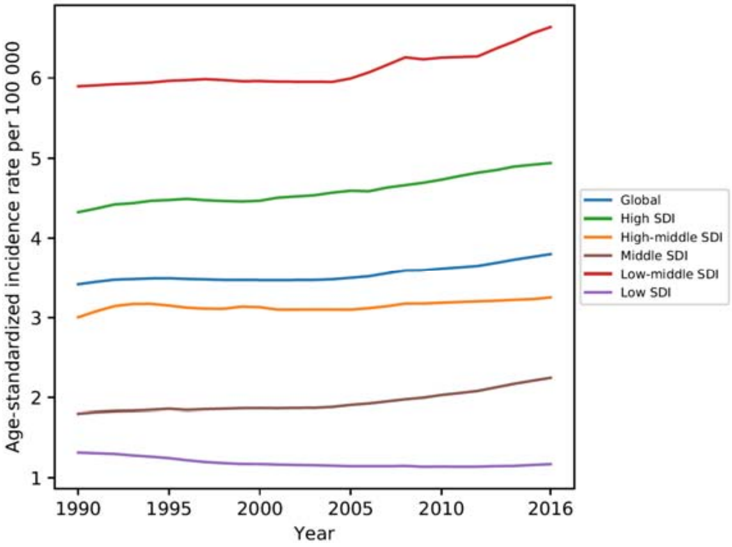

eFigure 62: Trends in Age-Standardized Incidence Rates for Other Pharynx Cancer, 1990-2016, Male

© 2018 Fitzmaurice C et al. JAMA Oncology.

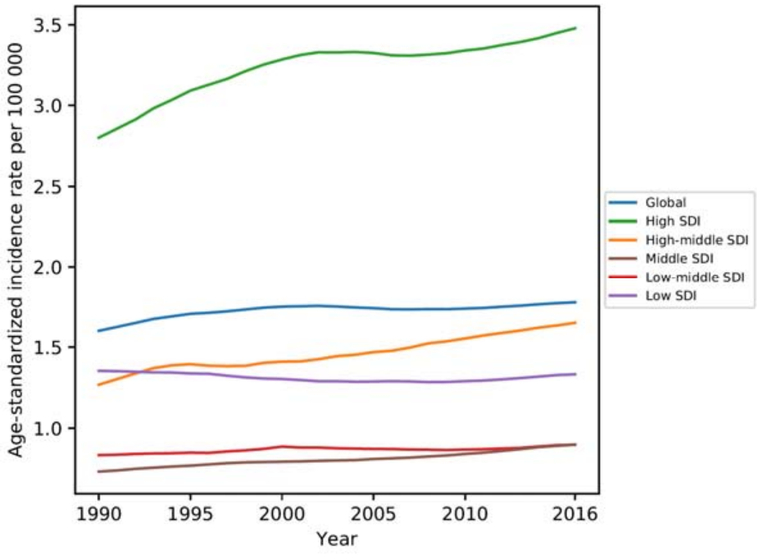

eFigure 63: Trends in Age-Standardized Incidence Rates for Multiple Myeloma, 1990-2016, Female

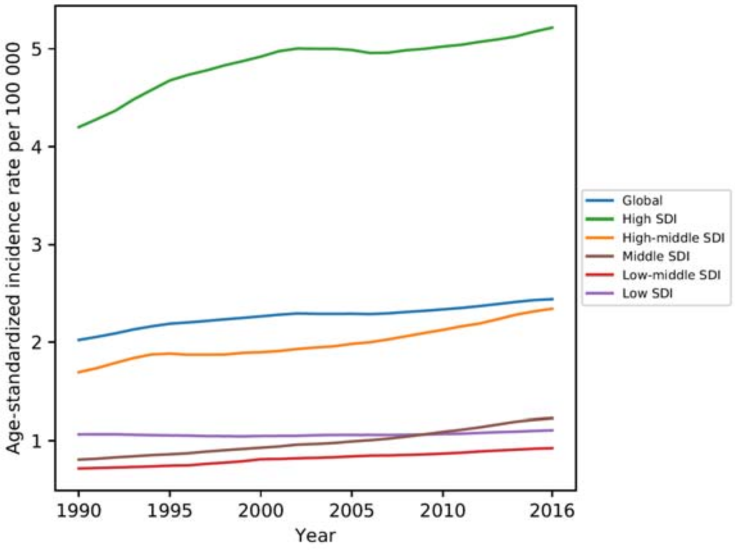

eFigure 64: Trends in Age-Standardized Incidence Rates for Multiple Myeloma, 1990-2016, Male

© 2018 Fitzmaurice C et al. JAMA Oncology.

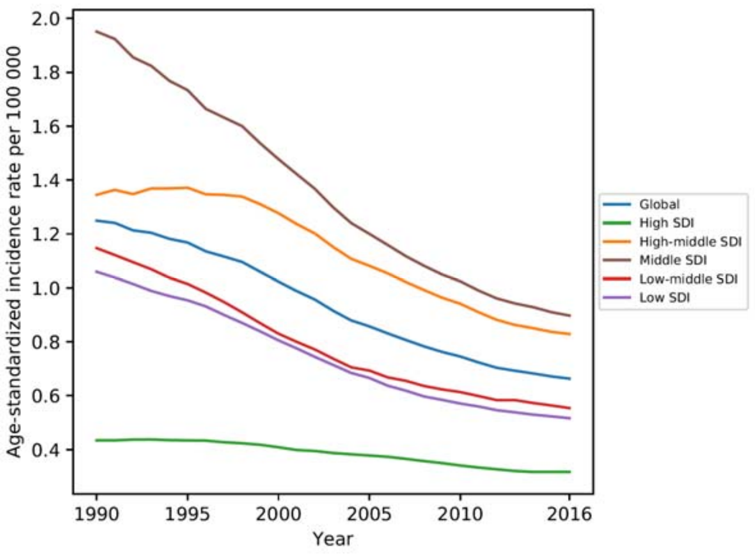

eFigure 65: Trends in Age-Standardized Incidence Rates for Nasopharynx Cancer, 1990-2016, Female

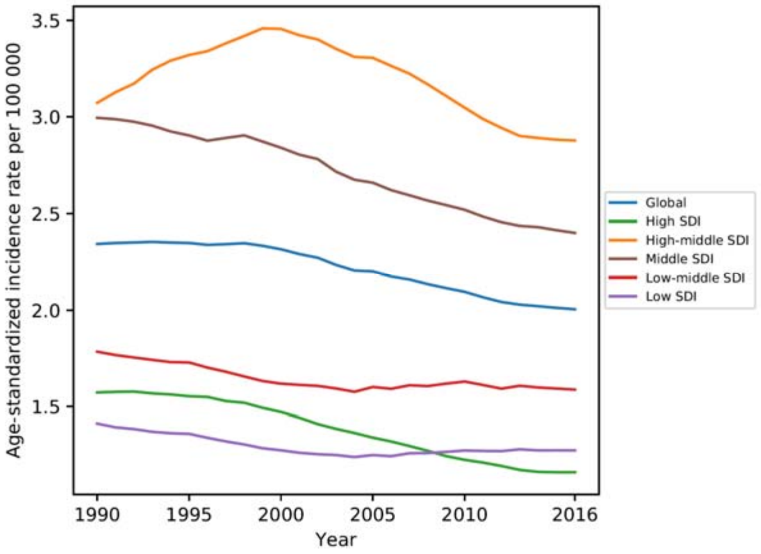

eFigure 66: Trends in Age-Standardized Incidence Rates for Nasopharynx Cancer, 1990-2016, Male

© 2018 Fitzmaurice C et al. JAMA Oncology.

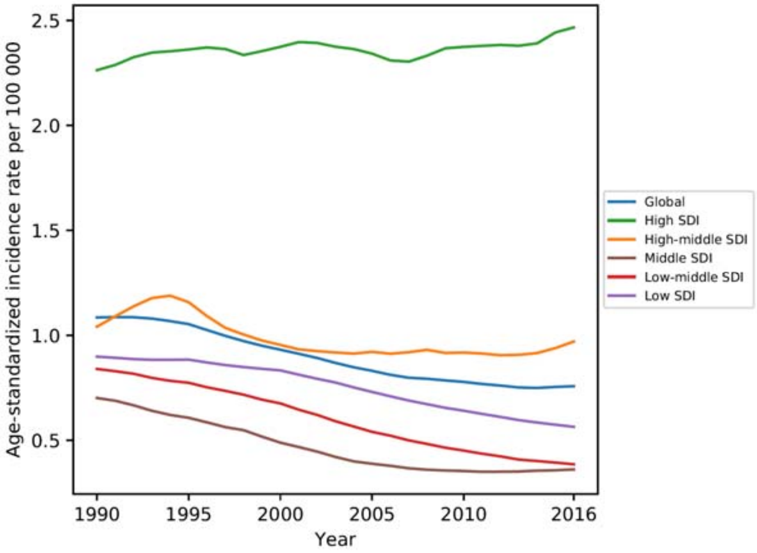

eFigure 67: Trends in Age-Standardized Incidence Rates for Hodgkin Lymphoma, 1990-2016, Female

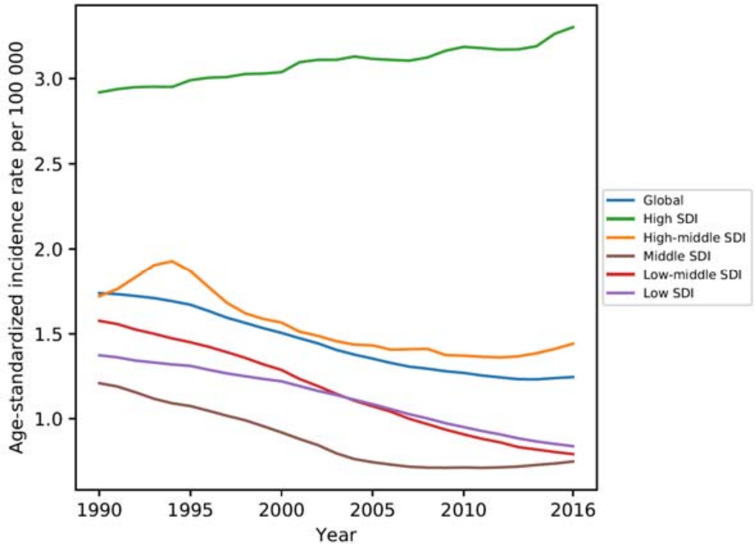

eFigure 68: Trends in Age-Standardized Incidence Rates for Hodgkin Lymphoma, 1990-2016, Male

© 2018 Fitzmaurice C et al. JAMA Oncology.

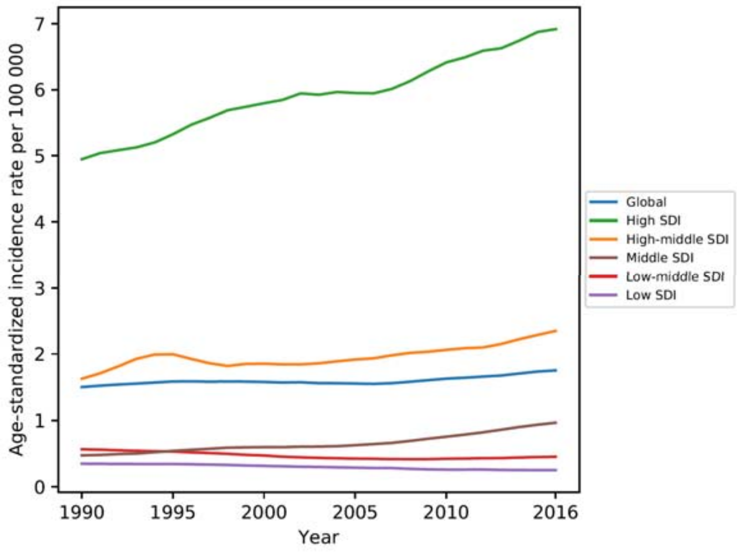

eFigure 69: Trends in Age-Standardized Incidence Rates for Testicular Cancer, 1990-2016, Male

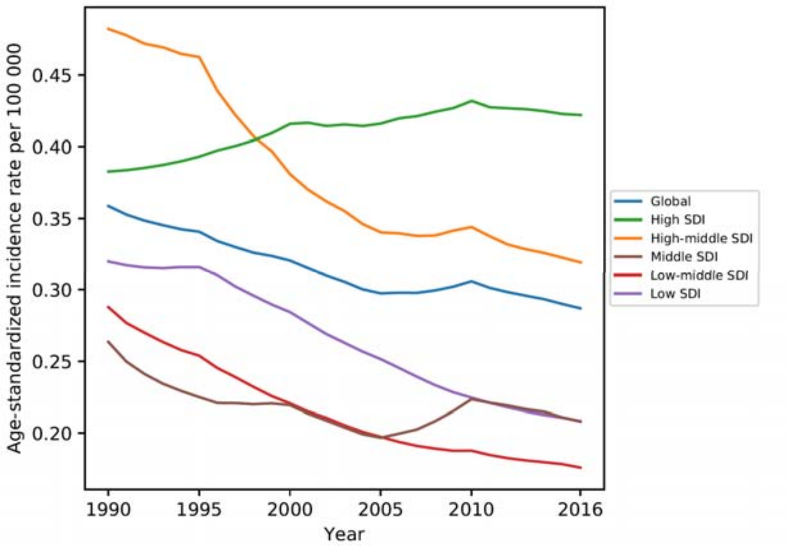

eFigure 70: Trends in Age-Standardized Incidence Rates for Mesothelioma, 1990-2016, Female

© 2018 Fitzmaurice C et al. JAMA Oncology.

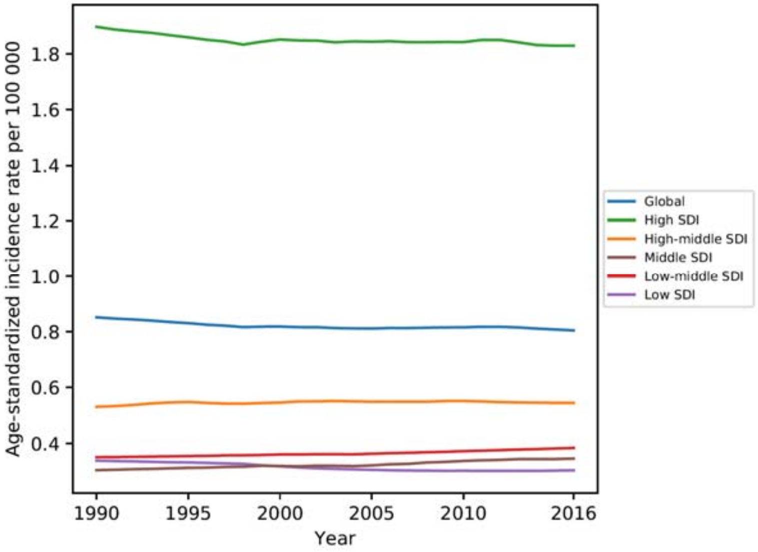

eFigure 71: Trends in Age-Standardized Incidence Rates for Mesothelioma, 1990-2016, Male

© 2018 Fitzmaurice C et al. JAMA Oncology.
